# Supplementary material for: A simple model for glioma grading based on texture analysis applied to conventional brain MRI
Source: PLoS One. 2020 May 15;15(5):e0228972. doi: 10.1371/journal.pone.0228972 (PMC7228074; doi:10.1371/journal.pone.0228972)
Supplement: S1 Table — (DOCX) [file pone.0228972.s001.docx]

**HGGs**

| **BRATS 2013** | |
| --- | --- |
| **Number** | **Key name** |
| 1H | Brats17_2013_10_1 |
| 2H | Brats17_2013_11_1 |
| 3H | Brats17_2013_12_1 |
| 4H | Brats17_2013_13_1 |
| 5H | Brats17_2013_14_1 |
| 6H | Brats17_2013_17_1 |
| 7H | Brats17_2013_18_1 |
| 8H | Brats17_2013_19_1 |
| 9H | Brats17_2013_20_1 |
| 10H | Brats17_2013_21_1 |
| 11H | Brats17_2013_22_1 |
| 12H | Brats17_2013_23_1 |
| 13H | Brats17_2013_25_1 |
| 14H | Brats17_2013_26_1 |
| 15H | Brats17_2013_27_1 |
| 16H | Brats17_2013_2_1 |
| 17H | Brats17_2013_3_1 |
| 18H | Brats17_2013_4_1 |
| 19H | Brats17_2013_5_1 |
| 20H | Brats17_2013_7_1 |

| **CBICA** | |
| --- | --- |
| **Number** | **Key name** |
| 21H | Brats17_CBICA_AAB_1 |
| 22H | Brats17_CBICA_AAG_1 |
| 23H | Brats17_CBICA_AAL_1 |
| 24H | Brats17_CBICA_AAP_1 |
| 25H | Brats17_CBICA_ABB_1 |
| 26H | Brats17_CBICA_ABE_1 |
| 27H | Brats17_CBICA_ABM_1 |
| 28H | Brats17_CBICA_ABN_1 |
| 29H | Brats17_CBICA_ABO_1 |
| 30H | Brats17_CBICA_ABY_1 |
| 31H | Brats17_CBICA_ALN_1 |
| 32H | Brats17_CBICA_ALU_1 |
| 33H | Brats17_CBICA_ALX_1 |
| 34H | Brats17_CBICA_AME_1 |
| 35H | Brats17_CBICA_AMH_1 |
| 36H | Brats17_CBICA_ANG_1 |
| 37H | Brats17_CBICA_ANI_1 |
| 38H | Brats17_CBICA_ANP_1 |
| 39H | Brats17_CBICA_ANZ_1 |
| 40H | Brats17_CBICA_AOD_1 |
| 41H | Brats17_CBICA_AOH_1 |
| 42H | Brats17_CBICA_AOO_1 |
| 43H | Brats17_CBICA_AOP_1 |
| 44H | Brats17_CBICA_AOZ_1 |
| 45H | Brats17_CBICA_APR_1 |
| 46H | Brats17_CBICA_APY_1 |
| 47H | Brats17_CBICA_APZ_1 |
| 48H | Brats17_CBICA_AQA_1 |
| 49H | Brats17_CBICA_AQD_1 |
| 50H | Brats17_CBICA_AQG_1 |
| 51H | Brats17_CBICA_AQJ_1 |
| 52H | Brats17_CBICA_AQN_1 |
| 53H | Brats17_CBICA_AQO_1 |
| 54H | Brats17_CBICA_AQP_1 |
| 55H | Brats17_CBICA_AQQ_1 |
| 56H | Brats17_CBICA_AQR_1 |
| 57H | Brats17_CBICA_AQT_1 |
| 58H | Brats17_CBICA_AQU_1 |
| 59H | Brats17_CBICA_AQV_1 |
| 60H | Brats17_CBICA_AQY_1 |
| 61H | Brats17_CBICA_AQZ_1 |
| 62H | Brats17_CBICA_ARF_1 |
| 63H | Brats17_CBICA_ARW_1 |
| 64H | Brats17_CBICA_ARZ_1 |
| 65H | Brats17_CBICA_ASA_1 |
| 66H | Brats17_CBICA_ASE_1 |
| 67H | Brats17_CBICA_ASG_1 |
| 68H | Brats17_CBICA_ASH_1 |
| 69H | Brats17_CBICA_ASK_1 |
| 70H | Brats17_CBICA_ASN_1 |
| 71H | Brats17_CBICA_ASO_1 |
| 72H | Brats17_CBICA_ASU_1 |
| 73H | Brats17_CBICA_ASV_1 |
| 74H | Brats17_CBICA_ASW_1 |
| 75H | Brats17_CBICA_ASY_1 |
| 76H | Brats17_CBICA_ATB_1 |
| 77H | Brats17_CBICA_ATD_1 |
| 78H | Brats17_CBICA_ATF_1 |
| 79H | Brats17_CBICA_ATP_1 |
| 80H | Brats17_CBICA_ATV_1 |
| 81H | Brats17_CBICA_ATX_1 |
| 82H | Brats17_CBICA_AUN_1 |
| 83H | Brats17_CBICA_AUQ_1 |
| 84H | Brats17_CBICA_AUR_1 |
| 85H | Brats17_CBICA_AVG_1 |
| 86H | Brats17_CBICA_AVJ_1 |
| 87H | Brats17_CBICA_AVV_1 |
| 88H | Brats17_CBICA_AWG_1 |
| 89H | Brats17_CBICA_AWH_1 |
| 90H | Brats17_CBICA_AWI_1 |
| 91H | Brats17_CBICA_AXJ_1 |
| 92H | Brats17_CBICA_AXL_1 |
| 93H | Brats17_CBICA_AXM_1 |
| 94H | Brats17_CBICA_AXN_1 |
| 95H | Brats17_CBICA_AXO_1 |
| 96H | Brats17_CBICA_AXQ_1 |
| 97H | Brats17_CBICA_AXW_1 |
| 98H | Brats17_CBICA_AYA_1 |
| 99H | Brats17_CBICA_AYI_1 |
| 100H | Brats17_CBICA_AYU_1 |
| 101H | Brats17_CBICA_AYW_1 |
| 102H | Brats17_CBICA_AZD_1 |
| 103H | Brats17_CBICA_AZH_1 |
| 104H | Brats17_CBICA_BFB_1 |
| 105H | Brats17_CBICA_BFP_1 |
| 106H | Brats17_CBICA_BHB_1 |
| 107H | Brats17_CBICA_BHK_1 |
| 108H | Brats17_CBICA_BHM_1 |

| **TCIA** | |
| --- | --- |
| **Number** | **Key name** |
| 109H | Brats17_TCIA_105_1 |
| 110H | Brats17_TCIA_111_1 |
| 111H | Brats17_TCIA_113_1 |
| 112H | Brats17_TCIA_117_1 |
| 113H | Brats17_TCIA_118_1 |
| 114H | Brats17_TCIA_121_1 |
| 115H | Brats17_TCIA_131_1 |
| 116H | Brats17_TCIA_133_1 |
| 117H | Brats17_TCIA_135_1 |
| 118H | Brats17_TCIA_138_1 |
| 119H | Brats17_TCIA_147_1 |
| 120H | Brats17_TCIA_149_1 |
| 121H | Brats17_TCIA_150_1 |
| 122H | Brats17_TCIA_151_1 |
| 123H | Brats17_TCIA_162_1 |
| 124H | Brats17_TCIA_165_1 |
| 125H | Brats17_TCIA_167_1 |
| 126H | Brats17_TCIA_168_1 |
| 127H | Brats17_TCIA_171_1 |
| 128H | Brats17_TCIA_179_1 |
| 129H | Brats17_TCIA_180_1 |
| 130H | Brats17_TCIA_184_1 |
| 131H | Brats17_TCIA_186_1 |
| 132H | Brats17_TCIA_190_1 |
| 133H | Brats17_TCIA_192_1 |
| 134H | Brats17_TCIA_198_1 |
| 135H | Brats17_TCIA_199_1 |
| 136H | Brats17_TCIA_201_1 |
| 137H | Brats17_TCIA_203_1 |
| 138H | Brats17_TCIA_205_1 |
| 139H | Brats17_TCIA_208_1 |
| 140H | Brats17_TCIA_211_1 |
| 141H | Brats17_TCIA_218_1 |
| 142H | Brats17_TCIA_221_1 |
| 143H | Brats17_TCIA_222_1 |
| 144H | Brats17_TCIA_226_1 |
| 145H | Brats17_TCIA_231_1 |
| 146H | Brats17_TCIA_234_1 |
| 147H | Brats17_TCIA_235_1 |
| 148H | Brats17_TCIA_242_1 |
| 149H | Brats17_TCIA_247_1 |
| 150H | Brats17_TCIA_257_1 |
| 151H | Brats17_TCIA_265_1 |
| 152H | Brats17_TCIA_274_1 |
| 153H | Brats17_TCIA_277_1 |
| 154H | Brats17_TCIA_278_1 |
| 155H | Brats17_TCIA_280_1 |
| 156H | Brats17_TCIA_283_1 |
| 157H | Brats17_TCIA_290_1 |
| 158H | Brats17_TCIA_296_1 |
| 159H | Brats17_TCIA_300_1 |
| 160H | Brats17_TCIA_309_1 |
| 161H | Brats17_TCIA_314_1 |
| 162H | Brats17_TCIA_319_1 |
| 163H | Brats17_TCIA_321_1 |
| 164H | Brats17_TCIA_322_1 |
| 165H | Brats17_TCIA_328_1 |
| 166H | Brats17_TCIA_331_1 |
| 167H | Brats17_TCIA_332_1 |
| 168H | Brats17_TCIA_335_1 |
| 169H | Brats17_TCIA_338_1 |
| 170H | Brats17_TCIA_343_1 |
| 171H | Brats17_TCIA_361_1 |
| 172H | Brats17_TCIA_368_1 |
| 173H | Brats17_TCIA_370_1 |
| 174H | Brats17_TCIA_372_1 |
| 175H | Brats17_TCIA_374_1 |
| 176H | Brats17_TCIA_375_1 |
| 177H | Brats17_TCIA_377_1 |
| 178H | Brats17_TCIA_378_1 |
| 179H | Brats17_TCIA_390_1 |
| 180H | Brats17_TCIA_394_1 |
| 181H | Brats17_TCIA_396_1 |
| 182H | Brats17_TCIA_401_1 |
| 183H | Brats17_TCIA_406_1 |
| 184H | Brats17_TCIA_409_1 |
| 185H | Brats17_TCIA_411_1 |
| 186H | Brats17_TCIA_412_1 |
| 187H | Brats17_TCIA_419_1 |
| 188H | Brats17_TCIA_425_1 |
| 189H | Brats17_TCIA_429_1 |
| 190H | Brats17_TCIA_430_1 |
| 191H | Brats17_TCIA_436_1 |
| 192H | Brats17_TCIA_437_1 |
| 193H | Brats17_TCIA_444_1 |
| 194H | Brats17_TCIA_448_1 |
| 195H | Brats17_TCIA_455_1 |
| 196H | Brats17_TCIA_460_1 |
| 197H | Brats17_TCIA_469_1 |
| 198H | Brats17_TCIA_471_1 |
| 199H | Brats17_TCIA_473_1 |
| 200H | Brats17_TCIA_474_1 |
| 201H | Brats17_TCIA_478_1 |
| 202H | Brats17_TCIA_479_1 |
| 203H | Brats17_TCIA_491_1 |
| 204H | Brats17_TCIA_498_1 |
| 205H | Brats17_TCIA_499_1 |
| 206H | Brats17_TCIA_603_1 |
| 207H | Brats17_TCIA_605_1 |
| 208H | Brats17_TCIA_606_1 |
| 209H | Brats17_TCIA_607_1 |
| 210H | Brats17_TCIA_608_1 |

**LGGs**

| **BRATS 2013** | |
| --- | --- |
| **Number** | **Key name** |
| 1L | Brats17_2013_0_1 |
| 2L | Brats17_2013_15_1 |
| 3L | Brats17_2013_16_1 |
| 4L | Brats17_2013_1_1 |
| 5L | Brats17_2013_24_1 |
| 6L | Brats17_2013_28_1 |
| 7L | Brats17_2013_29_1 |
| 8L | Brats17_2013_6_1 |
| 9L | Brats17_2013_8_1 |
| 10L | Brats17_2013_9_1 |

| **TCIA** | |
| --- | --- |
| **Number** | **Key name** |
| 11L | Brats17_TCIA_101_1 |
| 12L | Brats17_TCIA_103_1 |
| 13L | Brats17_TCIA_109_1 |
| 14L | Brats17_TCIA_130_1 |
| 15L | Brats17_TCIA_141_1 |
| 16L | Brats17_TCIA_152_1 |
| 17L | Brats17_TCIA_175_1 |
| 18L | Brats17_TCIA_177_1 |
| 19L | Brats17_TCIA_202_1 |
| 20L | Brats17_TCIA_241_1 |
| 21L | Brats17_TCIA_249_1 |
| 22L | Brats17_TCIA_254_1 |
| 23L | Brats17_TCIA_255_1 |
| 24L | Brats17_TCIA_261_1 |
| 25L | Brats17_TCIA_266_1 |
| 26L | Brats17_TCIA_276_1 |
| 27L | Brats17_TCIA_282_1 |
| 28L | Brats17_TCIA_298_1 |
| 29L | Brats17_TCIA_299_1 |
| 30L | Brats17_TCIA_307_1 |
| 31L | Brats17_TCIA_310_1 |
| 32L | Brats17_TCIA_312_1 |
| 33L | Brats17_TCIA_325_1 |
| 34L | Brats17_TCIA_330_1 |
| 35L | Brats17_TCIA_346_1 |
| 36L | Brats17_TCIA_351_1 |
| 37L | Brats17_TCIA_387_1 |
| 38L | Brats17_TCIA_393_1 |
| 39L | Brats17_TCIA_402_1 |
| 40L | Brats17_TCIA_408_1 |
| 41L | Brats17_TCIA_410_1 |
| 42L | Brats17_TCIA_413_1 |
| 43L | Brats17_TCIA_420_1 |
| 44L | Brats17_TCIA_428_1 |
| 45L | Brats17_TCIA_442_1 |
| 46L | Brats17_TCIA_449_1 |
| 47L | Brats17_TCIA_451_1 |
| 48L | Brats17_TCIA_462_1 |
| 49L | Brats17_TCIA_466_1 |
| 50L | Brats17_TCIA_470_1 |
| 51L | Brats17_TCIA_480_1 |
| 52L | Brats17_TCIA_490_1 |
| 53L | Brats17_TCIA_493_1 |
| 54L | Brats17_TCIA_615_1 |
| 55L | Brats17_TCIA_618_1 |
| 56L | Brats17_TCIA_620_1 |
| 57L | Brats17_TCIA_621_1 |
| 58L | Brats17_TCIA_623_1 |
| 59L | Brats17_TCIA_624_1 |
| 60L | Brats17_TCIA_625_1 |
| 61L | Brats17_TCIA_628_1 |
| 62L | Brats17_TCIA_629_1 |
| 63L | Brats17_TCIA_630_1 |
| 64L | Brats17_TCIA_632_1 |
| 65L | Brats17_TCIA_633_1 |
| 66L | Brats17_TCIA_634_1 |
| 67L | Brats17_TCIA_637_1 |
| 68L | Brats17_TCIA_639_1 |
| 69L | Brats17_TCIA_640_1 |
| 70L | Brats17_TCIA_642_1 |
| 71L | Brats17_TCIA_644_1 |
| 72L | Brats17_TCIA_645_1 |
| 73L | Brats17_TCIA_650_1 |
| 74L | Brats17_TCIA_653_1 |
| 75L | Brats17_TCIA_654_1 |
