# Supplementary material for: A simple model for glioma grading based on texture analysis applied to conventional brain MRI
Source: PLoS One. 2020 May 15;15(5):e0228972. doi: 10.1371/journal.pone.0228972 (PMC7228074; doi:10.1371/journal.pone.0228972)
Supplement: S2 Table — (DOCX) [file pone.0228972.s002.docx]

| **MRI** | **HGG** | | | | | | **LGG** | | | |
| --- | --- | --- | --- | --- | --- | --- | --- | --- | --- | --- |
| T_1Gd_ | 6H | 18H | 48H | 63H | 204H | 205H | 6L | 8L | 23L | 56L |
| T_1_ | 5H | 11H | 43H | 46H | 159H | 179H | 2L | 6L | 64L | 69L |
| T_2_ | 3H | 18H | 77H | 107H | 159H | 179H | 6L | 10L | 67L | 69L |
| FLAIR | 6H | 15H | 100H | 107H | 159H | 179H | 2L | 10L | 68L | 69L |
