# Supplementary material for: A simple model for glioma grading based on texture analysis applied to conventional brain MRI
Source: PLoS One. 2020 May 15;15(5):e0228972. doi: 10.1371/journal.pone.0228972 (PMC7228074; doi:10.1371/journal.pone.0228972)
Supplement: S3 Table — (DOCX) [file pone.0228972.s003.docx]

| **TESTING SUBSET** | |
| --- | --- |
| **HGG** | **LGG** |
| 1H | 3L |
| 17H | 7L |
| 20H | 11L |
| 25H | 14L |
| 30H | 15L |
| 31H | 16L |
| 39H | 17L |
| 43H | 19L |
| 47H | 20L |
| 49H | 21L |
| 55H | 22L |
| 58H | 25L |
| 59H | 26L |
| 65H | 28L |
| 72H | 30L |
| 85H | 31L |
| 89H | 35L |
| 90H | 36L |
| 94H | 40L |
| 97H | 43L |
| 99H | 44L |
| 117H | 47L |
| 120H | 49L |
| 121H | 50L |
| 140H | 55L |
| 145H | 58L |
| 161H | 59L |
| 173H | 61L |
| 181H | 62L |
| 182H | 63L |
| 183H | 66L |
| 185H | 71L |
| 187H | 73L |
| 192H | 75L |
