# Supplementary material for: A simple model for glioma grading based on texture analysis applied to conventional brain MRI
Source: PLoS One. 2020 May 15;15(5):e0228972. doi: 10.1371/journal.pone.0228972 (PMC7228074; doi:10.1371/journal.pone.0228972)
Supplement: S4 Table — (DOCX) [file pone.0228972.s004.docx]

| **1** | 7H | 9H | 27H | 28H | 29H | 38H | 45H | 66H | 70H | 81H | 83H | 95H | 96H | 98H | 104H | 112H | 113H | 115H | 118H | 122H | 136H | 151H | 152H | 153H | 176H | 189H | 194H | 196H | 202H | 209H |
| --- | --- | --- | --- | --- | --- | --- | --- | --- | --- | --- | --- | --- | --- | --- | --- | --- | --- | --- | --- | --- | --- | --- | --- | --- | --- | --- | --- | --- | --- | --- |
|  | 1L | 4L | 5L | 9L | 12L | 13L | 18L | 24L | 27L | 29L | 32L | 33L | 34L | 37L | 38L | 39L | 41L | 42L | 45L | 46L | 48L | 51L | 52L | 53L | 57L | 60L | 65L | 70L | 72L | 74L |
|  |  |  |  |  |  |  |  |  |  |  |  |  |  |  |  |  |  |  |  |  |  |  |  |  |  |  |  |  |  |  |
| **2** | 2H | 16H | 22H | 23H | 35H | 36H | 50H | 53H | 54H | 56H | 60H | 64H | 70H | 76H | 80H | 104H | 109H | 112H | 115H | 127H | 130H | 133H | 144H | 151H | 167H | 168H | 171H | 196H | 197H | 201H |
|  | 1L | 4L | 5L | 9L | 12L | 13L | 18L | 24L | 27L | 29L | 32L | 33L | 34L | 37L | 38L | 39L | 41L | 42L | 45L | 46L | 48L | 51L | 52L | 53L | 57L | 60L | 65L | 70L | 72L | 74L |
|  |  |  |  |  |  |  |  |  |  |  |  |  |  |  |  |  |  |  |  |  |  |  |  |  |  |  |  |  |  |  |
| **3** | 27H | 28H | 33H | 36H | 45H | 61H | 64H | 71H | 75H | 87H | 92H | 98H | 103H | 109H | 113H | 114H | 122H | 130H | 131H | 138H | 146H | 162H | 163H | 164H | 172H | 174H | 186H | 198H | 207H | 210H |
|  | 1L | 4L | 5L | 9L | 12L | 13L | 18L | 24L | 27L | 29L | 32L | 33L | 34L | 37L | 38L | 39L | 41L | 42L | 45L | 46L | 48L | 51L | 52L | 53L | 57L | 60L | 65L | 70L | 72L | 74L |
|  |  |  |  |  |  |  |  |  |  |  |  |  |  |  |  |  |  |  |  |  |  |  |  |  |  |  |  |  |  |  |
| **4** | 4H | 13H | 14H | 56H | 68H | 69H | 75H | 82H | 86H | 87H | 95H | 101H | 103H | 105H | 112H | 118H | 135H | 137H | 138H | 158H | 162H | 167H | 169H | 170H | 188H | 191H | 200H | 202H | 203H | 210H |
|  | 1L | 4L | 5L | 9L | 12L | 13L | 18L | 24L | 27L | 29L | 32L | 33L | 34L | 37L | 38L | 39L | 41L | 42L | 45L | 46L | 48L | 51L | 52L | 53L | 57L | 60L | 65L | 70L | 72L | 74L |
|  |  |  |  |  |  |  |  |  |  |  |  |  |  |  |  |  |  |  |  |  |  |  |  |  |  |  |  |  |  |  |
| **5** | 2H | 19H | 23H | 26H | 29H | 32H | 37H | 40H | 42H | 45H | 56H | 61H | 69H | 73H | 81H | 83H | 93H | 104H | 105H | 109H | 118H | 125H | 127H | 148H | 164H | 190H | 195H | 201H | 207H | 210H |
|  | 1L | 4L | 5L | 9L | 12L | 13L | 18L | 24L | 27L | 29L | 32L | 33L | 34L | 37L | 38L | 39L | 41L | 42L | 45L | 46L | 48L | 51L | 52L | 53L | 57L | 60L | 65L | 70L | 72L | 74L |
|  |  |  |  |  |  |  |  |  |  |  |  |  |  |  |  |  |  |  |  |  |  |  |  |  |  |  |  |  |  |  |
| **6** | 13H | 22H | 27H | 29H | 35H | 40H | 53H | 54H | 57H | 76H | 80H | 86H | 88H | 92H | 95H | 96H | 111H | 119H | 130H | 144H | 152H | 154H | 160H | 169H | 176H | 177H | 178H | 193H | 202H | 203H |
|  | 1L | 4L | 5L | 9L | 12L | 13L | 18L | 24L | 27L | 29L | 32L | 33L | 34L | 37L | 38L | 39L | 41L | 42L | 45L | 46L | 48L | 51L | 52L | 53L | 57L | 60L | 65L | 70L | 72L | 74L |
|  |  |  |  |  |  |  |  |  |  |  |  |  |  |  |  |  |  |  |  |  |  |  |  |  |  |  |  |  |  |  |
| **7** | 12H | 14H | 16H | 22H | 24H | 26H | 53H | 54H | 56H | 67H | 79H | 80H | 84H | 91H | 93H | 102H | 108H | 122H | 123H | 125H | 130H | 135H | 138H | 158H | 163H | 168H | 170H | 174H | 176H | 177H |
|  | 1L | 4L | 5L | 9L | 12L | 13L | 18L | 24L | 27L | 29L | 32L | 33L | 34L | 37L | 38L | 39L | 41L | 42L | 45L | 46L | 48L | 51L | 52L | 53L | 57L | 60L | 65L | 70L | 72L | 74L |
|  |  |  |  |  |  |  |  |  |  |  |  |  |  |  |  |  |  |  |  |  |  |  |  |  |  |  |  |  |  |  |
| **8** | 9H | 14H | 28H | 33H | 37H | 38H | 53H | 57H | 62H | 71H | 87H | 95H | 102H | 105H | 110H | 119H | 123H | 126H | 129H | 134H | 137H | 146H | 147H | 157H | 167H | 176H | 193H | 194H | 197H | 201H |
|  | 1L | 4L | 5L | 9L | 12L | 13L | 18L | 24L | 27L | 29L | 32L | 33L | 34L | 37L | 38L | 39L | 41L | 42L | 45L | 46L | 48L | 51L | 52L | 53L | 57L | 60L | 65L | 70L | 72L | 74L |
|  |  |  |  |  |  |  |  |  |  |  |  |  |  |  |  |  |  |  |  |  |  |  |  |  |  |  |  |  |  |  |
| **9** | 2H | 26H | 27H | 37H | 44H | 50H | 73H | 78H | 81H | 86H | 92H | 102H | 114H | 115H | 118H | 129H | 131H | 136H | 139H | 146H | 148H | 149H | 156H | 158H | 167H | 171H | 174H | 175H | 178H | 201H |
|  | 1L | 4L | 5L | 9L | 12L | 13L | 18L | 24L | 27L | 29L | 32L | 33L | 34L | 37L | 38L | 39L | 41L | 42L | 45L | 46L | 48L | 51L | 52L | 53L | 57L | 60L | 65L | 70L | 72L | 74L |
|  |  |  |  |  |  |  |  |  |  |  |  |  |  |  |  |  |  |  |  |  |  |  |  |  |  |  |  |  |  |  |
| **10** | 22H | 23H | 26H | 28H | 32H | 40H | 44H | 51H | 56H | 69H | 79H | 87H | 88H | 96H | 101H | 104H | 110H | 116H | 118H | 127H | 129H | 132H | 134H | 149H | 156H | 172H | 174H | 178H | 188H | 209H |
|  | 1L | 4L | 5L | 9L | 12L | 13L | 18L | 24L | 27L | 29L | 32L | 33L | 34L | 37L | 38L | 39L | 41L | 42L | 45L | 46L | 48L | 51L | 52L | 53L | 57L | 60L | 65L | 70L | 72L | 74L |
|  |  |  |  |  |  |  |  |  |  |  |  |  |  |  |  |  |  |  |  |  |  |  |  |  |  |  |  |  |  |  |
| **11** | 12H | 19H | 22H | 24H | 27H | 36H | 37H | 38H | 44H | 54H | 68H | 73H | 78H | 109H | 111H | 118H | 124H | 128H | 135H | 154H | 158H | 162H | 163H | 164H | 176H | 189H | 191H | 194H | 197H | 210H |
|  | 1L | 4L | 5L | 9L | 12L | 13L | 18L | 24L | 27L | 29L | 32L | 33L | 34L | 37L | 38L | 39L | 41L | 42L | 45L | 46L | 48L | 51L | 52L | 53L | 57L | 60L | 65L | 70L | 72L | 74L |
|  |  |  |  |  |  |  |  |  |  |  |  |  |  |  |  |  |  |  |  |  |  |  |  |  |  |  |  |  |  |  |
| **12** | 4H | 21H | 27H | 52H | 64H | 74H | 79H | 83H | 88H | 95H | 104H | 123H | 124H | 131H | 135H | 141H | 144H | 147H | 148H | 152H | 153H | 156H | 157H | 163H | 170H | 172H | 186H | 189H | 191H | 206H |
|  | 1L | 4L | 5L | 9L | 12L | 13L | 18L | 24L | 27L | 29L | 32L | 33L | 34L | 37L | 38L | 39L | 41L | 42L | 45L | 46L | 48L | 51L | 52L | 53L | 57L | 60L | 65L | 70L | 72L | 74L |
|  |  |  |  |  |  |  |  |  |  |  |  |  |  |  |  |  |  |  |  |  |  |  |  |  |  |  |  |  |  |  |
| **13** | 2H | 8H | 13H | 27H | 57H | 86H | 92H | 93H | 101H | 104H | 108H | 109H | 116H | 127H | 128H | 138H | 139H | 142H | 146H | 147H | 154H | 166H | 167H | 174H | 175H | 186H | 191H | 198H | 201H | 208H |
|  | 1L | 4L | 5L | 9L | 12L | 13L | 18L | 24L | 27L | 29L | 32L | 33L | 34L | 37L | 38L | 39L | 41L | 42L | 45L | 46L | 48L | 51L | 52L | 53L | 57L | 60L | 65L | 70L | 72L | 74L |
|  |  |  |  |  |  |  |  |  |  |  |  |  |  |  |  |  |  |  |  |  |  |  |  |  |  |  |  |  |  |  |
| **14** | 19H | 21H | 24H | 34H | 37H | 44H | 54H | 68H | 73H | 78H | 86H | 92H | 93H | 95H | 113H | 127H | 132H | 134H | 139H | 142H | 147H | 150H | 151H | 160H | 169H | 175H | 190H | 196H | 202H | 210H |
|  | 1L | 4L | 5L | 9L | 12L | 13L | 18L | 24L | 27L | 29L | 32L | 33L | 34L | 37L | 38L | 39L | 41L | 42L | 45L | 46L | 48L | 51L | 52L | 53L | 57L | 60L | 65L | 70L | 72L | 74L |
|  |  |  |  |  |  |  |  |  |  |  |  |  |  |  |  |  |  |  |  |  |  |  |  |  |  |  |  |  |  |  |
| **15** | 23H | 33H | 42H | 57H | 66H | 82H | 83H | 95H | 103H | 110H | 112H | 113H | 115H | 122H | 129H | 130H | 131H | 133H | 137H | 141H | 147H | 153H | 154H | 155H | 160H | 172H | 174H | 177H | 191H | 195H |
|  | 1L | 4L | 5L | 9L | 12L | 13L | 18L | 24L | 27L | 29L | 32L | 33L | 34L | 37L | 38L | 39L | 41L | 42L | 45L | 46L | 48L | 51L | 52L | 53L | 57L | 60L | 65L | 70L | 72L | 74L |
|  |  |  |  |  |  |  |  |  |  |  |  |  |  |  |  |  |  |  |  |  |  |  |  |  |  |  |  |  |  |  |
| **16** | 7H | 8H | 26H | 37H | 41H | 42H | 56H | 60H | 66H | 70H | 74H | 86H | 87H | 98H | 104H | 108H | 116H | 128H | 129H | 132H | 136H | 139H | 142H | 147H | 158H | 164H | 172H | 174H | 188H | 201H |
|  | 1L | 4L | 5L | 9L | 12L | 13L | 18L | 24L | 27L | 29L | 32L | 33L | 34L | 37L | 38L | 39L | 41L | 42L | 45L | 46L | 48L | 51L | 52L | 53L | 57L | 60L | 65L | 70L | 72L | 74L |
|  |  |  |  |  |  |  |  |  |  |  |  |  |  |  |  |  |  |  |  |  |  |  |  |  |  |  |  |  |  |  |
| **17** | 4H | 9H | 14H | 23H | 37H | 40H | 53H | 60H | 61H | 69H | 71H | 88H | 103H | 114H | 124H | 139H | 149H | 151H | 157H | 162H | 176H | 190H | 191H | 195H | 198H | 199H | 200H | 207H | 208H | 210H |
|  | 1L | 4L | 5L | 9L | 12L | 13L | 18L | 24L | 27L | 29L | 32L | 33L | 34L | 37L | 38L | 39L | 41L | 42L | 45L | 46L | 48L | 51L | 52L | 53L | 57L | 60L | 65L | 70L | 72L | 74L |
|  |  |  |  |  |  |  |  |  |  |  |  |  |  |  |  |  |  |  |  |  |  |  |  |  |  |  |  |  |  |  |
| **18** | 2H | 14H | 23H | 34H | 36H | 44H | 53H | 69H | 71H | 78H | 80H | 98H | 105H | 109H | 115H | 116H | 122H | 125H | 127H | 128H | 131H | 139H | 143H | 150H | 172H | 177H | 189H | 199H | 201H | 203H |
|  | 1L | 4L | 5L | 9L | 12L | 13L | 18L | 24L | 27L | 29L | 32L | 33L | 34L | 37L | 38L | 39L | 41L | 42L | 45L | 46L | 48L | 51L | 52L | 53L | 57L | 60L | 65L | 70L | 72L | 74L |
|  |  |  |  |  |  |  |  |  |  |  |  |  |  |  |  |  |  |  |  |  |  |  |  |  |  |  |  |  |  |  |
| **19** | 7H | 13H | 22H | 35H | 50H | 60H | 73H | 74H | 75H | 86H | 92H | 96H | 102H | 103H | 108H | 113H | 114H | 130H | 136H | 139H | 142H | 148H | 154H | 162H | 165H | 170H | 194H | 199H | 200H | 206H |
|  | 1L | 4L | 5L | 9L | 12L | 13L | 18L | 24L | 27L | 29L | 32L | 33L | 34L | 37L | 38L | 39L | 41L | 42L | 45L | 46L | 48L | 51L | 52L | 53L | 57L | 60L | 65L | 70L | 72L | 74L |
|  |  |  |  |  |  |  |  |  |  |  |  |  |  |  |  |  |  |  |  |  |  |  |  |  |  |  |  |  |  |  |
| **20** | 16H | 33H | 34H | 41H | 42H | 44H | 50H | 66H | 76H | 78H | 93H | 104H | 108H | 113H | 114H | 119H | 125H | 126H | 127H | 131H | 142H | 156H | 169H | 170H | 171H | 172H | 189H | 196H | 199H | 206H |
|  | 1L | 4L | 5L | 9L | 12L | 13L | 18L | 24L | 27L | 29L | 32L | 33L | 34L | 37L | 38L | 39L | 41L | 42L | 45L | 46L | 48L | 51L | 52L | 53L | 57L | 60L | 65L | 70L | 72L | 74L |
|  |  |  |  |  |  |  |  |  |  |  |  |  |  |  |  |  |  |  |  |  |  |  |  |  |  |  |  |  |  |  |
| **21** | 7H | 12H | 14H | 19H | 21H | 34H | 35H | 42H | 80H | 84H | 88H | 105H | 108H | 110H | 113H | 124H | 126H | 131H | 137H | 157H | 175H | 176H | 186H | 191H | 193H | 194H | 195H | 198H | 203H | 207H |
|  | 1L | 4L | 5L | 9L | 12L | 13L | 18L | 24L | 27L | 29L | 32L | 33L | 34L | 37L | 38L | 39L | 41L | 42L | 45L | 46L | 48L | 51L | 52L | 53L | 57L | 60L | 65L | 70L | 72L | 74L |
|  |  |  |  |  |  |  |  |  |  |  |  |  |  |  |  |  |  |  |  |  |  |  |  |  |  |  |  |  |  |  |
| **22** | 8H | 19H | 21H | 32H | 35H | 42H | 50H | 51H | 52H | 69H | 79H | 81H | 91H | 105H | 109H | 111H | 116H | 133H | 137H | 143H | 154H | 156H | 160H | 165H | 167H | 175H | 176H | 186H | 188H | 202H |
|  | 1L | 4L | 5L | 9L | 12L | 13L | 18L | 24L | 27L | 29L | 32L | 33L | 34L | 37L | 38L | 39L | 41L | 42L | 45L | 46L | 48L | 51L | 52L | 53L | 57L | 60L | 65L | 70L | 72L | 74L |
|  |  |  |  |  |  |  |  |  |  |  |  |  |  |  |  |  |  |  |  |  |  |  |  |  |  |  |  |  |  |  |
| **23** | 9H | 14H | 28H | 34H | 42H | 62H | 70H | 73H | 78H | 88H | 91H | 104H | 127H | 134H | 137H | 148H | 155H | 156H | 157H | 160H | 165H | 167H | 168H | 171H | 178H | 193H | 194H | 198H | 203H | 208H |
|  | 1L | 4L | 5L | 9L | 12L | 13L | 18L | 24L | 27L | 29L | 32L | 33L | 34L | 37L | 38L | 39L | 41L | 42L | 45L | 46L | 48L | 51L | 52L | 53L | 57L | 60L | 65L | 70L | 72L | 74L |
|  |  |  |  |  |  |  |  |  |  |  |  |  |  |  |  |  |  |  |  |  |  |  |  |  |  |  |  |  |  |  |
| **24** | 9H | 16H | 27H | 28H | 29H | 61H | 74H | 76H | 83H | 88H | 102H | 103H | 108H | 111H | 130H | 131H | 139H | 147H | 148H | 154H | 167H | 168H | 169H | 174H | 176H | 191H | 196H | 197H | 198H | 207H |
|  | 1L | 4L | 5L | 9L | 12L | 13L | 18L | 24L | 27L | 29L | 32L | 33L | 34L | 37L | 38L | 39L | 41L | 42L | 45L | 46L | 48L | 51L | 52L | 53L | 57L | 60L | 65L | 70L | 72L | 74L |
|  |  |  |  |  |  |  |  |  |  |  |  |  |  |  |  |  |  |  |  |  |  |  |  |  |  |  |  |  |  |  |
| **25** | 28H | 32H | 37H | 53H | 73H | 79H | 91H | 103H | 110H | 116H | 123H | 124H | 131H | 133H | 134H | 137H | 142H | 144H | 146H | 154H | 156H | 164H | 165H | 168H | 176H | 177H | 196H | 199H | 206H | 208H |
|  | 1L | 4L | 5L | 9L | 12L | 13L | 18L | 24L | 27L | 29L | 32L | 33L | 34L | 37L | 38L | 39L | 41L | 42L | 45L | 46L | 48L | 51L | 52L | 53L | 57L | 60L | 65L | 70L | 72L | 74L |
|  |  |  |  |  |  |  |  |  |  |  |  |  |  |  |  |  |  |  |  |  |  |  |  |  |  |  |  |  |  |  |
| **26** | 13H | 21H | 27H | 29H | 36H | 51H | 54H | 67H | 69H | 76H | 84H | 92H | 105H | 114H | 122H | 125H | 129H | 134H | 136H | 144H | 147H | 160H | 163H | 166H | 177H | 186H | 188H | 194H | 202H | 203H |
|  | 1L | 4L | 5L | 9L | 12L | 13L | 18L | 24L | 27L | 29L | 32L | 33L | 34L | 37L | 38L | 39L | 41L | 42L | 45L | 46L | 48L | 51L | 52L | 53L | 57L | 60L | 65L | 70L | 72L | 74L |
|  |  |  |  |  |  |  |  |  |  |  |  |  |  |  |  |  |  |  |  |  |  |  |  |  |  |  |  |  |  |  |
| **27** | 7H | 21H | 36H | 61H | 71H | 76H | 78H | 79H | 93H | 110H | 112H | 114H | 119H | 134H | 138H | 141H | 149H | 157H | 160H | 162H | 170H | 172H | 175H | 176H | 177H | 178H | 184H | 190H | 194H | 199H |
|  | 1L | 4L | 5L | 9L | 12L | 13L | 18L | 24L | 27L | 29L | 32L | 33L | 34L | 37L | 38L | 39L | 41L | 42L | 45L | 46L | 48L | 51L | 52L | 53L | 57L | 60L | 65L | 70L | 72L | 74L |
|  |  |  |  |  |  |  |  |  |  |  |  |  |  |  |  |  |  |  |  |  |  |  |  |  |  |  |  |  |  |  |
| **28** | 4H | 8H | 12H | 13H | 19H | 26H | 44H | 50H | 53H | 56H | 68H | 71H | 80H | 84H | 92H | 101H | 112H | 116H | 128H | 137H | 155H | 160H | 163H | 166H | 167H | 170H | 190H | 194H | 198H | 203H |
|  | 1L | 4L | 5L | 9L | 12L | 13L | 18L | 24L | 27L | 29L | 32L | 33L | 34L | 37L | 38L | 39L | 41L | 42L | 45L | 46L | 48L | 51L | 52L | 53L | 57L | 60L | 65L | 70L | 72L | 74L |
|  |  |  |  |  |  |  |  |  |  |  |  |  |  |  |  |  |  |  |  |  |  |  |  |  |  |  |  |  |  |  |
| **29** | 13H | 14H | 22H | 26H | 27H | 32H | 33H | 37H | 42H | 51H | 60H | 61H | 62H | 66H | 95H | 109H | 112H | 118H | 119H | 122H | 131H | 134H | 136H | 141H | 142H | 146H | 151H | 152H | 167H | 207H |
|  | 1L | 4L | 5L | 9L | 12L | 13L | 18L | 24L | 27L | 29L | 32L | 33L | 34L | 37L | 38L | 39L | 41L | 42L | 45L | 46L | 48L | 51L | 52L | 53L | 57L | 60L | 65L | 70L | 72L | 74L |
|  |  |  |  |  |  |  |  |  |  |  |  |  |  |  |  |  |  |  |  |  |  |  |  |  |  |  |  |  |  |  |
| **30** | 2H | 27H | 37H | 38H | 56H | 71H | 73H | 78H | 84H | 88H | 108H | 112H | 124H | 128H | 130H | 134H | 135H | 136H | 137H | 139H | 143H | 149H | 154H | 157H | 163H | 168H | 170H | 175H | 186H | 196H |
|  | 1L | 4L | 5L | 9L | 12L | 13L | 18L | 24L | 27L | 29L | 32L | 33L | 34L | 37L | 38L | 39L | 41L | 42L | 45L | 46L | 48L | 51L | 52L | 53L | 57L | 60L | 65L | 70L | 72L | 74L |
|  |  |  |  |  |  |  |  |  |  |  |  |  |  |  |  |  |  |  |  |  |  |  |  |  |  |  |  |  |  |  |
| **31** | 8H | 12H | 13H | 38H | 44H | 45H | 53H | 56H | 73H | 86H | 87H | 92H | 104H | 114H | 122H | 123H | 130H | 135H | 148H | 149H | 154H | 158H | 160H | 165H | 166H | 168H | 174H | 197H | 200H | 203H |
|  | 1L | 4L | 5L | 9L | 12L | 13L | 18L | 24L | 27L | 29L | 32L | 33L | 34L | 37L | 38L | 39L | 41L | 42L | 45L | 46L | 48L | 51L | 52L | 53L | 57L | 60L | 65L | 70L | 72L | 74L |
|  |  |  |  |  |  |  |  |  |  |  |  |  |  |  |  |  |  |  |  |  |  |  |  |  |  |  |  |  |  |  |
| **32** | 2H | 9H | 14H | 28H | 40H | 44H | 45H | 50H | 51H | 53H | 54H | 66H | 76H | 80H | 93H | 111H | 115H | 128H | 134H | 149H | 160H | 162H | 168H | 172H | 186H | 189H | 199H | 202H | 206H | 207H |
|  | 1L | 4L | 5L | 9L | 12L | 13L | 18L | 24L | 27L | 29L | 32L | 33L | 34L | 37L | 38L | 39L | 41L | 42L | 45L | 46L | 48L | 51L | 52L | 53L | 57L | 60L | 65L | 70L | 72L | 74L |
|  |  |  |  |  |  |  |  |  |  |  |  |  |  |  |  |  |  |  |  |  |  |  |  |  |  |  |  |  |  |  |
| **33** | 19H | 24H | 45H | 57H | 60H | 64H | 71H | 75H | 78H | 80H | 82H | 84H | 86H | 88H | 91H | 98H | 116H | 126H | 139H | 141H | 144H | 146H | 151H | 166H | 167H | 188H | 190H | 195H | 196H | 202H |
|  | 1L | 4L | 5L | 9L | 12L | 13L | 18L | 24L | 27L | 29L | 32L | 33L | 34L | 37L | 38L | 39L | 41L | 42L | 45L | 46L | 48L | 51L | 52L | 53L | 57L | 60L | 65L | 70L | 72L | 74L |
|  |  |  |  |  |  |  |  |  |  |  |  |  |  |  |  |  |  |  |  |  |  |  |  |  |  |  |  |  |  |  |
| **34** | 4H | 7H | 8H | 13H | 21H | 23H | 37H | 40H | 44H | 52H | 62H | 66H | 73H | 75H | 76H | 88H | 109H | 111H | 112H | 128H | 137H | 146H | 149H | 155H | 160H | 165H | 177H | 188H | 193H | 200H |
|  | 1L | 4L | 5L | 9L | 12L | 13L | 18L | 24L | 27L | 29L | 32L | 33L | 34L | 37L | 38L | 39L | 41L | 42L | 45L | 46L | 48L | 51L | 52L | 53L | 57L | 60L | 65L | 70L | 72L | 74L |
|  |  |  |  |  |  |  |  |  |  |  |  |  |  |  |  |  |  |  |  |  |  |  |  |  |  |  |  |  |  |  |
| **35** | 2H | 16H | 22H | 28H | 34H | 57H | 61H | 67H | 80H | 92H | 93H | 95H | 108H | 109H | 111H | 116H | 130H | 139H | 141H | 151H | 156H | 157H | 162H | 163H | 164H | 167H | 171H | 186H | 195H | 199H |
|  | 1L | 4L | 5L | 9L | 12L | 13L | 18L | 24L | 27L | 29L | 32L | 33L | 34L | 37L | 38L | 39L | 41L | 42L | 45L | 46L | 48L | 51L | 52L | 53L | 57L | 60L | 65L | 70L | 72L | 74L |
|  |  |  |  |  |  |  |  |  |  |  |  |  |  |  |  |  |  |  |  |  |  |  |  |  |  |  |  |  |  |  |
| **36** | 14H | 21H | 36H | 42H | 45H | 54H | 56H | 57H | 60H | 61H | 64H | 73H | 76H | 78H | 86H | 101H | 105H | 109H | 118H | 119H | 127H | 132H | 138H | 139H | 152H | 163H | 166H | 190H | 198H | 208H |
|  | 1L | 4L | 5L | 9L | 12L | 13L | 18L | 24L | 27L | 29L | 32L | 33L | 34L | 37L | 38L | 39L | 41L | 42L | 45L | 46L | 48L | 51L | 52L | 53L | 57L | 60L | 65L | 70L | 72L | 74L |
|  |  |  |  |  |  |  |  |  |  |  |  |  |  |  |  |  |  |  |  |  |  |  |  |  |  |  |  |  |  |  |
| **37** | 2H | 7H | 26H | 34H | 36H | 51H | 57H | 60H | 68H | 70H | 78H | 84H | 88H | 123H | 128H | 129H | 130H | 131H | 146H | 151H | 155H | 156H | 157H | 160H | 162H | 174H | 193H | 201H | 206H | 207H |
|  | 1L | 4L | 5L | 9L | 12L | 13L | 18L | 24L | 27L | 29L | 32L | 33L | 34L | 37L | 38L | 39L | 41L | 42L | 45L | 46L | 48L | 51L | 52L | 53L | 57L | 60L | 65L | 70L | 72L | 74L |
|  |  |  |  |  |  |  |  |  |  |  |  |  |  |  |  |  |  |  |  |  |  |  |  |  |  |  |  |  |  |  |
| **38** | 7H | 9H | 22H | 24H | 33H | 34H | 53H | 60H | 68H | 73H | 74H | 80H | 126H | 137H | 139H | 147H | 150H | 153H | 158H | 166H | 170H | 177H | 184H | 186H | 188H | 189H | 193H | 198H | 203H | 208H |
|  | 1L | 4L | 5L | 9L | 12L | 13L | 18L | 24L | 27L | 29L | 32L | 33L | 34L | 37L | 38L | 39L | 41L | 42L | 45L | 46L | 48L | 51L | 52L | 53L | 57L | 60L | 65L | 70L | 72L | 74L |
|  |  |  |  |  |  |  |  |  |  |  |  |  |  |  |  |  |  |  |  |  |  |  |  |  |  |  |  |  |  |  |
| **39** | 2H | 16H | 27H | 50H | 52H | 53H | 66H | 69H | 70H | 71H | 80H | 93H | 105H | 108H | 115H | 123H | 132H | 133H | 143H | 146H | 150H | 162H | 170H | 178H | 194H | 201H | 202H | 206H | 209H | 210H |
|  | 1L | 4L | 5L | 9L | 12L | 13L | 18L | 24L | 27L | 29L | 32L | 33L | 34L | 37L | 38L | 39L | 41L | 42L | 45L | 46L | 48L | 51L | 52L | 53L | 57L | 60L | 65L | 70L | 72L | 74L |
|  |  |  |  |  |  |  |  |  |  |  |  |  |  |  |  |  |  |  |  |  |  |  |  |  |  |  |  |  |  |  |
| **40** | 13H | 19H | 22H | 23H | 24H | 26H | 37H | 38H | 40H | 51H | 60H | 76H | 83H | 86H | 87H | 93H | 102H | 104H | 112H | 116H | 138H | 141H | 143H | 154H | 164H | 165H | 167H | 189H | 191H | 198H |
|  | 1L | 4L | 5L | 9L | 12L | 13L | 18L | 24L | 27L | 29L | 32L | 33L | 34L | 37L | 38L | 39L | 41L | 42L | 45L | 46L | 48L | 51L | 52L | 53L | 57L | 60L | 65L | 70L | 72L | 74L |
|  |  |  |  |  |  |  |  |  |  |  |  |  |  |  |  |  |  |  |  |  |  |  |  |  |  |  |  |  |  |  |
| **41** | 21H | 37H | 50H | 51H | 56H | 57H | 60H | 68H | 70H | 71H | 76H | 82H | 84H | 87H | 93H | 96H | 109H | 111H | 115H | 119H | 149H | 157H | 158H | 165H | 184H | 186H | 191H | 197H | 203H | 209H |
|  | 1L | 4L | 5L | 9L | 12L | 13L | 18L | 24L | 27L | 29L | 32L | 33L | 34L | 37L | 38L | 39L | 41L | 42L | 45L | 46L | 48L | 51L | 52L | 53L | 57L | 60L | 65L | 70L | 72L | 74L |
|  |  |  |  |  |  |  |  |  |  |  |  |  |  |  |  |  |  |  |  |  |  |  |  |  |  |  |  |  |  |  |
| **42** | 28H | 50H | 61H | 69H | 76H | 80H | 87H | 96H | 109H | 110H | 116H | 127H | 131H | 133H | 134H | 138H | 139H | 141H | 142H | 144H | 152H | 166H | 172H | 174H | 176H | 177H | 188H | 202H | 207H | 209H |
|  | 1L | 4L | 5L | 9L | 12L | 13L | 18L | 24L | 27L | 29L | 32L | 33L | 34L | 37L | 38L | 39L | 41L | 42L | 45L | 46L | 48L | 51L | 52L | 53L | 57L | 60L | 65L | 70L | 72L | 74L |
|  |  |  |  |  |  |  |  |  |  |  |  |  |  |  |  |  |  |  |  |  |  |  |  |  |  |  |  |  |  |  |
| **43** | 22H | 23H | 33H | 34H | 40H | 60H | 79H | 83H | 86H | 93H | 103H | 108H | 114H | 115H | 119H | 127H | 137H | 149H | 155H | 160H | 165H | 170H | 171H | 172H | 184H | 190H | 195H | 197H | 208H | 209H |
|  | 1L | 4L | 5L | 9L | 12L | 13L | 18L | 24L | 27L | 29L | 32L | 33L | 34L | 37L | 38L | 39L | 41L | 42L | 45L | 46L | 48L | 51L | 52L | 53L | 57L | 60L | 65L | 70L | 72L | 74L |
|  |  |  |  |  |  |  |  |  |  |  |  |  |  |  |  |  |  |  |  |  |  |  |  |  |  |  |  |  |  |  |
| **44** | 4H | 24H | 26H | 40H | 44H | 52H | 60H | 61H | 67H | 68H | 70H | 78H | 81H | 91H | 92H | 95H | 103H | 104H | 113H | 118H | 122H | 124H | 128H | 135H | 153H | 157H | 162H | 184H | 189H | 201H |
|  | 1L | 4L | 5L | 9L | 12L | 13L | 18L | 24L | 27L | 29L | 32L | 33L | 34L | 37L | 38L | 39L | 41L | 42L | 45L | 46L | 48L | 51L | 52L | 53L | 57L | 60L | 65L | 70L | 72L | 74L |
|  |  |  |  |  |  |  |  |  |  |  |  |  |  |  |  |  |  |  |  |  |  |  |  |  |  |  |  |  |  |  |
| **45** | 22H | 23H | 24H | 27H | 29H | 32H | 38H | 81H | 83H | 87H | 93H | 111H | 114H | 115H | 119H | 123H | 124H | 127H | 130H | 133H | 135H | 136H | 150H | 155H | 162H | 170H | 171H | 175H | 208H | 210H |
|  | 1L | 4L | 5L | 9L | 12L | 13L | 18L | 24L | 27L | 29L | 32L | 33L | 34L | 37L | 38L | 39L | 41L | 42L | 45L | 46L | 48L | 51L | 52L | 53L | 57L | 60L | 65L | 70L | 72L | 74L |
|  |  |  |  |  |  |  |  |  |  |  |  |  |  |  |  |  |  |  |  |  |  |  |  |  |  |  |  |  |  |  |
| **46** | 26H | 37H | 38H | 41H | 51H | 54H | 56H | 60H | 73H | 75H | 82H | 84H | 109H | 116H | 128H | 129H | 131H | 139H | 146H | 156H | 157H | 158H | 160H | 176H | 190H | 196H | 200H | 201H | 203H | 210H |
|  | 1L | 4L | 5L | 9L | 12L | 13L | 18L | 24L | 27L | 29L | 32L | 33L | 34L | 37L | 38L | 39L | 41L | 42L | 45L | 46L | 48L | 51L | 52L | 53L | 57L | 60L | 65L | 70L | 72L | 74L |
|  |  |  |  |  |  |  |  |  |  |  |  |  |  |  |  |  |  |  |  |  |  |  |  |  |  |  |  |  |  |  |
| **47** | 8H | 9H | 12H | 26H | 32H | 34H | 40H | 42H | 56H | 57H | 66H | 74H | 81H | 86H | 88H | 101H | 105H | 118H | 122H | 125H | 126H | 143H | 156H | 162H | 165H | 176H | 177H | 178H | 190H | 203H |
|  | 1L | 4L | 5L | 9L | 12L | 13L | 18L | 24L | 27L | 29L | 32L | 33L | 34L | 37L | 38L | 39L | 41L | 42L | 45L | 46L | 48L | 51L | 52L | 53L | 57L | 60L | 65L | 70L | 72L | 74L |
|  |  |  |  |  |  |  |  |  |  |  |  |  |  |  |  |  |  |  |  |  |  |  |  |  |  |  |  |  |  |  |
| **48** | 9H | 12H | 23H | 32H | 37H | 44H | 51H | 54H | 62H | 64H | 69H | 83H | 84H | 86H | 95H | 123H | 136H | 137H | 143H | 152H | 157H | 160H | 167H | 175H | 176H | 177H | 178H | 189H | 190H | 195H |
|  | 1L | 4L | 5L | 9L | 12L | 13L | 18L | 24L | 27L | 29L | 32L | 33L | 34L | 37L | 38L | 39L | 41L | 42L | 45L | 46L | 48L | 51L | 52L | 53L | 57L | 60L | 65L | 70L | 72L | 74L |
|  |  |  |  |  |  |  |  |  |  |  |  |  |  |  |  |  |  |  |  |  |  |  |  |  |  |  |  |  |  |  |
| **49** | 8H | 9H | 33H | 36H | 64H | 68H | 78H | 81H | 86H | 88H | 112H | 119H | 125H | 130H | 131H | 133H | 134H | 141H | 143H | 146H | 160H | 168H | 172H | 184H | 186H | 188H | 191H | 194H | 199H | 202H |
|  | 1L | 4L | 5L | 9L | 12L | 13L | 18L | 24L | 27L | 29L | 32L | 33L | 34L | 37L | 38L | 39L | 41L | 42L | 45L | 46L | 48L | 51L | 52L | 53L | 57L | 60L | 65L | 70L | 72L | 74L |
|  |  |  |  |  |  |  |  |  |  |  |  |  |  |  |  |  |  |  |  |  |  |  |  |  |  |  |  |  |  |  |
| **50** | 7H | 14H | 24H | 32H | 33H | 36H | 40H | 52H | 57H | 64H | 69H | 75H | 82H | 95H | 103H | 105H | 112H | 126H | 127H | 128H | 135H | 141H | 163H | 167H | 171H | 175H | 188H | 191H | 201H | 207H |
|  | 1L | 4L | 5L | 9L | 12L | 13L | 18L | 24L | 27L | 29L | 32L | 33L | 34L | 37L | 38L | 39L | 41L | 42L | 45L | 46L | 48L | 51L | 52L | 53L | 57L | 60L | 65L | 70L | 72L | 74L |
|  |  |  |  |  |  |  |  |  |  |  |  |  |  |  |  |  |  |  |  |  |  |  |  |  |  |  |  |  |  |  |
| **51** | 8H | 9H | 13H | 14H | 23H | 24H | 26H | 35H | 51H | 56H | 68H | 78H | 81H | 111H | 118H | 119H | 127H | 133H | 136H | 152H | 154H | 162H | 166H | 171H | 174H | 177H | 189H | 190H | 196H | 199H |
|  | 1L | 4L | 5L | 9L | 12L | 13L | 18L | 24L | 27L | 29L | 32L | 33L | 34L | 37L | 38L | 39L | 41L | 42L | 45L | 46L | 48L | 51L | 52L | 53L | 57L | 60L | 65L | 70L | 72L | 74L |
|  |  |  |  |  |  |  |  |  |  |  |  |  |  |  |  |  |  |  |  |  |  |  |  |  |  |  |  |  |  |  |
| **52** | 7H | 9H | 19H | 22H | 34H | 37H | 51H | 71H | 74H | 98H | 108H | 109H | 111H | 118H | 128H | 135H | 141H | 142H | 148H | 149H | 152H | 160H | 163H | 168H | 194H | 197H | 199H | 201H | 203H | 209H |
|  | 1L | 4L | 5L | 9L | 12L | 13L | 18L | 24L | 27L | 29L | 32L | 33L | 34L | 37L | 38L | 39L | 41L | 42L | 45L | 46L | 48L | 51L | 52L | 53L | 57L | 60L | 65L | 70L | 72L | 74L |
|  |  |  |  |  |  |  |  |  |  |  |  |  |  |  |  |  |  |  |  |  |  |  |  |  |  |  |  |  |  |  |
| **53** | 7H | 12H | 21H | 26H | 33H | 41H | 54H | 57H | 60H | 61H | 74H | 81H | 95H | 116H | 119H | 123H | 128H | 131H | 135H | 144H | 146H | 149H | 152H | 167H | 171H | 175H | 189H | 194H | 195H | 201H |
|  | 1L | 4L | 5L | 9L | 12L | 13L | 18L | 24L | 27L | 29L | 32L | 33L | 34L | 37L | 38L | 39L | 41L | 42L | 45L | 46L | 48L | 51L | 52L | 53L | 57L | 60L | 65L | 70L | 72L | 74L |
|  |  |  |  |  |  |  |  |  |  |  |  |  |  |  |  |  |  |  |  |  |  |  |  |  |  |  |  |  |  |  |
| **54** | 8H | 21H | 27H | 28H | 38H | 50H | 51H | 61H | 64H | 73H | 76H | 79H | 83H | 84H | 98H | 102H | 104H | 105H | 114H | 122H | 125H | 146H | 156H | 160H | 189H | 198H | 201H | 202H | 207H | 208H |
|  | 1L | 4L | 5L | 9L | 12L | 13L | 18L | 24L | 27L | 29L | 32L | 33L | 34L | 37L | 38L | 39L | 41L | 42L | 45L | 46L | 48L | 51L | 52L | 53L | 57L | 60L | 65L | 70L | 72L | 74L |
|  |  |  |  |  |  |  |  |  |  |  |  |  |  |  |  |  |  |  |  |  |  |  |  |  |  |  |  |  |  |  |
| **55** | 14H | 16H | 23H | 26H | 28H | 45H | 56H | 57H | 66H | 71H | 75H | 80H | 83H | 92H | 111H | 114H | 119H | 130H | 133H | 144H | 147H | 149H | 154H | 156H | 170H | 171H | 172H | 175H | 184H | 193H |
|  | 1L | 4L | 5L | 9L | 12L | 13L | 18L | 24L | 27L | 29L | 32L | 33L | 34L | 37L | 38L | 39L | 41L | 42L | 45L | 46L | 48L | 51L | 52L | 53L | 57L | 60L | 65L | 70L | 72L | 74L |
|  |  |  |  |  |  |  |  |  |  |  |  |  |  |  |  |  |  |  |  |  |  |  |  |  |  |  |  |  |  |  |
| **56** | 14H | 16H | 28H | 36H | 45H | 57H | 60H | 64H | 66H | 74H | 81H | 84H | 87H | 91H | 101H | 104H | 105H | 127H | 130H | 137H | 146H | 151H | 157H | 169H | 174H | 175H | 186H | 189H | 194H | 195H |
|  | 1L | 4L | 5L | 9L | 12L | 13L | 18L | 24L | 27L | 29L | 32L | 33L | 34L | 37L | 38L | 39L | 41L | 42L | 45L | 46L | 48L | 51L | 52L | 53L | 57L | 60L | 65L | 70L | 72L | 74L |
|  |  |  |  |  |  |  |  |  |  |  |  |  |  |  |  |  |  |  |  |  |  |  |  |  |  |  |  |  |  |  |
| **57** | 2H | 8H | 23H | 26H | 32H | 33H | 38H | 42H | 45H | 50H | 57H | 61H | 66H | 78H | 87H | 110H | 122H | 127H | 129H | 133H | 148H | 150H | 153H | 172H | 174H | 184H | 188H | 198H | 201H | 208H |
|  | 1L | 4L | 5L | 9L | 12L | 13L | 18L | 24L | 27L | 29L | 32L | 33L | 34L | 37L | 38L | 39L | 41L | 42L | 45L | 46L | 48L | 51L | 52L | 53L | 57L | 60L | 65L | 70L | 72L | 74L |
|  |  |  |  |  |  |  |  |  |  |  |  |  |  |  |  |  |  |  |  |  |  |  |  |  |  |  |  |  |  |  |
| **58** | 16H | 36H | 40H | 44H | 50H | 62H | 64H | 83H | 98H | 102H | 109H | 111H | 114H | 118H | 119H | 125H | 131H | 142H | 144H | 150H | 158H | 160H | 164H | 172H | 178H | 197H | 199H | 203H | 206H | 209H |
|  | 1L | 4L | 5L | 9L | 12L | 13L | 18L | 24L | 27L | 29L | 32L | 33L | 34L | 37L | 38L | 39L | 41L | 42L | 45L | 46L | 48L | 51L | 52L | 53L | 57L | 60L | 65L | 70L | 72L | 74L |
|  |  |  |  |  |  |  |  |  |  |  |  |  |  |  |  |  |  |  |  |  |  |  |  |  |  |  |  |  |  |  |
| **59** | 21H | 26H | 32H | 33H | 44H | 51H | 60H | 64H | 87H | 95H | 108H | 112H | 114H | 115H | 116H | 118H | 124H | 125H | 126H | 130H | 136H | 142H | 146H | 155H | 165H | 176H | 186H | 188H | 189H | 196H |
|  | 1L | 4L | 5L | 9L | 12L | 13L | 18L | 24L | 27L | 29L | 32L | 33L | 34L | 37L | 38L | 39L | 41L | 42L | 45L | 46L | 48L | 51L | 52L | 53L | 57L | 60L | 65L | 70L | 72L | 74L |
|  |  |  |  |  |  |  |  |  |  |  |  |  |  |  |  |  |  |  |  |  |  |  |  |  |  |  |  |  |  |  |
| **60** | 7H | 26H | 36H | 51H | 68H | 71H | 95H | 98H | 102H | 103H | 104H | 109H | 111H | 116H | 122H | 125H | 134H | 136H | 138H | 153H | 155H | 160H | 164H | 165H | 174H | 176H | 197H | 202H | 203H | 210H |
|  | 1L | 4L | 5L | 9L | 12L | 13L | 18L | 24L | 27L | 29L | 32L | 33L | 34L | 37L | 38L | 39L | 41L | 42L | 45L | 46L | 48L | 51L | 52L | 53L | 57L | 60L | 65L | 70L | 72L | 74L |
|  |  |  |  |  |  |  |  |  |  |  |  |  |  |  |  |  |  |  |  |  |  |  |  |  |  |  |  |  |  |  |
| **61** | 13H | 23H | 27H | 28H | 34H | 36H | 38H | 41H | 42H | 61H | 66H | 74H | 76H | 96H | 109H | 112H | 113H | 116H | 143H | 147H | 149H | 153H | 156H | 157H | 171H | 172H | 189H | 194H | 201H | 210H |
|  | 1L | 4L | 5L | 9L | 12L | 13L | 18L | 24L | 27L | 29L | 32L | 33L | 34L | 37L | 38L | 39L | 41L | 42L | 45L | 46L | 48L | 51L | 52L | 53L | 57L | 60L | 65L | 70L | 72L | 74L |
|  |  |  |  |  |  |  |  |  |  |  |  |  |  |  |  |  |  |  |  |  |  |  |  |  |  |  |  |  |  |  |
| **62** | 7H | 8H | 12H | 16H | 19H | 32H | 41H | 44H | 52H | 60H | 64H | 66H | 69H | 91H | 93H | 108H | 114H | 125H | 128H | 130H | 134H | 146H | 147H | 150H | 153H | 166H | 167H | 184H | 193H | 207H |
|  | 1L | 4L | 5L | 9L | 12L | 13L | 18L | 24L | 27L | 29L | 32L | 33L | 34L | 37L | 38L | 39L | 41L | 42L | 45L | 46L | 48L | 51L | 52L | 53L | 57L | 60L | 65L | 70L | 72L | 74L |
|  |  |  |  |  |  |  |  |  |  |  |  |  |  |  |  |  |  |  |  |  |  |  |  |  |  |  |  |  |  |  |
| **63** | 2H | 4H | 16H | 29H | 60H | 61H | 74H | 88H | 91H | 102H | 103H | 108H | 124H | 126H | 129H | 135H | 136H | 144H | 148H | 160H | 162H | 165H | 177H | 178H | 188H | 190H | 194H | 198H | 199H | 208H |
|  | 1L | 4L | 5L | 9L | 12L | 13L | 18L | 24L | 27L | 29L | 32L | 33L | 34L | 37L | 38L | 39L | 41L | 42L | 45L | 46L | 48L | 51L | 52L | 53L | 57L | 60L | 65L | 70L | 72L | 74L |
|  |  |  |  |  |  |  |  |  |  |  |  |  |  |  |  |  |  |  |  |  |  |  |  |  |  |  |  |  |  |  |
| **64** | 7H | 29H | 37H | 38H | 45H | 52H | 67H | 69H | 73H | 104H | 109H | 112H | 114H | 116H | 122H | 127H | 132H | 137H | 148H | 149H | 150H | 154H | 157H | 160H | 162H | 171H | 176H | 193H | 201H | 206H |
|  | 1L | 4L | 5L | 9L | 12L | 13L | 18L | 24L | 27L | 29L | 32L | 33L | 34L | 37L | 38L | 39L | 41L | 42L | 45L | 46L | 48L | 51L | 52L | 53L | 57L | 60L | 65L | 70L | 72L | 74L |
|  |  |  |  |  |  |  |  |  |  |  |  |  |  |  |  |  |  |  |  |  |  |  |  |  |  |  |  |  |  |  |
| **65** | 4H | 7H | 13H | 19H | 21H | 37H | 51H | 53H | 68H | 69H | 80H | 95H | 101H | 108H | 112H | 116H | 122H | 128H | 131H | 133H | 138H | 139H | 149H | 152H | 172H | 174H | 190H | 191H | 198H | 202H |
|  | 1L | 4L | 5L | 9L | 12L | 13L | 18L | 24L | 27L | 29L | 32L | 33L | 34L | 37L | 38L | 39L | 41L | 42L | 45L | 46L | 48L | 51L | 52L | 53L | 57L | 60L | 65L | 70L | 72L | 74L |
|  |  |  |  |  |  |  |  |  |  |  |  |  |  |  |  |  |  |  |  |  |  |  |  |  |  |  |  |  |  |  |
| **66** | 13H | 26H | 32H | 33H | 37H | 40H | 44H | 57H | 62H | 66H | 67H | 71H | 76H | 79H | 80H | 84H | 102H | 108H | 122H | 129H | 147H | 148H | 156H | 157H | 158H | 160H | 168H | 170H | 190H | 198H |
|  | 1L | 4L | 5L | 9L | 12L | 13L | 18L | 24L | 27L | 29L | 32L | 33L | 34L | 37L | 38L | 39L | 41L | 42L | 45L | 46L | 48L | 51L | 52L | 53L | 57L | 60L | 65L | 70L | 72L | 74L |
|  |  |  |  |  |  |  |  |  |  |  |  |  |  |  |  |  |  |  |  |  |  |  |  |  |  |  |  |  |  |  |
| **67** | 8H | 19H | 34H | 38H | 42H | 54H | 74H | 83H | 87H | 95H | 102H | 105H | 111H | 112H | 119H | 125H | 132H | 134H | 136H | 148H | 152H | 164H | 167H | 170H | 186H | 188H | 193H | 194H | 201H | 208H |
|  | 1L | 4L | 5L | 9L | 12L | 13L | 18L | 24L | 27L | 29L | 32L | 33L | 34L | 37L | 38L | 39L | 41L | 42L | 45L | 46L | 48L | 51L | 52L | 53L | 57L | 60L | 65L | 70L | 72L | 74L |
|  |  |  |  |  |  |  |  |  |  |  |  |  |  |  |  |  |  |  |  |  |  |  |  |  |  |  |  |  |  |  |
| **68** | 12H | 14H | 19H | 21H | 24H | 34H | 40H | 45H | 70H | 74H | 80H | 82H | 84H | 104H | 122H | 125H | 126H | 130H | 131H | 137H | 138H | 143H | 148H | 156H | 158H | 184H | 189H | 193H | 197H | 207H |
|  | 1L | 4L | 5L | 9L | 12L | 13L | 18L | 24L | 27L | 29L | 32L | 33L | 34L | 37L | 38L | 39L | 41L | 42L | 45L | 46L | 48L | 51L | 52L | 53L | 57L | 60L | 65L | 70L | 72L | 74L |
|  |  |  |  |  |  |  |  |  |  |  |  |  |  |  |  |  |  |  |  |  |  |  |  |  |  |  |  |  |  |  |
| **69** | 8H | 12H | 16H | 22H | 23H | 44H | 56H | 57H | 67H | 68H | 86H | 93H | 102H | 104H | 108H | 113H | 116H | 119H | 126H | 133H | 150H | 151H | 153H | 157H | 177H | 178H | 195H | 196H | 202H | 209H |
|  | 1L | 4L | 5L | 9L | 12L | 13L | 18L | 24L | 27L | 29L | 32L | 33L | 34L | 37L | 38L | 39L | 41L | 42L | 45L | 46L | 48L | 51L | 52L | 53L | 57L | 60L | 65L | 70L | 72L | 74L |
|  |  |  |  |  |  |  |  |  |  |  |  |  |  |  |  |  |  |  |  |  |  |  |  |  |  |  |  |  |  |  |
| **70** | 19H | 32H | 52H | 62H | 67H | 70H | 74H | 76H | 78H | 82H | 84H | 87H | 101H | 104H | 105H | 115H | 116H | 127H | 133H | 135H | 139H | 146H | 147H | 148H | 167H | 170H | 176H | 195H | 199H | 202H |
|  | 1L | 4L | 5L | 9L | 12L | 13L | 18L | 24L | 27L | 29L | 32L | 33L | 34L | 37L | 38L | 39L | 41L | 42L | 45L | 46L | 48L | 51L | 52L | 53L | 57L | 60L | 65L | 70L | 72L | 74L |
|  |  |  |  |  |  |  |  |  |  |  |  |  |  |  |  |  |  |  |  |  |  |  |  |  |  |  |  |  |  |  |
| **71** | 2H | 7H | 12H | 13H | 36H | 38H | 41H | 42H | 45H | 50H | 56H | 57H | 60H | 68H | 71H | 73H | 81H | 83H | 113H | 114H | 122H | 142H | 149H | 152H | 158H | 190H | 191H | 193H | 197H | 210H |
|  | 1L | 4L | 5L | 9L | 12L | 13L | 18L | 24L | 27L | 29L | 32L | 33L | 34L | 37L | 38L | 39L | 41L | 42L | 45L | 46L | 48L | 51L | 52L | 53L | 57L | 60L | 65L | 70L | 72L | 74L |
|  |  |  |  |  |  |  |  |  |  |  |  |  |  |  |  |  |  |  |  |  |  |  |  |  |  |  |  |  |  |  |
| **72** | 7H | 22H | 29H | 36H | 51H | 57H | 61H | 66H | 79H | 84H | 86H | 96H | 114H | 115H | 118H | 124H | 126H | 127H | 131H | 143H | 152H | 157H | 160H | 172H | 175H | 177H | 186H | 194H | 198H | 206H |
|  | 1L | 4L | 5L | 9L | 12L | 13L | 18L | 24L | 27L | 29L | 32L | 33L | 34L | 37L | 38L | 39L | 41L | 42L | 45L | 46L | 48L | 51L | 52L | 53L | 57L | 60L | 65L | 70L | 72L | 74L |
|  |  |  |  |  |  |  |  |  |  |  |  |  |  |  |  |  |  |  |  |  |  |  |  |  |  |  |  |  |  |  |
| **73** | 2H | 7H | 22H | 35H | 36H | 45H | 67H | 73H | 75H | 82H | 83H | 91H | 96H | 102H | 104H | 108H | 109H | 111H | 112H | 124H | 127H | 141H | 144H | 148H | 153H | 158H | 164H | 170H | 184H | 196H |
|  | 1L | 4L | 5L | 9L | 12L | 13L | 18L | 24L | 27L | 29L | 32L | 33L | 34L | 37L | 38L | 39L | 41L | 42L | 45L | 46L | 48L | 51L | 52L | 53L | 57L | 60L | 65L | 70L | 72L | 74L |
|  |  |  |  |  |  |  |  |  |  |  |  |  |  |  |  |  |  |  |  |  |  |  |  |  |  |  |  |  |  |  |
| **74** | 14H | 16H | 34H | 36H | 40H | 44H | 74H | 75H | 78H | 86H | 92H | 95H | 102H | 109H | 119H | 122H | 126H | 128H | 132H | 138H | 151H | 154H | 165H | 169H | 175H | 184H | 193H | 198H | 202H | 203H |
|  | 1L | 4L | 5L | 9L | 12L | 13L | 18L | 24L | 27L | 29L | 32L | 33L | 34L | 37L | 38L | 39L | 41L | 42L | 45L | 46L | 48L | 51L | 52L | 53L | 57L | 60L | 65L | 70L | 72L | 74L |
|  |  |  |  |  |  |  |  |  |  |  |  |  |  |  |  |  |  |  |  |  |  |  |  |  |  |  |  |  |  |  |
| **75** | 2H | 9H | 19H | 22H | 29H | 34H | 36H | 37H | 42H | 67H | 82H | 84H | 95H | 98H | 110H | 113H | 116H | 130H | 131H | 139H | 143H | 146H | 148H | 154H | 155H | 177H | 189H | 194H | 200H | 210H |
|  | 1L | 4L | 5L | 9L | 12L | 13L | 18L | 24L | 27L | 29L | 32L | 33L | 34L | 37L | 38L | 39L | 41L | 42L | 45L | 46L | 48L | 51L | 52L | 53L | 57L | 60L | 65L | 70L | 72L | 74L |
|  |  |  |  |  |  |  |  |  |  |  |  |  |  |  |  |  |  |  |  |  |  |  |  |  |  |  |  |  |  |  |
| **76** | 24H | 28H | 36H | 54H | 57H | 60H | 62H | 67H | 79H | 82H | 83H | 91H | 92H | 112H | 116H | 119H | 126H | 128H | 129H | 132H | 154H | 158H | 162H | 168H | 188H | 193H | 197H | 203H | 208H | 209H |
|  | 1L | 4L | 5L | 9L | 12L | 13L | 18L | 24L | 27L | 29L | 32L | 33L | 34L | 37L | 38L | 39L | 41L | 42L | 45L | 46L | 48L | 51L | 52L | 53L | 57L | 60L | 65L | 70L | 72L | 74L |
|  |  |  |  |  |  |  |  |  |  |  |  |  |  |  |  |  |  |  |  |  |  |  |  |  |  |  |  |  |  |  |
| **77** | 8H | 32H | 37H | 60H | 62H | 68H | 70H | 73H | 98H | 104H | 109H | 122H | 126H | 142H | 144H | 156H | 160H | 163H | 168H | 169H | 170H | 172H | 178H | 184H | 186H | 189H | 191H | 195H | 197H | 207H |
|  | 1L | 4L | 5L | 9L | 12L | 13L | 18L | 24L | 27L | 29L | 32L | 33L | 34L | 37L | 38L | 39L | 41L | 42L | 45L | 46L | 48L | 51L | 52L | 53L | 57L | 60L | 65L | 70L | 72L | 74L |
|  |  |  |  |  |  |  |  |  |  |  |  |  |  |  |  |  |  |  |  |  |  |  |  |  |  |  |  |  |  |  |
| **78** | 8H | 16H | 24H | 26H | 28H | 33H | 38H | 44H | 45H | 71H | 74H | 75H | 84H | 87H | 93H | 98H | 111H | 112H | 116H | 123H | 130H | 135H | 139H | 148H | 154H | 155H | 157H | 160H | 164H | 189H |
|  | 1L | 4L | 5L | 9L | 12L | 13L | 18L | 24L | 27L | 29L | 32L | 33L | 34L | 37L | 38L | 39L | 41L | 42L | 45L | 46L | 48L | 51L | 52L | 53L | 57L | 60L | 65L | 70L | 72L | 74L |
|  |  |  |  |  |  |  |  |  |  |  |  |  |  |  |  |  |  |  |  |  |  |  |  |  |  |  |  |  |  |  |
| **79** | 9H | 21H | 32H | 34H | 36H | 38H | 41H | 42H | 66H | 70H | 76H | 80H | 91H | 103H | 104H | 105H | 111H | 131H | 137H | 144H | 150H | 151H | 152H | 162H | 164H | 168H | 170H | 186H | 200H | 206H |
|  | 1L | 4L | 5L | 9L | 12L | 13L | 18L | 24L | 27L | 29L | 32L | 33L | 34L | 37L | 38L | 39L | 41L | 42L | 45L | 46L | 48L | 51L | 52L | 53L | 57L | 60L | 65L | 70L | 72L | 74L |
|  |  |  |  |  |  |  |  |  |  |  |  |  |  |  |  |  |  |  |  |  |  |  |  |  |  |  |  |  |  |  |
| **80** | 9H | 13H | 22H | 23H | 37H | 53H | 67H | 76H | 80H | 87H | 88H | 104H | 109H | 122H | 133H | 142H | 147H | 148H | 149H | 155H | 164H | 165H | 167H | 176H | 191H | 193H | 194H | 196H | 201H | 209H |
|  | 1L | 4L | 5L | 9L | 12L | 13L | 18L | 24L | 27L | 29L | 32L | 33L | 34L | 37L | 38L | 39L | 41L | 42L | 45L | 46L | 48L | 51L | 52L | 53L | 57L | 60L | 65L | 70L | 72L | 74L |
|  |  |  |  |  |  |  |  |  |  |  |  |  |  |  |  |  |  |  |  |  |  |  |  |  |  |  |  |  |  |  |
| **81** | 4H | 16H | 19H | 32H | 44H | 51H | 53H | 60H | 61H | 75H | 76H | 82H | 96H | 119H | 122H | 130H | 131H | 133H | 137H | 147H | 149H | 152H | 153H | 164H | 168H | 172H | 188H | 190H | 195H | 209H |
|  | 1L | 4L | 5L | 9L | 12L | 13L | 18L | 24L | 27L | 29L | 32L | 33L | 34L | 37L | 38L | 39L | 41L | 42L | 45L | 46L | 48L | 51L | 52L | 53L | 57L | 60L | 65L | 70L | 72L | 74L |
|  |  |  |  |  |  |  |  |  |  |  |  |  |  |  |  |  |  |  |  |  |  |  |  |  |  |  |  |  |  |  |
| **82** | 9H | 16H | 19H | 24H | 33H | 36H | 37H | 42H | 66H | 71H | 76H | 83H | 91H | 95H | 98H | 110H | 115H | 147H | 154H | 155H | 163H | 164H | 170H | 184H | 188H | 194H | 200H | 206H | 207H | 209H |
|  | 1L | 4L | 5L | 9L | 12L | 13L | 18L | 24L | 27L | 29L | 32L | 33L | 34L | 37L | 38L | 39L | 41L | 42L | 45L | 46L | 48L | 51L | 52L | 53L | 57L | 60L | 65L | 70L | 72L | 74L |
|  |  |  |  |  |  |  |  |  |  |  |  |  |  |  |  |  |  |  |  |  |  |  |  |  |  |  |  |  |  |  |
| **83** | 2H | 9H | 12H | 22H | 24H | 34H | 52H | 57H | 78H | 81H | 82H | 87H | 93H | 101H | 103H | 108H | 113H | 126H | 137H | 142H | 151H | 153H | 157H | 158H | 164H | 174H | 176H | 199H | 207H | 209H |
|  | 1L | 4L | 5L | 9L | 12L | 13L | 18L | 24L | 27L | 29L | 32L | 33L | 34L | 37L | 38L | 39L | 41L | 42L | 45L | 46L | 48L | 51L | 52L | 53L | 57L | 60L | 65L | 70L | 72L | 74L |
|  |  |  |  |  |  |  |  |  |  |  |  |  |  |  |  |  |  |  |  |  |  |  |  |  |  |  |  |  |  |  |
| **84** | 2H | 9H | 12H | 19H | 27H | 36H | 38H | 60H | 69H | 71H | 75H | 78H | 79H | 84H | 92H | 95H | 111H | 124H | 128H | 129H | 131H | 133H | 143H | 146H | 148H | 149H | 155H | 174H | 190H | 196H |
|  | 1L | 4L | 5L | 9L | 12L | 13L | 18L | 24L | 27L | 29L | 32L | 33L | 34L | 37L | 38L | 39L | 41L | 42L | 45L | 46L | 48L | 51L | 52L | 53L | 57L | 60L | 65L | 70L | 72L | 74L |
|  |  |  |  |  |  |  |  |  |  |  |  |  |  |  |  |  |  |  |  |  |  |  |  |  |  |  |  |  |  |  |
| **85** | 2H | 4H | 8H | 9H | 16H | 19H | 27H | 32H | 50H | 51H | 61H | 68H | 69H | 76H | 86H | 112H | 128H | 132H | 142H | 146H | 149H | 160H | 167H | 168H | 175H | 184H | 188H | 189H | 194H | 203H |
|  | 1L | 4L | 5L | 9L | 12L | 13L | 18L | 24L | 27L | 29L | 32L | 33L | 34L | 37L | 38L | 39L | 41L | 42L | 45L | 46L | 48L | 51L | 52L | 53L | 57L | 60L | 65L | 70L | 72L | 74L |
|  |  |  |  |  |  |  |  |  |  |  |  |  |  |  |  |  |  |  |  |  |  |  |  |  |  |  |  |  |  |  |
| **86** | 26H | 40H | 53H | 62H | 64H | 66H | 68H | 71H | 87H | 91H | 96H | 112H | 119H | 123H | 124H | 137H | 138H | 139H | 143H | 153H | 157H | 162H | 163H | 176H | 177H | 178H | 186H | 198H | 209H | 210H |
|  | 1L | 4L | 5L | 9L | 12L | 13L | 18L | 24L | 27L | 29L | 32L | 33L | 34L | 37L | 38L | 39L | 41L | 42L | 45L | 46L | 48L | 51L | 52L | 53L | 57L | 60L | 65L | 70L | 72L | 74L |
|  |  |  |  |  |  |  |  |  |  |  |  |  |  |  |  |  |  |  |  |  |  |  |  |  |  |  |  |  |  |  |
| **87** | 16H | 19H | 28H | 33H | 35H | 38H | 61H | 69H | 76H | 81H | 82H | 84H | 92H | 95H | 123H | 133H | 137H | 142H | 143H | 152H | 153H | 158H | 160H | 163H | 175H | 177H | 188H | 193H | 198H | 210H |
|  | 1L | 4L | 5L | 9L | 12L | 13L | 18L | 24L | 27L | 29L | 32L | 33L | 34L | 37L | 38L | 39L | 41L | 42L | 45L | 46L | 48L | 51L | 52L | 53L | 57L | 60L | 65L | 70L | 72L | 74L |
|  |  |  |  |  |  |  |  |  |  |  |  |  |  |  |  |  |  |  |  |  |  |  |  |  |  |  |  |  |  |  |
| **88** | 9H | 12H | 13H | 14H | 22H | 38H | 40H | 41H | 57H | 88H | 104H | 111H | 115H | 118H | 122H | 128H | 132H | 134H | 136H | 148H | 149H | 150H | 153H | 157H | 164H | 194H | 202H | 203H | 206H | 210H |
|  | 1L | 4L | 5L | 9L | 12L | 13L | 18L | 24L | 27L | 29L | 32L | 33L | 34L | 37L | 38L | 39L | 41L | 42L | 45L | 46L | 48L | 51L | 52L | 53L | 57L | 60L | 65L | 70L | 72L | 74L |
|  |  |  |  |  |  |  |  |  |  |  |  |  |  |  |  |  |  |  |  |  |  |  |  |  |  |  |  |  |  |  |
| **89** | 2H | 14H | 21H | 22H | 23H | 41H | 69H | 71H | 83H | 86H | 88H | 101H | 104H | 111H | 112H | 118H | 129H | 130H | 131H | 141H | 142H | 143H | 152H | 158H | 160H | 164H | 165H | 168H | 176H | 202H |
|  | 1L | 4L | 5L | 9L | 12L | 13L | 18L | 24L | 27L | 29L | 32L | 33L | 34L | 37L | 38L | 39L | 41L | 42L | 45L | 46L | 48L | 51L | 52L | 53L | 57L | 60L | 65L | 70L | 72L | 74L |
|  |  |  |  |  |  |  |  |  |  |  |  |  |  |  |  |  |  |  |  |  |  |  |  |  |  |  |  |  |  |  |
| **90** | 16H | 35H | 44H | 51H | 60H | 61H | 67H | 70H | 75H | 76H | 82H | 91H | 112H | 116H | 118H | 124H | 129H | 139H | 144H | 149H | 163H | 167H | 168H | 171H | 175H | 176H | 178H | 184H | 193H | 202H |
|  | 1L | 4L | 5L | 9L | 12L | 13L | 18L | 24L | 27L | 29L | 32L | 33L | 34L | 37L | 38L | 39L | 41L | 42L | 45L | 46L | 48L | 51L | 52L | 53L | 57L | 60L | 65L | 70L | 72L | 74L |
|  |  |  |  |  |  |  |  |  |  |  |  |  |  |  |  |  |  |  |  |  |  |  |  |  |  |  |  |  |  |  |
| **91** | 7H | 21H | 32H | 36H | 37H | 40H | 42H | 44H | 45H | 56H | 60H | 64H | 67H | 87H | 88H | 98H | 110H | 112H | 113H | 114H | 118H | 122H | 126H | 130H | 141H | 154H | 166H | 189H | 202H | 203H |
|  | 1L | 4L | 5L | 9L | 12L | 13L | 18L | 24L | 27L | 29L | 32L | 33L | 34L | 37L | 38L | 39L | 41L | 42L | 45L | 46L | 48L | 51L | 52L | 53L | 57L | 60L | 65L | 70L | 72L | 74L |
|  |  |  |  |  |  |  |  |  |  |  |  |  |  |  |  |  |  |  |  |  |  |  |  |  |  |  |  |  |  |  |
| **92** | 2H | 14H | 19H | 22H | 32H | 53H | 67H | 86H | 88H | 110H | 113H | 118H | 122H | 125H | 126H | 136H | 137H | 141H | 148H | 150H | 151H | 154H | 158H | 163H | 189H | 202H | 206H | 208H | 209H | 210H |
|  | 1L | 4L | 5L | 9L | 12L | 13L | 18L | 24L | 27L | 29L | 32L | 33L | 34L | 37L | 38L | 39L | 41L | 42L | 45L | 46L | 48L | 51L | 52L | 53L | 57L | 60L | 65L | 70L | 72L | 74L |
|  |  |  |  |  |  |  |  |  |  |  |  |  |  |  |  |  |  |  |  |  |  |  |  |  |  |  |  |  |  |  |
| **93** | 4H | 8H | 9H | 23H | 41H | 60H | 66H | 68H | 76H | 96H | 102H | 103H | 105H | 123H | 124H | 125H | 137H | 139H | 146H | 148H | 155H | 160H | 174H | 184H | 195H | 198H | 201H | 202H | 206H | 207H |
|  | 1L | 4L | 5L | 9L | 12L | 13L | 18L | 24L | 27L | 29L | 32L | 33L | 34L | 37L | 38L | 39L | 41L | 42L | 45L | 46L | 48L | 51L | 52L | 53L | 57L | 60L | 65L | 70L | 72L | 74L |
|  |  |  |  |  |  |  |  |  |  |  |  |  |  |  |  |  |  |  |  |  |  |  |  |  |  |  |  |  |  |  |
| **94** | 16H | 24H | 37H | 38H | 42H | 54H | 69H | 70H | 78H | 81H | 83H | 113H | 131H | 132H | 133H | 136H | 137H | 141H | 144H | 147H | 148H | 158H | 165H | 171H | 172H | 176H | 184H | 199H | 202H | 203H |
|  | 1L | 4L | 5L | 9L | 12L | 13L | 18L | 24L | 27L | 29L | 32L | 33L | 34L | 37L | 38L | 39L | 41L | 42L | 45L | 46L | 48L | 51L | 52L | 53L | 57L | 60L | 65L | 70L | 72L | 74L |
|  |  |  |  |  |  |  |  |  |  |  |  |  |  |  |  |  |  |  |  |  |  |  |  |  |  |  |  |  |  |  |
| **95** | 9H | 14H | 24H | 28H | 52H | 53H | 57H | 70H | 71H | 74H | 88H | 103H | 104H | 108H | 125H | 131H | 139H | 141H | 150H | 151H | 157H | 163H | 168H | 174H | 196H | 198H | 200H | 203H | 207H | 209H |
|  | 1L | 4L | 5L | 9L | 12L | 13L | 18L | 24L | 27L | 29L | 32L | 33L | 34L | 37L | 38L | 39L | 41L | 42L | 45L | 46L | 48L | 51L | 52L | 53L | 57L | 60L | 65L | 70L | 72L | 74L |
|  |  |  |  |  |  |  |  |  |  |  |  |  |  |  |  |  |  |  |  |  |  |  |  |  |  |  |  |  |  |  |
| **96** | 7H | 9H | 12H | 16H | 19H | 29H | 32H | 33H | 35H | 57H | 61H | 73H | 81H | 83H | 88H | 98H | 103H | 109H | 122H | 127H | 146H | 156H | 160H | 167H | 172H | 174H | 188H | 191H | 193H | 195H |
|  | 1L | 4L | 5L | 9L | 12L | 13L | 18L | 24L | 27L | 29L | 32L | 33L | 34L | 37L | 38L | 39L | 41L | 42L | 45L | 46L | 48L | 51L | 52L | 53L | 57L | 60L | 65L | 70L | 72L | 74L |
|  |  |  |  |  |  |  |  |  |  |  |  |  |  |  |  |  |  |  |  |  |  |  |  |  |  |  |  |  |  |  |
| **97** | 9H | 13H | 16H | 23H | 24H | 37H | 44H | 61H | 67H | 70H | 101H | 105H | 110H | 112H | 114H | 115H | 116H | 126H | 127H | 135H | 143H | 146H | 155H | 170H | 172H | 178H | 186H | 200H | 209H | 210H |
|  | 1L | 4L | 5L | 9L | 12L | 13L | 18L | 24L | 27L | 29L | 32L | 33L | 34L | 37L | 38L | 39L | 41L | 42L | 45L | 46L | 48L | 51L | 52L | 53L | 57L | 60L | 65L | 70L | 72L | 74L |
|  |  |  |  |  |  |  |  |  |  |  |  |  |  |  |  |  |  |  |  |  |  |  |  |  |  |  |  |  |  |  |
| **98** | 9H | 19H | 22H | 32H | 42H | 51H | 67H | 93H | 98H | 102H | 113H | 125H | 132H | 138H | 141H | 143H | 153H | 156H | 157H | 166H | 168H | 170H | 184H | 186H | 188H | 190H | 197H | 198H | 200H | 206H |
|  | 1L | 4L | 5L | 9L | 12L | 13L | 18L | 24L | 27L | 29L | 32L | 33L | 34L | 37L | 38L | 39L | 41L | 42L | 45L | 46L | 48L | 51L | 52L | 53L | 57L | 60L | 65L | 70L | 72L | 74L |
|  |  |  |  |  |  |  |  |  |  |  |  |  |  |  |  |  |  |  |  |  |  |  |  |  |  |  |  |  |  |  |
| **99** | 12H | 21H | 29H | 56H | 57H | 61H | 74H | 86H | 88H | 101H | 105H | 109H | 111H | 113H | 114H | 123H | 135H | 136H | 138H | 141H | 142H | 156H | 157H | 162H | 172H | 194H | 196H | 206H | 207H | 208H |
|  | 1L | 4L | 5L | 9L | 12L | 13L | 18L | 24L | 27L | 29L | 32L | 33L | 34L | 37L | 38L | 39L | 41L | 42L | 45L | 46L | 48L | 51L | 52L | 53L | 57L | 60L | 65L | 70L | 72L | 74L |
|  |  |  |  |  |  |  |  |  |  |  |  |  |  |  |  |  |  |  |  |  |  |  |  |  |  |  |  |  |  |  |
| **100** | 19H | 21H | 22H | 24H | 42H | 44H | 56H | 69H | 70H | 71H | 76H | 78H | 82H | 91H | 102H | 109H | 111H | 118H | 125H | 128H | 144H | 147H | 154H | 164H | 174H | 184H | 186H | 189H | 193H | 200H |
|  | 1L | 4L | 5L | 9L | 12L | 13L | 18L | 24L | 27L | 29L | 32L | 33L | 34L | 37L | 38L | 39L | 41L | 42L | 45L | 46L | 48L | 51L | 52L | 53L | 57L | 60L | 65L | 70L | 72L | 74L |
