# Supplementary material for: A simple model for glioma grading based on texture analysis applied to conventional brain MRI
Source: PLoS One. 2020 May 15;15(5):e0228972. doi: 10.1371/journal.pone.0228972 (PMC7228074; doi:10.1371/journal.pone.0228972)
Supplement: S5 Table — (DOCX) [file pone.0228972.s005.docx]

**T_1Gd_^1^**

| **HGG** | ***F*_szm.sze_** | ***F*_szm.lze_** | ***F*_szm.glnu_** | ***F*_szm.zsnu_** | ***F*_szm.z.perc_** | ***F*_szm.lgze_** | ***F*_szm.hgze_** | ***F*_szm.szlge_** | ***F*_szm.szhge_** | ***F*_szm.lzlge_** | ***F*_szm.lzhge_** | ***F*_szm.gl.var_** | ***F*_szm.zs.var_** |
| --- | --- | --- | --- | --- | --- | --- | --- | --- | --- | --- | --- | --- | --- |
| **1H** | 7.618E-01 | 4.959E+02 | 1.962E-02 | 5.388E-01 | 3.503E-01 | 3.064E-04 | 4.157E+03 | 2.189E-04 | 3.391E+03 | 2.415E-01 | 1.045E+06 | 3.917E+03 | 8.150E+00 |
| **2H** | 6.262E-01 | 1.010E+03 | 3.761E-02 | 3.638E-01 | 2.414E-01 | 1.995E-04 | 5.558E+03 | 1.211E-04 | 3.587E+03 | 2.958E-01 | 3.678E+06 | 5.429E+03 | 1.716E+01 |
| **4H** | 8.844E-01 | 1.839E+00 | 2.597E-02 | 7.416E-01 | 8.281E-01 | 1.816E-04 | 6.128E+03 | 1.556E-04 | 5.556E+03 | 3.789E-04 | 1.015E+04 | 5.966E+03 | 1.455E+00 |
| **7H** | 8.676E-01 | 2.652E+00 | 1.800E-02 | 7.118E-01 | 7.721E-01 | 1.793E-04 | 6.729E+03 | 1.523E-04 | 5.987E+03 | 5.312E-04 | 1.511E+04 | 6.447E+03 | 1.676E+00 |
| **8H** | 7.745E-01 | 1.660E+03 | 1.672E-02 | 5.575E-01 | 3.475E-01 | 9.124E-04 | 2.274E+03 | 6.637E-04 | 1.884E+03 | 3.577E+00 | 7.889E+05 | 1.976E+03 | 8.281E+00 |
| **9H** | 7.162E-01 | 3.358E+01 | 1.722E-02 | 4.743E-01 | 4.926E-01 | 3.208E-04 | 4.371E+03 | 2.229E-04 | 3.231E+03 | 2.136E-02 | 6.608E+04 | 4.108E+03 | 4.121E+00 |
| **12H** | 7.640E-01 | 6.331E+02 | 2.461E-02 | 5.418E-01 | 3.966E-01 | 6.483E-04 | 2.140E+03 | 4.661E-04 | 1.770E+03 | 6.511E-01 | 6.387E+05 | 1.967E+03 | 6.357E+00 |
| **13H** | 6.990E-01 | 1.572E+01 | 3.073E-02 | 4.516E-01 | 4.743E-01 | 3.397E-04 | 3.325E+03 | 2.320E-04 | 2.415E+03 | 5.892E-03 | 4.360E+04 | 3.219E+03 | 4.445E+00 |
| **14H** | 8.821E-01 | 1.798E+00 | 3.810E-02 | 7.362E-01 | 8.323E-01 | 1.580E-04 | 6.619E+03 | 1.380E-04 | 5.903E+03 | 3.011E-04 | 1.120E+04 | 6.532E+03 | 1.440E+00 |
| **16H** | 8.526E-01 | 4.138E+00 | 1.711E-02 | 6.836E-01 | 7.510E-01 | 2.838E-04 | 4.761E+03 | 2.337E-04 | 4.162E+03 | 2.352E-03 | 1.155E+04 | 4.478E+03 | 1.773E+00 |
| **17H** | 7.168E-01 | 5.142E+01 | 3.218E-02 | 4.751E-01 | 4.486E-01 | 3.725E-04 | 7.229E+03 | 3.282E-04 | 5.302E+03 | 8.324E-03 | 3.323E+05 | 7.104E+03 | 4.969E+00 |
| **19H** | 8.813E-01 | 1.816E+00 | 4.180E-02 | 7.353E-01 | 8.273E-01 | 2.207E-04 | 4.724E+03 | 1.936E-04 | 4.193E+03 | 4.113E-04 | 8.291E+03 | 4.661E+03 | 1.457E+00 |
| **20H** | 7.981E-01 | 4.815E+01 | 1.602E-02 | 5.942E-01 | 5.567E-01 | 3.801E-04 | 3.844E+03 | 2.840E-04 | 3.252E+03 | 3.698E-02 | 6.946E+04 | 3.531E+03 | 3.226E+00 |
| **21H** | 8.247E-01 | 2.931E+00 | 1.406E-02 | 6.360E-01 | 7.237E-01 | 2.512E-04 | 5.616E+03 | 2.016E-04 | 4.819E+03 | 8.138E-04 | 1.367E+04 | 5.190E+03 | 1.908E+00 |
| **22H** | 7.338E-01 | 5.080E+01 | 2.502E-02 | 4.989E-01 | 4.487E-01 | 5.638E-04 | 2.502E+03 | 3.889E-04 | 2.030E+03 | 3.479E-02 | 7.633E+04 | 2.264E+03 | 4.967E+00 |
| **23H** | 8.296E-01 | 2.227E+00 | 1.967E-02 | 6.428E-01 | 7.640E-01 | 2.358E-04 | 5.424E+03 | 1.936E-04 | 4.576E+03 | 5.760E-04 | 1.093E+04 | 5.153E+03 | 1.709E+00 |
| **24H** | 7.942E-01 | 7.708E+00 | 1.689E-02 | 5.869E-01 | 6.354E-01 | 2.833E-04 | 4.674E+03 | 2.152E-04 | 3.850E+03 | 3.374E-03 | 2.312E+04 | 4.396E+03 | 2.476E+00 |
| **25H** | 8.370E-01 | 2.751E+00 | 2.245E-02 | 6.566E-01 | 7.409E-01 | 1.576E-04 | 7.140E+03 | 1.301E-04 | 6.073E+03 | 4.588E-04 | 1.838E+04 | 6.951E+03 | 1.820E+00 |
| **26H** | 7.732E-01 | 4.587E+00 | 2.078E-02 | 5.545E-01 | 6.375E-01 | 2.317E-04 | 5.185E+03 | 1.752E-04 | 4.087E+03 | 1.272E-03 | 2.080E+04 | 4.989E+03 | 2.460E+00 |
| **27H** | 8.078E-01 | 4.067E+00 | 1.770E-02 | 6.082E-01 | 6.825E-01 | 3.387E-04 | 3.974E+03 | 2.625E-04 | 3.359E+03 | 1.765E-03 | 1.183E+04 | 3.710E+03 | 2.146E+00 |
| **28H** | 9.224E-01 | 1.555E+00 | 4.182E-02 | 8.232E-01 | 8.726E-01 | 1.856E-04 | 5.735E+03 | 1.701E-04 | 5.317E+03 | 2.961E-04 | 8.755E+03 | 5.628E+03 | 1.306E+00 |
| **29H** | 7.570E-01 | 7.177E+00 | 2.598E-02 | 5.311E-01 | 5.816E-01 | 1.923E-04 | 5.737E+03 | 1.438E-04 | 4.437E+03 | 1.451E-03 | 3.732E+04 | 5.604E+03 | 2.956E+00 |
| **30H** | 8.570E-01 | 1.931E+00 | 2.676E-02 | 6.901E-01 | 8.023E-01 | 2.070E-04 | 5.544E+03 | 1.726E-04 | 4.870E+03 | 4.515E-04 | 9.547E+03 | 5.365E+03 | 1.548E+00 |
| **31H** | 8.780E-01 | 1.958E+00 | 1.696E-02 | 7.294E-01 | 8.177E-01 | 1.686E-04 | 7.739E+03 | 1.457E-04 | 6.877E+03 | 3.491E-04 | 1.452E+04 | 7.369E+03 | 1.494E+00 |
| **32H** | 8.273E-01 | 4.883E+00 | 1.844E-02 | 6.415E-01 | 6.866E-01 | 3.453E-04 | 3.902E+03 | 2.625E-04 | 3.384E+03 | 3.032E-03 | 1.092E+04 | 3.667E+03 | 2.120E+00 |
| **33H** | 7.761E-01 | 2.490E+01 | 1.840E-02 | 5.599E-01 | 5.401E-01 | 4.398E-04 | 3.239E+03 | 3.213E-04 | 2.692E+03 | 1.561E-02 | 4.245E+04 | 2.968E+03 | 3.428E+00 |
| **34H** | 8.599E-01 | 2.978E+00 | 1.468E-02 | 6.971E-01 | 7.637E-01 | 1.788E-04 | 7.389E+03 | 1.474E-04 | 6.523E+03 | 7.789E-04 | 1.597E+04 | 6.980E+03 | 1.714E+00 |
| **35H** | 7.708E-01 | 1.104E+01 | 2.800E-02 | 5.520E-01 | 5.640E-01 | 1.742E-04 | 6.459E+03 | 1.334E-04 | 5.051E+03 | 1.781E-03 | 7.120E+04 | 6.304E+03 | 3.143E+00 |
| **36H** | 7.100E-01 | 2.048E+01 | 2.408E-02 | 4.660E-01 | 4.548E-01 | 9.385E-05 | 1.118E+04 | 6.567E-05 | 8.085E+03 | 2.106E-03 | 2.037E+05 | 1.104E+04 | 4.834E+00 |
| **37H** | 8.288E-01 | 3.425E+00 | 1.530E-02 | 6.429E-01 | 7.154E-01 | 2.204E-04 | 5.918E+03 | 1.738E-04 | 5.096E+03 | 1.017E-03 | 1.520E+04 | 5.576E+03 | 1.953E+00 |
| **38H** | 8.688E-01 | 1.946E+00 | 1.428E-02 | 7.119E-01 | 8.094E-01 | 2.233E-04 | 6.778E+03 | 1.885E-04 | 5.997E+03 | 5.116E-04 | 1.129E+04 | 6.171E+03 | 1.523E+00 |
| **39H** | 7.735E-01 | 4.260E+00 | 2.091E-02 | 5.549E-01 | 6.441E-01 | 2.259E-04 | 5.693E+03 | 1.735E-04 | 4.421E+03 | 9.718E-04 | 2.441E+04 | 5.441E+03 | 2.409E+00 |
| **40H** | 7.609E-01 | 6.569E+00 | 1.847E-02 | 5.365E-01 | 6.070E-01 | 2.507E-04 | 4.997E+03 | 1.871E-04 | 3.894E+03 | 2.289E-03 | 2.434E+04 | 4.767E+03 | 2.714E+00 |
| **41H** | 7.478E-01 | 2.040E+01 | 4.920E-02 | 5.185E-01 | 4.771E-01 | 1.390E-04 | 7.466E+03 | 1.039E-04 | 5.610E+03 | 2.792E-03 | 1.501E+05 | 7.405E+03 | 4.392E+00 |
| **42H** | 7.817E-01 | 1.002E+01 | 1.835E-02 | 5.681E-01 | 5.868E-01 | 2.745E-04 | 4.689E+03 | 2.085E-04 | 3.787E+03 | 2.982E-03 | 3.859E+04 | 4.442E+03 | 2.903E+00 |
| **43H** | 7.137E-01 | 5.647E+01 | 2.674E-02 | 4.709E-01 | 4.585E-01 | 1.587E-04 | 6.839E+03 | 1.115E-04 | 5.003E+03 | 8.569E-03 | 3.747E+05 | 6.719E+03 | 4.757E+00 |
| **44H** | 8.228E-01 | 5.870E+00 | 2.076E-02 | 6.336E-01 | 6.712E-01 | 2.515E-04 | 4.730E+03 | 1.980E-04 | 4.034E+03 | 2.141E-03 | 1.847E+04 | 4.548E+03 | 2.219E+00 |
| **45H** | 7.362E-01 | 3.150E+01 | 2.362E-02 | 5.020E-01 | 4.621E-01 | 3.465E-04 | 3.683E+03 | 2.448E-04 | 2.922E+03 | 1.227E-02 | 8.298E+04 | 3.442E+03 | 4.683E+00 |
| **47H** | 7.648E-01 | 7.866E+00 | 2.059E-02 | 5.426E-01 | 5.954E-01 | 3.478E-04 | 3.653E+03 | 2.574E-04 | 2.931E+03 | 3.216E-03 | 2.208E+04 | 3.454E+03 | 2.820E+00 |
| **49H** | 8.603E-01 | 2.054E+00 | 1.533E-02 | 6.967E-01 | 7.944E-01 | 2.168E-04 | 6.033E+03 | 1.808E-04 | 5.343E+03 | 5.058E-04 | 1.085E+04 | 5.676E+03 | 1.581E+00 |
| **50H** | 7.874E-01 | 6.601E+00 | 3.618E-02 | 5.768E-01 | 6.259E-01 | 1.570E-04 | 7.188E+03 | 1.248E-04 | 5.673E+03 | 9.876E-04 | 4.583E+04 | 7.062E+03 | 2.552E+00 |
| **51H** | 8.133E-01 | 7.006E+00 | 1.812E-02 | 6.172E-01 | 6.657E-01 | 3.225E-04 | 4.180E+03 | 2.516E-04 | 3.519E+03 | 3.953E-03 | 1.605E+04 | 3.937E+03 | 2.256E+00 |
| **52H** | 7.590E-01 | 5.186E+00 | 2.133E-02 | 5.338E-01 | 6.123E-01 | 2.632E-04 | 4.506E+03 | 1.962E-04 | 3.512E+03 | 1.511E-03 | 2.007E+04 | 4.332E+03 | 2.665E+00 |
| **53H** | 8.429E-01 | 4.710E+00 | 1.680E-02 | 6.678E-01 | 7.121E-01 | 2.560E-04 | 5.271E+03 | 2.027E-04 | 4.621E+03 | 2.143E-03 | 1.450E+04 | 4.955E+03 | 1.972E+00 |
| **54H** | 7.353E-01 | 1.071E+01 | 2.606E-02 | 5.003E-01 | 5.431E-01 | 2.429E-04 | 4.581E+03 | 1.759E-04 | 3.445E+03 | 2.835E-03 | 4.236E+04 | 4.468E+03 | 3.389E+00 |
| **55H** | 8.019E-01 | 2.929E+00 | 1.845E-02 | 5.981E-01 | 7.104E-01 | 2.135E-04 | 6.034E+03 | 1.722E-04 | 4.845E+03 | 6.011E-04 | 1.780E+04 | 5.761E+03 | 1.981E+00 |
| **56H** | 8.311E-01 | 2.945E+00 | 1.764E-02 | 6.478E-01 | 7.214E-01 | 3.202E-04 | 4.328E+03 | 2.490E-04 | 3.828E+03 | 1.292E-03 | 8.813E+03 | 3.987E+03 | 1.918E+00 |
| **57H** | 8.236E-01 | 4.216E+00 | 1.521E-02 | 6.347E-01 | 6.905E-01 | 3.513E-04 | 4.226E+03 | 2.764E-04 | 3.640E+03 | 1.998E-03 | 1.217E+04 | 3.860E+03 | 2.096E+00 |
| **58H** | 8.240E-01 | 4.240E+00 | 1.663E-02 | 6.350E-01 | 6.998E-01 | 2.594E-04 | 5.129E+03 | 2.059E-04 | 4.370E+03 | 1.296E-03 | 1.850E+04 | 4.818E+03 | 2.041E+00 |
| **59H** | 7.572E-01 | 9.946E+00 | 1.936E-02 | 5.315E-01 | 5.520E-01 | 2.999E-04 | 4.232E+03 | 2.148E-04 | 3.412E+03 | 3.745E-03 | 2.911E+04 | 3.981E+03 | 3.281E+00 |
| **60H** | 7.689E-01 | 6.132E+00 | 1.876E-02 | 5.484E-01 | 6.111E-01 | 3.123E-04 | 4.160E+03 | 2.325E-04 | 3.376E+03 | 2.229E-03 | 1.910E+04 | 3.896E+03 | 2.677E+00 |
| **61H** | 8.007E-01 | 1.223E+01 | 1.408E-02 | 5.980E-01 | 6.170E-01 | 2.725E-04 | 5.454E+03 | 2.070E-04 | 4.583E+03 | 5.323E-03 | 3.409E+04 | 5.007E+03 | 2.627E+00 |
| **62H** | 7.417E-01 | 8.405E+01 | 2.382E-02 | 5.094E-01 | 4.626E-01 | 2.279E-04 | 4.965E+03 | 1.640E-04 | 3.826E+03 | 2.300E-02 | 3.100E+05 | 4.814E+03 | 4.672E+00 |
| **64H** | 7.748E-01 | 3.410E+00 | 4.713E-02 | 5.566E-01 | 6.741E-01 | 1.539E-04 | 6.687E+03 | 1.195E-04 | 5.187E+03 | 5.119E-04 | 2.307E+04 | 6.631E+03 | 2.197E+00 |
| **65H** | 8.412E-01 | 4.152E+00 | 1.534E-02 | 6.644E-01 | 7.180E-01 | 2.253E-04 | 5.847E+03 | 1.829E-04 | 5.106E+03 | 1.281E-03 | 1.650E+04 | 5.502E+03 | 1.939E+00 |
| **66H** | 8.964E-01 | 1.674E+00 | 2.468E-02 | 7.657E-01 | 8.466E-01 | 1.319E-04 | 8.452E+03 | 1.178E-04 | 7.611E+03 | 2.241E-04 | 1.378E+04 | 8.215E+03 | 1.390E+00 |
| **67H** | 7.856E-01 | 4.702E+00 | 1.907E-02 | 5.733E-01 | 6.504E-01 | 2.587E-04 | 4.819E+03 | 1.998E-04 | 3.891E+03 | 1.307E-03 | 1.942E+04 | 4.595E+03 | 2.363E+00 |
| **68H** | 7.882E-01 | 1.215E+01 | 1.924E-02 | 5.781E-01 | 5.754E-01 | 3.829E-04 | 3.671E+03 | 2.799E-04 | 3.090E+03 | 7.253E-03 | 2.297E+04 | 3.390E+03 | 3.020E+00 |
| **69H** | 8.318E-01 | 2.433E+00 | 2.960E-02 | 6.471E-01 | 7.525E-01 | 1.361E-04 | 7.964E+03 | 1.132E-04 | 6.651E+03 | 3.368E-04 | 1.886E+04 | 7.828E+03 | 1.763E+00 |
| **70H** | 7.689E-01 | 2.190E+01 | 2.250E-02 | 5.485E-01 | 5.689E-01 | 1.969E-04 | 5.837E+03 | 1.503E-04 | 4.558E+03 | 3.656E-03 | 1.353E+05 | 5.665E+03 | 3.089E+00 |
| **71H** | 8.330E-01 | 3.332E+00 | 1.780E-02 | 6.502E-01 | 7.179E-01 | 2.018E-04 | 5.977E+03 | 1.632E-04 | 5.138E+03 | 8.014E-04 | 1.591E+04 | 5.715E+03 | 1.939E+00 |
| **72H** | 8.368E-01 | 2.890E+00 | 1.504E-02 | 6.559E-01 | 7.427E-01 | 3.399E-04 | 4.389E+03 | 2.754E-04 | 3.812E+03 | 1.223E-03 | 9.539E+03 | 4.023E+03 | 1.812E+00 |
| **73H** | 7.560E-01 | 1.111E+01 | 2.018E-02 | 5.300E-01 | 5.463E-01 | 1.713E-04 | 6.752E+03 | 1.276E-04 | 5.231E+03 | 1.796E-03 | 7.460E+04 | 6.535E+03 | 3.350E+00 |
| **74H** | 8.503E-01 | 3.592E+00 | 1.733E-02 | 6.808E-01 | 7.370E-01 | 2.701E-04 | 4.862E+03 | 2.211E-04 | 4.308E+03 | 1.201E-03 | 1.275E+04 | 4.573E+03 | 1.840E+00 |
| **75H** | 8.166E-01 | 9.638E+00 | 1.823E-02 | 6.242E-01 | 6.185E-01 | 2.047E-04 | 6.207E+03 | 1.551E-04 | 5.335E+03 | 3.502E-03 | 3.083E+04 | 5.897E+03 | 2.613E+00 |
| **76H** | 7.415E-01 | 9.486E+00 | 2.399E-02 | 5.091E-01 | 5.414E-01 | 1.623E-04 | 6.700E+03 | 1.168E-04 | 5.135E+03 | 1.750E-03 | 5.350E+04 | 6.566E+03 | 3.411E+00 |
| **78H** | 7.340E-01 | 4.820E+00 | 2.216E-02 | 4.985E-01 | 6.006E-01 | 3.083E-04 | 4.004E+03 | 2.295E-04 | 2.958E+03 | 1.440E-03 | 1.867E+04 | 3.836E+03 | 2.770E+00 |
| **79H** | 7.700E-01 | 7.379E+00 | 3.948E-02 | 5.508E-01 | 5.805E-01 | 1.737E-04 | 6.306E+03 | 1.327E-04 | 4.888E+03 | 1.221E-03 | 4.731E+04 | 6.190E+03 | 2.966E+00 |
| **80H** | 7.387E-01 | 7.153E+01 | 2.038E-02 | 5.053E-01 | 4.368E-01 | 3.518E-04 | 3.646E+03 | 2.474E-04 | 2.856E+03 | 3.388E-02 | 1.558E+05 | 3.421E+03 | 5.242E+00 |
| **81H** | 7.608E-01 | 8.110E+02 | 3.696E-02 | 5.368E-01 | 2.956E-01 | 1.243E-04 | 8.319E+03 | 9.360E-05 | 6.405E+03 | 1.024E-01 | 6.433E+06 | 8.254E+03 | 1.145E+01 |
| **82H** | 7.749E-01 | 6.488E+00 | 1.709E-02 | 5.575E-01 | 6.083E-01 | 2.190E-04 | 5.643E+03 | 1.643E-04 | 4.570E+03 | 1.652E-03 | 2.820E+04 | 5.361E+03 | 2.702E+00 |
| **83H** | 8.625E-01 | 2.208E+00 | 1.839E-02 | 7.013E-01 | 7.877E-01 | 2.967E-04 | 4.754E+03 | 2.484E-04 | 4.217E+03 | 7.916E-04 | 8.738E+03 | 4.448E+03 | 1.609E+00 |
| **84H** | 7.647E-01 | 1.158E+01 | 1.669E-02 | 5.427E-01 | 5.567E-01 | 2.533E-04 | 5.131E+03 | 1.885E-04 | 4.096E+03 | 3.441E-03 | 4.365E+04 | 4.841E+03 | 3.226E+00 |
| **85H** | 8.487E-01 | 2.296E+00 | 1.815E-02 | 6.767E-01 | 7.718E-01 | 1.908E-04 | 6.281E+03 | 1.554E-04 | 5.536E+03 | 5.067E-04 | 1.217E+04 | 6.016E+03 | 1.676E+00 |
| **86H** | 7.757E-01 | 3.330E+01 | 1.882E-02 | 5.589E-01 | 5.474E-01 | 3.116E-04 | 4.104E+03 | 2.274E-04 | 3.372E+03 | 1.626E-02 | 7.134E+04 | 3.864E+03 | 3.337E+00 |
| **87H** | 7.990E-01 | 6.462E+00 | 1.421E-02 | 5.951E-01 | 6.303E-01 | 1.582E-04 | 7.825E+03 | 1.191E-04 | 6.584E+03 | 1.484E-03 | 3.295E+04 | 7.442E+03 | 2.517E+00 |
| **88H** | 7.564E-01 | 3.075E+01 | 2.099E-02 | 5.308E-01 | 4.975E-01 | 2.785E-04 | 4.302E+03 | 2.031E-04 | 3.408E+03 | 1.101E-02 | 8.999E+04 | 4.115E+03 | 4.040E+00 |
| **89H** | 7.803E-01 | 2.224E+01 | 1.896E-02 | 5.667E-01 | 5.354E-01 | 2.726E-04 | 4.544E+03 | 2.025E-04 | 3.721E+03 | 9.423E-03 | 5.725E+04 | 4.326E+03 | 3.488E+00 |
| **90H** | 7.563E-01 | 9.821E+00 | 2.174E-02 | 5.299E-01 | 5.693E-01 | 2.380E-04 | 4.967E+03 | 1.753E-04 | 3.843E+03 | 2.895E-03 | 4.087E+04 | 4.787E+03 | 3.085E+00 |
| **91H** | 7.481E-01 | 3.941E+01 | 2.569E-02 | 5.185E-01 | 4.826E-01 | 2.547E-04 | 4.430E+03 | 1.849E-04 | 3.415E+03 | 1.319E-02 | 1.218E+05 | 4.309E+03 | 4.293E+00 |
| **92H** | 8.040E-01 | 3.460E+00 | 2.179E-02 | 6.022E-01 | 6.885E-01 | 1.812E-04 | 6.459E+03 | 1.436E-04 | 5.271E+03 | 6.553E-04 | 2.116E+04 | 6.248E+03 | 2.107E+00 |
| **93H** | 7.522E-01 | 3.334E+02 | 2.755E-02 | 5.248E-01 | 3.830E-01 | 1.714E-04 | 6.386E+03 | 1.270E-04 | 4.911E+03 | 5.260E-02 | 2.118E+06 | 6.261E+03 | 6.815E+00 |
| **94H** | 7.423E-01 | 6.676E+00 | 2.991E-02 | 5.099E-01 | 5.740E-01 | 1.847E-04 | 5.830E+03 | 1.360E-04 | 4.393E+03 | 1.271E-03 | 3.669E+04 | 5.725E+03 | 3.034E+00 |
| **95H** | 7.079E-01 | 2.802E+02 | 2.327E-02 | 4.633E-01 | 4.117E-01 | 2.703E-04 | 4.327E+03 | 1.860E-04 | 3.179E+03 | 8.011E-02 | 9.886E+05 | 4.179E+03 | 5.901E+00 |
| **96H** | 7.870E-01 | 5.197E+00 | 2.502E-02 | 5.756E-01 | 6.466E-01 | 2.653E-04 | 4.809E+03 | 2.066E-04 | 3.795E+03 | 1.299E-03 | 2.676E+04 | 4.612E+03 | 2.391E+00 |
| **97H** | 8.474E-01 | 2.985E+00 | 1.446E-02 | 6.751E-01 | 7.454E-01 | 2.643E-04 | 5.371E+03 | 2.143E-04 | 4.785E+03 | 9.754E-04 | 1.186E+04 | 4.959E+03 | 1.799E+00 |
| **98H** | 8.821E-01 | 1.843E+00 | 1.793E-02 | 7.373E-01 | 8.251E-01 | 1.698E-04 | 7.498E+03 | 1.492E-04 | 6.679E+03 | 3.255E-04 | 1.298E+04 | 7.174E+03 | 1.466E+00 |
| **99H** | 7.994E-01 | 4.573E+00 | 2.830E-02 | 5.953E-01 | 6.597E-01 | 1.256E-04 | 8.437E+03 | 1.016E-04 | 6.718E+03 | 5.426E-04 | 3.945E+04 | 8.323E+03 | 2.296E+00 |
| **101H** | 7.255E-01 | 2.213E+01 | 2.414E-02 | 4.870E-01 | 4.664E-01 | 3.701E-04 | 3.307E+03 | 2.563E-04 | 2.562E+03 | 9.239E-03 | 5.568E+04 | 3.136E+03 | 4.596E+00 |
| **102H** | 8.331E-01 | 3.103E+00 | 2.108E-02 | 6.501E-01 | 7.288E-01 | 2.655E-04 | 4.473E+03 | 2.162E-04 | 3.828E+03 | 9.704E-04 | 1.138E+04 | 4.291E+03 | 1.881E+00 |
| **103H** | 8.388E-01 | 5.094E+00 | 1.675E-02 | 6.612E-01 | 6.971E-01 | 3.544E-04 | 3.960E+03 | 2.788E-04 | 3.486E+03 | 2.983E-03 | 1.145E+04 | 3.668E+03 | 2.057E+00 |
| **104H** | 8.523E-01 | 2.447E+00 | 1.607E-02 | 6.829E-01 | 7.702E-01 | 1.767E-04 | 7.437E+03 | 1.456E-04 | 6.487E+03 | 5.440E-04 | 1.523E+04 | 7.052E+03 | 1.684E+00 |
| **105H** | 7.903E-01 | 4.414E+00 | 2.336E-02 | 5.809E-01 | 6.475E-01 | 1.788E-04 | 6.845E+03 | 1.399E-04 | 5.472E+03 | 7.575E-04 | 2.996E+04 | 6.597E+03 | 2.383E+00 |
| **108H** | 7.945E-01 | 4.479E+00 | 2.421E-02 | 5.875E-01 | 6.567E-01 | 2.320E-04 | 4.906E+03 | 1.792E-04 | 4.022E+03 | 1.226E-03 | 1.796E+04 | 4.752E+03 | 2.318E+00 |
| **109H** | 7.176E-01 | 1.155E+02 | 2.822E-02 | 4.761E-01 | 4.407E-01 | 1.684E-04 | 6.360E+03 | 1.175E-04 | 4.692E+03 | 2.847E-02 | 4.741E+05 | 6.254E+03 | 5.148E+00 |
| **110H** | 7.078E-01 | 1.543E+01 | 3.431E-02 | 4.633E-01 | 4.922E-01 | 2.150E-04 | 4.946E+03 | 1.495E-04 | 3.568E+03 | 4.076E-03 | 6.099E+04 | 4.875E+03 | 4.126E+00 |
| **111H** | 8.109E-01 | 2.960E+00 | 2.531E-02 | 6.127E-01 | 7.142E-01 | 1.478E-04 | 7.320E+03 | 1.176E-04 | 5.992E+03 | 5.015E-04 | 1.974E+04 | 7.184E+03 | 1.958E+00 |
| **112H** | 7.851E-01 | 4.261E+00 | 1.804E-02 | 5.726E-01 | 6.544E-01 | 4.910E-04 | 3.112E+03 | 3.747E-04 | 2.548E+03 | 2.446E-03 | 1.016E+04 | 2.863E+03 | 2.334E+00 |
| **113H** | 6.976E-01 | 1.321E+01 | 4.942E-02 | 4.501E-01 | 4.941E-01 | 1.561E-04 | 6.553E+03 | 1.079E-04 | 4.620E+03 | 2.224E-03 | 7.962E+04 | 6.517E+03 | 4.095E+00 |
| **114H** | 8.087E-01 | 1.241E+01 | 2.364E-02 | 6.101E-01 | 6.347E-01 | 2.272E-04 | 5.127E+03 | 1.781E-04 | 4.253E+03 | 4.243E-03 | 3.944E+04 | 4.959E+03 | 2.482E+00 |
| **115H** | 7.270E-01 | 1.544E+01 | 2.806E-02 | 4.891E-01 | 4.995E-01 | 2.039E-04 | 5.328E+03 | 1.435E-04 | 3.988E+03 | 4.726E-03 | 5.543E+04 | 5.225E+03 | 4.006E+00 |
| **116H** | 7.249E-01 | 6.179E+01 | 3.410E-02 | 4.862E-01 | 4.285E-01 | 2.459E-04 | 4.415E+03 | 1.708E-04 | 3.324E+03 | 2.344E-02 | 1.663E+05 | 4.331E+03 | 5.445E+00 |
| **117H** | 7.579E-01 | 5.677E+00 | 3.460E-02 | 5.322E-01 | 6.085E-01 | 2.574E-04 | 4.209E+03 | 1.935E-04 | 3.235E+03 | 1.486E-03 | 2.263E+04 | 4.134E+03 | 2.699E+00 |
| **118H** | 8.135E-01 | 3.623E+00 | 1.834E-02 | 6.173E-01 | 6.988E-01 | 1.905E-04 | 6.453E+03 | 1.528E-04 | 5.340E+03 | 8.135E-04 | 2.021E+04 | 6.174E+03 | 2.047E+00 |
| **119H** | 7.299E-01 | 7.637E+00 | 3.990E-02 | 4.930E-01 | 5.617E-01 | 1.731E-04 | 6.003E+03 | 1.251E-04 | 4.430E+03 | 1.364E-03 | 4.373E+04 | 5.943E+03 | 3.168E+00 |
| **120H** | 8.196E-01 | 4.708E+00 | 2.159E-02 | 6.277E-01 | 6.909E-01 | 1.341E-04 | 8.159E+03 | 1.078E-04 | 6.806E+03 | 7.510E-04 | 3.183E+04 | 7.983E+03 | 2.094E+00 |
| **121H** | 6.369E-01 | 6.912E+01 | 3.586E-02 | 3.755E-01 | 3.117E-01 | 1.812E-04 | 6.131E+03 | 1.205E-04 | 3.894E+03 | 1.121E-02 | 4.323E+05 | 6.043E+03 | 1.029E+01 |
| **122H** | 6.762E-01 | 1.202E+04 | 5.249E-02 | 4.239E-01 | 1.398E-01 | 1.700E-04 | 6.012E+03 | 1.126E-04 | 4.152E+03 | 2.368E+00 | 6.102E+07 | 5.980E+03 | 5.116E+01 |
| **123H** | 6.749E-01 | 4.234E+03 | 3.938E-02 | 4.217E-01 | 1.983E-01 | 1.916E-04 | 5.429E+03 | 1.265E-04 | 3.744E+03 | 1.092E+00 | 1.651E+07 | 5.377E+03 | 2.544E+01 |
| **124H** | 7.205E-01 | 1.002E+01 | 2.477E-02 | 4.801E-01 | 5.387E-01 | 1.689E-04 | 6.532E+03 | 1.212E-04 | 4.744E+03 | 1.697E-03 | 6.265E+04 | 6.396E+03 | 3.446E+00 |
| **125H** | 6.783E-01 | 3.432E+01 | 2.774E-02 | 4.254E-01 | 4.109E-01 | 1.604E-04 | 6.675E+03 | 1.069E-04 | 4.622E+03 | 6.562E-03 | 1.861E+05 | 6.572E+03 | 5.923E+00 |
| **126H** | 6.860E-01 | 3.825E+02 | 2.959E-02 | 4.353E-01 | 3.169E-01 | 2.342E-04 | 4.757E+03 | 1.595E-04 | 3.266E+03 | 1.183E-01 | 1.531E+06 | 4.646E+03 | 9.957E+00 |
| **127H** | 8.918E-01 | 1.579E+00 | 2.192E-02 | 7.542E-01 | 8.560E-01 | 1.508E-04 | 7.991E+03 | 1.327E-04 | 7.194E+03 | 2.473E-04 | 1.214E+04 | 7.647E+03 | 1.360E+00 |
| **128H** | 8.437E-01 | 4.215E+00 | 1.367E-02 | 6.697E-01 | 7.046E-01 | 6.708E-04 | 3.374E+03 | 5.426E-04 | 3.033E+03 | 3.695E-03 | 7.715E+03 | 2.916E+03 | 2.013E+00 |
| **129H** | 7.152E-01 | 1.200E+03 | 2.360E-02 | 4.730E-01 | 3.536E-01 | 2.631E-04 | 4.352E+03 | 1.820E-04 | 3.226E+03 | 5.369E-01 | 2.700E+06 | 4.209E+03 | 8.000E+00 |
| **130H** | 6.499E-01 | 1.398E+04 | 3.055E-02 | 3.921E-01 | 1.306E-01 | 8.637E-05 | 1.194E+04 | 5.651E-05 | 7.726E+03 | 1.060E+00 | 1.858E+08 | 1.186E+04 | 5.860E+01 |
| **131H** | 7.008E-01 | 2.965E+01 | 2.889E-02 | 4.542E-01 | 4.446E-01 | 1.877E-04 | 5.739E+03 | 1.276E-04 | 4.138E+03 | 6.908E-03 | 1.314E+05 | 5.642E+03 | 5.059E+00 |
| **132H** | 6.405E-01 | 2.800E+02 | 6.459E-02 | 3.802E-01 | 2.529E-01 | 1.427E-04 | 7.094E+03 | 9.077E-05 | 4.584E+03 | 4.126E-02 | 1.904E+06 | 7.072E+03 | 1.563E+01 |
| **133H** | 9.480E-01 | 1.250E+00 | 5.274E-02 | 8.729E-01 | 9.286E-01 | 1.504E-04 | 6.787E+03 | 1.429E-04 | 6.423E+03 | 1.871E-04 | 8.512E+03 | 6.710E+03 | 1.152E+00 |
| **134H** | 8.290E-01 | 2.676E+00 | 1.594E-02 | 6.429E-01 | 7.368E-01 | 8.825E-04 | 2.565E+03 | 6.864E-04 | 2.252E+03 | 3.100E-03 | 5.072E+03 | 2.228E+03 | 1.840E+00 |
| **135H** | 7.989E-01 | 4.797E+00 | 1.945E-02 | 5.938E-01 | 6.680E-01 | 2.006E-04 | 6.126E+03 | 1.551E-04 | 5.103E+03 | 1.143E-03 | 2.269E+04 | 5.809E+03 | 2.240E+00 |
| **136H** | 6.978E-01 | 5.806E+02 | 2.742E-02 | 4.503E-01 | 3.321E-01 | 1.970E-04 | 5.505E+03 | 1.331E-04 | 3.961E+03 | 1.588E-01 | 2.135E+06 | 5.399E+03 | 9.065E+00 |
| **137H** | 6.973E-01 | 1.012E+01 | 2.282E-02 | 4.496E-01 | 5.081E-01 | 2.232E-04 | 5.156E+03 | 1.489E-04 | 3.778E+03 | 2.867E-03 | 3.983E+04 | 4.981E+03 | 3.872E+00 |
| **138H** | 6.049E-01 | 1.532E+03 | 3.673E-02 | 3.417E-01 | 2.111E-01 | 1.800E-04 | 5.802E+03 | 1.082E-04 | 3.530E+03 | 3.462E-01 | 6.912E+06 | 5.742E+03 | 2.243E+01 |
| **139H** | 8.806E-01 | 1.704E+00 | 2.058E-02 | 7.330E-01 | 8.367E-01 | 4.362E-04 | 3.399E+03 | 3.792E-04 | 3.019E+03 | 7.934E-04 | 5.331E+03 | 3.144E+03 | 1.423E+00 |
| **140H** | 6.767E-01 | 2.070E+01 | 2.712E-02 | 4.236E-01 | 4.237E-01 | 1.617E-04 | 6.816E+03 | 1.081E-04 | 4.709E+03 | 3.568E-03 | 1.251E+05 | 6.675E+03 | 5.568E+00 |
| **141H** | 7.933E-01 | 5.438E+00 | 3.254E-02 | 5.859E-01 | 6.353E-01 | 2.249E-04 | 4.908E+03 | 1.700E-04 | 4.016E+03 | 1.774E-03 | 1.946E+04 | 4.803E+03 | 2.475E+00 |
| **142H** | 6.617E-01 | 1.180E+02 | 2.013E-02 | 4.052E-01 | 3.536E-01 | 2.157E-04 | 5.411E+03 | 1.417E-04 | 3.626E+03 | 3.458E-02 | 4.227E+05 | 5.223E+03 | 7.996E+00 |
| **143H** | 7.806E-01 | 9.749E+00 | 1.746E-02 | 5.660E-01 | 6.068E-01 | 2.057E-04 | 6.072E+03 | 1.529E-04 | 4.893E+03 | 3.711E-03 | 3.321E+04 | 5.805E+03 | 2.715E+00 |
| **144H** | 8.376E-01 | 2.256E+00 | 5.107E-02 | 6.567E-01 | 7.669E-01 | 2.013E-04 | 5.141E+03 | 1.673E-04 | 4.337E+03 | 4.597E-04 | 1.137E+04 | 5.084E+03 | 1.695E+00 |
| **145H** | 6.859E-01 | 4.925E+01 | 3.231E-02 | 4.352E-01 | 3.845E-01 | 1.770E-04 | 6.038E+03 | 1.180E-04 | 4.277E+03 | 1.085E-02 | 2.266E+05 | 5.931E+03 | 6.763E+00 |
| **146H** | 7.639E-01 | 4.145E+00 | 6.085E-02 | 5.409E-01 | 6.382E-01 | 1.570E-04 | 6.461E+03 | 1.201E-04 | 4.932E+03 | 6.449E-04 | 2.692E+04 | 6.429E+03 | 2.451E+00 |
| **147H** | 7.513E-01 | 3.420E+01 | 2.720E-02 | 5.228E-01 | 5.249E-01 | 1.674E-04 | 6.394E+03 | 1.223E-04 | 4.927E+03 | 8.368E-03 | 1.438E+05 | 6.291E+03 | 3.630E+00 |
| **148H** | 7.479E-01 | 2.178E+01 | 3.348E-02 | 5.179E-01 | 5.382E-01 | 1.694E-04 | 6.213E+03 | 1.244E-04 | 4.721E+03 | 5.852E-03 | 8.599E+04 | 6.138E+03 | 3.451E+00 |
| **149H** | 7.235E-01 | 2.744E+01 | 2.960E-02 | 4.843E-01 | 5.026E-01 | 2.146E-04 | 5.017E+03 | 1.505E-04 | 3.731E+03 | 9.507E-03 | 8.369E+04 | 4.930E+03 | 3.959E+00 |
| **150H** | 7.807E-01 | 8.921E+00 | 1.789E-02 | 5.660E-01 | 6.185E-01 | 4.316E-04 | 3.660E+03 | 3.360E-04 | 2.914E+03 | 2.875E-03 | 3.361E+04 | 3.385E+03 | 2.613E+00 |
| **151H** | 7.351E-01 | 1.452E+02 | 1.987E-02 | 5.003E-01 | 4.499E-01 | 4.159E-04 | 3.214E+03 | 2.963E-04 | 2.443E+03 | 9.899E-02 | 2.245E+05 | 3.007E+03 | 4.940E+00 |
| **152H** | 7.420E-01 | 3.166E+01 | 2.547E-02 | 5.099E-01 | 4.745E-01 | 1.648E-04 | 6.539E+03 | 1.172E-04 | 5.044E+03 | 7.201E-03 | 1.434E+05 | 6.423E+03 | 4.442E+00 |
| **153H** | 7.554E-01 | 2.441E+01 | 2.533E-02 | 5.292E-01 | 5.031E-01 | 2.538E-04 | 5.815E+03 | 1.970E-04 | 4.475E+03 | 6.114E-03 | 1.044E+05 | 5.618E+03 | 3.950E+00 |
| **154H** | 6.230E-01 | 2.719E+02 | 3.145E-02 | 3.601E-01 | 2.671E-01 | 1.736E-04 | 6.307E+03 | 1.101E-04 | 3.935E+03 | 4.114E-02 | 1.831E+06 | 6.211E+03 | 1.402E+01 |
| **155H** | 6.733E-01 | 1.166E+01 | 5.609E-02 | 4.205E-01 | 4.805E-01 | 1.857E-04 | 5.650E+03 | 1.259E-04 | 3.757E+03 | 1.826E-03 | 8.353E+04 | 5.567E+03 | 4.329E+00 |
| **156H** | 7.786E-01 | 3.680E+00 | 1.597E-02 | 5.626E-01 | 6.643E-01 | 3.453E-04 | 4.179E+03 | 2.569E-04 | 3.401E+03 | 1.519E-03 | 1.208E+04 | 3.848E+03 | 2.263E+00 |
| **157H** | 8.010E-01 | 4.355E+00 | 1.670E-02 | 5.979E-01 | 6.629E-01 | 4.691E-04 | 3.271E+03 | 3.555E-04 | 2.779E+03 | 2.770E-03 | 9.515E+03 | 2.978E+03 | 2.275E+00 |
| **158H** | 7.705E-01 | 8.924E+00 | 2.427E-02 | 5.508E-01 | 5.941E-01 | 3.067E-04 | 4.116E+03 | 2.304E-04 | 3.256E+03 | 3.787E-03 | 2.555E+04 | 3.926E+03 | 2.833E+00 |
| **160H** | 7.184E-01 | 8.485E+00 | 2.201E-02 | 4.772E-01 | 5.360E-01 | 1.838E-04 | 6.118E+03 | 1.311E-04 | 4.421E+03 | 1.365E-03 | 5.904E+04 | 5.950E+03 | 3.479E+00 |
| **161H** | 8.349E-01 | 4.551E+00 | 1.367E-02 | 6.534E-01 | 7.069E-01 | 4.324E-04 | 4.362E+03 | 3.405E-04 | 3.791E+03 | 3.583E-03 | 1.063E+04 | 3.934E+03 | 2.000E+00 |
| **162H** | 7.236E-01 | 6.306E+00 | 2.889E-02 | 4.841E-01 | 5.618E-01 | 1.504E-04 | 7.332E+03 | 1.077E-04 | 5.344E+03 | 9.880E-04 | 4.471E+04 | 7.191E+03 | 3.166E+00 |
| **163H** | 7.405E-01 | 1.965E+01 | 3.599E-02 | 5.076E-01 | 5.092E-01 | 1.860E-04 | 5.639E+03 | 1.342E-04 | 4.288E+03 | 4.226E-03 | 9.291E+04 | 5.573E+03 | 3.856E+00 |
| **164H** | 7.390E-01 | 2.361E+01 | 3.137E-02 | 5.111E-01 | 3.891E-01 | 1.644E-04 | 6.642E+03 | 1.272E-04 | 4.746E+03 | 3.141E-03 | 1.803E+05 | 6.514E+03 | 6.598E+00 |
| **165H** | 6.948E-01 | 1.240E+01 | 2.895E-02 | 4.466E-01 | 4.899E-01 | 1.763E-04 | 6.212E+03 | 1.198E-04 | 4.424E+03 | 2.331E-03 | 6.959E+04 | 6.092E+03 | 4.164E+00 |
| **166H** | 9.378E-01 | 1.318E+00 | 1.185E-02 | 8.504E-01 | 9.130E-01 | 6.064E-04 | 4.592E+03 | 5.510E-04 | 4.362E+03 | 9.651E-04 | 5.709E+03 | 3.919E+03 | 1.194E+00 |
| **167H** | 7.692E-01 | 9.092E+00 | 2.599E-02 | 5.492E-01 | 5.718E-01 | 2.044E-04 | 5.366E+03 | 1.524E-04 | 4.259E+03 | 2.246E-03 | 3.862E+04 | 5.245E+03 | 3.058E+00 |
| **168H** | 6.991E-01 | 2.361E+03 | 5.448E-02 | 4.521E-01 | 1.907E-01 | 1.801E-04 | 5.692E+03 | 1.245E-04 | 4.038E+03 | 4.739E-01 | 1.180E+07 | 5.657E+03 | 2.750E+01 |
| **169H** | 8.138E-01 | 4.315E+00 | 2.508E-02 | 6.186E-01 | 6.774E-01 | 3.496E-04 | 3.574E+03 | 2.771E-04 | 2.995E+03 | 1.755E-03 | 1.259E+04 | 3.399E+03 | 2.178E+00 |
| **170H** | 6.557E-01 | 1.705E+01 | 5.122E-02 | 3.983E-01 | 4.233E-01 | 1.976E-04 | 5.191E+03 | 1.279E-04 | 3.453E+03 | 3.388E-03 | 8.707E+04 | 5.155E+03 | 5.577E+00 |
| **171H** | 7.087E-01 | 2.086E+01 | 3.776E-02 | 4.650E-01 | 5.053E-01 | 2.320E-04 | 4.590E+03 | 1.595E-04 | 3.338E+03 | 7.434E-03 | 6.207E+04 | 4.525E+03 | 3.916E+00 |
| **172H** | 7.124E-01 | 5.533E+01 | 4.825E-02 | 4.694E-01 | 3.809E-01 | 2.110E-04 | 4.917E+03 | 1.487E-04 | 3.556E+03 | 1.224E-02 | 2.526E+05 | 4.874E+03 | 6.891E+00 |
| **173H** | 6.801E-01 | 3.546E+01 | 2.527E-02 | 4.278E-01 | 4.139E-01 | 1.634E-04 | 6.619E+03 | 1.081E-04 | 4.639E+03 | 6.886E-03 | 1.876E+05 | 6.497E+03 | 5.836E+00 |
| **174H** | 6.365E-01 | 1.364E+02 | 3.947E-02 | 3.762E-01 | 3.489E-01 | 1.565E-04 | 6.598E+03 | 9.858E-05 | 4.244E+03 | 2.700E-02 | 7.006E+05 | 6.547E+03 | 8.214E+00 |
| **175H** | 7.018E-01 | 4.649E+02 | 3.161E-02 | 4.553E-01 | 3.702E-01 | 3.487E-04 | 3.195E+03 | 2.374E-04 | 2.314E+03 | 2.525E-01 | 8.640E+05 | 3.114E+03 | 7.295E+00 |
| **176H** | 7.068E-01 | 4.486E+01 | 2.968E-02 | 4.618E-01 | 4.667E-01 | 2.722E-04 | 4.251E+03 | 1.880E-04 | 3.042E+03 | 2.344E-02 | 1.147E+05 | 4.129E+03 | 4.591E+00 |
| **177H** | 7.264E-01 | 3.422E+01 | 3.411E-02 | 4.881E-01 | 4.637E-01 | 2.168E-04 | 4.906E+03 | 1.526E-04 | 3.678E+03 | 9.628E-03 | 1.244E+05 | 4.834E+03 | 4.651E+00 |
| **178H** | 7.306E-01 | 4.661E+02 | 2.512E-02 | 4.938E-01 | 3.736E-01 | 2.039E-04 | 5.389E+03 | 1.428E-04 | 4.088E+03 | 1.511E-01 | 1.445E+06 | 5.268E+03 | 7.164E+00 |
| **181H** | 6.892E-01 | 1.018E+01 | 3.059E-02 | 4.398E-01 | 5.038E-01 | 1.769E-04 | 6.148E+03 | 1.212E-04 | 4.255E+03 | 1.912E-03 | 6.148E+04 | 6.038E+03 | 3.938E+00 |
| **182H** | 6.974E-01 | 3.138E+02 | 2.977E-02 | 4.501E-01 | 3.871E-01 | 3.099E-04 | 3.571E+03 | 2.075E-04 | 2.581E+03 | 1.633E-01 | 6.087E+05 | 3.486E+03 | 6.675E+00 |
| **183H** | 1.000E+00 | 1.000E+00 | 3.033E-02 | 1.000E+00 | 1.000E+00 | 1.113E-04 | 1.136E+04 | 1.113E-04 | 1.136E+04 | 1.113E-04 | 1.136E+04 | 1.072E+04 | 9.861E-01 |
| **184H** | 6.924E-01 | 2.234E+01 | 3.004E-02 | 4.435E-01 | 4.634E-01 | 1.678E-04 | 6.352E+03 | 1.149E-04 | 4.454E+03 | 3.952E-03 | 1.299E+05 | 6.260E+03 | 4.657E+00 |
| **185H** | 6.938E-01 | 1.004E+01 | 8.376E-02 | 4.515E-01 | 5.245E-01 | 1.492E-04 | 6.761E+03 | 1.037E-04 | 4.683E+03 | 1.444E-03 | 7.001E+04 | 6.741E+03 | 3.632E+00 |
| **186H** | 6.889E-01 | 2.676E+03 | 3.462E-02 | 4.388E-01 | 2.281E-01 | 1.722E-04 | 6.217E+03 | 1.150E-04 | 4.452E+03 | 6.022E-01 | 1.192E+07 | 6.095E+03 | 1.921E+01 |
| **187H** | 6.928E-01 | 9.967E+02 | 3.042E-02 | 4.439E-01 | 3.508E-01 | 3.016E-04 | 3.664E+03 | 2.032E-04 | 2.619E+03 | 3.565E-01 | 2.832E+06 | 3.579E+03 | 8.124E+00 |
| **188H** | 7.247E-01 | 4.034E+00 | 5.510E-02 | 4.859E-01 | 6.120E-01 | 1.313E-04 | 7.739E+03 | 9.598E-05 | 5.572E+03 | 5.152E-04 | 3.192E+04 | 7.690E+03 | 2.664E+00 |
| **189H** | 6.780E-01 | 5.573E+02 | 4.416E-02 | 4.258E-01 | 2.807E-01 | 2.047E-04 | 5.088E+03 | 1.369E-04 | 3.508E+03 | 1.290E-01 | 2.416E+06 | 5.038E+03 | 1.270E+01 |
| **190H** | 6.923E-01 | 4.500E+02 | 3.426E-02 | 4.432E-01 | 3.127E-01 | 1.552E-04 | 6.737E+03 | 1.057E-04 | 4.744E+03 | 7.646E-02 | 2.747E+06 | 6.661E+03 | 1.023E+01 |
| **191H** | 7.059E-01 | 2.551E+02 | 2.069E-02 | 4.609E-01 | 4.014E-01 | 1.522E-04 | 7.308E+03 | 1.039E-04 | 5.317E+03 | 7.211E-02 | 9.226E+05 | 7.127E+03 | 6.207E+00 |
| **192H** | 8.964E-01 | 1.500E+00 | 7.840E-02 | 7.626E-01 | 8.667E-01 | 1.156E-04 | 8.755E+03 | 1.021E-04 | 7.959E+03 | 1.804E-04 | 1.261E+04 | 8.674E+03 | 1.323E+00 |
| **193H** | 6.050E-01 | 1.826E+04 | 3.433E-02 | 3.410E-01 | 1.481E-01 | 3.695E-04 | 2.998E+03 | 2.224E-04 | 1.841E+03 | 8.562E+00 | 3.938E+07 | 2.930E+03 | 4.561E+01 |
| **194H** | 6.736E-01 | 4.305E+01 | 4.912E-02 | 4.199E-01 | 3.799E-01 | 1.619E-04 | 6.330E+03 | 1.083E-04 | 4.304E+03 | 7.604E-03 | 2.479E+05 | 6.292E+03 | 6.929E+00 |
| **195H** | 7.619E-01 | 2.075E+01 | 3.342E-02 | 5.386E-01 | 5.043E-01 | 2.250E-04 | 4.745E+03 | 1.656E-04 | 3.730E+03 | 6.117E-03 | 7.222E+04 | 4.672E+03 | 3.931E+00 |
| **196H** | 6.129E-01 | 3.076E+03 | 4.032E-02 | 3.487E-01 | 1.825E-01 | 2.403E-04 | 4.423E+03 | 1.463E-04 | 2.741E+03 | 7.119E-01 | 1.337E+07 | 4.363E+03 | 3.001E+01 |
| **197H** | 7.646E-01 | 2.172E+01 | 1.733E-02 | 5.421E-01 | 5.621E-01 | 4.419E-04 | 3.405E+03 | 3.267E-04 | 2.731E+03 | 1.163E-02 | 4.412E+04 | 3.114E+03 | 3.165E+00 |
| **198H** | 7.452E-01 | 1.347E+01 | 3.219E-02 | 5.142E-01 | 5.318E-01 | 2.158E-04 | 4.966E+03 | 1.574E-04 | 3.792E+03 | 3.335E-03 | 5.607E+04 | 4.885E+03 | 3.535E+00 |
| **199H** | 9.471E-01 | 1.211E+00 | 1.890E-02 | 8.690E-01 | 9.342E-01 | 2.158E-04 | 6.039E+03 | 2.027E-04 | 5.757E+03 | 2.679E-04 | 7.165E+03 | 5.676E+03 | 1.138E+00 |
| **200H** | 7.637E-01 | 9.802E+00 | 2.355E-02 | 5.409E-01 | 5.717E-01 | 2.335E-04 | 5.009E+03 | 1.745E-04 | 3.949E+03 | 2.588E-03 | 4.102E+04 | 4.820E+03 | 3.059E+00 |
| **201H** | 6.734E-01 | 9.306E+03 | 3.450E-02 | 4.195E-01 | 2.037E-01 | 2.369E-04 | 4.490E+03 | 1.554E-04 | 3.101E+03 | 2.690E+00 | 3.227E+07 | 4.421E+03 | 2.411E+01 |
| **202H** | 7.635E-01 | 7.055E+00 | 2.987E-02 | 5.406E-01 | 5.912E-01 | 1.987E-04 | 5.441E+03 | 1.481E-04 | 4.250E+03 | 1.712E-03 | 3.132E+04 | 5.344E+03 | 2.860E+00 |
| **203H** | 7.556E-01 | 5.158E+01 | 1.950E-02 | 5.293E-01 | 4.704E-01 | 1.506E-04 | 7.475E+03 | 1.083E-04 | 5.921E+03 | 1.171E-02 | 2.329E+05 | 7.258E+03 | 4.518E+00 |
| **206H** | 7.263E-01 | 3.421E+01 | 1.839E-02 | 4.880E-01 | 4.915E-01 | 2.607E-04 | 4.839E+03 | 1.832E-04 | 3.646E+03 | 1.551E-02 | 8.566E+04 | 4.559E+03 | 4.140E+00 |
| **207H** | 8.219E-01 | 2.969E+00 | 2.553E-02 | 6.307E-01 | 7.240E-01 | 2.064E-04 | 5.608E+03 | 1.723E-04 | 4.578E+03 | 5.617E-04 | 1.733E+04 | 5.456E+03 | 1.906E+00 |
| **208H** | 7.403E-01 | 5.234E+01 | 1.716E-02 | 5.072E-01 | 5.147E-01 | 4.714E-04 | 3.729E+03 | 3.325E-04 | 2.795E+03 | 1.728E-01 | 4.698E+04 | 3.435E+03 | 3.774E+00 |
| **209H** | 7.820E-01 | 9.404E+02 | 1.632E-02 | 5.695E-01 | 3.763E-01 | 7.916E-04 | 2.522E+03 | 5.554E-04 | 2.163E+03 | 1.643E+00 | 5.483E+05 | 2.139E+03 | 7.064E+00 |
| **210H** | 7.551E-01 | 2.597E+01 | 1.859E-02 | 5.291E-01 | 4.776E-01 | 5.505E-04 | 2.709E+03 | 3.820E-04 | 2.207E+03 | 2.189E-02 | 3.574E+04 | 2.485E+03 | 4.384E+00 |

| **LGG** | ***F*_szm.sze_** | ***F*_szm.lze_** | ***F*_szm.glnu_** | ***F*_szm.zsnu_** | ***F*_szm.z.perc_** | ***F*_szm.lgze_** | ***F*_szm.hgze_** | ***F*_szm.szlge_** | ***F*_szm.szhge_** | ***F*_szm.lzlge_** | ***F*_szm.lzhge_** | ***F*_szm.gl.var_** | ***F*_szm.zs.var_** |
| --- | --- | --- | --- | --- | --- | --- | --- | --- | --- | --- | --- | --- | --- |
| **1L** | 7.575E-01 | 1.879E+01 | 1.493E-02 | 5.318E-01 | 5.534E-01 | 9.488E-04 | 4.812E+03 | 8.425E-04 | 3.697E+03 | 1.087E-02 | 4.647E+04 | 4.403E+03 | 3.265E+00 |
| **3L** | 5.926E-01 | 3.867E+01 | 3.238E-02 | 3.287E-01 | 3.269E-01 | 2.982E-04 | 3.714E+03 | 1.775E-04 | 2.211E+03 | 1.281E-02 | 1.240E+05 | 3.630E+03 | 9.357E+00 |
| **4L** | 5.338E-01 | 4.318E+03 | 5.826E-02 | 2.678E-01 | 8.468E-02 | 1.797E-04 | 5.776E+03 | 9.424E-05 | 3.142E+03 | 8.270E-01 | 2.290E+07 | 5.724E+03 | 1.394E+02 |
| **5L** | 6.089E-01 | 1.218E+04 | 4.536E-02 | 3.457E-01 | 1.123E-01 | 2.510E-04 | 4.493E+03 | 1.490E-04 | 2.828E+03 | 4.073E+00 | 3.779E+07 | 4.361E+03 | 7.928E+01 |
| **7L** | 5.967E-01 | 2.376E+02 | 3.296E-02 | 3.315E-01 | 2.556E-01 | 1.827E-04 | 5.921E+03 | 1.094E-04 | 3.565E+03 | 3.775E-02 | 1.538E+06 | 5.822E+03 | 1.531E+01 |
| **9L** | 6.294E-01 | 1.130E+02 | 3.027E-02 | 3.678E-01 | 3.144E-01 | 3.600E-04 | 3.149E+03 | 2.257E-04 | 2.021E+03 | 4.869E-02 | 2.735E+05 | 3.059E+03 | 1.011E+01 |
| **11L** | 6.132E-01 | 6.862E+01 | 6.829E-02 | 3.500E-01 | 2.987E-01 | 1.908E-04 | 5.339E+03 | 1.164E-04 | 3.300E+03 | 1.278E-02 | 3.699E+05 | 5.314E+03 | 1.121E+01 |
| **12L** | 7.059E-01 | 9.885E+00 | 4.053E-02 | 4.617E-01 | 5.148E-01 | 1.620E-04 | 6.399E+03 | 1.140E-04 | 4.549E+03 | 1.594E-03 | 6.224E+04 | 6.340E+03 | 3.772E+00 |
| **13L** | 5.743E-01 | 3.342E+02 | 3.232E-02 | 3.080E-01 | 2.222E-01 | 3.067E-04 | 3.619E+03 | 1.753E-04 | 2.090E+03 | 9.854E-02 | 1.183E+06 | 3.537E+03 | 2.025E+01 |
| **14L** | 7.224E-01 | 7.252E+00 | 1.886E-02 | 4.827E-01 | 5.519E-01 | 6.255E-04 | 2.593E+03 | 4.538E-04 | 1.926E+03 | 4.498E-03 | 1.577E+04 | 2.369E+03 | 3.280E+00 |
| **15L** | 6.397E-01 | 2.004E+02 | 3.634E-02 | 3.790E-01 | 2.829E-01 | 1.725E-04 | 6.144E+03 | 1.098E-04 | 3.970E+03 | 3.414E-02 | 1.204E+06 | 6.067E+03 | 1.249E+01 |
| **16L** | 6.572E-01 | 1.226E+02 | 4.487E-02 | 4.011E-01 | 3.238E-01 | 1.996E-04 | 5.167E+03 | 1.291E-04 | 3.450E+03 | 2.924E-02 | 5.199E+05 | 5.128E+03 | 9.538E+00 |
| **17L** | 5.885E-01 | 8.401E+03 | 4.942E-02 | 3.253E-01 | 1.216E-01 | 3.090E-04 | 3.385E+03 | 1.798E-04 | 2.020E+03 | 2.968E+00 | 2.409E+07 | 3.350E+03 | 6.758E+01 |
| **18L** | 6.405E-01 | 1.680E+02 | 2.621E-02 | 3.806E-01 | 2.651E-01 | 2.845E-04 | 4.060E+03 | 1.833E-04 | 2.649E+03 | 4.870E-02 | 6.007E+05 | 3.930E+03 | 1.423E+01 |
| **19L** | 6.675E-01 | 2.763E+01 | 2.347E-02 | 4.121E-01 | 4.108E-01 | 6.490E-04 | 2.175E+03 | 4.306E-04 | 1.520E+03 | 1.994E-02 | 4.392E+04 | 2.006E+03 | 5.925E+00 |
| **20L** | 5.982E-01 | 1.157E+02 | 3.491E-02 | 3.337E-01 | 2.872E-01 | 1.985E-04 | 5.324E+03 | 1.195E-04 | 3.175E+03 | 2.244E-02 | 6.183E+05 | 5.257E+03 | 1.212E+01 |
| **21L** | 6.237E-01 | 2.334E+03 | 3.007E-02 | 3.618E-01 | 2.246E-01 | 3.166E-04 | 3.536E+03 | 1.941E-04 | 2.259E+03 | 8.974E-01 | 6.123E+06 | 3.445E+03 | 1.983E+01 |
| **22L** | 6.858E-01 | 1.710E+02 | 2.687E-02 | 4.347E-01 | 3.869E-01 | 2.569E-04 | 4.441E+03 | 1.752E-04 | 3.121E+03 | 5.107E-02 | 5.797E+05 | 4.289E+03 | 6.681E+00 |
| **24L** | 6.834E-01 | 1.780E+01 | 2.231E-02 | 4.318E-01 | 4.527E-01 | 3.379E-04 | 4.523E+03 | 2.326E-04 | 3.156E+03 | 5.663E-03 | 7.224E+04 | 4.289E+03 | 4.880E+00 |
| **25L** | 6.790E-01 | 1.421E+01 | 2.455E-02 | 4.264E-01 | 4.565E-01 | 3.336E-04 | 3.822E+03 | 2.319E-04 | 2.615E+03 | 4.391E-03 | 5.103E+04 | 3.661E+03 | 4.798E+00 |
| **26L** | 6.771E-01 | 1.190E+01 | 2.668E-02 | 4.244E-01 | 4.764E-01 | 2.690E-04 | 4.211E+03 | 1.814E-04 | 2.892E+03 | 3.396E-03 | 4.523E+04 | 4.095E+03 | 4.406E+00 |
| **27L** | 7.128E-01 | 7.969E+00 | 2.068E-02 | 4.698E-01 | 5.411E-01 | 5.225E-04 | 2.949E+03 | 3.807E-04 | 2.131E+03 | 3.603E-03 | 2.225E+04 | 2.753E+03 | 3.415E+00 |
| **28L** | 6.511E-01 | 3.118E+03 | 2.022E-02 | 3.926E-01 | 2.269E-01 | 3.850E-04 | 3.409E+03 | 2.385E-04 | 2.357E+03 | 1.902E+00 | 5.214E+06 | 3.187E+03 | 1.942E+01 |
| **29L** | 7.831E-01 | 2.808E+00 | 6.010E-02 | 5.703E-01 | 7.027E-01 | 1.570E-04 | 6.521E+03 | 1.229E-04 | 5.124E+03 | 4.364E-04 | 1.829E+04 | 6.463E+03 | 2.018E+00 |
| **30L** | 6.793E-01 | 1.205E+01 | 2.267E-02 | 4.268E-01 | 4.718E-01 | 4.355E-04 | 3.329E+03 | 3.022E-04 | 2.276E+03 | 4.739E-03 | 3.774E+04 | 3.164E+03 | 4.492E+00 |
| **31L** | 6.280E-01 | 4.776E+01 | 1.956E-02 | 3.658E-01 | 3.459E-01 | 3.984E-04 | 3.382E+03 | 2.456E-04 | 2.209E+03 | 2.055E-02 | 1.265E+05 | 3.170E+03 | 8.356E+00 |
| **32L** | 7.774E-01 | 3.099E+00 | 4.200E-02 | 5.604E-01 | 6.855E-01 | 1.700E-04 | 6.089E+03 | 1.323E-04 | 4.732E+03 | 5.221E-04 | 1.890E+04 | 6.032E+03 | 2.124E+00 |
| **33L** | 7.244E-01 | 6.915E+00 | 1.751E-02 | 4.854E-01 | 5.651E-01 | 6.215E-04 | 2.882E+03 | 4.574E-04 | 2.121E+03 | 3.695E-03 | 1.849E+04 | 2.616E+03 | 3.130E+00 |
| **34L** | 6.639E-01 | 2.908E+01 | 2.762E-02 | 4.079E-01 | 4.055E-01 | 1.090E-03 | 1.375E+03 | 7.304E-04 | 9.464E+02 | 3.216E-02 | 3.039E+04 | 1.267E+03 | 6.081E+00 |
| **35L** | 6.693E-01 | 8.615E+02 | 2.215E-02 | 4.142E-01 | 3.290E-01 | 4.497E-03 | 8.800E+02 | 2.869E-03 | 6.531E+02 | 2.233E+01 | 6.739E+04 | 6.927E+02 | 9.237E+00 |
| **36L** | 6.814E-01 | 1.347E+01 | 2.108E-02 | 4.294E-01 | 4.680E-01 | 3.541E-04 | 3.550E+03 | 2.410E-04 | 2.458E+03 | 5.133E-03 | 4.149E+04 | 3.375E+03 | 4.564E+00 |
| **37L** | 6.992E-01 | 8.805E+00 | 1.911E-02 | 4.520E-01 | 5.157E-01 | 4.425E-04 | 3.450E+03 | 3.169E-04 | 2.459E+03 | 3.675E-03 | 2.746E+04 | 3.215E+03 | 3.760E+00 |
| **38L** | 6.859E-01 | 1.327E+03 | 1.778E-02 | 4.350E-01 | 3.659E-01 | 4.538E-04 | 3.202E+03 | 3.025E-04 | 2.274E+03 | 1.279E+00 | 1.410E+06 | 2.955E+03 | 7.468E+00 |
| **39L** | 6.878E-01 | 2.538E+03 | 1.819E-02 | 4.373E-01 | 3.203E-01 | 3.197E-04 | 4.039E+03 | 2.151E-04 | 2.856E+03 | 1.686E+00 | 3.896E+06 | 3.797E+03 | 9.750E+00 |
| **40L** | 7.609E-01 | 7.321E+00 | 1.969E-02 | 5.367E-01 | 5.890E-01 | 3.737E-04 | 3.567E+03 | 2.778E-04 | 2.834E+03 | 3.133E-03 | 2.012E+04 | 3.347E+03 | 2.881E+00 |
| **41L** | 8.014E-01 | 3.640E+00 | 1.489E-02 | 5.978E-01 | 6.847E-01 | 3.472E-04 | 5.550E+03 | 2.782E-04 | 4.515E+03 | 1.208E-03 | 1.858E+04 | 5.152E+03 | 2.132E+00 |
| **42L** | 6.433E-01 | 2.225E+01 | 2.313E-02 | 3.835E-01 | 3.970E-01 | 5.767E-04 | 2.396E+03 | 3.660E-04 | 1.580E+03 | 1.610E-02 | 4.180E+04 | 2.250E+03 | 6.345E+00 |
| **43L** | 7.095E-01 | 8.564E+00 | 1.885E-02 | 4.654E-01 | 5.266E-01 | 5.546E-04 | 3.080E+03 | 3.902E-04 | 2.223E+03 | 5.860E-03 | 2.295E+04 | 2.845E+03 | 3.606E+00 |
| **44L** | 8.031E-01 | 3.899E+00 | 4.532E-02 | 6.028E-01 | 6.593E-01 | 1.712E-04 | 6.118E+03 | 1.362E-04 | 4.996E+03 | 7.114E-04 | 2.182E+04 | 6.035E+03 | 2.295E+00 |
| **45L** | 7.223E-01 | 1.113E+01 | 1.491E-02 | 4.824E-01 | 5.249E-01 | 7.876E-04 | 2.809E+03 | 5.717E-04 | 2.131E+03 | 1.104E-02 | 1.864E+04 | 2.434E+03 | 3.629E+00 |
| **46L** | 6.641E-01 | 1.610E+01 | 3.414E-02 | 4.078E-01 | 4.281E-01 | 2.222E-04 | 4.802E+03 | 1.450E-04 | 3.253E+03 | 4.158E-03 | 6.500E+04 | 4.727E+03 | 5.453E+00 |
| **47L** | 5.556E-01 | 1.776E+04 | 4.181E-02 | 2.915E-01 | 5.727E-02 | 2.346E-04 | 4.469E+03 | 1.295E-04 | 2.515E+03 | 4.777E+00 | 6.655E+07 | 4.420E+03 | 3.049E+02 |
| **48L** | 5.422E-01 | 3.173E+04 | 5.669E-02 | 2.784E-01 | 5.279E-02 | 2.024E-04 | 5.068E+03 | 1.091E-04 | 2.778E+03 | 6.691E+00 | 1.507E+08 | 5.036E+03 | 3.588E+02 |
| **49L** | 5.870E-01 | 6.619E+02 | 3.615E-02 | 3.215E-01 | 2.535E-01 | 1.002E-02 | 3.670E+02 | 5.966E-03 | 2.285E+02 | 1.420E+01 | 4.198E+04 | 2.941E+02 | 1.557E+01 |
| **50L** | 7.283E-01 | 9.864E+00 | 3.123E-02 | 4.907E-01 | 5.205E-01 | 2.091E-04 | 5.290E+03 | 1.530E-04 | 3.902E+03 | 2.108E-03 | 4.834E+04 | 5.189E+03 | 3.689E+00 |
| **51L** | 6.571E-01 | 6.047E+03 | 2.137E-02 | 3.995E-01 | 2.169E-01 | 3.966E-04 | 3.339E+03 | 2.584E-04 | 2.208E+03 | 5.202E+00 | 7.744E+06 | 3.152E+03 | 2.127E+01 |
| **52L** | 6.877E-01 | 7.981E+03 | 2.137E-02 | 4.376E-01 | 2.682E-01 | 4.325E-04 | 3.018E+03 | 2.887E-04 | 2.122E+03 | 6.188E+00 | 1.038E+07 | 2.840E+03 | 1.390E+01 |
| **53L** | 5.833E-01 | 2.888E+04 | 3.783E-02 | 3.196E-01 | 8.126E-02 | 1.672E-04 | 6.275E+03 | 9.881E-05 | 3.655E+03 | 4.332E+00 | 1.933E+08 | 6.208E+03 | 1.514E+02 |
| **55L** | 5.996E-01 | 4.296E+03 | 3.285E-02 | 3.345E-01 | 1.418E-01 | 1.869E-04 | 5.659E+03 | 1.108E-04 | 3.445E+03 | 9.194E-01 | 2.027E+07 | 5.582E+03 | 4.976E+01 |
| **57L** | 6.474E-01 | 1.360E+02 | 3.144E-02 | 3.880E-01 | 3.169E-01 | 2.089E-04 | 5.128E+03 | 1.328E-04 | 3.402E+03 | 3.217E-02 | 5.811E+05 | 5.041E+03 | 9.959E+00 |
| **58L** | 6.084E-01 | 6.552E+02 | 2.537E-02 | 3.443E-01 | 2.329E-01 | 2.946E-04 | 3.898E+03 | 1.747E-04 | 2.459E+03 | 3.005E-01 | 1.505E+06 | 3.768E+03 | 1.844E+01 |
| **59L** | 8.765E-01 | 1.832E+00 | 3.374E-02 | 7.263E-01 | 8.221E-01 | 1.561E-04 | 6.683E+03 | 1.368E-04 | 5.864E+03 | 2.835E-04 | 1.228E+04 | 6.597E+03 | 1.476E+00 |
| **60L** | 6.096E-01 | 3.940E+02 | 2.971E-02 | 3.455E-01 | 2.626E-01 | 2.603E-04 | 4.361E+03 | 1.598E-04 | 2.689E+03 | 1.074E-01 | 1.472E+06 | 4.252E+03 | 1.450E+01 |
| **61L** | 6.499E-01 | 3.691E+01 | 1.651E-02 | 3.913E-01 | 3.904E-01 | 5.793E-04 | 3.129E+03 | 3.613E-04 | 2.124E+03 | 5.903E-02 | 7.100E+04 | 2.841E+03 | 6.562E+00 |
| **62L** | 8.691E-01 | 1.817E+00 | 3.262E-02 | 7.118E-01 | 8.195E-01 | 1.718E-04 | 6.190E+03 | 1.478E-04 | 5.427E+03 | 3.185E-04 | 1.107E+04 | 6.083E+03 | 1.484E+00 |
| **63L** | 6.292E-01 | 1.569E+03 | 3.126E-02 | 3.671E-01 | 2.081E-01 | 3.457E-04 | 3.290E+03 | 2.132E-04 | 2.154E+03 | 6.618E-01 | 3.792E+06 | 3.178E+03 | 2.310E+01 |
| **65L** | 6.143E-01 | 6.184E+01 | 2.172E-02 | 3.512E-01 | 3.392E-01 | 1.570E-04 | 7.136E+03 | 9.513E-05 | 4.492E+03 | 1.075E-02 | 3.759E+05 | 6.926E+03 | 8.692E+00 |
| **66L** | 6.538E-01 | 5.345E+02 | 2.494E-02 | 3.954E-01 | 2.762E-01 | 1.499E-04 | 7.506E+03 | 9.517E-05 | 5.169E+03 | 8.238E-02 | 3.496E+06 | 7.238E+03 | 1.311E+01 |
| **70L** | 6.375E-01 | 2.116E+03 | 2.201E-02 | 3.767E-01 | 2.349E-01 | 1.807E-04 | 6.292E+03 | 1.137E-04 | 4.123E+03 | 5.507E-01 | 8.239E+06 | 6.109E+03 | 1.812E+01 |
| **71L** | 6.166E-01 | 1.355E+03 | 2.986E-02 | 3.536E-01 | 2.235E-01 | 1.596E-04 | 6.640E+03 | 9.714E-05 | 4.154E+03 | 2.524E-01 | 7.329E+06 | 6.548E+03 | 2.003E+01 |
| **72L** | 6.539E-01 | 7.343E+02 | 2.620E-02 | 3.955E-01 | 2.299E-01 | 2.325E-04 | 4.870E+03 | 1.481E-04 | 3.308E+03 | 2.199E-01 | 2.516E+06 | 4.709E+03 | 1.892E+01 |
| **73L** | 6.569E-01 | 1.271E+02 | 2.090E-02 | 3.990E-01 | 3.109E-01 | 2.651E-04 | 4.614E+03 | 1.778E-04 | 2.932E+03 | 2.706E-02 | 7.019E+05 | 4.422E+03 | 1.035E+01 |
| **74L** | 8.164E-01 | 2.514E+00 | 3.939E-02 | 6.217E-01 | 7.346E-01 | 1.731E-04 | 6.139E+03 | 1.394E-04 | 5.103E+03 | 4.589E-04 | 1.443E+04 | 6.021E+03 | 1.849E+00 |
| **75L** | 6.570E-01 | 5.419E+02 | 1.872E-02 | 3.997E-01 | 3.347E-01 | 2.703E-04 | 4.677E+03 | 1.708E-04 | 3.189E+03 | 2.940E-01 | 1.133E+06 | 4.447E+03 | 8.927E+00 |
