# Supplementary material for: A simple model for glioma grading based on texture analysis applied to conventional brain MRI
Source: PLoS One. 2020 May 15;15(5):e0228972. doi: 10.1371/journal.pone.0228972 (PMC7228074; doi:10.1371/journal.pone.0228972)
Supplement: S6 Table — (DOCX) [file pone.0228972.s006.docx]

**T_2_^1^**

| **HGG** | ***F*_szm.sze_** | ***F*_szm.lze_** | ***F*_szm.glnu_** | ***F*_szm.zsnu_** | ***F*_szm.z.perc_** | ***F*_szm.lgze_** | ***F*_szm.hgze_** | ***F*_szm.szlge_** | ***F*_szm.szhge_** | ***F*_szm.lzlge_** | ***F*_szm.lzhge_** | ***F*_szm.gl.var_** | ***F*_szm.zs.var_** |
| --- | --- | --- | --- | --- | --- | --- | --- | --- | --- | --- | --- | --- | --- |
| **1H** | 7.409E-01 | 5.974E+00 | 7.966E-03 | 5.080E-01 | 5.847E-01 | 4.263E-05 | 3.084E+04 | 3.295E-05 | 2.247E+04 | 2.068E-04 | 2.081E+05 | 2.957E+04 | 2.925E+00 |
| **2H** | 7.576E-01 | 7.708E+01 | 9.068E-03 | 5.322E-01 | 4.765E-01 | 8.403E-05 | 1.606E+04 | 6.301E-05 | 1.219E+04 | 3.540E-03 | 1.947E+06 | 1.491E+04 | 4.404E+00 |
| **4H** | 8.620E-01 | 1.860E+00 | 1.350E-02 | 6.990E-01 | 8.111E-01 | 6.859E-05 | 1.673E+04 | 5.977E-05 | 1.430E+04 | 1.197E-04 | 3.276E+04 | 1.616E+04 | 1.517E+00 |
| **7H** | 7.168E-01 | 8.428E+00 | 2.287E-02 | 4.755E-01 | 5.381E-01 | 8.693E-05 | 1.226E+04 | 6.331E-05 | 8.700E+03 | 6.633E-04 | 1.102E+05 | 1.208E+04 | 3.453E+00 |
| **8H** | 7.970E-01 | 2.026E+02 | 9.753E-03 | 5.923E-01 | 4.630E-01 | 4.832E-05 | 2.625E+04 | 4.061E-05 | 2.004E+04 | 5.414E-03 | 7.625E+06 | 2.518E+04 | 4.664E+00 |
| **9H** | 7.719E-01 | 1.044E+02 | 6.988E-03 | 5.529E-01 | 5.299E-01 | 5.027E-05 | 2.782E+04 | 3.944E-05 | 2.149E+04 | 2.091E-03 | 5.798E+06 | 2.613E+04 | 3.561E+00 |
| **12H** | 7.213E-01 | 1.638E+01 | 9.019E-03 | 4.811E-01 | 5.105E-01 | 6.401E-05 | 2.434E+04 | 5.085E-05 | 1.700E+04 | 6.057E-04 | 4.925E+05 | 2.336E+04 | 3.838E+00 |
| **13H** | 6.945E-01 | 1.047E+01 | 1.545E-02 | 4.461E-01 | 5.066E-01 | 9.683E-05 | 1.184E+04 | 6.932E-05 | 8.048E+03 | 8.549E-04 | 1.421E+05 | 1.150E+04 | 3.896E+00 |
| **14H** | 8.377E-01 | 2.356E+00 | 2.679E-02 | 6.569E-01 | 7.613E-01 | 7.150E-05 | 1.500E+04 | 6.178E-05 | 1.221E+04 | 1.507E-04 | 3.866E+04 | 1.473E+04 | 1.722E+00 |
| **16H** | 7.778E-01 | 3.538E+00 | 1.651E-02 | 5.613E-01 | 6.642E-01 | 6.943E-05 | 1.567E+04 | 5.472E-05 | 1.210E+04 | 2.358E-04 | 5.644E+04 | 1.535E+04 | 2.265E+00 |
| **17H** | 6.647E-01 | 1.017E+01 | 2.606E-02 | 4.095E-01 | 4.812E-01 | 1.203E-04 | 1.100E+04 | 8.891E-05 | 7.281E+03 | 9.530E-04 | 1.157E+05 | 1.086E+04 | 4.317E+00 |
| **19H** | 8.933E-01 | 1.600E+00 | 1.695E-02 | 7.577E-01 | 8.535E-01 | 6.468E-05 | 1.684E+04 | 5.760E-05 | 1.508E+04 | 1.049E-04 | 2.669E+04 | 1.646E+04 | 1.369E+00 |
| **20H** | 7.030E-01 | 9.608E+00 | 1.350E-02 | 4.571E-01 | 5.165E-01 | 5.699E-05 | 1.933E+04 | 4.064E-05 | 1.340E+04 | 5.039E-04 | 2.046E+05 | 1.887E+04 | 3.747E+00 |
| **21H** | 7.608E-01 | 1.173E+01 | 1.613E-02 | 5.365E-01 | 5.696E-01 | 5.568E-05 | 1.937E+04 | 4.296E-05 | 1.458E+04 | 5.520E-04 | 2.590E+05 | 1.903E+04 | 3.082E+00 |
| **22H** | 7.109E-01 | 1.730E+01 | 1.696E-02 | 4.673E-01 | 4.810E-01 | 4.885E-05 | 2.191E+04 | 3.563E-05 | 1.534E+04 | 7.505E-04 | 4.101E+05 | 2.161E+04 | 4.322E+00 |
| **23H** | 7.008E-01 | 1.045E+01 | 3.710E-02 | 4.544E-01 | 5.075E-01 | 7.661E-05 | 1.341E+04 | 5.399E-05 | 9.370E+03 | 7.559E-04 | 1.465E+05 | 1.332E+04 | 3.881E+00 |
| **24H** | 7.610E-01 | 7.480E+00 | 1.024E-02 | 5.368E-01 | 5.877E-01 | 7.975E-05 | 1.719E+04 | 6.281E-05 | 1.282E+04 | 4.908E-04 | 1.485E+05 | 1.628E+04 | 2.895E+00 |
| **25H** | 8.307E-01 | 2.608E+00 | 1.286E-02 | 6.456E-01 | 7.420E-01 | 6.733E-04 | 1.410E+04 | 6.490E-04 | 1.168E+04 | 9.661E-04 | 3.680E+04 | 1.342E+04 | 1.815E+00 |
| **26H** | 7.680E-01 | 5.973E+00 | 1.252E-02 | 5.469E-01 | 6.181E-01 | 1.097E-04 | 1.109E+04 | 8.295E-05 | 8.649E+03 | 8.019E-04 | 5.339E+04 | 1.058E+04 | 2.617E+00 |
| **27H** | 8.089E-01 | 3.781E+00 | 1.360E-02 | 6.102E-01 | 6.830E-01 | 5.756E-05 | 2.211E+04 | 4.771E-05 | 1.755E+04 | 1.815E-04 | 9.339E+04 | 2.123E+04 | 2.142E+00 |
| **28H** | 8.230E-01 | 2.836E+00 | 5.024E-02 | 6.327E-01 | 7.389E-01 | 7.121E-05 | 1.432E+04 | 5.940E-05 | 1.160E+04 | 1.866E-04 | 4.410E+04 | 1.422E+04 | 1.829E+00 |
| **29H** | 7.906E-01 | 5.558E+00 | 1.219E-02 | 5.811E-01 | 6.522E-01 | 1.327E-04 | 1.058E+04 | 1.085E-04 | 8.363E+03 | 6.421E-04 | 5.686E+04 | 1.001E+04 | 2.351E+00 |
| **30H** | 8.669E-01 | 1.751E+00 | 1.632E-02 | 7.074E-01 | 8.234E-01 | 7.775E-05 | 1.551E+04 | 6.846E-05 | 1.340E+04 | 1.294E-04 | 2.731E+04 | 1.489E+04 | 1.472E+00 |
| **31H** | 7.479E-01 | 6.716E+00 | 2.232E-02 | 5.180E-01 | 5.777E-01 | 6.785E-05 | 1.545E+04 | 5.085E-05 | 1.159E+04 | 4.654E-04 | 9.961E+04 | 1.527E+04 | 2.996E+00 |
| **32H** | 8.121E-01 | 3.080E+00 | 1.448E-02 | 6.151E-01 | 7.061E-01 | 4.768E-05 | 2.245E+04 | 3.936E-05 | 1.790E+04 | 1.315E-04 | 7.780E+04 | 2.206E+04 | 2.004E+00 |
| **33H** | 8.069E-01 | 3.698E+00 | 1.069E-02 | 6.069E-01 | 6.868E-01 | 3.506E-05 | 3.286E+04 | 2.905E-05 | 2.605E+04 | 1.118E-04 | 1.345E+05 | 3.195E+04 | 2.119E+00 |
| **34H** | 8.175E-01 | 3.303E+00 | 1.226E-02 | 6.242E-01 | 7.049E-01 | 9.605E-05 | 1.286E+04 | 7.887E-05 | 1.049E+04 | 3.165E-04 | 4.277E+04 | 1.223E+04 | 2.011E+00 |
| **35H** | 8.106E-01 | 4.653E+00 | 1.220E-02 | 6.135E-01 | 6.708E-01 | 8.864E-05 | 1.434E+04 | 7.081E-05 | 1.191E+04 | 4.411E-04 | 5.579E+04 | 1.348E+04 | 2.222E+00 |
| **36H** | 7.251E-01 | 1.290E+01 | 1.511E-02 | 4.863E-01 | 5.217E-01 | 9.952E-05 | 1.184E+04 | 7.489E-05 | 8.425E+03 | 1.011E-03 | 1.806E+05 | 1.148E+04 | 3.674E+00 |
| **37H** | 7.990E-01 | 3.374E+00 | 1.569E-02 | 5.938E-01 | 6.932E-01 | 1.112E-04 | 1.549E+04 | 8.771E-05 | 1.220E+04 | 3.016E-04 | 5.582E+04 | 1.510E+04 | 2.080E+00 |
| **38H** | 8.837E-01 | 1.683E+00 | 1.400E-02 | 7.389E-01 | 8.416E-01 | 4.880E-05 | 2.356E+04 | 4.397E-05 | 2.038E+04 | 7.510E-05 | 4.340E+04 | 2.276E+04 | 1.409E+00 |
| **39H** | 7.967E-01 | 4.086E+00 | 1.264E-02 | 5.908E-01 | 6.661E-01 | 6.656E-05 | 1.780E+04 | 5.298E-05 | 1.430E+04 | 2.882E-04 | 6.795E+04 | 1.719E+04 | 2.253E+00 |
| **40H** | 7.581E-01 | 5.776E+00 | 1.090E-02 | 5.324E-01 | 6.063E-01 | 1.713E-04 | 1.212E+04 | 1.398E-04 | 9.151E+03 | 7.284E-04 | 6.775E+04 | 1.127E+04 | 2.720E+00 |
| **41H** | 7.881E-01 | 4.505E+00 | 1.829E-02 | 5.774E-01 | 6.519E-01 | 8.920E-05 | 1.223E+04 | 7.147E-05 | 9.479E+03 | 3.432E-04 | 6.367E+04 | 1.197E+04 | 2.352E+00 |
| **42H** | 8.095E-01 | 3.162E+00 | 1.048E-02 | 6.106E-01 | 7.059E-01 | 8.230E-05 | 1.617E+04 | 6.843E-05 | 1.292E+04 | 2.240E-04 | 5.598E+04 | 1.541E+04 | 2.006E+00 |
| **43H** | 8.164E-01 | 3.018E+00 | 8.095E-03 | 6.216E-01 | 7.183E-01 | 5.027E-05 | 2.514E+04 | 4.215E-05 | 2.009E+04 | 1.287E-04 | 8.497E+04 | 2.389E+04 | 1.937E+00 |
| **44H** | 7.810E-01 | 5.089E+00 | 1.202E-02 | 5.663E-01 | 6.388E-01 | 7.984E-05 | 1.512E+04 | 6.326E-05 | 1.168E+04 | 3.592E-04 | 8.535E+04 | 1.447E+04 | 2.449E+00 |
| **45H** | 7.742E-01 | 4.582E+00 | 1.054E-02 | 5.560E-01 | 6.388E-01 | 6.927E-05 | 1.846E+04 | 5.559E-05 | 1.393E+04 | 2.471E-04 | 9.987E+04 | 1.769E+04 | 2.450E+00 |
| **47H** | 7.748E-01 | 5.387E+00 | 1.108E-02 | 5.572E-01 | 6.267E-01 | 5.292E-05 | 2.194E+04 | 4.160E-05 | 1.678E+04 | 2.764E-04 | 1.220E+05 | 2.125E+04 | 2.545E+00 |
| **49H** | 7.755E-01 | 5.050E+00 | 1.965E-02 | 5.582E-01 | 6.297E-01 | 4.818E-05 | 2.173E+04 | 3.784E-05 | 1.666E+04 | 2.233E-04 | 1.179E+05 | 2.148E+04 | 2.521E+00 |
| **50H** | 8.082E-01 | 4.023E+00 | 1.841E-02 | 6.091E-01 | 6.831E-01 | 1.129E-04 | 1.122E+04 | 8.982E-05 | 9.397E+03 | 4.901E-04 | 3.704E+04 | 1.042E+04 | 2.142E+00 |
| **51H** | 7.798E-01 | 7.478E+00 | 1.385E-02 | 5.646E-01 | 6.217E-01 | 4.958E-05 | 2.195E+04 | 3.898E-05 | 1.699E+04 | 2.953E-04 | 2.039E+05 | 2.149E+04 | 2.587E+00 |
| **52H** | 7.169E-01 | 8.951E+00 | 1.156E-02 | 4.756E-01 | 5.310E-01 | 6.686E-05 | 1.740E+04 | 4.822E-05 | 1.241E+04 | 6.183E-04 | 1.547E+05 | 1.676E+04 | 3.546E+00 |
| **53H** | 8.222E-01 | 4.731E+00 | 1.191E-02 | 6.322E-01 | 6.855E-01 | 5.922E-05 | 1.939E+04 | 4.962E-05 | 1.567E+04 | 2.230E-04 | 1.110E+05 | 1.879E+04 | 2.128E+00 |
| **54H** | 7.752E-01 | 7.282E+00 | 8.934E-03 | 5.576E-01 | 6.204E-01 | 8.139E-05 | 1.704E+04 | 6.378E-05 | 1.315E+04 | 4.843E-04 | 1.365E+05 | 1.597E+04 | 2.598E+00 |
| **55H** | 7.782E-01 | 4.165E+00 | 1.444E-02 | 5.621E-01 | 6.487E-01 | 1.013E-04 | 1.199E+04 | 7.926E-05 | 9.469E+03 | 4.307E-04 | 4.568E+04 | 1.156E+04 | 2.375E+00 |
| **56H** | 8.259E-01 | 3.396E+00 | 1.518E-02 | 6.377E-01 | 7.162E-01 | 3.693E-05 | 3.063E+04 | 3.133E-05 | 2.467E+04 | 1.019E-04 | 1.227E+05 | 2.991E+04 | 1.948E+00 |
| **57H** | 7.145E-01 | 8.452E+00 | 1.125E-02 | 4.723E-01 | 5.409E-01 | 7.593E-05 | 1.663E+04 | 5.634E-05 | 1.172E+04 | 5.366E-04 | 1.665E+05 | 1.602E+04 | 3.417E+00 |
| **58H** | 8.059E-01 | 3.587E+00 | 9.201E-03 | 6.050E-01 | 6.901E-01 | 5.144E-05 | 2.338E+04 | 4.162E-05 | 1.869E+04 | 1.713E-04 | 9.103E+04 | 2.245E+04 | 2.099E+00 |
| **59H** | 8.196E-01 | 3.321E+00 | 7.628E-03 | 6.265E-01 | 7.250E-01 | 6.962E-04 | 9.553E+03 | 5.273E-04 | 7.811E+03 | 5.503E-03 | 2.537E+04 | 8.029E+03 | 1.902E+00 |
| **60H** | 7.247E-01 | 2.390E+01 | 1.332E-02 | 4.858E-01 | 4.887E-01 | 4.403E-05 | 2.484E+04 | 3.246E-05 | 1.785E+04 | 8.312E-04 | 7.325E+05 | 2.433E+04 | 4.188E+00 |
| **61H** | 7.542E-01 | 5.299E+00 | 1.163E-02 | 5.267E-01 | 6.090E-01 | 2.528E-04 | 1.586E+04 | 2.183E-04 | 1.170E+04 | 6.866E-04 | 9.030E+04 | 1.511E+04 | 2.696E+00 |
| **62H** | 7.881E-01 | 7.235E+00 | 6.794E-03 | 5.775E-01 | 6.212E-01 | 4.119E-05 | 3.137E+04 | 3.300E-05 | 2.414E+04 | 2.096E-04 | 3.014E+05 | 2.972E+04 | 2.591E+00 |
| **64H** | 6.874E-01 | 1.209E+01 | 4.710E-02 | 4.374E-01 | 4.478E-01 | 1.074E-04 | 9.594E+03 | 7.598E-05 | 6.456E+03 | 1.231E-03 | 1.195E+05 | 9.523E+03 | 4.983E+00 |
| **65H** | 7.911E-01 | 3.612E+00 | 1.187E-02 | 5.816E-01 | 6.779E-01 | 5.808E-05 | 1.949E+04 | 4.672E-05 | 1.518E+04 | 1.953E-04 | 7.565E+04 | 1.892E+04 | 2.176E+00 |
| **66H** | 8.837E-01 | 1.561E+00 | 1.548E-02 | 7.379E-01 | 8.528E-01 | 5.364E-05 | 2.155E+04 | 4.791E-05 | 1.881E+04 | 8.173E-05 | 3.445E+04 | 2.075E+04 | 1.370E+00 |
| **67H** | 7.490E-01 | 5.678E+00 | 1.569E-02 | 5.194E-01 | 5.960E-01 | 9.918E-05 | 1.590E+04 | 7.469E-05 | 1.178E+04 | 4.657E-04 | 9.475E+04 | 1.538E+04 | 2.814E+00 |
| **68H** | 8.162E-01 | 1.718E+01 | 1.142E-02 | 6.224E-01 | 5.937E-01 | 4.119E-05 | 3.085E+04 | 3.452E-05 | 2.440E+04 | 4.114E-04 | 7.562E+05 | 2.942E+04 | 2.837E+00 |
| **69H** | 8.368E-01 | 2.355E+00 | 1.411E-02 | 6.556E-01 | 7.588E-01 | 6.654E-05 | 1.715E+04 | 5.691E-05 | 1.417E+04 | 1.412E-04 | 4.336E+04 | 1.669E+04 | 1.735E+00 |
| **70H** | 7.713E-01 | 4.851E+00 | 1.205E-02 | 5.518E-01 | 6.307E-01 | 7.811E-05 | 1.520E+04 | 6.043E-05 | 1.180E+04 | 3.751E-04 | 7.081E+04 | 1.456E+04 | 2.513E+00 |
| **71H** | 7.879E-01 | 3.230E+00 | 1.583E-02 | 5.764E-01 | 6.864E-01 | 5.172E-05 | 2.078E+04 | 4.146E-05 | 1.613E+04 | 1.567E-04 | 7.042E+04 | 2.042E+04 | 2.121E+00 |
| **72H** | 8.084E-01 | 5.659E+00 | 1.227E-02 | 6.097E-01 | 6.572E-01 | 4.943E-05 | 2.263E+04 | 4.112E-05 | 1.778E+04 | 2.127E-04 | 1.610E+05 | 2.208E+04 | 2.315E+00 |
| **73H** | 6.982E-01 | 1.459E+01 | 2.197E-02 | 4.508E-01 | 4.971E-01 | 8.990E-05 | 1.184E+04 | 6.310E-05 | 8.261E+03 | 1.527E-03 | 1.487E+05 | 1.168E+04 | 4.047E+00 |
| **74H** | 7.063E-01 | 1.051E+01 | 2.141E-02 | 4.612E-01 | 4.999E-01 | 7.591E-05 | 1.395E+04 | 5.396E-05 | 9.846E+03 | 7.793E-04 | 1.457E+05 | 1.376E+04 | 4.000E+00 |
| **75H** | 7.802E-01 | 4.065E+00 | 1.147E-02 | 5.649E-01 | 6.596E-01 | 5.405E-05 | 2.105E+04 | 4.314E-05 | 1.607E+04 | 1.872E-04 | 9.922E+04 | 2.041E+04 | 2.297E+00 |
| **76H** | 8.232E-01 | 2.841E+00 | 7.797E-03 | 6.329E-01 | 7.291E-01 | 5.466E-05 | 2.392E+04 | 4.577E-05 | 1.950E+04 | 1.440E-04 | 6.974E+04 | 2.253E+04 | 1.880E+00 |
| **78H** | 7.816E-01 | 7.348E+00 | 1.314E-02 | 5.673E-01 | 6.315E-01 | 3.759E-04 | 5.276E+03 | 2.869E-04 | 4.347E+03 | 3.774E-03 | 2.226E+04 | 4.445E+03 | 2.508E+00 |
| **79H** | 7.931E-01 | 1.106E+01 | 2.539E-02 | 5.858E-01 | 5.949E-01 | 7.742E-05 | 1.400E+04 | 6.214E-05 | 1.106E+04 | 7.326E-04 | 1.699E+05 | 1.376E+04 | 2.825E+00 |
| **80H** | 7.758E-01 | 1.714E+02 | 9.114E-03 | 5.596E-01 | 3.897E-01 | 4.919E-05 | 2.506E+04 | 3.897E-05 | 1.898E+04 | 4.700E-03 | 6.392E+06 | 2.398E+04 | 6.585E+00 |
| **81H** | 8.007E-01 | 1.261E+02 | 1.073E-02 | 5.993E-01 | 4.478E-01 | 3.763E-05 | 3.815E+04 | 3.127E-05 | 2.922E+04 | 2.255E-03 | 7.192E+06 | 3.554E+04 | 4.987E+00 |
| **82H** | 7.118E-01 | 1.018E+01 | 1.742E-02 | 4.684E-01 | 5.206E-01 | 8.513E-05 | 1.296E+04 | 6.058E-05 | 9.294E+03 | 9.328E-04 | 1.171E+05 | 1.266E+04 | 3.689E+00 |
| **83H** | 8.916E-01 | 1.786E+00 | 1.153E-02 | 7.554E-01 | 8.376E-01 | 4.994E-05 | 2.482E+04 | 4.485E-05 | 2.190E+04 | 8.286E-05 | 4.801E+04 | 2.360E+04 | 1.424E+00 |
| **84H** | 7.898E-01 | 4.078E+00 | 9.185E-03 | 5.798E-01 | 6.625E-01 | 1.607E-04 | 2.001E+04 | 1.362E-04 | 1.570E+04 | 4.647E-04 | 8.088E+04 | 1.862E+04 | 2.278E+00 |
| **85H** | 7.541E-01 | 4.305E+00 | 1.771E-02 | 5.265E-01 | 6.277E-01 | 7.632E-05 | 1.433E+04 | 5.873E-05 | 1.067E+04 | 3.150E-04 | 6.184E+04 | 1.403E+04 | 2.536E+00 |
| **86H** | 7.674E-01 | 5.993E+00 | 1.020E-02 | 5.460E-01 | 6.151E-01 | 5.716E-05 | 2.092E+04 | 4.430E-05 | 1.587E+04 | 3.116E-04 | 1.380E+05 | 2.006E+04 | 2.643E+00 |
| **87H** | 7.760E-01 | 3.594E+01 | 1.308E-02 | 5.596E-01 | 5.355E-01 | 8.536E-05 | 1.744E+04 | 7.209E-05 | 1.309E+04 | 1.769E-03 | 7.525E+05 | 1.672E+04 | 3.487E+00 |
| **88H** | 7.756E-01 | 4.680E+00 | 1.321E-02 | 5.581E-01 | 6.393E-01 | 5.403E-05 | 2.135E+04 | 4.264E-05 | 1.638E+04 | 2.274E-04 | 1.067E+05 | 2.077E+04 | 2.446E+00 |
| **89H** | 8.301E-01 | 3.300E+00 | 8.979E-03 | 6.447E-01 | 7.292E-01 | 5.505E-05 | 2.330E+04 | 4.686E-05 | 1.897E+04 | 1.469E-04 | 9.037E+04 | 2.219E+04 | 1.880E+00 |
| **90H** | 7.525E-01 | 8.770E+00 | 1.551E-02 | 5.244E-01 | 5.966E-01 | 7.932E-05 | 1.402E+04 | 6.075E-05 | 1.044E+04 | 7.607E-04 | 1.103E+05 | 1.369E+04 | 2.809E+00 |
| **91H** | 7.083E-01 | 4.923E+01 | 1.999E-02 | 4.641E-01 | 3.839E-01 | 8.843E-05 | 1.212E+04 | 6.220E-05 | 8.655E+03 | 4.345E-03 | 5.824E+05 | 1.191E+04 | 6.786E+00 |
| **92H** | 8.103E-01 | 4.359E+00 | 1.227E-02 | 6.125E-01 | 6.836E-01 | 9.695E-05 | 1.302E+04 | 7.852E-05 | 1.057E+04 | 4.841E-04 | 5.020E+04 | 1.229E+04 | 2.140E+00 |
| **93H** | 7.830E-01 | 2.474E+01 | 9.430E-03 | 5.703E-01 | 5.311E-01 | 3.139E-05 | 3.778E+04 | 2.537E-05 | 2.884E+04 | 5.413E-04 | 1.161E+06 | 3.657E+04 | 3.545E+00 |
| **94H** | 7.865E-01 | 4.403E+00 | 1.390E-02 | 5.748E-01 | 6.556E-01 | 1.012E-04 | 1.278E+04 | 7.854E-05 | 1.026E+04 | 4.903E-04 | 4.752E+04 | 1.187E+04 | 2.326E+00 |
| **95H** | 7.753E-01 | 9.971E+00 | 7.484E-03 | 5.581E-01 | 5.983E-01 | 6.163E-05 | 2.381E+04 | 5.010E-05 | 1.800E+04 | 3.925E-04 | 3.052E+05 | 2.237E+04 | 2.793E+00 |
| **96H** | 7.929E-01 | 4.332E+00 | 1.121E-02 | 5.846E-01 | 6.628E-01 | 1.124E-04 | 1.242E+04 | 8.896E-05 | 9.938E+03 | 5.158E-04 | 4.836E+04 | 1.138E+04 | 2.275E+00 |
| **97H** | 8.031E-01 | 1.696E+01 | 1.168E-02 | 6.030E-01 | 5.784E-01 | 5.333E-05 | 2.144E+04 | 4.368E-05 | 1.688E+04 | 6.096E-04 | 4.927E+05 | 2.078E+04 | 2.989E+00 |
| **98H** | 8.369E-01 | 2.436E+00 | 1.513E-02 | 6.559E-01 | 7.564E-01 | 6.602E-05 | 1.660E+04 | 5.575E-05 | 1.381E+04 | 1.565E-04 | 4.080E+04 | 1.620E+04 | 1.746E+00 |
| **99H** | 8.081E-01 | 7.827E+00 | 2.330E-02 | 6.100E-01 | 6.288E-01 | 1.250E-04 | 8.897E+03 | 1.025E-04 | 7.171E+03 | 8.467E-04 | 7.450E+04 | 8.674E+03 | 2.528E+00 |
| **101H** | 7.802E-01 | 6.907E+00 | 1.394E-02 | 5.655E-01 | 6.101E-01 | 1.044E-04 | 1.667E+04 | 9.013E-05 | 1.260E+04 | 4.380E-04 | 1.267E+05 | 1.595E+04 | 2.686E+00 |
| **102H** | 8.062E-01 | 3.921E+00 | 2.411E-02 | 6.064E-01 | 6.753E-01 | 4.430E-05 | 2.334E+04 | 3.565E-05 | 1.891E+04 | 1.741E-04 | 8.972E+04 | 2.313E+04 | 2.192E+00 |
| **103H** | 8.275E-01 | 2.939E+00 | 1.478E-02 | 6.402E-01 | 7.316E-01 | 5.560E-05 | 1.960E+04 | 4.671E-05 | 1.603E+04 | 1.524E-04 | 6.037E+04 | 1.922E+04 | 1.867E+00 |
| **104H** | 7.558E-01 | 5.211E+00 | 1.390E-02 | 5.291E-01 | 6.111E-01 | 8.199E-05 | 1.399E+04 | 6.214E-05 | 1.062E+04 | 4.252E-04 | 7.023E+04 | 1.349E+04 | 2.677E+00 |
| **105H** | 7.062E-01 | 2.720E+01 | 1.810E-02 | 4.615E-01 | 4.558E-01 | 7.374E-05 | 1.476E+04 | 5.238E-05 | 1.041E+04 | 2.318E-03 | 3.288E+05 | 1.445E+04 | 4.812E+00 |
| **108H** | 8.011E-01 | 4.144E+00 | 1.474E-02 | 5.977E-01 | 6.688E-01 | 6.472E-05 | 1.712E+04 | 5.326E-05 | 1.340E+04 | 2.243E-04 | 8.217E+04 | 1.673E+04 | 2.234E+00 |
| **109H** | 7.662E-01 | 4.247E+00 | 1.051E-02 | 5.440E-01 | 6.378E-01 | 6.479E-05 | 2.054E+04 | 5.087E-05 | 1.565E+04 | 2.447E-04 | 8.894E+04 | 1.969E+04 | 2.458E+00 |
| **110H** | 7.344E-01 | 5.924E+00 | 1.283E-02 | 4.990E-01 | 5.775E-01 | 4.933E-05 | 2.366E+04 | 3.725E-05 | 1.722E+04 | 2.620E-04 | 1.434E+05 | 2.295E+04 | 2.998E+00 |
| **111H** | 7.573E-01 | 3.447E+00 | 1.605E-02 | 5.312E-01 | 6.575E-01 | 6.839E-05 | 1.599E+04 | 5.196E-05 | 1.210E+04 | 2.267E-04 | 5.672E+04 | 1.565E+04 | 2.311E+00 |
| **112H** | 6.826E-01 | 1.370E+01 | 1.926E-02 | 4.309E-01 | 4.681E-01 | 8.651E-05 | 1.257E+04 | 5.974E-05 | 8.550E+03 | 1.137E-03 | 1.731E+05 | 1.232E+04 | 4.563E+00 |
| **113H** | 7.216E-01 | 1.433E+01 | 2.099E-02 | 4.815E-01 | 5.155E-01 | 1.080E-04 | 1.019E+04 | 7.767E-05 | 7.417E+03 | 1.472E-03 | 1.451E+05 | 9.930E+03 | 3.763E+00 |
| **114H** | 8.073E-01 | 3.280E+00 | 1.178E-02 | 6.072E-01 | 6.993E-01 | 8.256E-05 | 1.458E+04 | 6.611E-05 | 1.196E+04 | 2.772E-04 | 4.407E+04 | 1.390E+04 | 2.044E+00 |
| **115H** | 6.993E-01 | 1.556E+01 | 2.278E-02 | 4.525E-01 | 4.692E-01 | 4.453E-05 | 2.343E+04 | 3.190E-05 | 1.611E+04 | 6.378E-04 | 3.840E+05 | 2.321E+04 | 4.541E+00 |
| **116H** | 7.837E-01 | 2.208E+01 | 1.401E-02 | 5.724E-01 | 5.028E-01 | 7.342E-05 | 1.589E+04 | 6.016E-05 | 1.199E+04 | 1.111E-03 | 4.492E+05 | 1.537E+04 | 3.954E+00 |
| **117H** | 7.926E-01 | 3.221E+00 | 1.000E-02 | 5.839E-01 | 6.876E-01 | 4.202E-05 | 2.819E+04 | 3.386E-05 | 2.206E+04 | 1.276E-04 | 9.390E+04 | 2.719E+04 | 2.114E+00 |
| **118H** | 8.240E-01 | 2.557E+00 | 8.707E-03 | 6.339E-01 | 7.407E-01 | 5.509E-05 | 2.263E+04 | 4.523E-05 | 1.873E+04 | 1.435E-04 | 5.641E+04 | 2.155E+04 | 1.822E+00 |
| **119H** | 7.748E-01 | 5.024E+00 | 1.009E-02 | 5.569E-01 | 6.429E-01 | 4.486E-05 | 2.610E+04 | 3.519E-05 | 2.005E+04 | 2.044E-04 | 1.359E+05 | 2.503E+04 | 2.419E+00 |
| **120H** | 7.909E-01 | 3.458E+00 | 9.983E-03 | 5.813E-01 | 6.820E-01 | 6.248E-05 | 2.048E+04 | 5.068E-05 | 1.595E+04 | 1.931E-04 | 7.595E+04 | 1.933E+04 | 2.149E+00 |
| **121H** | 6.140E-01 | 5.593E+01 | 3.135E-02 | 3.509E-01 | 3.141E-01 | 8.028E-05 | 1.481E+04 | 5.495E-05 | 8.693E+03 | 3.373E-03 | 9.349E+05 | 1.446E+04 | 1.013E+01 |
| **122H** | 6.742E-01 | 2.653E+02 | 1.654E-02 | 4.201E-01 | 2.866E-01 | 2.464E-05 | 4.438E+04 | 1.731E-05 | 2.899E+04 | 5.032E-03 | 1.403E+07 | 4.365E+04 | 1.217E+01 |
| **123H** | 7.611E-01 | 1.627E+02 | 1.113E-02 | 5.373E-01 | 4.128E-01 | 3.431E-05 | 3.328E+04 | 2.705E-05 | 2.459E+04 | 3.574E-03 | 7.480E+06 | 3.245E+04 | 5.868E+00 |
| **124H** | 6.919E-01 | 1.605E+01 | 1.330E-02 | 4.426E-01 | 4.666E-01 | 1.131E-04 | 1.163E+04 | 7.688E-05 | 8.072E+03 | 1.739E-03 | 1.619E+05 | 1.112E+04 | 4.593E+00 |
| **125H** | 7.157E-01 | 9.537E+00 | 1.402E-02 | 4.735E-01 | 5.246E-01 | 5.926E-05 | 1.894E+04 | 4.339E-05 | 1.332E+04 | 4.800E-04 | 2.050E+05 | 1.849E+04 | 3.633E+00 |
| **126H** | 7.939E-01 | 2.387E+01 | 8.051E-03 | 5.870E-01 | 5.857E-01 | 4.088E-05 | 3.079E+04 | 3.365E-05 | 2.355E+04 | 5.577E-04 | 1.071E+06 | 2.940E+04 | 2.915E+00 |
| **127H** | 8.153E-01 | 3.588E+00 | 3.336E-02 | 6.227E-01 | 6.800E-01 | 3.332E-05 | 3.039E+04 | 2.700E-05 | 2.495E+04 | 1.216E-04 | 1.067E+05 | 3.025E+04 | 2.160E+00 |
| **128H** | 7.282E-01 | 6.260E+00 | 2.046E-02 | 4.906E-01 | 5.673E-01 | 8.115E-05 | 1.324E+04 | 6.017E-05 | 9.531E+03 | 4.761E-04 | 8.539E+04 | 1.302E+04 | 3.106E+00 |
| **129H** | 6.699E-01 | 1.433E+02 | 8.882E-03 | 4.149E-01 | 3.263E-01 | 8.996E-05 | 1.496E+04 | 6.205E-05 | 9.744E+03 | 7.299E-03 | 3.155E+06 | 1.399E+04 | 9.390E+00 |
| **130H** | 7.801E-01 | 3.956E+03 | 7.915E-03 | 5.651E-01 | 2.723E-01 | 3.702E-05 | 3.319E+04 | 3.010E-05 | 2.490E+04 | 7.912E-02 | 1.982E+08 | 3.183E+04 | 1.349E+01 |
| **131H** | 7.622E-01 | 6.971E+00 | 1.224E-02 | 5.385E-01 | 5.933E-01 | 3.954E-05 | 2.798E+04 | 3.112E-05 | 2.081E+04 | 2.301E-04 | 2.200E+05 | 2.737E+04 | 2.841E+00 |
| **132H** | 6.825E-01 | 4.268E+01 | 2.631E-02 | 4.309E-01 | 4.166E-01 | 9.093E-05 | 1.150E+04 | 6.312E-05 | 7.754E+03 | 3.409E-03 | 5.375E+05 | 1.137E+04 | 5.760E+00 |
| **133H** | 9.421E-01 | 1.278E+00 | 1.460E-02 | 8.592E-01 | 9.216E-01 | 7.172E-05 | 1.576E+04 | 6.782E-05 | 1.480E+04 | 8.985E-05 | 2.053E+04 | 1.525E+04 | 1.174E+00 |
| **134H** | 7.973E-01 | 3.095E+00 | 1.419E-02 | 5.913E-01 | 6.949E-01 | 5.653E-05 | 1.963E+04 | 4.608E-05 | 1.540E+04 | 1.611E-04 | 6.364E+04 | 1.916E+04 | 2.069E+00 |
| **135H** | 7.366E-01 | 5.188E+00 | 2.777E-02 | 5.020E-01 | 5.890E-01 | 1.069E-04 | 9.784E+03 | 7.961E-05 | 7.146E+03 | 5.294E-04 | 5.256E+04 | 9.668E+03 | 2.880E+00 |
| **136H** | 6.910E-01 | 1.583E+01 | 1.341E-02 | 4.417E-01 | 4.745E-01 | 5.188E-05 | 2.142E+04 | 3.690E-05 | 1.449E+04 | 6.890E-04 | 3.796E+05 | 2.094E+04 | 4.441E+00 |
| **137H** | 7.934E-01 | 3.436E+00 | 9.907E-03 | 5.851E-01 | 6.858E-01 | 5.040E-05 | 2.519E+04 | 4.096E-05 | 1.973E+04 | 1.508E-04 | 9.482E+04 | 2.417E+04 | 2.126E+00 |
| **138H** | 7.534E-01 | 5.925E+00 | 6.443E-03 | 5.256E-01 | 6.063E-01 | 7.141E-05 | 2.115E+04 | 5.489E-05 | 1.554E+04 | 3.615E-04 | 1.502E+05 | 1.934E+04 | 2.720E+00 |
| **139H** | 7.798E-01 | 3.427E+00 | 3.653E-02 | 5.644E-01 | 6.764E-01 | 1.097E-04 | 9.402E+03 | 8.496E-05 | 7.373E+03 | 3.990E-04 | 3.053E+04 | 9.316E+03 | 2.182E+00 |
| **140H** | 7.463E-01 | 1.191E+01 | 1.634E-02 | 5.157E-01 | 5.415E-01 | 9.986E-05 | 1.150E+04 | 7.294E-05 | 8.819E+03 | 1.455E-03 | 1.045E+05 | 1.108E+04 | 3.410E+00 |
| **141H** | 8.492E-01 | 2.568E+00 | 8.812E-03 | 6.780E-01 | 7.590E-01 | 3.529E-05 | 3.571E+04 | 3.106E-05 | 2.944E+04 | 7.218E-05 | 1.098E+05 | 3.417E+04 | 1.735E+00 |
| **142H** | 6.362E-01 | 6.677E+02 | 2.065E-02 | 3.747E-01 | 2.126E-01 | 9.385E-05 | 1.148E+04 | 6.074E-05 | 7.228E+03 | 6.244E-02 | 7.247E+06 | 1.128E+04 | 2.212E+01 |
| **143H** | 6.728E-01 | 1.560E+01 | 1.366E-02 | 4.190E-01 | 4.518E-01 | 8.150E-05 | 1.414E+04 | 5.612E-05 | 9.386E+03 | 1.201E-03 | 2.248E+05 | 1.369E+04 | 4.899E+00 |
| **144H** | 8.364E-01 | 2.224E+00 | 2.710E-02 | 6.547E-01 | 7.669E-01 | 7.612E-05 | 1.507E+04 | 6.395E-05 | 1.260E+04 | 1.657E-04 | 3.336E+04 | 1.444E+04 | 1.698E+00 |
| **145H** | 7.614E-01 | 4.133E+00 | 1.373E-02 | 5.371E-01 | 6.387E-01 | 3.999E-05 | 2.755E+04 | 3.109E-05 | 2.069E+04 | 1.531E-04 | 1.188E+05 | 2.699E+04 | 2.450E+00 |
| **146H** | 7.881E-01 | 3.437E+00 | 2.098E-02 | 5.768E-01 | 6.802E-01 | 6.109E-05 | 1.713E+04 | 4.806E-05 | 1.352E+04 | 2.174E-04 | 5.706E+04 | 1.693E+04 | 2.159E+00 |
| **147H** | 7.428E-01 | 7.837E+00 | 1.237E-02 | 5.109E-01 | 5.572E-01 | 4.700E-05 | 2.468E+04 | 3.638E-05 | 1.775E+04 | 2.891E-04 | 2.265E+05 | 2.398E+04 | 3.220E+00 |
| **148H** | 7.748E-01 | 5.428E+00 | 1.180E-02 | 5.571E-01 | 6.287E-01 | 5.896E-05 | 1.965E+04 | 4.655E-05 | 1.505E+04 | 2.640E-04 | 1.253E+05 | 1.903E+04 | 2.529E+00 |
| **149H** | 7.869E-01 | 3.426E+00 | 8.944E-03 | 5.750E-01 | 6.797E-01 | 5.002E-05 | 2.395E+04 | 3.993E-05 | 1.864E+04 | 1.587E-04 | 8.596E+04 | 2.290E+04 | 2.164E+00 |
| **150H** | 7.946E-01 | 3.394E+00 | 1.085E-02 | 5.870E-01 | 6.864E-01 | 1.304E-04 | 1.176E+04 | 1.054E-04 | 9.181E+03 | 3.860E-04 | 4.493E+04 | 1.099E+04 | 2.121E+00 |
| **151H** | 7.870E-01 | 3.459E+00 | 7.784E-03 | 5.752E-01 | 6.765E-01 | 5.837E-05 | 2.460E+04 | 4.660E-05 | 1.903E+04 | 1.839E-04 | 9.376E+04 | 2.317E+04 | 2.184E+00 |
| **152H** | 7.492E-01 | 7.395E+00 | 8.055E-03 | 5.197E-01 | 5.848E-01 | 4.359E-05 | 2.911E+04 | 3.374E-05 | 2.132E+04 | 2.474E-04 | 2.610E+05 | 2.777E+04 | 2.924E+00 |
| **153H** | 8.032E-01 | 6.414E+00 | 1.154E-02 | 6.016E-01 | 6.456E-01 | 1.894E-03 | 3.358E+03 | 1.326E-03 | 2.840E+03 | 5.560E-02 | 1.525E+04 | 2.548E+03 | 2.399E+00 |
| **154H** | 7.629E-01 | 5.711E+00 | 9.983E-03 | 5.395E-01 | 6.141E-01 | 1.526E-04 | 1.253E+04 | 1.213E-04 | 9.681E+03 | 7.057E-04 | 6.202E+04 | 1.125E+04 | 2.651E+00 |
| **155H** | 7.958E-01 | 3.190E+00 | 1.534E-02 | 5.889E-01 | 6.956E-01 | 6.066E-05 | 1.979E+04 | 4.953E-05 | 1.534E+04 | 1.775E-04 | 6.582E+04 | 1.882E+04 | 2.066E+00 |
| **156H** | 7.081E-01 | 8.363E+00 | 2.564E-02 | 4.642E-01 | 5.340E-01 | 9.662E-05 | 1.088E+04 | 6.907E-05 | 7.663E+03 | 7.557E-04 | 9.509E+04 | 1.073E+04 | 3.505E+00 |
| **157H** | 6.975E-01 | 1.090E+01 | 1.755E-02 | 4.499E-01 | 4.980E-01 | 2.035E-04 | 6.874E+03 | 1.518E-04 | 4.611E+03 | 1.493E-03 | 9.211E+04 | 6.552E+03 | 4.030E+00 |
| **158H** | 8.300E-01 | 3.001E+00 | 8.419E-03 | 6.446E-01 | 7.304E-01 | 6.478E-05 | 2.359E+04 | 5.503E-05 | 1.924E+04 | 1.609E-04 | 8.360E+04 | 2.222E+04 | 1.874E+00 |
| **160H** | 7.488E-01 | 6.167E+00 | 1.192E-02 | 5.194E-01 | 6.010E-01 | 7.071E-05 | 1.688E+04 | 5.321E-05 | 1.255E+04 | 3.873E-04 | 1.131E+05 | 1.621E+04 | 2.768E+00 |
| **161H** | 6.981E-01 | 1.071E+01 | 2.144E-02 | 4.506E-01 | 4.999E-01 | 8.870E-05 | 1.224E+04 | 6.265E-05 | 8.513E+03 | 9.213E-04 | 1.290E+05 | 1.202E+04 | 4.000E+00 |
| **162H** | 7.880E-01 | 3.679E+00 | 8.371E-03 | 5.769E-01 | 6.708E-01 | 5.221E-05 | 2.370E+04 | 4.075E-05 | 1.885E+04 | 2.058E-04 | 8.185E+04 | 2.245E+04 | 2.222E+00 |
| **163H** | 7.793E-01 | 3.822E+00 | 1.378E-02 | 5.635E-01 | 6.608E-01 | 7.274E-05 | 1.637E+04 | 5.833E-05 | 1.262E+04 | 2.519E-04 | 6.376E+04 | 1.583E+04 | 2.289E+00 |
| **164H** | 8.681E-01 | 1.872E+00 | 9.692E-03 | 7.100E-01 | 8.152E-01 | 4.380E-05 | 3.557E+04 | 3.889E-05 | 3.024E+04 | 7.530E-05 | 7.140E+04 | 3.298E+04 | 1.503E+00 |
| **165H** | 6.977E-01 | 8.538E+00 | 1.387E-02 | 4.500E-01 | 5.147E-01 | 9.690E-05 | 1.199E+04 | 6.803E-05 | 8.360E+03 | 8.711E-04 | 9.438E+04 | 1.157E+04 | 3.773E+00 |
| **166H** | 7.897E-01 | 3.261E+00 | 1.881E-02 | 5.797E-01 | 6.807E-01 | 9.910E-05 | 1.142E+04 | 7.959E-05 | 8.906E+03 | 2.921E-04 | 4.003E+04 | 1.109E+04 | 2.154E+00 |
| **167H** | 7.118E-01 | 9.524E+00 | 1.821E-02 | 4.686E-01 | 5.248E-01 | 1.158E-04 | 9.761E+03 | 8.193E-05 | 7.020E+03 | 1.084E-03 | 8.976E+04 | 9.479E+03 | 3.630E+00 |
| **168H** | 7.968E-01 | 4.220E+00 | 6.453E-03 | 5.911E-01 | 6.614E-01 | 5.385E-05 | 2.826E+04 | 4.176E-05 | 2.268E+04 | 2.819E-04 | 1.100E+05 | 2.587E+04 | 2.285E+00 |
| **169H** | 8.001E-01 | 2.849E+00 | 1.438E-02 | 5.953E-01 | 7.120E-01 | 5.261E-05 | 2.119E+04 | 4.269E-05 | 1.679E+04 | 1.421E-04 | 6.222E+04 | 2.070E+04 | 1.971E+00 |
| **170H** | 7.423E-01 | 7.609E+00 | 2.406E-02 | 5.100E-01 | 5.638E-01 | 9.243E-05 | 1.144E+04 | 6.843E-05 | 8.519E+03 | 7.802E-04 | 7.747E+04 | 1.127E+04 | 3.144E+00 |
| **171H** | 7.984E-01 | 3.406E+00 | 1.248E-02 | 5.929E-01 | 6.912E-01 | 4.830E-05 | 2.278E+04 | 3.943E-05 | 1.775E+04 | 1.418E-04 | 9.067E+04 | 2.226E+04 | 2.092E+00 |
| **172H** | 7.654E-01 | 6.737E+00 | 1.017E-02 | 5.436E-01 | 5.896E-01 | 3.481E-05 | 3.700E+04 | 2.813E-05 | 2.743E+04 | 1.689E-04 | 3.004E+05 | 3.547E+04 | 2.876E+00 |
| **173H** | 7.271E-01 | 1.018E+01 | 7.150E-03 | 4.890E-01 | 5.358E-01 | 6.950E-05 | 2.078E+04 | 4.954E-05 | 1.517E+04 | 7.041E-04 | 2.681E+05 | 1.904E+04 | 3.482E+00 |
| **174H** | 7.506E-01 | 8.030E+00 | 9.360E-03 | 5.217E-01 | 5.847E-01 | 8.899E-05 | 1.564E+04 | 6.552E-05 | 1.187E+04 | 8.742E-04 | 1.001E+05 | 1.435E+04 | 2.925E+00 |
| **175H** | 7.174E-01 | 6.650E+01 | 9.474E-03 | 4.762E-01 | 4.442E-01 | 5.697E-05 | 2.218E+04 | 4.229E-05 | 1.553E+04 | 2.322E-03 | 1.996E+06 | 2.121E+04 | 5.068E+00 |
| **176H** | 7.983E-01 | 4.438E+00 | 6.124E-03 | 5.929E-01 | 6.750E-01 | 6.826E-05 | 2.377E+04 | 5.433E-05 | 1.877E+04 | 2.454E-04 | 1.434E+05 | 2.133E+04 | 2.195E+00 |
| **177H** | 7.653E-01 | 5.293E+00 | 1.189E-02 | 5.431E-01 | 6.179E-01 | 7.784E-05 | 1.534E+04 | 5.928E-05 | 1.187E+04 | 4.468E-04 | 7.130E+04 | 1.472E+04 | 2.618E+00 |
| **178H** | 7.088E-01 | 1.198E+01 | 1.485E-02 | 4.648E-01 | 5.066E-01 | 3.520E-05 | 3.261E+04 | 2.586E-05 | 2.256E+04 | 3.265E-04 | 4.602E+05 | 3.193E+04 | 3.896E+00 |
| **181H** | 7.595E-01 | 5.070E+00 | 1.121E-02 | 5.344E-01 | 6.206E-01 | 7.299E-05 | 1.650E+04 | 5.492E-05 | 1.257E+04 | 3.712E-04 | 8.698E+04 | 1.576E+04 | 2.595E+00 |
| **182H** | 6.596E-01 | 3.361E+02 | 2.627E-02 | 4.032E-01 | 3.255E-01 | 3.364E-05 | 3.027E+04 | 2.261E-05 | 1.962E+04 | 9.697E-03 | 1.166E+07 | 3.015E+04 | 9.439E+00 |
| **183H** | 6.866E-01 | 5.560E+00 | 4.640E-02 | 4.400E-01 | 5.319E-01 | 7.480E-05 | 1.374E+04 | 5.236E-05 | 9.316E+03 | 4.034E-04 | 7.743E+04 | 1.362E+04 | 3.526E+00 |
| **184H** | 7.287E-01 | 8.827E+00 | 1.394E-02 | 4.912E-01 | 5.483E-01 | 9.688E-05 | 1.262E+04 | 6.925E-05 | 9.465E+03 | 9.920E-04 | 8.681E+04 | 1.193E+04 | 3.326E+00 |
| **185H** | 7.485E-01 | 3.727E+00 | 3.294E-02 | 5.207E-01 | 6.471E-01 | 1.016E-04 | 1.027E+04 | 7.528E-05 | 7.763E+03 | 3.897E-04 | 3.708E+04 | 1.014E+04 | 2.385E+00 |
| **186H** | 7.259E-01 | 1.459E+01 | 1.345E-02 | 4.875E-01 | 4.930E-01 | 3.217E-05 | 3.636E+04 | 2.456E-05 | 2.559E+04 | 3.530E-04 | 6.265E+05 | 3.548E+04 | 4.114E+00 |
| **187H** | 7.266E-01 | 7.658E+00 | 1.448E-02 | 4.883E-01 | 5.508E-01 | 3.499E-05 | 3.359E+04 | 2.648E-05 | 2.387E+04 | 2.201E-04 | 2.850E+05 | 3.274E+04 | 3.296E+00 |
| **188H** | 8.897E-01 | 1.824E+00 | 1.497E-02 | 7.531E-01 | 8.307E-01 | 6.437E-05 | 1.852E+04 | 5.699E-05 | 1.663E+04 | 1.213E-04 | 3.152E+04 | 1.759E+04 | 1.447E+00 |
| **189H** | 7.135E-01 | 6.680E+00 | 1.686E-02 | 4.709E-01 | 5.536E-01 | 8.053E-05 | 1.501E+04 | 5.703E-05 | 1.084E+04 | 5.499E-04 | 1.003E+05 | 1.412E+04 | 3.262E+00 |
| **190H** | 7.737E-01 | 1.907E+01 | 7.551E-03 | 5.558E-01 | 5.560E-01 | 9.743E-05 | 1.590E+04 | 7.287E-05 | 1.239E+04 | 2.113E-03 | 2.536E+05 | 1.446E+04 | 3.234E+00 |
| **191H** | 6.958E-01 | 3.913E+01 | 1.250E-02 | 4.476E-01 | 4.315E-01 | 9.175E-05 | 1.390E+04 | 6.673E-05 | 9.375E+03 | 2.129E-03 | 7.577E+05 | 1.330E+04 | 5.370E+00 |
| **192H** | 8.690E-01 | 1.796E+00 | 8.205E-02 | 7.143E-01 | 8.167E-01 | 1.007E-04 | 9.981E+03 | 8.718E-05 | 8.707E+03 | 1.826E-04 | 1.776E+04 | 9.908E+03 | 1.490E+00 |
| **193H** | 7.307E-01 | 2.154E+03 | 1.103E-02 | 4.941E-01 | 3.017E-01 | 3.401E-05 | 3.278E+04 | 2.580E-05 | 2.316E+04 | 4.678E-02 | 9.942E+07 | 3.204E+04 | 1.099E+01 |
| **194H** | 6.864E-01 | 2.328E+01 | 1.966E-02 | 4.358E-01 | 4.570E-01 | 9.819E-05 | 1.122E+04 | 6.736E-05 | 7.727E+03 | 2.658E-03 | 2.147E+05 | 1.093E+04 | 4.787E+00 |
| **195H** | 7.537E-01 | 4.364E+00 | 1.012E-02 | 5.260E-01 | 6.291E-01 | 5.910E-05 | 1.997E+04 | 4.422E-05 | 1.519E+04 | 2.802E-04 | 7.985E+04 | 1.919E+04 | 2.525E+00 |
| **196H** | 6.639E-01 | 5.359E+01 | 1.342E-02 | 4.076E-01 | 3.689E-01 | 4.616E-05 | 2.408E+04 | 3.193E-05 | 1.554E+04 | 2.050E-03 | 1.451E+06 | 2.358E+04 | 7.347E+00 |
| **197H** | 6.969E-01 | 3.071E+01 | 1.083E-02 | 4.494E-01 | 4.584E-01 | 7.955E-05 | 1.523E+04 | 5.697E-05 | 1.036E+04 | 1.614E-03 | 6.607E+05 | 1.459E+04 | 4.758E+00 |
| **198H** | 7.408E-01 | 6.155E+00 | 1.001E-02 | 5.078E-01 | 5.796E-01 | 5.531E-05 | 2.237E+04 | 4.209E-05 | 1.621E+04 | 2.852E-04 | 1.596E+05 | 2.145E+04 | 2.976E+00 |
| **199H** | 8.201E-01 | 3.820E+00 | 3.914E-02 | 6.311E-01 | 6.803E-01 | 8.486E-05 | 1.200E+04 | 6.943E-05 | 9.859E+03 | 3.396E-04 | 4.364E+04 | 1.193E+04 | 2.158E+00 |
| **200H** | 8.397E-01 | 2.336E+00 | 9.673E-03 | 6.606E-01 | 7.627E-01 | 5.364E-05 | 2.306E+04 | 4.580E-05 | 1.921E+04 | 1.195E-04 | 5.514E+04 | 2.210E+04 | 1.718E+00 |
| **201H** | 7.687E-01 | 3.744E+02 | 7.435E-03 | 5.484E-01 | 3.766E-01 | 3.817E-05 | 3.529E+04 | 3.039E-05 | 2.616E+04 | 7.240E-03 | 1.952E+07 | 3.340E+04 | 7.051E+00 |
| **202H** | 8.070E-01 | 3.462E+00 | 8.930E-03 | 6.068E-01 | 6.940E-01 | 4.483E-05 | 2.627E+04 | 3.691E-05 | 2.087E+04 | 1.430E-04 | 9.503E+04 | 2.527E+04 | 2.076E+00 |
| **203H** | 6.665E-01 | 2.860E+01 | 1.591E-02 | 4.109E-01 | 4.153E-01 | 3.629E-05 | 2.991E+04 | 2.527E-05 | 1.937E+04 | 8.109E-04 | 1.030E+06 | 2.946E+04 | 5.799E+00 |
| **206H** | 7.804E-01 | 5.394E+00 | 7.902E-03 | 5.653E-01 | 6.454E-01 | 1.284E-04 | 1.789E+04 | 1.033E-04 | 1.367E+04 | 4.419E-04 | 1.357E+05 | 1.655E+04 | 2.400E+00 |
| **207H** | 6.615E-01 | 1.684E+01 | 2.453E-02 | 4.061E-01 | 4.292E-01 | 8.627E-05 | 1.212E+04 | 5.681E-05 | 8.068E+03 | 1.434E-03 | 2.066E+05 | 1.198E+04 | 5.427E+00 |
| **208H** | 6.696E-01 | 1.611E+02 | 1.516E-02 | 4.149E-01 | 3.624E-01 | 9.490E-05 | 1.208E+04 | 6.342E-05 | 8.111E+03 | 2.202E-02 | 1.287E+06 | 1.166E+04 | 7.613E+00 |
| **209H** | 6.591E-01 | 4.199E+02 | 1.742E-02 | 4.025E-01 | 2.767E-01 | 8.036E-05 | 1.350E+04 | 5.391E-05 | 8.762E+03 | 2.566E-02 | 6.992E+06 | 1.324E+04 | 1.306E+01 |
| **210H** | 6.423E-01 | 2.380E+01 | 2.053E-02 | 3.824E-01 | 4.006E-01 | 1.129E-04 | 9.731E+03 | 7.413E-05 | 6.145E+03 | 2.245E-03 | 2.698E+05 | 9.532E+03 | 6.231E+00 |

| **LGG** | ***F*_szm.sze_** | ***F*_szm.lze_** | ***F*_szm.glnu_** | ***F*_szm.zsnu_** | ***F*_szm.z.perc_** | ***F*_szm.lgze_** | ***F*_szm.hgze_** | ***F*_szm.szlge_** | ***F*_szm.szhge_** | ***F*_szm.lzlge_** | ***F*_szm.lzhge_** | ***F*_szm.gl.var_** | ***F*_szm.zs.var_** |
| --- | --- | --- | --- | --- | --- | --- | --- | --- | --- | --- | --- | --- | --- |
| **1L** | 7.376E-01 | 4.421E+01 | 1.300E-02 | 5.035E-01 | 4.848E-01 | 7.984E-04 | 1.105E+04 | 7.661E-04 | 7.962E+03 | 3.398E-03 | 8.055E+05 | 1.052E+04 | 4.254E+00 |
| **3L** | 7.540E-01 | 6.337E+00 | 1.071E-02 | 5.265E-01 | 6.012E-01 | 3.557E-05 | 3.124E+04 | 2.739E-05 | 2.304E+04 | 1.842E-04 | 2.396E+05 | 3.052E+04 | 2.766E+00 |
| **4L** | 6.613E-01 | 3.724E+01 | 1.815E-02 | 4.051E-01 | 3.815E-01 | 7.332E-05 | 1.464E+04 | 5.003E-05 | 9.425E+03 | 2.247E-03 | 6.299E+05 | 1.440E+04 | 6.870E+00 |
| **5L** | 7.134E-01 | 4.697E+01 | 1.315E-02 | 4.705E-01 | 4.287E-01 | 2.976E-05 | 3.616E+04 | 2.212E-05 | 2.491E+04 | 1.053E-03 | 2.124E+06 | 3.560E+04 | 5.440E+00 |
| **7L** | 7.172E-01 | 8.193E+00 | 9.281E-03 | 4.758E-01 | 5.422E-01 | 4.727E-05 | 2.488E+04 | 3.423E-05 | 1.763E+04 | 3.476E-04 | 2.240E+05 | 2.398E+04 | 3.401E+00 |
| **9L** | 7.333E-01 | 1.597E+01 | 1.000E-02 | 4.976E-01 | 5.125E-01 | 5.510E-05 | 2.399E+04 | 4.360E-05 | 1.685E+04 | 5.629E-04 | 4.937E+05 | 2.304E+04 | 3.807E+00 |
| **11L** | 6.187E-01 | 3.175E+01 | 3.645E-02 | 3.554E-01 | 3.428E-01 | 1.232E-04 | 8.387E+03 | 7.637E-05 | 5.196E+03 | 3.922E-03 | 2.614E+05 | 8.317E+03 | 8.507E+00 |
| **12L** | 7.842E-01 | 3.759E+00 | 2.357E-02 | 5.710E-01 | 6.686E-01 | 8.438E-05 | 1.246E+04 | 6.669E-05 | 9.713E+03 | 3.085E-04 | 4.807E+04 | 1.230E+04 | 2.235E+00 |
| **13L** | 6.309E-01 | 1.577E+02 | 2.261E-02 | 3.697E-01 | 3.389E-01 | 3.059E-05 | 3.361E+04 | 1.958E-05 | 2.096E+04 | 4.195E-03 | 5.961E+06 | 3.341E+04 | 8.707E+00 |
| **14L** | 6.551E-01 | 9.897E+01 | 2.071E-02 | 3.981E-01 | 3.280E-01 | 7.497E-05 | 1.413E+04 | 4.984E-05 | 9.141E+03 | 6.642E-03 | 1.496E+06 | 1.394E+04 | 9.295E+00 |
| **15L** | 7.351E-01 | 8.166E+00 | 1.057E-02 | 4.999E-01 | 5.590E-01 | 1.588E-04 | 1.014E+04 | 1.167E-04 | 7.496E+03 | 1.171E-03 | 1.013E+05 | 9.003E+03 | 3.199E+00 |
| **16L** | 7.023E-01 | 9.010E+00 | 1.600E-02 | 4.560E-01 | 5.142E-01 | 3.855E-05 | 2.735E+04 | 2.720E-05 | 1.918E+04 | 3.458E-04 | 2.455E+05 | 2.702E+04 | 3.782E+00 |
| **17L** | 6.528E-01 | 3.818E+01 | 1.514E-02 | 3.947E-01 | 3.838E-01 | 4.711E-05 | 2.258E+04 | 3.112E-05 | 1.460E+04 | 1.606E-03 | 9.326E+05 | 2.225E+04 | 6.787E+00 |
| **18L** | 6.662E-01 | 1.191E+02 | 1.744E-02 | 4.103E-01 | 3.339E-01 | 1.125E-04 | 1.006E+04 | 7.337E-05 | 6.884E+03 | 1.187E-02 | 1.215E+06 | 9.762E+03 | 8.969E+00 |
| **19L** | 6.708E-01 | 1.038E+03 | 1.553E-02 | 4.161E-01 | 2.630E-01 | 8.619E-05 | 1.309E+04 | 5.990E-05 | 8.527E+03 | 6.624E-02 | 1.637E+07 | 1.275E+04 | 1.446E+01 |
| **20L** | 6.451E-01 | 4.901E+01 | 1.051E-02 | 3.859E-01 | 3.684E-01 | 7.324E-05 | 1.653E+04 | 4.785E-05 | 1.060E+04 | 4.124E-03 | 7.231E+05 | 1.585E+04 | 7.366E+00 |
| **21L** | 7.176E-01 | 5.259E+02 | 1.334E-02 | 4.762E-01 | 3.403E-01 | 3.486E-05 | 3.057E+04 | 2.583E-05 | 2.128E+04 | 1.338E-02 | 2.072E+07 | 3.013E+04 | 8.634E+00 |
| **22L** | 5.840E-01 | 1.371E+04 | 2.682E-02 | 3.201E-01 | 1.160E-01 | 1.356E-04 | 8.017E+03 | 8.167E-05 | 4.581E+03 | 1.484E+00 | 1.276E+08 | 7.879E+03 | 7.432E+01 |
| **24L** | 6.159E-01 | 6.219E+02 | 1.931E-02 | 3.525E-01 | 2.281E-01 | 1.810E-04 | 6.486E+03 | 1.153E-04 | 3.950E+03 | 1.059E-01 | 3.826E+06 | 6.263E+03 | 1.921E+01 |
| **25L** | 6.827E-01 | 3.045E+01 | 1.766E-02 | 4.309E-01 | 4.165E-01 | 9.475E-05 | 1.174E+04 | 6.670E-05 | 7.883E+03 | 2.374E-03 | 4.050E+05 | 1.147E+04 | 5.763E+00 |
| **26L** | 5.880E-01 | 8.328E+01 | 2.388E-02 | 3.228E-01 | 2.856E-01 | 9.799E-05 | 1.083E+04 | 5.931E-05 | 6.221E+03 | 6.523E-03 | 1.090E+06 | 1.068E+04 | 1.226E+01 |
| **27L** | 6.257E-01 | 4.760E+02 | 2.069E-02 | 3.633E-01 | 2.384E-01 | 1.139E-04 | 9.699E+03 | 7.283E-05 | 6.022E+03 | 5.151E-02 | 4.495E+06 | 9.492E+03 | 1.760E+01 |
| **28L** | 7.711E-01 | 1.369E+03 | 8.401E-03 | 5.520E-01 | 3.672E-01 | 3.867E-05 | 3.638E+04 | 3.169E-05 | 2.699E+04 | 2.461E-02 | 7.641E+07 | 3.476E+04 | 7.418E+00 |
| **29L** | 8.806E-01 | 2.050E+00 | 3.500E-02 | 7.350E-01 | 8.108E-01 | 6.937E-05 | 1.488E+04 | 6.170E-05 | 1.301E+04 | 1.353E-04 | 3.167E+04 | 1.475E+04 | 1.518E+00 |
| **30L** | 6.118E-01 | 1.778E+02 | 2.079E-02 | 3.478E-01 | 2.689E-01 | 9.501E-05 | 1.125E+04 | 5.859E-05 | 6.859E+03 | 1.514E-02 | 2.162E+06 | 1.107E+04 | 1.383E+01 |
| **31L** | 6.405E-01 | 3.943E+02 | 2.024E-02 | 3.801E-01 | 2.446E-01 | 8.804E-05 | 1.229E+04 | 5.816E-05 | 7.721E+03 | 2.490E-02 | 6.359E+06 | 1.208E+04 | 1.672E+01 |
| **32L** | 8.067E-01 | 3.040E+00 | 1.924E-02 | 6.059E-01 | 7.075E-01 | 6.256E-05 | 1.689E+04 | 5.049E-05 | 1.365E+04 | 1.939E-04 | 4.962E+04 | 1.664E+04 | 1.996E+00 |
| **33L** | 6.678E-01 | 2.304E+01 | 1.625E-02 | 4.127E-01 | 4.341E-01 | 9.570E-05 | 1.168E+04 | 6.385E-05 | 7.833E+03 | 2.264E-03 | 2.510E+05 | 1.137E+04 | 5.306E+00 |
| **34L** | 6.224E-01 | 3.547E+02 | 2.046E-02 | 3.588E-01 | 2.303E-01 | 7.421E-05 | 1.433E+04 | 4.673E-05 | 8.855E+03 | 2.562E-02 | 5.008E+06 | 1.411E+04 | 1.885E+01 |
| **35L** | 6.614E-01 | 1.795E+03 | 1.650E-02 | 4.046E-01 | 1.980E-01 | 1.311E-04 | 1.374E+04 | 1.061E-04 | 8.765E+03 | 1.277E-01 | 2.629E+07 | 1.340E+04 | 2.552E+01 |
| **36L** | 6.132E-01 | 2.215E+03 | 2.418E-02 | 3.489E-01 | 1.501E-01 | 1.020E-04 | 1.036E+04 | 6.264E-05 | 6.366E+03 | 2.192E-01 | 2.324E+07 | 1.023E+04 | 4.440E+01 |
| **37L** | 6.377E-01 | 2.332E+02 | 1.970E-02 | 3.763E-01 | 2.429E-01 | 2.296E-04 | 9.018E+03 | 1.766E-04 | 5.620E+03 | 2.755E-02 | 2.179E+06 | 8.738E+03 | 1.694E+01 |
| **38L** | 7.351E-01 | 1.568E+02 | 6.549E-03 | 5.000E-01 | 4.716E-01 | 4.427E-05 | 3.053E+04 | 3.347E-05 | 2.185E+04 | 3.015E-03 | 8.427E+06 | 2.873E+04 | 4.496E+00 |
| **39L** | 7.366E-01 | 2.334E+02 | 8.684E-03 | 5.023E-01 | 3.998E-01 | 4.019E-05 | 3.269E+04 | 3.095E-05 | 2.315E+04 | 4.849E-03 | 1.164E+07 | 3.122E+04 | 6.255E+00 |
| **40L** | 6.556E-01 | 4.324E+01 | 1.579E-02 | 3.978E-01 | 3.652E-01 | 1.302E-04 | 9.458E+03 | 8.756E-05 | 6.181E+03 | 5.157E-03 | 4.098E+05 | 9.109E+03 | 7.498E+00 |
| **41L** | 6.951E-01 | 2.352E+01 | 1.741E-02 | 4.467E-01 | 4.556E-01 | 1.709E-04 | 7.198E+03 | 1.191E-04 | 5.103E+03 | 3.864E-03 | 1.529E+05 | 6.866E+03 | 4.817E+00 |
| **42L** | 6.179E-01 | 6.534E+01 | 1.896E-02 | 3.554E-01 | 3.301E-01 | 1.230E-04 | 8.965E+03 | 7.634E-05 | 5.501E+03 | 6.568E-03 | 7.034E+05 | 8.754E+03 | 9.177E+00 |
| **43L** | 6.833E-01 | 2.234E+01 | 1.502E-02 | 4.321E-01 | 4.402E-01 | 9.795E-05 | 1.164E+04 | 6.839E-05 | 7.812E+03 | 1.979E-03 | 3.031E+05 | 1.128E+04 | 5.159E+00 |
| **44L** | 8.582E-01 | 2.356E+00 | 2.533E-02 | 6.966E-01 | 7.704E-01 | 5.635E-05 | 1.875E+04 | 4.832E-05 | 1.618E+04 | 1.331E-04 | 4.318E+04 | 1.846E+04 | 1.682E+00 |
| **45L** | 7.032E-01 | 1.968E+02 | 1.236E-02 | 4.570E-01 | 3.673E-01 | 6.778E-04 | 1.015E+04 | 4.948E-04 | 6.781E+03 | 2.068E-02 | 2.313E+06 | 9.343E+03 | 7.411E+00 |
| **46L** | 7.667E-01 | 8.008E+00 | 1.454E-02 | 5.456E-01 | 5.821E-01 | 6.034E-05 | 1.878E+04 | 4.766E-05 | 1.404E+04 | 3.697E-04 | 1.881E+05 | 1.834E+04 | 2.950E+00 |
| **47L** | 6.472E-01 | 4.512E+02 | 2.029E-02 | 3.884E-01 | 2.677E-01 | 4.161E-05 | 2.481E+04 | 2.745E-05 | 1.577E+04 | 1.614E-02 | 1.265E+07 | 2.462E+04 | 1.395E+01 |
| **48L** | 6.509E-01 | 1.738E+01 | 1.675E-02 | 3.927E-01 | 4.341E-01 | 6.236E-05 | 1.713E+04 | 4.048E-05 | 1.120E+04 | 1.202E-03 | 2.640E+05 | 1.685E+04 | 5.306E+00 |
| **49L** | 6.388E-01 | 4.131E+01 | 1.496E-02 | 3.780E-01 | 3.522E-01 | 6.759E-05 | 1.623E+04 | 4.327E-05 | 1.040E+04 | 2.645E-03 | 6.780E+05 | 1.587E+04 | 8.060E+00 |
| **50L** | 7.544E-01 | 4.273E+00 | 1.503E-02 | 5.269E-01 | 6.307E-01 | 6.687E-05 | 1.668E+04 | 5.073E-05 | 1.261E+04 | 2.936E-04 | 6.749E+04 | 1.629E+04 | 2.513E+00 |
| **51L** | 7.018E-01 | 6.375E+03 | 1.359E-02 | 4.553E-01 | 1.941E-01 | 1.267E-04 | 1.083E+04 | 9.171E-05 | 7.620E+03 | 4.303E-01 | 1.036E+08 | 1.028E+04 | 2.654E+01 |
| **52L** | 7.559E-01 | 7.760E+02 | 9.307E-03 | 5.298E-01 | 4.037E-01 | 3.083E-05 | 4.009E+04 | 2.487E-05 | 2.895E+04 | 1.367E-02 | 4.414E+07 | 3.877E+04 | 6.136E+00 |
| **53L** | 5.963E-01 | 2.053E+02 | 1.962E-02 | 3.308E-01 | 2.597E-01 | 1.462E-04 | 8.549E+03 | 8.679E-05 | 5.075E+03 | 3.037E-02 | 1.464E+06 | 8.320E+03 | 1.483E+01 |
| **55L** | 6.263E-01 | 1.878E+02 | 2.100E-02 | 3.639E-01 | 2.750E-01 | 1.144E-04 | 9.491E+03 | 7.252E-05 | 5.893E+03 | 1.921E-02 | 1.919E+06 | 9.295E+03 | 1.322E+01 |
| **57L** | 6.550E-01 | 3.726E+01 | 1.940E-02 | 3.968E-01 | 3.682E-01 | 6.165E-05 | 1.710E+04 | 4.107E-05 | 1.101E+04 | 1.994E-03 | 7.158E+05 | 1.689E+04 | 7.375E+00 |
| **58L** | 6.191E-01 | 6.972E+03 | 1.709E-02 | 3.554E-01 | 1.329E-01 | 1.968E-04 | 6.874E+03 | 1.349E-04 | 4.109E+03 | 1.081E+00 | 4.615E+07 | 6.575E+03 | 5.660E+01 |
| **59L** | 6.962E-01 | 5.133E+00 | 3.556E-02 | 4.501E-01 | 5.741E-01 | 1.241E-04 | 8.335E+03 | 8.671E-05 | 5.798E+03 | 6.131E-04 | 4.432E+04 | 8.253E+03 | 3.030E+00 |
| **60L** | 6.191E-01 | 4.933E+02 | 1.577E-02 | 3.561E-01 | 2.531E-01 | 1.503E-04 | 8.462E+03 | 9.903E-05 | 5.110E+03 | 4.533E-02 | 5.564E+06 | 8.137E+03 | 1.561E+01 |
| **61L** | 6.192E-01 | 2.080E+02 | 1.542E-02 | 3.565E-01 | 2.825E-01 | 1.215E-04 | 9.831E+03 | 7.894E-05 | 5.945E+03 | 1.764E-02 | 2.725E+06 | 9.492E+03 | 1.253E+01 |
| **62L** | 8.005E-01 | 3.010E+00 | 2.952E-02 | 5.960E-01 | 7.074E-01 | 1.071E-04 | 9.782E+03 | 8.531E-05 | 7.869E+03 | 3.441E-04 | 2.752E+04 | 9.651E+03 | 1.996E+00 |
| **63L** | 7.060E-01 | 4.220E+01 | 1.255E-02 | 4.609E-01 | 4.192E-01 | 4.094E-05 | 2.768E+04 | 3.010E-05 | 1.891E+04 | 1.195E-03 | 1.525E+06 | 2.702E+04 | 5.689E+00 |
| **65L** | 6.552E-01 | 2.047E+03 | 1.720E-02 | 3.977E-01 | 3.028E-01 | 2.112E-04 | 6.263E+03 | 1.389E-04 | 4.178E+03 | 4.080E-01 | 1.033E+07 | 5.950E+03 | 1.091E+01 |
| **66L** | 6.557E-01 | 5.217E+02 | 1.411E-02 | 3.979E-01 | 2.788E-01 | 1.188E-04 | 1.044E+04 | 8.274E-05 | 6.592E+03 | 4.323E-02 | 6.370E+06 | 1.004E+04 | 1.287E+01 |
| **70L** | 6.537E-01 | 8.339E+02 | 1.271E-02 | 3.959E-01 | 2.609E-01 | 2.447E-04 | 1.013E+04 | 2.031E-04 | 6.360E+03 | 8.602E-02 | 8.425E+06 | 9.600E+03 | 1.469E+01 |
| **71L** | 6.862E-01 | 2.487E+01 | 1.140E-02 | 4.356E-01 | 4.419E-01 | 6.388E-05 | 1.817E+04 | 4.528E-05 | 1.214E+04 | 1.159E-03 | 5.794E+05 | 1.758E+04 | 5.121E+00 |
| **72L** | 7.217E-01 | 7.723E+01 | 1.082E-02 | 4.818E-01 | 4.261E-01 | 6.901E-05 | 1.746E+04 | 5.170E-05 | 1.218E+04 | 3.599E-03 | 1.709E+06 | 1.679E+04 | 5.509E+00 |
| **73L** | 7.146E-01 | 1.112E+01 | 1.520E-02 | 4.722E-01 | 5.203E-01 | 8.379E-05 | 1.398E+04 | 6.115E-05 | 9.918E+03 | 8.862E-04 | 1.474E+05 | 1.356E+04 | 3.693E+00 |
| **74L** | 6.959E-01 | 1.434E+01 | 5.690E-02 | 4.487E-01 | 4.577E-01 | 8.273E-05 | 1.226E+04 | 5.717E-05 | 8.620E+03 | 1.200E-03 | 1.718E+05 | 1.221E+04 | 4.772E+00 |
| **75L** | 6.256E-01 | 3.646E+04 | 1.692E-02 | 3.632E-01 | 1.344E-01 | 1.874E-04 | 6.634E+03 | 1.213E-04 | 4.113E+03 | 7.403E+00 | 1.801E+08 | 6.318E+03 | 5.532E+01 |
