# Supplementary material for: A simple model for glioma grading based on texture analysis applied to conventional brain MRI
Source: PLoS One. 2020 May 15;15(5):e0228972. doi: 10.1371/journal.pone.0228972 (PMC7228074; doi:10.1371/journal.pone.0228972)
Supplement: S7 Table — (DOCX) [file pone.0228972.s007.docx]

**T_1Gd_^2^**

| **HGG** | ***F*_szm.sze_** | ***F*_szm.lze_** | ***F*_szm.glnu_** | ***F*_szm.zsnu_** | ***F*_szm.z.perc_** | ***F*_szm.lgze_** | ***F*_szm.hgze_** | ***F*_szm.szlge_** | ***F*_szm.szhge_** | ***F*_szm.lzlge_** | ***F*_szm.lzhge_** | ***F*_szm.gl.var_** | ***F*_szm.zs.var_** |
| --- | --- | --- | --- | --- | --- | --- | --- | --- | --- | --- | --- | --- | --- |
| **1H** | 6.452E-01 | 1.088E+03 | 3.158E-02 | 3.849E-01 | 2.217E-01 | 2.564E-04 | 4.878E+03 | 1.706E-04 | 3.279E+03 | 3.139E-01 | 3.953E+06 | 4.739E+03 | 2.035E+01 |
| **2H** | 6.274E-01 | 4.674E+03 | 3.263E-02 | 3.650E-01 | 1.875E-01 | 1.866E-04 | 6.516E+03 | 1.184E-04 | 4.264E+03 | 1.036E+00 | 2.204E+07 | 6.281E+03 | 2.846E+01 |
| **4H** | 6.511E-01 | 2.031E+03 | 3.913E-02 | 3.923E-01 | 1.843E-01 | 1.997E-04 | 5.268E+03 | 1.301E-04 | 3.457E+03 | 3.818E-01 | 1.084E+07 | 5.208E+03 | 2.943E+01 |
| **7H** | 6.640E-01 | 2.689E+02 | 2.309E-02 | 4.079E-01 | 3.488E-01 | 3.076E-04 | 3.854E+03 | 2.012E-04 | 2.623E+03 | 1.069E-01 | 7.154E+05 | 3.704E+03 | 8.218E+00 |
| **8H** | 6.787E-01 | 1.661E+02 | 3.025E-02 | 4.257E-01 | 3.178E-01 | 3.632E-04 | 3.809E+03 | 2.638E-04 | 2.589E+03 | 5.459E-02 | 5.301E+05 | 3.656E+03 | 9.903E+00 |
| **9H** | 6.582E-01 | 1.539E+02 | 2.883E-02 | 4.007E-01 | 3.630E-01 | 2.601E-04 | 5.288E+03 | 1.913E-04 | 3.471E+03 | 3.771E-02 | 6.398E+05 | 5.173E+03 | 7.590E+00 |
| **12H** | 7.328E-01 | 1.686E+01 | 2.394E-02 | 4.970E-01 | 4.985E-01 | 3.172E-04 | 3.867E+03 | 2.263E-04 | 2.969E+03 | 6.933E-03 | 4.489E+04 | 3.685E+03 | 4.024E+00 |
| **13H** | 6.685E-01 | 1.523E+01 | 2.874E-02 | 4.136E-01 | 4.509E-01 | 3.690E-04 | 3.689E+03 | 2.706E-04 | 2.475E+03 | 4.600E-03 | 5.358E+04 | 3.577E+03 | 4.917E+00 |
| **14H** | 6.692E-01 | 1.722E+01 | 3.079E-02 | 4.148E-01 | 4.493E-01 | 2.170E-04 | 4.978E+03 | 1.438E-04 | 3.379E+03 | 4.616E-03 | 7.043E+04 | 4.890E+03 | 4.952E+00 |
| **16H** | 6.627E-01 | 6.233E+01 | 3.040E-02 | 4.059E-01 | 3.547E-01 | 2.244E-04 | 4.845E+03 | 1.468E-04 | 3.292E+03 | 1.443E-02 | 2.825E+05 | 4.743E+03 | 7.949E+00 |
| **17H** | 6.361E-01 | 1.195E+04 | 4.352E-02 | 3.747E-01 | 1.742E-01 | 2.704E-04 | 6.850E+03 | 1.396E-04 | 4.399E+03 | 1.763E+00 | 8.112E+07 | 6.754E+03 | 3.296E+01 |
| **19H** | 6.029E-01 | 4.818E+02 | 3.225E-02 | 3.383E-01 | 2.607E-01 | 2.323E-04 | 4.644E+03 | 1.389E-04 | 2.840E+03 | 1.384E-01 | 1.723E+06 | 4.561E+03 | 1.472E+01 |
| **20H** | 6.538E-01 | 3.981E+02 | 2.865E-02 | 3.956E-01 | 3.108E-01 | 2.679E-04 | 4.218E+03 | 1.735E-04 | 2.835E+03 | 1.456E-01 | 1.135E+06 | 4.101E+03 | 1.035E+01 |
| **21H** | 6.916E-01 | 1.420E+01 | 2.107E-02 | 4.424E-01 | 4.833E-01 | 2.051E-04 | 5.721E+03 | 1.425E-04 | 4.002E+03 | 2.728E-03 | 8.017E+04 | 5.525E+03 | 4.282E+00 |
| **22H** | 6.746E-01 | 2.893E+01 | 2.740E-02 | 4.211E-01 | 4.380E-01 | 2.650E-04 | 4.224E+03 | 1.780E-04 | 2.893E+03 | 8.508E-03 | 1.039E+05 | 4.114E+03 | 5.212E+00 |
| **23H** | 6.424E-01 | 6.099E+02 | 4.533E-02 | 3.820E-01 | 2.760E-01 | 1.829E-04 | 5.633E+03 | 1.184E-04 | 3.607E+03 | 1.022E-01 | 3.667E+06 | 5.593E+03 | 1.313E+01 |
| **24H** | 6.472E-01 | 4.783E+02 | 2.876E-02 | 3.878E-01 | 3.241E-01 | 2.384E-04 | 4.560E+03 | 1.534E-04 | 2.980E+03 | 1.332E-01 | 1.734E+06 | 4.468E+03 | 9.521E+00 |
| **25H** | 7.324E-01 | 9.575E+01 | 2.794E-02 | 4.966E-01 | 4.355E-01 | 1.902E-04 | 6.198E+03 | 1.431E-04 | 4.571E+03 | 1.500E-02 | 6.148E+05 | 5.986E+03 | 5.273E+00 |
| **26H** | 6.451E-01 | 2.580E+02 | 3.223E-02 | 3.855E-01 | 3.260E-01 | 2.240E-04 | 4.784E+03 | 1.431E-04 | 3.138E+03 | 6.971E-02 | 9.736E+05 | 4.704E+03 | 9.411E+00 |
| **27H** | 6.962E-01 | 1.606E+01 | 3.039E-02 | 4.479E-01 | 4.617E-01 | 1.889E-04 | 5.678E+03 | 1.314E-04 | 3.996E+03 | 3.082E-03 | 8.705E+04 | 5.580E+03 | 4.691E+00 |
| **28H** | 6.706E-01 | 3.785E+01 | 3.064E-02 | 4.163E-01 | 4.276E-01 | 2.165E-04 | 4.981E+03 | 1.465E-04 | 3.338E+03 | 6.610E-03 | 2.251E+05 | 4.890E+03 | 5.468E+00 |
| **29H** | 6.896E-01 | 1.644E+01 | 2.744E-02 | 4.396E-01 | 4.672E-01 | 2.008E-04 | 5.546E+03 | 1.376E-04 | 3.914E+03 | 3.560E-03 | 7.916E+04 | 5.390E+03 | 4.582E+00 |
| **30H** | 6.680E-01 | 1.910E+02 | 3.683E-02 | 4.128E-01 | 3.552E-01 | 1.837E-04 | 5.726E+03 | 1.224E-04 | 3.859E+03 | 3.019E-02 | 1.216E+06 | 5.656E+03 | 7.924E+00 |
| **31H** | 6.602E-01 | 4.492E+01 | 3.095E-02 | 4.035E-01 | 3.948E-01 | 2.225E-04 | 4.844E+03 | 1.461E-04 | 3.234E+03 | 9.501E-03 | 2.233E+05 | 4.760E+03 | 6.416E+00 |
| **32H** | 6.902E-01 | 1.482E+01 | 3.350E-02 | 4.405E-01 | 4.706E-01 | 2.239E-04 | 4.765E+03 | 1.533E-04 | 3.326E+03 | 3.842E-03 | 6.174E+04 | 4.692E+03 | 4.515E+00 |
| **33H** | 6.545E-01 | 5.003E+01 | 3.322E-02 | 3.964E-01 | 3.672E-01 | 2.076E-04 | 5.172E+03 | 1.382E-04 | 3.357E+03 | 9.270E-03 | 2.776E+05 | 5.093E+03 | 7.417E+00 |
| **34H** | 6.829E-01 | 2.224E+01 | 2.820E-02 | 4.311E-01 | 4.445E-01 | 2.130E-04 | 5.146E+03 | 1.451E-04 | 3.560E+03 | 5.263E-03 | 1.019E+05 | 5.039E+03 | 5.061E+00 |
| **35H** | 6.335E-01 | 1.161E+02 | 3.616E-02 | 3.722E-01 | 3.283E-01 | 1.973E-04 | 5.321E+03 | 1.252E-04 | 3.383E+03 | 2.425E-02 | 5.643E+05 | 5.257E+03 | 9.275E+00 |
| **36H** | 6.388E-01 | 4.274E+01 | 2.685E-02 | 3.782E-01 | 3.628E-01 | 2.056E-04 | 5.329E+03 | 1.314E-04 | 3.427E+03 | 8.810E-03 | 2.214E+05 | 5.210E+03 | 7.596E+00 |
| **37H** | 6.537E-01 | 2.228E+02 | 3.105E-02 | 3.955E-01 | 3.548E-01 | 2.236E-04 | 4.804E+03 | 1.454E-04 | 3.181E+03 | 5.862E-02 | 8.556E+05 | 4.720E+03 | 7.942E+00 |
| **38H** | 6.535E-01 | 2.482E+02 | 3.810E-02 | 3.951E-01 | 3.189E-01 | 1.984E-04 | 5.319E+03 | 1.306E-04 | 3.480E+03 | 4.074E-02 | 1.525E+06 | 5.254E+03 | 9.831E+00 |
| **39H** | 6.931E-01 | 1.372E+01 | 3.551E-02 | 4.442E-01 | 4.827E-01 | 2.022E-04 | 5.202E+03 | 1.407E-04 | 3.611E+03 | 2.667E-03 | 7.305E+04 | 5.138E+03 | 4.292E+00 |
| **40H** | 6.893E-01 | 1.588E+01 | 2.823E-02 | 4.393E-01 | 4.655E-01 | 2.159E-04 | 5.044E+03 | 1.477E-04 | 3.529E+03 | 3.773E-03 | 7.080E+04 | 4.941E+03 | 4.614E+00 |
| **41H** | 6.573E-01 | 2.506E+02 | 4.129E-02 | 3.995E-01 | 2.893E-01 | 1.753E-04 | 5.907E+03 | 1.155E-04 | 3.889E+03 | 3.911E-02 | 1.622E+06 | 5.856E+03 | 1.195E+01 |
| **42H** | 6.583E-01 | 5.128E+01 | 2.973E-02 | 4.010E-01 | 3.758E-01 | 2.524E-04 | 4.310E+03 | 1.643E-04 | 2.885E+03 | 1.563E-02 | 1.752E+05 | 4.224E+03 | 7.081E+00 |
| **43H** | 6.273E-01 | 4.486E+01 | 4.371E-02 | 3.654E-01 | 3.547E-01 | 1.749E-04 | 5.897E+03 | 1.097E-04 | 3.712E+03 | 7.281E-03 | 2.803E+05 | 5.853E+03 | 7.946E+00 |
| **44H** | 6.439E-01 | 6.809E+01 | 3.446E-02 | 3.843E-01 | 3.600E-01 | 2.086E-04 | 5.076E+03 | 1.341E-04 | 3.289E+03 | 1.345E-02 | 3.699E+05 | 5.008E+03 | 7.714E+00 |
| **45H** | 6.685E-01 | 3.075E+01 | 3.290E-02 | 4.132E-01 | 3.998E-01 | 1.967E-04 | 5.420E+03 | 1.297E-04 | 3.706E+03 | 6.661E-03 | 1.455E+05 | 5.329E+03 | 6.256E+00 |
| **47H** | 6.750E-01 | 2.402E+01 | 3.002E-02 | 4.213E-01 | 4.419E-01 | 2.183E-04 | 4.961E+03 | 1.458E-04 | 3.403E+03 | 6.309E-03 | 9.770E+04 | 4.869E+03 | 5.122E+00 |
| **49H** | 6.642E-01 | 2.409E+01 | 3.226E-02 | 4.084E-01 | 4.246E-01 | 2.128E-04 | 5.016E+03 | 1.411E-04 | 3.364E+03 | 5.471E-03 | 1.095E+05 | 4.937E+03 | 5.547E+00 |
| **50H** | 6.647E-01 | 1.432E+02 | 3.603E-02 | 4.088E-01 | 3.753E-01 | 1.809E-04 | 5.778E+03 | 1.206E-04 | 3.847E+03 | 2.214E-02 | 9.343E+05 | 5.717E+03 | 7.098E+00 |
| **51H** | 6.558E-01 | 1.280E+02 | 3.486E-02 | 3.977E-01 | 3.449E-01 | 2.003E-04 | 5.327E+03 | 1.308E-04 | 3.539E+03 | 2.778E-02 | 6.006E+05 | 5.253E+03 | 8.406E+00 |
| **52H** | 7.760E-01 | 5.234E+00 | 2.512E-02 | 5.588E-01 | 6.334E-01 | 2.130E-04 | 5.374E+03 | 1.649E-04 | 4.227E+03 | 1.086E-03 | 2.717E+04 | 5.223E+03 | 2.491E+00 |
| **53H** | 6.675E-01 | 1.250E+02 | 2.755E-02 | 4.121E-01 | 3.706E-01 | 2.548E-04 | 4.336E+03 | 1.690E-04 | 2.933E+03 | 3.580E-02 | 4.483E+05 | 4.233E+03 | 7.280E+00 |
| **54H** | 6.545E-01 | 5.581E+01 | 3.423E-02 | 3.967E-01 | 3.842E-01 | 2.008E-04 | 5.272E+03 | 1.318E-04 | 3.466E+03 | 1.224E-02 | 2.593E+05 | 5.203E+03 | 6.776E+00 |
| **55H** | 7.454E-01 | 6.684E+00 | 2.327E-02 | 5.144E-01 | 5.856E-01 | 2.089E-04 | 5.513E+03 | 1.580E-04 | 4.108E+03 | 1.257E-03 | 3.850E+04 | 5.360E+03 | 2.916E+00 |
| **56H** | 6.609E-01 | 8.021E+01 | 4.041E-02 | 4.041E-01 | 3.591E-01 | 1.884E-04 | 5.533E+03 | 1.237E-04 | 3.702E+03 | 1.596E-02 | 4.094E+05 | 5.476E+03 | 7.756E+00 |
| **57H** | 6.839E-01 | 6.043E+01 | 2.626E-02 | 4.324E-01 | 4.298E-01 | 2.299E-04 | 4.868E+03 | 1.572E-04 | 3.378E+03 | 1.115E-02 | 3.450E+05 | 4.736E+03 | 5.413E+00 |
| **58H** | 6.852E-01 | 6.221E+01 | 3.235E-02 | 4.341E-01 | 4.307E-01 | 1.902E-04 | 5.671E+03 | 1.320E-04 | 3.885E+03 | 9.856E-03 | 3.968E+05 | 5.581E+03 | 5.390E+00 |
| **59H** | 6.416E-01 | 1.525E+02 | 3.019E-02 | 3.814E-01 | 3.297E-01 | 2.239E-04 | 4.825E+03 | 1.413E-04 | 3.166E+03 | 4.328E-02 | 5.540E+05 | 4.735E+03 | 9.199E+00 |
| **60H** | 6.756E-01 | 1.774E+01 | 2.807E-02 | 4.222E-01 | 4.567E-01 | 2.097E-04 | 5.218E+03 | 1.424E-04 | 3.541E+03 | 3.543E-03 | 9.362E+04 | 5.109E+03 | 4.794E+00 |
| **61H** | 6.744E-01 | 2.061E+01 | 2.768E-02 | 4.207E-01 | 4.414E-01 | 2.306E-04 | 4.830E+03 | 1.548E-04 | 3.301E+03 | 5.206E-03 | 8.788E+04 | 4.710E+03 | 5.132E+00 |
| **62H** | 6.323E-01 | 6.043E+01 | 3.657E-02 | 3.710E-01 | 3.474E-01 | 1.967E-04 | 5.350E+03 | 1.245E-04 | 3.394E+03 | 1.213E-02 | 3.112E+05 | 5.287E+03 | 8.285E+00 |
| **64H** | 6.943E-01 | 4.840E+01 | 3.446E-02 | 4.456E-01 | 4.073E-01 | 2.027E-04 | 5.352E+03 | 1.437E-04 | 3.703E+03 | 8.516E-03 | 2.805E+05 | 5.268E+03 | 6.028E+00 |
| **65H** | 7.385E-01 | 9.254E+00 | 2.489E-02 | 5.047E-01 | 5.484E-01 | 2.027E-04 | 5.480E+03 | 1.467E-04 | 4.171E+03 | 2.209E-03 | 4.126E+04 | 5.338E+03 | 3.325E+00 |
| **66H** | 6.719E-01 | 5.087E+01 | 3.579E-02 | 4.177E-01 | 3.973E-01 | 1.917E-04 | 5.485E+03 | 1.290E-04 | 3.698E+03 | 9.099E-03 | 2.952E+05 | 5.417E+03 | 6.335E+00 |
| **67H** | 6.833E-01 | 4.161E+01 | 3.113E-02 | 4.318E-01 | 4.419E-01 | 2.022E-04 | 5.323E+03 | 1.389E-04 | 3.648E+03 | 6.911E-03 | 2.570E+05 | 5.233E+03 | 5.120E+00 |
| **68H** | 7.087E-01 | 2.375E+01 | 3.334E-02 | 4.643E-01 | 4.834E-01 | 2.042E-04 | 5.199E+03 | 1.458E-04 | 3.684E+03 | 4.082E-03 | 1.426E+05 | 5.123E+03 | 4.279E+00 |
| **69H** | 6.893E-01 | 1.060E+01 | 2.338E-02 | 4.396E-01 | 4.994E-01 | 2.429E-04 | 4.735E+03 | 1.677E-04 | 3.297E+03 | 2.622E-03 | 4.710E+04 | 4.586E+03 | 4.009E+00 |
| **70H** | 6.848E-01 | 2.709E+01 | 3.098E-02 | 4.335E-01 | 4.292E-01 | 2.264E-04 | 4.980E+03 | 1.545E-04 | 3.452E+03 | 6.424E-03 | 1.263E+05 | 4.866E+03 | 5.427E+00 |
| **71H** | 6.527E-01 | 9.701E+01 | 3.120E-02 | 3.947E-01 | 3.689E-01 | 2.342E-04 | 4.604E+03 | 1.522E-04 | 3.035E+03 | 2.625E-02 | 3.771E+05 | 4.523E+03 | 7.350E+00 |
| **72H** | 6.802E-01 | 4.263E+01 | 3.207E-02 | 4.280E-01 | 4.314E-01 | 2.092E-04 | 5.122E+03 | 1.416E-04 | 3.530E+03 | 8.325E-03 | 2.342E+05 | 5.032E+03 | 5.373E+00 |
| **73H** | 6.541E-01 | 5.427E+01 | 2.924E-02 | 3.964E-01 | 3.888E-01 | 2.060E-04 | 5.241E+03 | 1.350E-04 | 3.447E+03 | 1.139E-02 | 2.726E+05 | 5.145E+03 | 6.617E+00 |
| **74H** | 6.717E-01 | 6.678E+02 | 3.562E-02 | 4.172E-01 | 3.280E-01 | 1.651E-04 | 6.343E+03 | 1.105E-04 | 4.309E+03 | 1.018E-01 | 4.386E+06 | 6.270E+03 | 9.297E+00 |
| **75H** | 6.409E-01 | 6.342E+02 | 2.716E-02 | 3.806E-01 | 3.111E-01 | 2.326E-04 | 4.727E+03 | 1.460E-04 | 3.118E+03 | 1.958E-01 | 2.085E+06 | 4.618E+03 | 1.033E+01 |
| **76H** | 6.724E-01 | 1.040E+03 | 4.603E-02 | 4.180E-01 | 2.398E-01 | 1.517E-04 | 6.801E+03 | 1.020E-04 | 4.602E+03 | 1.518E-01 | 7.125E+06 | 6.750E+03 | 1.738E+01 |
| **78H** | 7.055E-01 | 1.904E+01 | 2.882E-02 | 4.601E-01 | 4.770E-01 | 1.637E-04 | 6.531E+03 | 1.170E-04 | 4.584E+03 | 2.814E-03 | 1.316E+05 | 6.429E+03 | 4.394E+00 |
| **79H** | 6.995E-01 | 1.892E+01 | 3.234E-02 | 4.523E-01 | 4.726E-01 | 2.065E-04 | 5.188E+03 | 1.462E-04 | 3.619E+03 | 3.387E-03 | 1.092E+05 | 5.107E+03 | 4.478E+00 |
| **80H** | 6.662E-01 | 2.764E+01 | 3.444E-02 | 4.106E-01 | 4.092E-01 | 1.888E-04 | 5.575E+03 | 1.253E-04 | 3.752E+03 | 5.355E-03 | 1.474E+05 | 5.506E+03 | 5.971E+00 |
| **81H** | 7.435E-01 | 7.732E+01 | 3.003E-02 | 5.123E-01 | 4.463E-01 | 1.838E-04 | 6.109E+03 | 1.401E-04 | 4.532E+03 | 1.194E-02 | 5.031E+05 | 5.983E+03 | 5.020E+00 |
| **82H** | 6.831E-01 | 3.590E+01 | 2.632E-02 | 4.314E-01 | 4.344E-01 | 2.044E-04 | 5.378E+03 | 1.383E-04 | 3.745E+03 | 6.988E-03 | 1.950E+05 | 5.254E+03 | 5.299E+00 |
| **83H** | 7.085E-01 | 1.267E+01 | 2.897E-02 | 4.640E-01 | 4.833E-01 | 1.850E-04 | 5.931E+03 | 1.281E-04 | 4.355E+03 | 2.591E-03 | 6.447E+04 | 5.773E+03 | 4.279E+00 |
| **84H** | 6.913E-01 | 1.463E+01 | 3.504E-02 | 4.417E-01 | 4.732E-01 | 1.831E-04 | 5.776E+03 | 1.264E-04 | 4.030E+03 | 2.556E-03 | 8.602E+04 | 5.698E+03 | 4.466E+00 |
| **85H** | 6.764E-01 | 2.631E+01 | 2.915E-02 | 4.230E-01 | 4.201E-01 | 2.163E-04 | 5.034E+03 | 1.442E-04 | 3.486E+03 | 5.968E-03 | 1.234E+05 | 4.928E+03 | 5.665E+00 |
| **86H** | 6.439E-01 | 1.067E+02 | 3.411E-02 | 3.840E-01 | 3.406E-01 | 2.083E-04 | 5.073E+03 | 1.331E-04 | 3.309E+03 | 2.590E-02 | 4.474E+05 | 5.006E+03 | 8.619E+00 |
| **87H** | 6.699E-01 | 3.414E+01 | 2.589E-02 | 4.151E-01 | 4.158E-01 | 1.605E-04 | 6.722E+03 | 1.057E-04 | 4.612E+03 | 6.141E-03 | 1.962E+05 | 6.598E+03 | 5.783E+00 |
| **88H** | 6.572E-01 | 7.065E+01 | 3.188E-02 | 3.996E-01 | 3.620E-01 | 2.265E-04 | 4.814E+03 | 1.489E-04 | 3.202E+03 | 1.845E-02 | 2.783E+05 | 4.725E+03 | 7.630E+00 |
| **89H** | 6.301E-01 | 1.065E+02 | 3.798E-02 | 3.684E-01 | 3.398E-01 | 2.094E-04 | 5.081E+03 | 1.334E-04 | 3.195E+03 | 2.077E-02 | 5.550E+05 | 5.016E+03 | 8.658E+00 |
| **90H** | 6.851E-01 | 2.676E+01 | 3.768E-02 | 4.338E-01 | 4.245E-01 | 1.816E-04 | 5.754E+03 | 1.244E-04 | 3.970E+03 | 4.924E-03 | 1.486E+05 | 5.691E+03 | 5.547E+00 |
| **91H** | 6.507E-01 | 3.914E+02 | 2.883E-02 | 3.918E-01 | 3.034E-01 | 2.402E-04 | 4.568E+03 | 1.552E-04 | 3.018E+03 | 1.288E-01 | 1.248E+06 | 4.471E+03 | 1.087E+01 |
| **92H** | 6.562E-01 | 1.941E+02 | 4.294E-02 | 3.985E-01 | 3.256E-01 | 1.599E-04 | 6.444E+03 | 1.052E-04 | 4.237E+03 | 2.954E-02 | 1.278E+06 | 6.398E+03 | 9.431E+00 |
| **93H** | 6.402E-01 | 4.190E+01 | 3.576E-02 | 3.799E-01 | 3.651E-01 | 1.992E-04 | 5.292E+03 | 1.263E-04 | 3.441E+03 | 8.376E-03 | 2.195E+05 | 5.226E+03 | 7.500E+00 |
| **94H** | 6.728E-01 | 2.142E+01 | 3.429E-02 | 4.191E-01 | 4.349E-01 | 1.918E-04 | 5.527E+03 | 1.297E-04 | 3.720E+03 | 4.049E-03 | 1.179E+05 | 5.454E+03 | 5.286E+00 |
| **95H** | 6.649E-01 | 1.197E+02 | 3.121E-02 | 4.090E-01 | 3.757E-01 | 2.080E-04 | 5.187E+03 | 1.376E-04 | 3.490E+03 | 2.832E-02 | 5.141E+05 | 5.100E+03 | 7.084E+00 |
| **96H** | 6.389E-01 | 7.586E+01 | 3.395E-02 | 3.783E-01 | 3.491E-01 | 2.611E-04 | 4.107E+03 | 1.646E-04 | 2.678E+03 | 2.307E-02 | 2.563E+05 | 4.038E+03 | 8.205E+00 |
| **97H** | 6.803E-01 | 2.047E+01 | 2.680E-02 | 4.282E-01 | 4.641E-01 | 2.086E-04 | 5.252E+03 | 1.409E-04 | 3.627E+03 | 5.143E-03 | 8.666E+04 | 5.137E+03 | 4.643E+00 |
| **98H** | 7.166E-01 | 8.106E+00 | 3.024E-02 | 4.747E-01 | 5.388E-01 | 2.101E-04 | 5.130E+03 | 1.517E-04 | 3.679E+03 | 1.681E-03 | 4.079E+04 | 5.040E+03 | 3.443E+00 |
| **99H** | 7.919E-01 | 4.414E+00 | 2.167E-02 | 5.837E-01 | 6.491E-01 | 1.864E-04 | 6.520E+03 | 1.489E-04 | 5.223E+03 | 7.688E-04 | 2.779E+04 | 6.229E+03 | 2.372E+00 |
| **101H** | 6.714E-01 | 1.867E+01 | 3.296E-02 | 4.171E-01 | 4.443E-01 | 1.910E-04 | 5.568E+03 | 1.264E-04 | 3.815E+03 | 4.094E-03 | 8.872E+04 | 5.481E+03 | 5.064E+00 |
| **102H** | 7.074E-01 | 7.792E+00 | 3.303E-02 | 4.627E-01 | 5.268E-01 | 2.191E-04 | 4.903E+03 | 1.560E-04 | 3.478E+03 | 1.660E-03 | 3.800E+04 | 4.821E+03 | 3.601E+00 |
| **103H** | 6.440E-01 | 1.644E+02 | 3.056E-02 | 3.843E-01 | 3.477E-01 | 2.415E-04 | 4.472E+03 | 1.543E-04 | 2.921E+03 | 4.805E-02 | 5.697E+05 | 4.388E+03 | 8.272E+00 |
| **104H** | 6.856E-01 | 1.833E+01 | 2.503E-02 | 4.345E-01 | 4.571E-01 | 2.072E-04 | 5.404E+03 | 1.412E-04 | 3.769E+03 | 3.892E-03 | 9.454E+04 | 5.257E+03 | 4.785E+00 |
| **105H** | 6.805E-01 | 2.588E+01 | 2.301E-02 | 4.283E-01 | 4.379E-01 | 1.994E-04 | 5.787E+03 | 1.331E-04 | 4.071E+03 | 6.166E-03 | 1.216E+05 | 5.566E+03 | 5.215E+00 |
| **108H** | 7.203E-01 | 1.462E+01 | 2.756E-02 | 4.797E-01 | 5.007E-01 | 2.225E-04 | 4.977E+03 | 1.593E-04 | 3.659E+03 | 3.470E-03 | 6.477E+04 | 4.855E+03 | 3.988E+00 |
| **109H** | 6.656E-01 | 2.068E+03 | 3.479E-02 | 4.099E-01 | 2.256E-01 | 1.570E-04 | 6.703E+03 | 1.027E-04 | 4.573E+03 | 3.598E-01 | 1.193E+07 | 6.617E+03 | 1.965E+01 |
| **110H** | 5.958E-01 | 7.835E+03 | 4.041E-02 | 3.318E-01 | 1.448E-01 | 1.711E-04 | 6.064E+03 | 1.014E-04 | 3.647E+03 | 1.276E+00 | 4.826E+07 | 6.009E+03 | 4.769E+01 |
| **111H** | 6.113E-01 | 7.950E+03 | 2.558E-02 | 3.468E-01 | 1.287E-01 | 1.890E-04 | 5.802E+03 | 1.131E-04 | 3.657E+03 | 2.096E+00 | 3.146E+07 | 5.670E+03 | 6.034E+01 |
| **112H** | 7.046E-01 | 9.194E+00 | 2.217E-02 | 4.590E-01 | 5.178E-01 | 3.144E-04 | 3.935E+03 | 2.209E-04 | 2.817E+03 | 3.000E-03 | 3.295E+04 | 3.773E+03 | 3.728E+00 |
| **113H** | 6.822E-01 | 3.225E+02 | 3.482E-02 | 4.302E-01 | 3.084E-01 | 1.592E-04 | 7.103E+03 | 1.058E-04 | 5.065E+03 | 6.457E-02 | 1.620E+06 | 6.795E+03 | 1.052E+01 |
| **114H** | 6.445E-01 | 1.250E+03 | 2.935E-02 | 3.849E-01 | 2.870E-01 | 2.370E-04 | 4.639E+03 | 1.510E-04 | 3.040E+03 | 3.513E-01 | 4.560E+06 | 4.535E+03 | 1.214E+01 |
| **115H** | 6.046E-01 | 2.213E+03 | 4.137E-02 | 3.393E-01 | 1.528E-01 | 1.659E-04 | 6.234E+03 | 9.874E-05 | 3.839E+03 | 4.254E-01 | 1.158E+07 | 6.184E+03 | 4.284E+01 |
| **116H** | 6.388E-01 | 1.247E+03 | 3.920E-02 | 3.778E-01 | 2.027E-01 | 2.213E-04 | 5.062E+03 | 1.433E-04 | 3.296E+03 | 2.438E-01 | 6.431E+06 | 4.970E+03 | 2.433E+01 |
| **117H** | 5.956E-01 | 6.925E+02 | 3.921E-02 | 3.305E-01 | 2.149E-01 | 2.105E-04 | 4.966E+03 | 1.249E-04 | 2.976E+03 | 1.571E-01 | 3.118E+06 | 4.914E+03 | 2.165E+01 |
| **118H** | 7.621E-01 | 1.450E+01 | 1.915E-02 | 5.385E-01 | 5.593E-01 | 1.780E-04 | 6.583E+03 | 1.330E-04 | 5.146E+03 | 3.176E-03 | 7.225E+04 | 6.342E+03 | 3.197E+00 |
| **119H** | 5.819E-01 | 1.544E+04 | 4.821E-02 | 3.162E-01 | 1.304E-01 | 1.726E-04 | 5.957E+03 | 1.002E-04 | 3.483E+03 | 2.741E+00 | 8.716E+07 | 5.917E+03 | 5.885E+01 |
| **120H** | 7.374E-01 | 9.726E+02 | 2.850E-02 | 5.037E-01 | 2.860E-01 | 1.433E-04 | 7.516E+03 | 1.023E-04 | 5.760E+03 | 1.699E-01 | 5.576E+06 | 7.359E+03 | 1.223E+01 |
| **121H** | 6.129E-01 | 9.313E+03 | 4.059E-02 | 3.499E-01 | 1.343E-01 | 1.621E-04 | 6.378E+03 | 9.958E-05 | 3.917E+03 | 1.469E+00 | 5.917E+07 | 6.327E+03 | 5.541E+01 |
| **122H** | 5.776E-01 | 1.175E+04 | 4.894E-02 | 3.116E-01 | 1.105E-01 | 1.989E-04 | 5.176E+03 | 1.141E-04 | 3.019E+03 | 2.493E+00 | 5.598E+07 | 5.140E+03 | 8.194E+01 |
| **123H** | 6.727E-01 | 1.044E+03 | 4.211E-02 | 4.183E-01 | 2.589E-01 | 1.492E-04 | 6.930E+03 | 9.877E-05 | 4.752E+03 | 1.604E-01 | 6.808E+06 | 6.874E+03 | 1.491E+01 |
| **124H** | 6.360E-01 | 5.345E+02 | 2.788E-02 | 3.749E-01 | 2.836E-01 | 1.907E-04 | 5.776E+03 | 1.206E-04 | 3.754E+03 | 1.358E-01 | 2.197E+06 | 5.641E+03 | 1.243E+01 |
| **125H** | 6.738E-01 | 1.411E+03 | 2.713E-02 | 4.199E-01 | 2.476E-01 | 1.709E-04 | 6.279E+03 | 1.129E-04 | 4.325E+03 | 2.619E-01 | 7.691E+06 | 6.175E+03 | 1.631E+01 |
| **126H** | 5.752E-01 | 3.875E+04 | 5.622E-02 | 3.086E-01 | 9.313E-02 | 1.911E-04 | 5.402E+03 | 1.117E-04 | 3.080E+03 | 6.130E+00 | 2.455E+08 | 5.365E+03 | 1.153E+02 |
| **127H** | 6.066E-01 | 6.695E+02 | 3.252E-02 | 3.418E-01 | 2.249E-01 | 2.306E-04 | 4.779E+03 | 1.424E-04 | 2.928E+03 | 1.639E-01 | 2.791E+06 | 4.682E+03 | 1.977E+01 |
| **128H** | 6.638E-01 | 1.950E+01 | 2.579E-02 | 4.078E-01 | 4.292E-01 | 2.605E-04 | 4.420E+03 | 1.730E-04 | 2.986E+03 | 5.354E-03 | 7.656E+04 | 4.291E+03 | 5.427E+00 |
| **129H** | 6.774E-01 | 6.187E+01 | 1.951E-02 | 4.242E-01 | 3.914E-01 | 2.661E-04 | 4.761E+03 | 1.764E-04 | 3.368E+03 | 1.479E-02 | 2.921E+05 | 4.467E+03 | 6.528E+00 |
| **130H** | 6.791E-01 | 4.514E+02 | 2.979E-02 | 4.266E-01 | 3.511E-01 | 1.320E-04 | 7.937E+03 | 8.793E-05 | 5.505E+03 | 7.635E-02 | 2.682E+06 | 7.845E+03 | 8.110E+00 |
| **131H** | 6.430E-01 | 6.485E+01 | 3.468E-02 | 3.827E-01 | 3.283E-01 | 1.585E-04 | 6.613E+03 | 1.021E-04 | 4.265E+03 | 1.025E-02 | 4.185E+05 | 6.540E+03 | 9.276E+00 |
| **132H** | 6.147E-01 | 2.758E+02 | 3.777E-02 | 3.505E-01 | 2.262E-01 | 1.748E-04 | 5.961E+03 | 1.088E-04 | 3.645E+03 | 4.617E-02 | 1.660E+06 | 5.903E+03 | 1.954E+01 |
| **133H** | 6.082E-01 | 1.251E+03 | 3.306E-02 | 3.440E-01 | 2.233E-01 | 2.034E-04 | 5.232E+03 | 1.228E-04 | 3.214E+03 | 3.066E-01 | 5.205E+06 | 5.156E+03 | 2.006E+01 |
| **134H** | 6.800E-01 | 2.050E+01 | 1.894E-02 | 4.280E-01 | 4.594E-01 | 4.804E-04 | 3.196E+03 | 3.256E-04 | 2.196E+03 | 1.068E-02 | 4.999E+04 | 2.978E+03 | 4.739E+00 |
| **135H** | 6.360E-01 | 3.346E+03 | 3.423E-02 | 3.751E-01 | 2.526E-01 | 2.076E-04 | 5.186E+03 | 1.314E-04 | 3.350E+03 | 7.346E-01 | 1.552E+07 | 5.093E+03 | 1.568E+01 |
| **136H** | 6.006E-01 | 2.606E+03 | 3.649E-02 | 3.357E-01 | 1.618E-01 | 1.675E-04 | 6.234E+03 | 9.929E-05 | 3.808E+03 | 4.610E-01 | 1.482E+07 | 6.170E+03 | 3.818E+01 |
| **137H** | 6.523E-01 | 7.040E+01 | 2.462E-02 | 3.941E-01 | 3.724E-01 | 1.740E-04 | 6.291E+03 | 1.121E-04 | 4.179E+03 | 1.289E-02 | 3.927E+05 | 6.156E+03 | 7.210E+00 |
| **138H** | 6.373E-01 | 2.848E+02 | 2.771E-02 | 3.765E-01 | 2.661E-01 | 1.956E-04 | 5.675E+03 | 1.246E-04 | 3.682E+03 | 6.076E-02 | 1.392E+06 | 5.549E+03 | 1.412E+01 |
| **139H** | 6.709E-01 | 1.540E+01 | 2.228E-02 | 4.166E-01 | 4.510E-01 | 3.482E-04 | 3.512E+03 | 2.307E-04 | 2.409E+03 | 5.860E-03 | 4.606E+04 | 3.356E+03 | 4.915E+00 |
| **140H** | 6.639E-01 | 9.160E+03 | 3.401E-02 | 4.076E-01 | 1.594E-01 | 1.759E-04 | 6.037E+03 | 1.153E-04 | 4.096E+03 | 1.745E+00 | 4.824E+07 | 5.948E+03 | 3.935E+01 |
| **141H** | 5.561E-01 | 5.735E+03 | 4.003E-02 | 2.900E-01 | 1.198E-01 | 1.963E-04 | 5.313E+03 | 1.088E-04 | 2.967E+03 | 1.228E+00 | 2.762E+07 | 5.260E+03 | 6.964E+01 |
| **142H** | 6.340E-01 | 8.884E+02 | 3.530E-02 | 3.723E-01 | 2.525E-01 | 1.705E-04 | 6.156E+03 | 1.085E-04 | 3.918E+03 | 1.411E-01 | 5.630E+06 | 6.085E+03 | 1.568E+01 |
| **143H** | 6.308E-01 | 8.379E+01 | 2.808E-02 | 3.684E-01 | 3.067E-01 | 1.818E-04 | 5.945E+03 | 1.123E-04 | 3.857E+03 | 1.774E-02 | 4.136E+05 | 5.829E+03 | 1.063E+01 |
| **144H** | 6.327E-01 | 1.440E+02 | 2.919E-02 | 3.716E-01 | 3.080E-01 | 1.930E-04 | 5.599E+03 | 1.214E-04 | 3.601E+03 | 2.984E-02 | 7.029E+05 | 5.489E+03 | 1.054E+01 |
| **145H** | 5.983E-01 | 6.915E+03 | 4.330E-02 | 3.325E-01 | 1.221E-01 | 1.890E-04 | 5.493E+03 | 1.124E-04 | 3.325E+03 | 1.465E+00 | 3.288E+07 | 5.445E+03 | 6.712E+01 |
| **146H** | 5.520E-01 | 3.239E+03 | 3.972E-02 | 2.873E-01 | 1.394E-01 | 1.733E-04 | 5.983E+03 | 9.481E-05 | 3.347E+03 | 6.197E-01 | 1.711E+07 | 5.930E+03 | 5.148E+01 |
| **147H** | 6.212E-01 | 1.588E+04 | 3.650E-02 | 3.579E-01 | 1.102E-01 | 1.644E-04 | 6.345E+03 | 1.008E-04 | 4.009E+03 | 2.842E+00 | 8.910E+07 | 6.279E+03 | 8.239E+01 |
| **148H** | 6.401E-01 | 1.240E+04 | 3.961E-02 | 3.795E-01 | 1.412E-01 | 1.601E-04 | 6.490E+03 | 1.005E-04 | 4.253E+03 | 2.242E+00 | 6.890E+07 | 6.426E+03 | 5.014E+01 |
| **149H** | 6.350E-01 | 7.754E+02 | 4.459E-02 | 3.747E-01 | 2.063E-01 | 1.763E-04 | 5.833E+03 | 1.099E-04 | 3.777E+03 | 1.661E-01 | 3.665E+06 | 5.792E+03 | 2.350E+01 |
| **150H** | 6.125E-01 | 5.408E+03 | 4.293E-02 | 3.484E-01 | 1.617E-01 | 1.819E-04 | 5.711E+03 | 1.124E-04 | 3.488E+03 | 9.261E-01 | 3.184E+07 | 5.661E+03 | 3.824E+01 |
| **151H** | 6.966E-01 | 4.209E+01 | 3.297E-02 | 4.487E-01 | 4.189E-01 | 1.729E-04 | 6.320E+03 | 1.231E-04 | 4.371E+03 | 7.211E-03 | 2.523E+05 | 6.221E+03 | 5.699E+00 |
| **152H** | 6.162E-01 | 3.035E+02 | 3.129E-02 | 3.529E-01 | 2.703E-01 | 1.695E-04 | 6.252E+03 | 1.034E-04 | 3.921E+03 | 6.150E-02 | 1.518E+06 | 6.166E+03 | 1.368E+01 |
| **153H** | 5.834E-01 | 6.902E+03 | 4.330E-02 | 3.183E-01 | 1.454E-01 | 1.765E-04 | 5.862E+03 | 1.024E-04 | 3.456E+03 | 1.215E+00 | 3.946E+07 | 5.814E+03 | 4.730E+01 |
| **154H** | 6.239E-01 | 6.102E+03 | 4.495E-02 | 3.606E-01 | 1.436E-01 | 1.653E-04 | 6.270E+03 | 1.042E-04 | 3.898E+03 | 9.526E-01 | 3.912E+07 | 6.218E+03 | 4.851E+01 |
| **155H** | 5.528E-01 | 3.647E+03 | 4.056E-02 | 2.873E-01 | 1.094E-01 | 1.881E-04 | 5.541E+03 | 1.046E-04 | 3.063E+03 | 6.555E-01 | 2.058E+07 | 5.485E+03 | 8.360E+01 |
| **156H** | 6.519E-01 | 4.387E+01 | 3.305E-02 | 3.934E-01 | 3.698E-01 | 1.824E-04 | 5.994E+03 | 1.214E-04 | 3.917E+03 | 7.936E-03 | 2.503E+05 | 5.912E+03 | 7.311E+00 |
| **157H** | 6.784E-01 | 1.768E+01 | 1.840E-02 | 4.256E-01 | 4.518E-01 | 4.234E-04 | 3.307E+03 | 2.841E-04 | 2.310E+03 | 9.332E-03 | 4.417E+04 | 3.078E+03 | 4.898E+00 |
| **158H** | 6.102E-01 | 4.133E+02 | 3.397E-02 | 3.468E-01 | 2.863E-01 | 2.254E-04 | 4.829E+03 | 1.365E-04 | 2.986E+03 | 1.285E-01 | 1.364E+06 | 4.734E+03 | 1.220E+01 |
| **160H** | 6.338E-01 | 2.735E+02 | 3.036E-02 | 3.730E-01 | 2.858E-01 | 1.869E-04 | 5.755E+03 | 1.182E-04 | 3.686E+03 | 4.847E-02 | 1.555E+06 | 5.657E+03 | 1.224E+01 |
| **161H** | 7.337E-01 | 7.727E+00 | 1.949E-02 | 4.979E-01 | 5.590E-01 | 2.917E-04 | 4.418E+03 | 2.116E-04 | 3.322E+03 | 2.260E-03 | 3.319E+04 | 4.201E+03 | 3.200E+00 |
| **162H** | 5.877E-01 | 1.336E+02 | 3.872E-02 | 3.231E-01 | 2.630E-01 | 1.365E-04 | 7.540E+03 | 7.978E-05 | 4.466E+03 | 2.034E-02 | 8.930E+05 | 7.488E+03 | 1.445E+01 |
| **163H** | 5.999E-01 | 1.405E+03 | 3.402E-02 | 3.352E-01 | 2.121E-01 | 1.990E-04 | 5.343E+03 | 1.199E-04 | 3.216E+03 | 2.811E-01 | 7.149E+06 | 5.267E+03 | 2.224E+01 |
| **164H** | 6.628E-01 | 5.964E+01 | 3.021E-02 | 4.061E-01 | 3.502E-01 | 1.961E-04 | 5.520E+03 | 1.323E-04 | 3.653E+03 | 1.053E-02 | 3.474E+05 | 5.424E+03 | 8.153E+00 |
| **165H** | 6.082E-01 | 6.983E+03 | 3.864E-02 | 3.444E-01 | 1.514E-01 | 1.592E-04 | 6.537E+03 | 9.609E-05 | 4.033E+03 | 1.116E+00 | 4.406E+07 | 6.471E+03 | 4.365E+01 |
| **166H** | 6.788E-01 | 2.098E+01 | 2.162E-02 | 4.262E-01 | 4.486E-01 | 2.752E-04 | 4.372E+03 | 1.843E-04 | 3.034E+03 | 6.464E-03 | 7.389E+04 | 4.203E+03 | 4.968E+00 |
| **167H** | 6.042E-01 | 4.085E+03 | 3.528E-02 | 3.397E-01 | 1.957E-01 | 2.118E-04 | 5.012E+03 | 1.269E-04 | 3.073E+03 | 9.319E-01 | 1.806E+07 | 4.939E+03 | 2.612E+01 |
| **168H** | 6.236E-01 | 1.222E+04 | 4.344E-02 | 3.610E-01 | 1.135E-01 | 1.886E-04 | 5.486E+03 | 1.155E-04 | 3.496E+03 | 2.363E+00 | 6.338E+07 | 5.440E+03 | 7.764E+01 |
| **169H** | 6.846E-01 | 7.289E+01 | 3.211E-02 | 4.337E-01 | 3.901E-01 | 2.352E-04 | 4.649E+03 | 1.600E-04 | 3.226E+03 | 1.971E-02 | 2.762E+05 | 4.552E+03 | 6.571E+00 |
| **170H** | 6.187E-01 | 4.826E+02 | 3.587E-02 | 3.555E-01 | 2.509E-01 | 1.900E-04 | 5.549E+03 | 1.185E-04 | 3.428E+03 | 8.835E-02 | 2.660E+06 | 5.480E+03 | 1.589E+01 |
| **171H** | 5.964E-01 | 9.121E+03 | 4.383E-02 | 3.306E-01 | 1.313E-01 | 1.749E-04 | 5.915E+03 | 1.034E-04 | 3.566E+03 | 1.675E+00 | 5.007E+07 | 5.865E+03 | 5.803E+01 |
| **172H** | 5.936E-01 | 3.776E+03 | 4.010E-02 | 3.281E-01 | 1.871E-01 | 2.044E-04 | 5.120E+03 | 1.214E-04 | 3.055E+03 | 8.303E-01 | 1.734E+07 | 5.067E+03 | 2.855E+01 |
| **173H** | 6.059E-01 | 5.988E+02 | 3.009E-02 | 3.423E-01 | 2.584E-01 | 1.685E-04 | 6.389E+03 | 1.025E-04 | 3.926E+03 | 1.019E-01 | 3.532E+06 | 6.284E+03 | 1.498E+01 |
| **174H** | 6.203E-01 | 1.110E+05 | 4.407E-02 | 3.568E-01 | 6.519E-02 | 1.540E-04 | 6.700E+03 | 9.466E-05 | 4.212E+03 | 1.767E+01 | 6.996E+08 | 6.645E+03 | 2.353E+02 |
| **175H** | 6.233E-01 | 2.019E+03 | 4.104E-02 | 3.605E-01 | 2.090E-01 | 2.293E-04 | 4.590E+03 | 1.438E-04 | 2.864E+03 | 4.837E-01 | 8.488E+06 | 4.537E+03 | 2.290E+01 |
| **176H** | 6.155E-01 | 1.865E+03 | 4.294E-02 | 3.523E-01 | 2.300E-01 | 2.227E-04 | 4.709E+03 | 1.366E-04 | 2.922E+03 | 4.231E-01 | 8.308E+06 | 4.656E+03 | 1.890E+01 |
| **177H** | 5.854E-01 | 1.634E+04 | 3.946E-02 | 3.200E-01 | 1.231E-01 | 2.018E-04 | 5.210E+03 | 1.175E-04 | 3.079E+03 | 3.412E+00 | 7.919E+07 | 5.149E+03 | 6.598E+01 |
| **178H** | 6.664E-01 | 3.856E+03 | 3.785E-02 | 4.107E-01 | 2.313E-01 | 1.580E-04 | 6.597E+03 | 1.038E-04 | 4.489E+03 | 6.720E-01 | 2.217E+07 | 6.528E+03 | 1.869E+01 |
| **181H** | 6.022E-01 | 4.409E+03 | 3.143E-02 | 3.372E-01 | 1.597E-01 | 2.088E-04 | 5.215E+03 | 1.269E-04 | 3.164E+03 | 9.365E-01 | 2.116E+07 | 5.129E+03 | 3.919E+01 |
| **182H** | 5.910E-01 | 3.685E+04 | 4.150E-02 | 3.261E-01 | 1.089E-01 | 1.957E-04 | 5.318E+03 | 1.149E-04 | 3.183E+03 | 7.179E+00 | 1.899E+08 | 5.265E+03 | 8.427E+01 |
| **183H** | 6.605E-01 | 2.197E+02 | 3.015E-02 | 4.036E-01 | 3.458E-01 | 2.435E-04 | 4.502E+03 | 1.605E-04 | 3.009E+03 | 5.669E-02 | 8.854E+05 | 4.405E+03 | 8.363E+00 |
| **184H** | 6.347E-01 | 3.171E+02 | 2.871E-02 | 3.737E-01 | 2.989E-01 | 1.880E-04 | 5.776E+03 | 1.202E-04 | 3.679E+03 | 5.742E-02 | 1.810E+06 | 5.672E+03 | 1.119E+01 |
| **185H** | 6.340E-01 | 1.624E+03 | 4.477E-02 | 3.722E-01 | 1.842E-01 | 1.701E-04 | 6.064E+03 | 1.085E-04 | 3.839E+03 | 2.622E-01 | 1.007E+07 | 6.019E+03 | 2.948E+01 |
| **186H** | 6.718E-01 | 8.631E+02 | 3.548E-02 | 4.172E-01 | 2.220E-01 | 1.464E-04 | 7.112E+03 | 9.660E-05 | 4.887E+03 | 1.396E-01 | 5.363E+06 | 7.040E+03 | 2.028E+01 |
| **187H** | 6.591E-01 | 7.181E+02 | 3.200E-02 | 4.021E-01 | 3.345E-01 | 2.516E-04 | 4.347E+03 | 1.657E-04 | 2.896E+03 | 1.831E-01 | 2.871E+06 | 4.260E+03 | 8.938E+00 |
| **188H** | 6.152E-01 | 3.833E+03 | 4.398E-02 | 3.514E-01 | 1.710E-01 | 1.557E-04 | 6.604E+03 | 9.607E-05 | 4.072E+03 | 5.794E-01 | 2.544E+07 | 6.558E+03 | 3.421E+01 |
| **189H** | 6.033E-01 | 3.383E+03 | 4.134E-02 | 3.384E-01 | 1.569E-01 | 1.882E-04 | 5.521E+03 | 1.136E-04 | 3.340E+03 | 6.256E-01 | 1.850E+07 | 5.471E+03 | 4.063E+01 |
| **190H** | 6.364E-01 | 2.158E+03 | 4.513E-02 | 3.747E-01 | 1.880E-01 | 1.799E-04 | 5.732E+03 | 1.128E-04 | 3.710E+03 | 4.032E-01 | 1.159E+07 | 5.689E+03 | 2.828E+01 |
| **191H** | 6.335E-01 | 5.117E+03 | 3.264E-02 | 3.715E-01 | 1.529E-01 | 1.551E-04 | 6.812E+03 | 9.647E-05 | 4.436E+03 | 8.560E-01 | 3.075E+07 | 6.716E+03 | 4.277E+01 |
| **192H** | 5.907E-01 | 2.874E+02 | 3.440E-02 | 3.244E-01 | 2.213E-01 | 1.303E-04 | 7.935E+03 | 7.683E-05 | 4.705E+03 | 4.300E-02 | 1.964E+06 | 7.869E+03 | 2.041E+01 |
| **193H** | 6.289E-01 | 1.564E+02 | 2.952E-02 | 3.667E-01 | 3.005E-01 | 2.324E-04 | 4.745E+03 | 1.512E-04 | 2.927E+03 | 2.826E-02 | 8.798E+05 | 4.646E+03 | 1.108E+01 |
| **194H** | 6.579E-01 | 5.577E+03 | 3.281E-02 | 4.003E-01 | 1.714E-01 | 2.016E-04 | 5.402E+03 | 1.321E-04 | 3.622E+03 | 1.124E+00 | 2.778E+07 | 5.290E+03 | 3.406E+01 |
| **195H** | 5.908E-01 | 1.525E+03 | 3.490E-02 | 3.251E-01 | 1.850E-01 | 2.167E-04 | 4.890E+03 | 1.278E-04 | 2.906E+03 | 3.927E-01 | 6.183E+06 | 4.823E+03 | 2.922E+01 |
| **196H** | 6.502E-01 | 1.217E+03 | 3.351E-02 | 3.910E-01 | 2.362E-01 | 2.109E-04 | 5.176E+03 | 1.379E-04 | 3.389E+03 | 2.188E-01 | 6.806E+06 | 5.076E+03 | 1.793E+01 |
| **197H** | 6.743E-01 | 3.132E+01 | 2.468E-02 | 4.207E-01 | 4.314E-01 | 1.934E-04 | 5.742E+03 | 1.315E-04 | 3.875E+03 | 6.359E-03 | 1.632E+05 | 5.610E+03 | 5.373E+00 |
| **198H** | 6.150E-01 | 1.605E+03 | 3.456E-02 | 3.510E-01 | 2.353E-01 | 2.032E-04 | 5.253E+03 | 1.256E-04 | 3.251E+03 | 3.252E-01 | 7.984E+06 | 5.173E+03 | 1.806E+01 |
| **199H** | 7.432E-01 | 5.683E+00 | 1.775E-02 | 5.112E-01 | 5.938E-01 | 9.001E-04 | 3.616E+03 | 7.245E-04 | 2.731E+03 | 3.814E-03 | 1.810E+04 | 3.319E+03 | 2.835E+00 |
| **200H** | 6.404E-01 | 2.372E+03 | 3.937E-02 | 3.801E-01 | 2.383E-01 | 2.511E-04 | 4.270E+03 | 1.586E-04 | 2.792E+03 | 6.578E-01 | 8.650E+06 | 4.199E+03 | 1.760E+01 |
| **201H** | 6.279E-01 | 1.130E+03 | 3.130E-02 | 3.653E-01 | 2.264E-01 | 2.079E-04 | 5.162E+03 | 1.290E-04 | 3.302E+03 | 2.770E-01 | 4.697E+06 | 5.078E+03 | 1.952E+01 |
| **202H** | 5.967E-01 | 2.611E+02 | 3.898E-02 | 3.310E-01 | 2.446E-01 | 1.825E-04 | 5.704E+03 | 1.080E-04 | 3.450E+03 | 5.210E-02 | 1.323E+06 | 5.648E+03 | 1.672E+01 |
| **203H** | 6.776E-01 | 1.593E+03 | 2.308E-02 | 4.243E-01 | 2.515E-01 | 1.447E-04 | 7.671E+03 | 9.524E-05 | 5.418E+03 | 2.423E-01 | 1.057E+07 | 7.474E+03 | 1.581E+01 |
| **206H** | 6.204E-01 | 3.833E+03 | 3.317E-02 | 3.570E-01 | 1.929E-01 | 2.238E-04 | 4.847E+03 | 1.349E-04 | 3.135E+03 | 1.077E+00 | 1.377E+07 | 4.744E+03 | 2.688E+01 |
| **207H** | 6.544E-01 | 8.598E+01 | 2.335E-02 | 3.965E-01 | 3.477E-01 | 2.838E-04 | 4.177E+03 | 1.854E-04 | 2.765E+03 | 2.274E-02 | 3.379E+05 | 4.030E+03 | 8.271E+00 |
| **208H** | 6.415E-01 | 2.406E+02 | 2.753E-02 | 3.811E-01 | 2.965E-01 | 2.188E-04 | 5.083E+03 | 1.409E-04 | 3.268E+03 | 4.614E-02 | 1.284E+06 | 4.969E+03 | 1.137E+01 |
| **209H** | 7.309E-01 | 2.201E+01 | 2.037E-02 | 4.944E-01 | 4.678E-01 | 2.019E-04 | 5.750E+03 | 1.445E-04 | 4.337E+03 | 4.823E-03 | 1.084E+05 | 5.554E+03 | 4.570E+00 |
| **210H** | 6.723E-01 | 2.547E+01 | 2.175E-02 | 4.181E-01 | 4.275E-01 | 2.698E-04 | 4.406E+03 | 1.801E-04 | 2.992E+03 | 6.185E-03 | 1.234E+05 | 4.245E+03 | 5.470E+00 |

| **LGG** | ***F*_szm.sze_** | ***F*_szm.lze_** | ***F*_szm.glnu_** | ***F*_szm.zsnu_** | ***F*_szm.z.perc_** | ***F*_szm.lgze_** | ***F*_szm.hgze_** | ***F*_szm.szlge_** | ***F*_szm.szhge_** | ***F*_szm.lzlge_** | ***F*_szm.lzhge_** | ***F*_szm.gl.var_** | ***F*_szm.zs.var_** |
| --- | --- | --- | --- | --- | --- | --- | --- | --- | --- | --- | --- | --- | --- |
| **1L** | 7.463E-01 | 9.475E+00 | 1.910E-02 | 5.157E-01 | 5.485E-01 | 4.584E-04 | 6.364E+03 | 3.938E-04 | 4.863E+03 | 2.433E-03 | 5.573E+04 | 5.882E+03 | 3.324E+00 |
| **3L** | 6.157E-01 | 1.633E+02 | 3.154E-02 | 3.519E-01 | 2.913E-01 | 2.200E-04 | 4.925E+03 | 1.358E-04 | 3.065E+03 | 3.611E-02 | 7.578E+05 | 4.832E+03 | 1.178E+01 |
| **4L** | 6.095E-01 | 6.528E+02 | 4.285E-02 | 3.450E-01 | 2.504E-01 | 1.572E-04 | 7.260E+03 | 9.786E-05 | 4.546E+03 | 1.018E-01 | 4.193E+06 | 7.098E+03 | 1.595E+01 |
| **5L** | 6.476E-01 | 4.533E+01 | 3.688E-02 | 3.890E-01 | 3.789E-01 | 1.686E-04 | 6.598E+03 | 1.103E-04 | 4.281E+03 | 7.765E-03 | 2.731E+05 | 6.431E+03 | 6.965E+00 |
| **7L** | 6.737E-01 | 2.024E+03 | 3.519E-02 | 4.194E-01 | 2.523E-01 | 4.045E-04 | 7.040E+03 | 3.527E-04 | 4.734E+03 | 2.796E-01 | 1.471E+07 | 6.755E+03 | 1.570E+01 |
| **9L** | 6.953E-01 | 4.642E+01 | 3.426E-02 | 4.469E-01 | 4.059E-01 | 2.357E-04 | 4.664E+03 | 1.679E-04 | 3.224E+03 | 1.080E-02 | 2.037E+05 | 4.588E+03 | 6.070E+00 |
| **11L** | 6.029E-01 | 1.007E+03 | 3.885E-02 | 3.377E-01 | 1.904E-01 | 1.783E-04 | 5.858E+03 | 1.076E-04 | 3.560E+03 | 1.754E-01 | 5.816E+06 | 5.794E+03 | 2.759E+01 |
| **12L** | 6.079E-01 | 3.111E+04 | 3.238E-02 | 3.440E-01 | 1.346E-01 | 1.713E-04 | 6.193E+03 | 1.049E-04 | 3.763E+03 | 5.257E+00 | 1.848E+08 | 6.110E+03 | 5.519E+01 |
| **13L** | 5.874E-01 | 2.680E+03 | 3.276E-02 | 3.216E-01 | 1.861E-01 | 2.004E-04 | 5.313E+03 | 1.196E-04 | 3.093E+03 | 4.158E-01 | 1.740E+07 | 5.237E+03 | 2.886E+01 |
| **14L** | 7.048E-01 | 9.763E+00 | 1.922E-02 | 4.591E-01 | 5.129E-01 | 2.632E-04 | 5.312E+03 | 1.911E-04 | 3.658E+03 | 1.857E-03 | 6.147E+04 | 5.043E+03 | 3.800E+00 |
| **15L** | 6.734E-01 | 1.042E+02 | 4.167E-02 | 4.194E-01 | 3.104E-01 | 1.663E-04 | 6.314E+03 | 1.128E-04 | 4.266E+03 | 1.706E-02 | 6.409E+05 | 6.234E+03 | 1.038E+01 |
| **16L** | 5.983E-01 | 7.224E+03 | 3.261E-02 | 3.330E-01 | 1.152E-01 | 2.452E-04 | 4.488E+03 | 1.468E-04 | 2.729E+03 | 2.039E+00 | 2.644E+07 | 4.385E+03 | 7.538E+01 |
| **17L** | 6.067E-01 | 7.866E+03 | 3.088E-02 | 3.424E-01 | 1.351E-01 | 2.150E-04 | 5.087E+03 | 1.303E-04 | 3.133E+03 | 1.576E+00 | 4.029E+07 | 4.972E+03 | 5.477E+01 |
| **18L** | 6.546E-01 | 1.676E+02 | 2.601E-02 | 3.963E-01 | 2.936E-01 | 2.820E-04 | 4.279E+03 | 1.890E-04 | 2.827E+03 | 4.055E-02 | 7.116E+05 | 4.117E+03 | 1.160E+01 |
| **19L** | 7.159E-01 | 8.986E+00 | 2.146E-02 | 4.739E-01 | 5.311E-01 | 2.225E-04 | 5.210E+03 | 1.619E-04 | 3.719E+03 | 1.864E-03 | 4.656E+04 | 5.039E+03 | 3.544E+00 |
| **20L** | 6.084E-01 | 5.369E+02 | 4.096E-02 | 3.441E-01 | 2.374E-01 | 1.892E-04 | 5.500E+03 | 1.161E-04 | 3.333E+03 | 9.910E-02 | 2.956E+06 | 5.449E+03 | 1.774E+01 |
| **21L** | 6.730E-01 | 5.644E+01 | 3.305E-02 | 4.187E-01 | 3.810E-01 | 1.951E-04 | 5.446E+03 | 1.311E-04 | 3.696E+03 | 1.149E-02 | 2.824E+05 | 5.364E+03 | 6.889E+00 |
| **22L** | 7.021E-01 | 1.932E+01 | 2.398E-02 | 4.557E-01 | 4.721E-01 | 1.864E-04 | 5.969E+03 | 1.306E-04 | 4.246E+03 | 3.575E-03 | 1.099E+05 | 5.820E+03 | 4.487E+00 |
| **24L** | 6.435E-01 | 2.897E+01 | 2.869E-02 | 3.838E-01 | 3.858E-01 | 2.370E-04 | 4.685E+03 | 1.538E-04 | 3.023E+03 | 6.474E-03 | 1.352E+05 | 4.583E+03 | 6.716E+00 |
| **25L** | 7.911E-01 | 3.544E+00 | 3.286E-02 | 5.815E-01 | 6.792E-01 | 1.943E-04 | 5.519E+03 | 1.553E-04 | 4.345E+03 | 6.871E-04 | 1.929E+04 | 5.432E+03 | 2.165E+00 |
| **26L** | 6.609E-01 | 1.772E+01 | 2.666E-02 | 4.045E-01 | 4.307E-01 | 2.570E-04 | 4.411E+03 | 1.718E-04 | 2.930E+03 | 4.432E-03 | 7.685E+04 | 4.294E+03 | 5.391E+00 |
| **27L** | 6.690E-01 | 1.807E+01 | 1.887E-02 | 4.140E-01 | 4.361E-01 | 2.791E-04 | 4.603E+03 | 1.893E-04 | 3.089E+03 | 4.601E-03 | 8.424E+04 | 4.382E+03 | 5.257E+00 |
| **28L** | 7.042E-01 | 1.237E+01 | 2.743E-02 | 4.585E-01 | 4.940E-01 | 2.017E-04 | 5.412E+03 | 1.428E-04 | 3.822E+03 | 2.488E-03 | 6.464E+04 | 5.293E+03 | 4.098E+00 |
| **29L** | 5.890E-01 | 3.162E+03 | 3.789E-02 | 3.245E-01 | 1.438E-01 | 1.721E-04 | 6.077E+03 | 1.031E-04 | 3.547E+03 | 4.890E-01 | 2.069E+07 | 6.014E+03 | 4.836E+01 |
| **30L** | 6.669E-01 | 1.569E+01 | 2.497E-02 | 4.116E-01 | 4.467E-01 | 2.635E-04 | 4.339E+03 | 1.772E-04 | 2.901E+03 | 4.188E-03 | 6.305E+04 | 4.213E+03 | 5.011E+00 |
| **31L** | 7.091E-01 | 9.721E+00 | 2.595E-02 | 4.650E-01 | 5.123E-01 | 2.116E-04 | 5.423E+03 | 1.511E-04 | 3.865E+03 | 2.059E-03 | 4.975E+04 | 5.264E+03 | 3.808E+00 |
| **32L** | 5.954E-01 | 4.502E+03 | 4.399E-02 | 3.304E-01 | 1.617E-01 | 2.003E-04 | 5.170E+03 | 1.191E-04 | 3.091E+03 | 8.447E-01 | 2.412E+07 | 5.127E+03 | 3.825E+01 |
| **33L** | 6.899E-01 | 1.293E+01 | 2.212E-02 | 4.399E-01 | 4.748E-01 | 2.421E-04 | 4.847E+03 | 1.701E-04 | 3.332E+03 | 2.807E-03 | 6.472E+04 | 4.687E+03 | 4.434E+00 |
| **34L** | 7.565E-01 | 4.923E+00 | 1.734E-02 | 5.300E-01 | 6.180E-01 | 3.775E-04 | 3.793E+03 | 2.931E-04 | 2.854E+03 | 1.659E-03 | 1.874E+04 | 3.536E+03 | 2.618E+00 |
| **35L** | 7.702E-01 | 3.974E+00 | 1.984E-02 | 5.499E-01 | 6.511E-01 | 3.147E-04 | 4.193E+03 | 2.521E-04 | 3.168E+03 | 1.027E-03 | 1.847E+04 | 3.996E+03 | 2.358E+00 |
| **36L** | 6.841E-01 | 2.023E+01 | 2.668E-02 | 4.325E-01 | 4.401E-01 | 2.022E-04 | 5.459E+03 | 1.403E-04 | 3.717E+03 | 3.827E-03 | 1.121E+05 | 5.341E+03 | 5.163E+00 |
| **37L** | 7.114E-01 | 8.669E+00 | 2.169E-02 | 4.678E-01 | 5.254E-01 | 2.314E-04 | 5.232E+03 | 1.701E-04 | 3.667E+03 | 1.619E-03 | 5.109E+04 | 5.048E+03 | 3.621E+00 |
| **38L** | 6.968E-01 | 1.849E+01 | 2.610E-02 | 4.488E-01 | 4.670E-01 | 2.177E-04 | 5.157E+03 | 1.524E-04 | 3.630E+03 | 3.799E-03 | 9.381E+04 | 5.020E+03 | 4.584E+00 |
| **39L** | 7.206E-01 | 1.989E+01 | 2.992E-02 | 4.802E-01 | 5.010E-01 | 1.424E-04 | 7.442E+03 | 1.038E-04 | 5.332E+03 | 2.496E-03 | 1.629E+05 | 7.333E+03 | 3.984E+00 |
| **40L** | 6.783E-01 | 1.760E+01 | 1.917E-02 | 4.255E-01 | 4.419E-01 | 4.653E-04 | 3.000E+03 | 3.099E-04 | 2.104E+03 | 9.503E-03 | 4.187E+04 | 2.790E+03 | 5.120E+00 |
| **41L** | 7.177E-01 | 7.905E+00 | 1.810E-02 | 4.763E-01 | 5.400E-01 | 3.616E-04 | 4.299E+03 | 2.667E-04 | 3.096E+03 | 2.495E-03 | 3.323E+04 | 4.033E+03 | 3.428E+00 |
| **42L** | 6.526E-01 | 1.143E+02 | 3.451E-02 | 3.941E-01 | 3.512E-01 | 2.487E-04 | 4.288E+03 | 1.649E-04 | 2.771E+03 | 2.404E-02 | 5.510E+05 | 4.224E+03 | 8.108E+00 |
| **43L** | 7.057E-01 | 8.233E+00 | 2.205E-02 | 4.605E-01 | 5.331E-01 | 2.758E-04 | 4.273E+03 | 1.968E-04 | 3.013E+03 | 2.321E-03 | 3.325E+04 | 4.118E+03 | 3.517E+00 |
| **44L** | 6.343E-01 | 2.041E+02 | 3.168E-02 | 3.725E-01 | 2.647E-01 | 1.791E-04 | 5.970E+03 | 1.143E-04 | 3.815E+03 | 3.670E-02 | 1.148E+06 | 5.875E+03 | 1.427E+01 |
| **45L** | 7.958E-01 | 3.553E+00 | 1.915E-02 | 5.892E-01 | 6.776E-01 | 2.503E-04 | 6.042E+03 | 2.123E-04 | 4.734E+03 | 6.785E-04 | 2.320E+04 | 5.751E+03 | 2.177E+00 |
| **46L** | 5.976E-01 | 7.122E+01 | 3.527E-02 | 3.321E-01 | 2.906E-01 | 2.289E-04 | 4.642E+03 | 1.374E-04 | 2.783E+03 | 1.641E-02 | 3.225E+05 | 4.577E+03 | 1.184E+01 |
| **47L** | 5.302E-01 | 3.409E+03 | 4.147E-02 | 2.675E-01 | 1.293E-01 | 1.915E-04 | 5.410E+03 | 1.034E-04 | 2.828E+03 | 5.832E-01 | 1.999E+07 | 5.365E+03 | 5.983E+01 |
| **48L** | 5.722E-01 | 1.080E+05 | 4.390E-02 | 3.078E-01 | 7.036E-02 | 1.772E-04 | 5.843E+03 | 1.007E-04 | 3.393E+03 | 1.864E+01 | 6.273E+08 | 5.793E+03 | 2.020E+02 |
| **49L** | 6.226E-01 | 1.014E+02 | 1.679E-02 | 3.603E-01 | 3.213E-01 | 4.685E-03 | 1.295E+03 | 3.048E-03 | 8.006E+02 | 1.144E+00 | 1.778E+05 | 1.018E+03 | 9.683E+00 |
| **50L** | 6.613E-01 | 6.294E+01 | 2.896E-02 | 4.044E-01 | 3.425E-01 | 2.198E-04 | 4.994E+03 | 1.469E-04 | 3.317E+03 | 1.295E-02 | 3.125E+05 | 4.892E+03 | 8.522E+00 |
| **51L** | 6.465E-01 | 1.214E+02 | 3.356E-02 | 3.869E-01 | 3.285E-01 | 2.167E-04 | 4.916E+03 | 1.415E-04 | 3.174E+03 | 2.530E-02 | 5.909E+05 | 4.842E+03 | 9.268E+00 |
| **52L** | 6.276E-01 | 1.996E+02 | 4.002E-02 | 3.657E-01 | 3.281E-01 | 1.911E-04 | 5.456E+03 | 1.222E-04 | 3.373E+03 | 3.193E-02 | 1.254E+06 | 5.404E+03 | 9.292E+00 |
| **53L** | 5.923E-01 | 2.647E+04 | 4.222E-02 | 3.273E-01 | 8.573E-02 | 1.441E-04 | 7.396E+03 | 8.362E-05 | 4.549E+03 | 3.876E+00 | 1.811E+08 | 7.253E+03 | 1.360E+02 |
| **55L** | 6.049E-01 | 9.779E+01 | 3.365E-02 | 3.400E-01 | 2.854E-01 | 1.587E-04 | 6.587E+03 | 9.758E-05 | 3.935E+03 | 1.487E-02 | 6.559E+05 | 6.515E+03 | 1.228E+01 |
| **57L** | 6.167E-01 | 2.072E+02 | 3.015E-02 | 3.539E-01 | 2.814E-01 | 1.747E-04 | 6.104E+03 | 1.075E-04 | 3.799E+03 | 3.742E-02 | 1.173E+06 | 6.010E+03 | 1.263E+01 |
| **58L** | 5.978E-01 | 4.517E+02 | 2.620E-02 | 3.330E-01 | 2.336E-01 | 1.684E-04 | 6.501E+03 | 1.014E-04 | 3.918E+03 | 6.529E-02 | 3.307E+06 | 6.364E+03 | 1.832E+01 |
| **59L** | 5.926E-01 | 1.327E+03 | 3.000E-02 | 3.280E-01 | 1.987E-01 | 1.603E-04 | 6.603E+03 | 9.393E-05 | 3.975E+03 | 2.849E-01 | 6.408E+06 | 6.513E+03 | 2.532E+01 |
| **60L** | 6.135E-01 | 4.410E+02 | 3.660E-02 | 3.495E-01 | 2.578E-01 | 1.999E-04 | 5.321E+03 | 1.255E-04 | 3.225E+03 | 6.900E-02 | 2.843E+06 | 5.251E+03 | 1.505E+01 |
| **61L** | 6.433E-01 | 1.602E+02 | 2.447E-02 | 3.834E-01 | 3.415E-01 | 2.242E-04 | 5.138E+03 | 1.486E-04 | 3.264E+03 | 2.797E-02 | 9.304E+05 | 4.986E+03 | 8.573E+00 |
| **62L** | 6.324E-01 | 2.533E+01 | 3.052E-02 | 3.709E-01 | 3.791E-01 | 2.000E-04 | 5.371E+03 | 1.275E-04 | 3.396E+03 | 4.979E-03 | 1.351E+05 | 5.283E+03 | 6.955E+00 |
| **63L** | 6.166E-01 | 6.125E+01 | 3.209E-02 | 3.532E-01 | 3.302E-01 | 2.189E-04 | 4.888E+03 | 1.350E-04 | 3.037E+03 | 1.306E-02 | 2.924E+05 | 4.805E+03 | 9.172E+00 |
| **65L** | 6.029E-01 | 1.292E+02 | 2.628E-02 | 3.386E-01 | 2.881E-01 | 1.690E-04 | 6.412E+03 | 1.018E-04 | 3.889E+03 | 2.312E-02 | 7.620E+05 | 6.293E+03 | 1.205E+01 |
| **66L** | 6.331E-01 | 6.240E+02 | 2.809E-02 | 3.714E-01 | 2.418E-01 | 1.372E-04 | 7.857E+03 | 8.658E-05 | 5.071E+03 | 9.106E-02 | 4.298E+06 | 7.706E+03 | 1.710E+01 |
| **70L** | 6.756E-01 | 2.276E+01 | 3.679E-02 | 4.221E-01 | 4.111E-01 | 1.250E-04 | 8.454E+03 | 8.403E-05 | 5.799E+03 | 2.918E-03 | 1.801E+05 | 8.315E+03 | 5.915E+00 |
| **71L** | 5.936E-01 | 7.825E+02 | 2.778E-02 | 3.290E-01 | 2.591E-01 | 1.275E-04 | 8.258E+03 | 7.688E-05 | 4.841E+03 | 8.027E-02 | 7.675E+06 | 8.156E+03 | 1.490E+01 |
| **72L** | 6.225E-01 | 1.832E+02 | 3.522E-02 | 3.598E-01 | 2.741E-01 | 1.914E-04 | 5.530E+03 | 1.213E-04 | 3.398E+03 | 2.973E-02 | 1.148E+06 | 5.460E+03 | 1.331E+01 |
| **73L** | 6.795E-01 | 2.462E+01 | 2.784E-02 | 4.269E-01 | 4.222E-01 | 1.794E-04 | 6.037E+03 | 1.248E-04 | 4.038E+03 | 3.990E-03 | 1.547E+05 | 5.929E+03 | 5.608E+00 |
| **74L** | 6.261E-01 | 5.288E+03 | 3.336E-02 | 3.634E-01 | 1.687E-01 | 2.172E-04 | 4.928E+03 | 1.330E-04 | 3.182E+03 | 1.536E+00 | 1.837E+07 | 4.842E+03 | 3.515E+01 |
| **75L** | 6.263E-01 | 5.447E+01 | 2.637E-02 | 3.641E-01 | 3.361E-01 | 1.777E-04 | 6.164E+03 | 1.110E-04 | 3.875E+03 | 1.043E-02 | 3.023E+05 | 6.041E+03 | 8.850E+00 |
