# Supplementary material for: A simple model for glioma grading based on texture analysis applied to conventional brain MRI
Source: PLoS One. 2020 May 15;15(5):e0228972. doi: 10.1371/journal.pone.0228972 (PMC7228074; doi:10.1371/journal.pone.0228972)
Supplement: S8 Table — (DOCX) [file pone.0228972.s008.docx]

**T_2_^2^**

| **HGG** | ***F*_szm.sze_** | ***F*_szm.lze_** | ***F*_szm.glnu_** | ***F*_szm.zsnu_** | ***F*_szm.z.perc_** | ***F*_szm.lgze_** | ***F*_szm.hgze_** | ***F*_szm.szlge_** | ***F*_szm.szhge_** | ***F*_szm.lzlge_** | ***F*_szm.lzhge_** | ***F*_szm.gl.var_** | ***F*_szm.zs.var_** |
| --- | --- | --- | --- | --- | --- | --- | --- | --- | --- | --- | --- | --- | --- |
| **1H** | 7.040E-01 | 1.786E+01 | 1.417E-02 | 4.583E-01 | 4.815E-01 | 8.521E-05 | 1.326E+04 | 6.079E-05 | 9.213E+03 | 1.413E-03 | 2.615E+05 | 1.287E+04 | 4.313E+00 |
| **2H** | 7.139E-01 | 3.865E+01 | 1.479E-02 | 4.713E-01 | 4.539E-01 | 1.034E-04 | 1.134E+04 | 7.706E-05 | 7.824E+03 | 2.687E-03 | 5.838E+05 | 1.099E+04 | 4.854E+00 |
| **4H** | 6.628E-01 | 2.523E+01 | 2.296E-02 | 4.068E-01 | 4.123E-01 | 1.118E-04 | 9.607E+03 | 7.439E-05 | 6.397E+03 | 2.592E-03 | 2.532E+05 | 9.428E+03 | 5.884E+00 |
| **7H** | 7.113E-01 | 3.639E+02 | 1.382E-02 | 4.679E-01 | 3.874E-01 | 8.591E-05 | 1.334E+04 | 6.259E-05 | 9.278E+03 | 1.938E-02 | 7.282E+06 | 1.294E+04 | 6.663E+00 |
| **8H** | 7.737E-01 | 7.267E+00 | 1.142E-02 | 5.554E-01 | 6.129E-01 | 9.861E-05 | 1.285E+04 | 7.718E-05 | 9.929E+03 | 5.554E-04 | 1.175E+05 | 1.219E+04 | 2.662E+00 |
| **9H** | 7.586E-01 | 1.981E+01 | 1.008E-02 | 5.332E-01 | 5.627E-01 | 1.274E-04 | 1.613E+04 | 1.099E-04 | 1.213E+04 | 9.537E-04 | 4.840E+05 | 1.530E+04 | 3.159E+00 |
| **12H** | 6.849E-01 | 1.667E+01 | 1.837E-02 | 4.341E-01 | 4.688E-01 | 1.308E-04 | 1.028E+04 | 9.796E-05 | 6.975E+03 | 1.624E-03 | 1.840E+05 | 1.001E+04 | 4.550E+00 |
| **13H** | 7.247E-01 | 5.725E+00 | 1.607E-02 | 4.859E-01 | 5.802E-01 | 4.797E-04 | 1.070E+04 | 4.512E-04 | 7.652E+03 | 9.314E-04 | 6.598E+04 | 1.036E+04 | 2.970E+00 |
| **14H** | 7.587E-01 | 4.017E+00 | 1.462E-02 | 5.333E-01 | 6.414E-01 | 9.043E-05 | 1.254E+04 | 6.928E-05 | 9.431E+03 | 3.416E-04 | 5.315E+04 | 1.214E+04 | 2.429E+00 |
| **16H** | 6.710E-01 | 1.797E+01 | 1.945E-02 | 4.170E-01 | 4.519E-01 | 1.008E-04 | 1.074E+04 | 6.801E-05 | 7.182E+03 | 1.749E-03 | 1.994E+05 | 1.052E+04 | 4.896E+00 |
| **17H** | 7.083E-01 | 1.900E+01 | 1.441E-02 | 4.642E-01 | 4.907E-01 | 1.110E-04 | 1.085E+04 | 8.025E-05 | 7.644E+03 | 1.676E-03 | 2.349E+05 | 1.044E+04 | 4.152E+00 |
| **19H** | 7.348E-01 | 8.103E+00 | 1.050E-02 | 4.996E-01 | 5.647E-01 | 7.710E-05 | 1.567E+04 | 5.778E-05 | 1.125E+04 | 5.051E-04 | 1.594E+05 | 1.500E+04 | 3.135E+00 |
| **20H** | 6.941E-01 | 5.198E+01 | 1.446E-02 | 4.457E-01 | 4.399E-01 | 8.826E-05 | 1.330E+04 | 6.415E-05 | 8.956E+03 | 2.753E-03 | 1.036E+06 | 1.293E+04 | 5.167E+00 |
| **21H** | 7.543E-01 | 1.134E+01 | 1.013E-02 | 5.271E-01 | 5.743E-01 | 9.824E-05 | 1.330E+04 | 7.531E-05 | 9.899E+03 | 8.334E-04 | 1.912E+05 | 1.252E+04 | 3.031E+00 |
| **22H** | 7.419E-01 | 1.038E+01 | 1.280E-02 | 5.095E-01 | 5.585E-01 | 9.537E-05 | 1.279E+04 | 7.275E-05 | 9.320E+03 | 7.307E-04 | 1.732E+05 | 1.229E+04 | 3.206E+00 |
| **23H** | 6.452E-01 | 3.685E+02 | 3.000E-02 | 3.854E-01 | 2.821E-01 | 1.262E-04 | 8.303E+03 | 8.191E-05 | 5.351E+03 | 4.245E-02 | 3.296E+06 | 8.204E+03 | 1.257E+01 |
| **24H** | 7.497E-01 | 1.031E+02 | 1.134E-02 | 5.208E-01 | 4.516E-01 | 6.690E-05 | 1.739E+04 | 5.215E-05 | 1.254E+04 | 4.113E-03 | 2.651E+06 | 1.679E+04 | 4.903E+00 |
| **25H** | 7.693E-01 | 5.343E+00 | 1.751E-02 | 5.491E-01 | 6.149E-01 | 1.562E-04 | 9.595E+03 | 1.275E-04 | 7.467E+03 | 6.594E-04 | 4.979E+04 | 9.201E+03 | 2.644E+00 |
| **26H** | 7.357E-01 | 6.746E+01 | 1.288E-02 | 5.010E-01 | 4.801E-01 | 9.738E-05 | 1.216E+04 | 7.429E-05 | 8.671E+03 | 3.873E-03 | 1.212E+06 | 1.169E+04 | 4.338E+00 |
| **27H** | 7.454E-01 | 8.394E+00 | 1.615E-02 | 5.143E-01 | 5.684E-01 | 1.129E-04 | 1.018E+04 | 8.503E-05 | 7.580E+03 | 8.588E-04 | 8.919E+04 | 9.840E+03 | 3.095E+00 |
| **28H** | 6.944E-01 | 1.668E+01 | 1.794E-02 | 4.459E-01 | 4.744E-01 | 8.100E-05 | 1.344E+04 | 5.658E-05 | 9.360E+03 | 1.310E-03 | 2.199E+05 | 1.318E+04 | 4.443E+00 |
| **29H** | 7.726E-01 | 7.585E+00 | 1.159E-02 | 5.538E-01 | 6.109E-01 | 1.087E-04 | 1.166E+04 | 8.585E-05 | 8.891E+03 | 7.171E-04 | 9.684E+04 | 1.106E+04 | 2.679E+00 |
| **30H** | 7.051E-01 | 1.509E+02 | 2.380E-02 | 4.595E-01 | 4.121E-01 | 1.235E-04 | 8.961E+03 | 8.853E-05 | 6.308E+03 | 1.788E-02 | 1.284E+06 | 8.753E+03 | 5.888E+00 |
| **31H** | 6.818E-01 | 1.692E+02 | 1.671E-02 | 4.301E-01 | 3.518E-01 | 8.749E-05 | 1.257E+04 | 6.036E-05 | 8.479E+03 | 1.573E-02 | 1.883E+06 | 1.227E+04 | 8.081E+00 |
| **32H** | 6.864E-01 | 1.902E+01 | 1.939E-02 | 4.358E-01 | 4.569E-01 | 9.335E-05 | 1.160E+04 | 6.437E-05 | 7.929E+03 | 1.725E-03 | 2.214E+05 | 1.136E+04 | 4.789E+00 |
| **33H** | 7.646E-01 | 7.157E+00 | 1.485E-02 | 5.419E-01 | 6.107E-01 | 1.079E-04 | 1.082E+04 | 8.243E-05 | 8.361E+03 | 8.194E-04 | 7.000E+04 | 1.039E+04 | 2.681E+00 |
| **34H** | 7.558E-01 | 7.637E+00 | 1.129E-02 | 5.292E-01 | 5.870E-01 | 9.908E-05 | 1.262E+04 | 7.542E-05 | 9.454E+03 | 7.054E-04 | 1.034E+05 | 1.194E+04 | 2.902E+00 |
| **35H** | 7.543E-01 | 1.459E+01 | 1.269E-02 | 5.271E-01 | 5.587E-01 | 7.098E-05 | 1.616E+04 | 5.485E-05 | 1.199E+04 | 8.106E-04 | 2.751E+05 | 1.566E+04 | 3.204E+00 |
| **36H** | 7.244E-01 | 1.662E+02 | 1.548E-02 | 4.854E-01 | 4.149E-01 | 8.204E-05 | 1.359E+04 | 6.178E-05 | 9.494E+03 | 9.759E-03 | 2.860E+06 | 1.326E+04 | 5.808E+00 |
| **37H** | 7.388E-01 | 1.952E+01 | 1.267E-02 | 5.052E-01 | 5.379E-01 | 7.672E-05 | 1.496E+04 | 5.813E-05 | 1.083E+04 | 1.122E-03 | 3.563E+05 | 1.448E+04 | 3.456E+00 |
| **38H** | 7.379E-01 | 1.152E+01 | 1.558E-02 | 5.038E-01 | 5.532E-01 | 9.814E-05 | 1.193E+04 | 7.152E-05 | 9.020E+03 | 1.283E-03 | 1.101E+05 | 1.141E+04 | 3.268E+00 |
| **39H** | 7.389E-01 | 1.059E+01 | 1.571E-02 | 5.052E-01 | 5.456E-01 | 1.036E-04 | 1.121E+04 | 7.757E-05 | 8.255E+03 | 1.056E-03 | 1.200E+05 | 1.086E+04 | 3.359E+00 |
| **40H** | 7.644E-01 | 7.618E+00 | 1.116E-02 | 5.416E-01 | 6.136E-01 | 9.254E-05 | 1.332E+04 | 7.195E-05 | 1.003E+04 | 7.672E-04 | 9.830E+04 | 1.268E+04 | 2.656E+00 |
| **41H** | 6.941E-01 | 3.377E+01 | 2.266E-02 | 4.453E-01 | 4.333E-01 | 1.136E-04 | 9.423E+03 | 7.905E-05 | 6.546E+03 | 3.695E-03 | 3.183E+05 | 9.265E+03 | 5.327E+00 |
| **42H** | 7.448E-01 | 3.048E+01 | 1.431E-02 | 5.137E-01 | 5.043E-01 | 8.129E-05 | 1.381E+04 | 6.196E-05 | 1.005E+04 | 1.674E-03 | 5.792E+05 | 1.344E+04 | 3.933E+00 |
| **43H** | 6.949E-01 | 1.377E+01 | 2.313E-02 | 4.466E-01 | 4.830E-01 | 1.118E-04 | 9.611E+03 | 7.832E-05 | 6.638E+03 | 1.529E-03 | 1.308E+05 | 9.429E+03 | 4.285E+00 |
| **44H** | 6.718E-01 | 4.916E+01 | 1.942E-02 | 4.174E-01 | 3.886E-01 | 1.114E-04 | 9.803E+03 | 7.573E-05 | 6.499E+03 | 4.384E-03 | 6.167E+05 | 9.589E+03 | 6.621E+00 |
| **45H** | 7.259E-01 | 2.036E+01 | 1.571E-02 | 4.875E-01 | 4.966E-01 | 9.037E-05 | 1.228E+04 | 6.782E-05 | 8.610E+03 | 1.251E-03 | 3.508E+05 | 1.198E+04 | 4.055E+00 |
| **47H** | 7.529E-01 | 5.541E+00 | 1.273E-02 | 5.249E-01 | 6.115E-01 | 8.540E-05 | 1.376E+04 | 6.486E-05 | 1.027E+04 | 4.657E-04 | 7.975E+04 | 1.320E+04 | 2.674E+00 |
| **49H** | 7.408E-01 | 3.815E+01 | 1.255E-02 | 5.080E-01 | 5.238E-01 | 7.140E-05 | 1.601E+04 | 5.379E-05 | 1.165E+04 | 1.773E-03 | 8.612E+05 | 1.550E+04 | 3.645E+00 |
| **50H** | 6.723E-01 | 1.005E+02 | 2.663E-02 | 4.180E-01 | 3.821E-01 | 1.233E-04 | 8.580E+03 | 8.347E-05 | 5.760E+03 | 1.191E-02 | 8.732E+05 | 8.458E+03 | 6.848E+00 |
| **51H** | 7.110E-01 | 6.441E+01 | 1.633E-02 | 4.676E-01 | 4.442E-01 | 9.840E-05 | 1.163E+04 | 7.165E-05 | 8.125E+03 | 5.413E-03 | 8.728E+05 | 1.131E+04 | 5.068E+00 |
| **52H** | 7.068E-01 | 1.681E+01 | 2.485E-02 | 4.619E-01 | 4.657E-01 | 1.116E-04 | 9.611E+03 | 7.971E-05 | 6.790E+03 | 1.755E-03 | 1.663E+05 | 9.457E+03 | 4.610E+00 |
| **53H** | 7.516E-01 | 5.013E+01 | 1.102E-02 | 5.237E-01 | 4.688E-01 | 9.706E-05 | 1.672E+04 | 8.109E-05 | 1.214E+04 | 2.321E-03 | 1.134E+06 | 1.604E+04 | 4.550E+00 |
| **54H** | 7.278E-01 | 1.329E+02 | 1.307E-02 | 4.901E-01 | 4.378E-01 | 8.113E-05 | 1.433E+04 | 6.063E-05 | 1.015E+04 | 6.446E-03 | 2.808E+06 | 1.386E+04 | 5.217E+00 |
| **55H** | 7.670E-01 | 4.578E+00 | 1.429E-02 | 5.453E-01 | 6.352E-01 | 1.075E-04 | 1.122E+04 | 8.264E-05 | 8.705E+03 | 4.933E-04 | 4.848E+04 | 1.073E+04 | 2.478E+00 |
| **56H** | 7.231E-01 | 9.145E+00 | 1.638E-02 | 4.836E-01 | 5.410E-01 | 9.941E-05 | 1.124E+04 | 7.230E-05 | 8.072E+03 | 8.046E-04 | 1.159E+05 | 1.092E+04 | 3.416E+00 |
| **57H** | 6.533E-01 | 3.240E+02 | 1.779E-02 | 3.950E-01 | 3.024E-01 | 1.125E-04 | 1.010E+04 | 7.418E-05 | 6.633E+03 | 3.514E-02 | 3.045E+06 | 9.783E+03 | 1.094E+01 |
| **58H** | 6.963E-01 | 1.507E+01 | 1.738E-02 | 4.484E-01 | 4.842E-01 | 9.248E-05 | 1.197E+04 | 6.452E-05 | 8.382E+03 | 1.328E-03 | 1.860E+05 | 1.165E+04 | 4.265E+00 |
| **59H** | 7.051E-01 | 7.187E+01 | 1.529E-02 | 4.598E-01 | 4.348E-01 | 8.989E-05 | 1.239E+04 | 6.436E-05 | 8.591E+03 | 3.854E-03 | 1.393E+06 | 1.206E+04 | 5.290E+00 |
| **60H** | 7.174E-01 | 3.312E+02 | 1.455E-02 | 4.760E-01 | 4.125E-01 | 8.810E-05 | 1.340E+04 | 6.520E-05 | 9.507E+03 | 1.984E-02 | 5.579E+06 | 1.301E+04 | 5.876E+00 |
| **61H** | 7.366E-01 | 2.582E+01 | 1.347E-02 | 5.021E-01 | 5.035E-01 | 7.324E-05 | 1.576E+04 | 5.496E-05 | 1.147E+04 | 1.505E-03 | 4.727E+05 | 1.514E+04 | 3.944E+00 |
| **62H** | 7.423E-01 | 1.690E+01 | 1.528E-02 | 5.101E-01 | 5.303E-01 | 8.610E-05 | 1.288E+04 | 6.564E-05 | 9.292E+03 | 9.819E-04 | 3.136E+05 | 1.256E+04 | 3.556E+00 |
| **64H** | 6.799E-01 | 4.175E+01 | 2.611E-02 | 4.276E-01 | 4.018E-01 | 1.108E-04 | 9.640E+03 | 7.493E-05 | 6.667E+03 | 4.432E-03 | 3.993E+05 | 9.464E+03 | 6.193E+00 |
| **65H** | 7.534E-01 | 1.715E+01 | 1.649E-02 | 5.258E-01 | 5.613E-01 | 8.566E-05 | 1.278E+04 | 6.595E-05 | 9.437E+03 | 9.780E-04 | 3.213E+05 | 1.250E+04 | 3.174E+00 |
| **66H** | 6.663E-01 | 8.964E+01 | 2.959E-02 | 4.106E-01 | 3.618E-01 | 1.172E-04 | 8.922E+03 | 7.745E-05 | 6.029E+03 | 9.853E-03 | 8.230E+05 | 8.822E+03 | 7.641E+00 |
| **67H** | 7.008E-01 | 2.832E+01 | 1.616E-02 | 4.541E-01 | 4.583E-01 | 9.279E-05 | 1.202E+04 | 6.488E-05 | 8.465E+03 | 2.807E-03 | 3.036E+05 | 1.169E+04 | 4.761E+00 |
| **68H** | 6.956E-01 | 2.431E+01 | 2.420E-02 | 4.473E-01 | 4.411E-01 | 1.005E-04 | 1.053E+04 | 6.899E-05 | 7.460E+03 | 2.474E-03 | 2.433E+05 | 1.037E+04 | 5.140E+00 |
| **69H** | 7.431E-01 | 6.392E+01 | 1.198E-02 | 5.113E-01 | 4.812E-01 | 8.099E-05 | 1.444E+04 | 6.147E-05 | 1.047E+04 | 3.017E-03 | 1.404E+06 | 1.392E+04 | 4.319E+00 |
| **70H** | 7.391E-01 | 7.716E+00 | 1.708E-02 | 5.055E-01 | 5.657E-01 | 1.024E-04 | 1.215E+04 | 7.482E-05 | 9.235E+03 | 8.089E-04 | 8.302E+04 | 1.130E+04 | 3.124E+00 |
| **71H** | 7.331E-01 | 4.003E+01 | 1.469E-02 | 4.973E-01 | 4.625E-01 | 7.462E-05 | 1.483E+04 | 5.600E-05 | 1.063E+04 | 2.088E-03 | 8.066E+05 | 1.447E+04 | 4.674E+00 |
| **72H** | 7.066E-01 | 1.435E+01 | 1.786E-02 | 4.617E-01 | 4.961E-01 | 7.897E-05 | 1.372E+04 | 5.707E-05 | 9.496E+03 | 9.054E-04 | 2.458E+05 | 1.348E+04 | 4.063E+00 |
| **73H** | 7.303E-01 | 1.481E+02 | 1.362E-02 | 4.934E-01 | 4.386E-01 | 7.869E-05 | 1.438E+04 | 5.927E-05 | 1.019E+04 | 7.333E-03 | 3.041E+06 | 1.398E+04 | 5.199E+00 |
| **74H** | 6.029E-01 | 5.635E+02 | 3.715E-02 | 3.381E-01 | 2.304E-01 | 1.375E-04 | 7.545E+03 | 8.361E-05 | 4.523E+03 | 7.790E-02 | 4.113E+06 | 7.474E+03 | 1.884E+01 |
| **75H** | 6.815E-01 | 1.554E+02 | 1.711E-02 | 4.296E-01 | 3.667E-01 | 7.625E-05 | 1.417E+04 | 5.299E-05 | 9.483E+03 | 9.091E-03 | 2.777E+06 | 1.391E+04 | 7.437E+00 |
| **76H** | 7.519E-01 | 1.001E+01 | 1.994E-02 | 5.236E-01 | 5.857E-01 | 1.558E-04 | 7.327E+03 | 1.166E-04 | 5.541E+03 | 1.742E-03 | 6.275E+04 | 7.076E+03 | 2.915E+00 |
| **78H** | 7.559E-01 | 7.439E+00 | 1.718E-02 | 5.294E-01 | 5.827E-01 | 1.410E-04 | 8.402E+03 | 1.072E-04 | 6.389E+03 | 1.044E-03 | 5.874E+04 | 8.056E+03 | 2.944E+00 |
| **79H** | 7.268E-01 | 2.284E+01 | 2.269E-02 | 4.886E-01 | 4.813E-01 | 1.074E-04 | 1.004E+04 | 7.894E-05 | 7.281E+03 | 2.118E-03 | 2.545E+05 | 9.860E+03 | 4.317E+00 |
| **80H** | 6.786E-01 | 5.296E+01 | 2.258E-02 | 4.257E-01 | 3.853E-01 | 1.179E-04 | 9.142E+03 | 8.055E-05 | 6.201E+03 | 5.934E-03 | 4.860E+05 | 8.969E+03 | 6.735E+00 |
| **81H** | 7.912E-01 | 4.572E+00 | 1.558E-02 | 5.821E-01 | 6.578E-01 | 9.072E-05 | 1.266E+04 | 7.117E-05 | 1.022E+04 | 4.218E-04 | 5.332E+04 | 1.219E+04 | 2.310E+00 |
| **82H** | 7.245E-01 | 4.000E+01 | 1.671E-02 | 4.857E-01 | 4.382E-01 | 9.215E-05 | 1.211E+04 | 6.877E-05 | 8.558E+03 | 2.971E-03 | 5.618E+05 | 1.179E+04 | 5.208E+00 |
| **83H** | 7.420E-01 | 2.269E+01 | 2.437E-02 | 5.097E-01 | 5.102E-01 | 9.237E-05 | 1.145E+04 | 6.934E-05 | 8.455E+03 | 1.812E-03 | 2.879E+05 | 1.130E+04 | 3.841E+00 |
| **84H** | 7.905E-01 | 3.478E+00 | 1.443E-02 | 5.806E-01 | 6.812E-01 | 8.840E-05 | 1.315E+04 | 7.100E-05 | 1.028E+04 | 2.819E-04 | 4.861E+04 | 1.269E+04 | 2.154E+00 |
| **85H** | 7.419E-01 | 8.439E+00 | 1.260E-02 | 5.094E-01 | 5.600E-01 | 7.035E-05 | 1.618E+04 | 5.303E-05 | 1.178E+04 | 4.953E-04 | 1.669E+05 | 1.567E+04 | 3.188E+00 |
| **86H** | 7.450E-01 | 1.731E+01 | 1.499E-02 | 5.139E-01 | 5.465E-01 | 8.523E-05 | 1.307E+04 | 6.528E-05 | 9.473E+03 | 1.067E-03 | 2.991E+05 | 1.274E+04 | 3.348E+00 |
| **87H** | 7.458E-01 | 1.246E+01 | 1.265E-02 | 5.150E-01 | 5.475E-01 | 9.061E-05 | 1.309E+04 | 6.981E-05 | 9.508E+03 | 8.230E-04 | 2.059E+05 | 1.260E+04 | 3.335E+00 |
| **88H** | 7.765E-01 | 6.119E+00 | 1.150E-02 | 5.596E-01 | 6.232E-01 | 9.529E-05 | 1.303E+04 | 7.563E-05 | 9.924E+03 | 4.490E-04 | 9.826E+04 | 1.240E+04 | 2.575E+00 |
| **89H** | 7.731E-01 | 4.669E+00 | 1.419E-02 | 5.544E-01 | 6.396E-01 | 1.071E-04 | 1.112E+04 | 8.311E-05 | 8.604E+03 | 4.811E-04 | 5.217E+04 | 1.062E+04 | 2.444E+00 |
| **90H** | 7.353E-01 | 9.355E+00 | 2.048E-02 | 5.002E-01 | 5.613E-01 | 1.037E-04 | 1.047E+04 | 7.767E-05 | 7.600E+03 | 8.569E-04 | 1.069E+05 | 1.027E+04 | 3.174E+00 |
| **91H** | 6.910E-01 | 9.598E+01 | 9.175E-03 | 4.413E-01 | 3.889E-01 | 8.069E-05 | 1.674E+04 | 5.848E-05 | 1.131E+04 | 8.562E-03 | 1.275E+06 | 1.580E+04 | 6.613E+00 |
| **92H** | 7.536E-01 | 7.250E+00 | 1.554E-02 | 5.261E-01 | 5.868E-01 | 9.968E-05 | 1.138E+04 | 7.618E-05 | 8.478E+03 | 6.500E-04 | 8.963E+04 | 1.103E+04 | 2.904E+00 |
| **93H** | 7.546E-01 | 6.489E+00 | 1.494E-02 | 5.275E-01 | 5.942E-01 | 9.316E-05 | 1.210E+04 | 7.138E-05 | 8.983E+03 | 5.308E-04 | 8.958E+04 | 1.175E+04 | 2.831E+00 |
| **94H** | 7.736E-01 | 6.152E+00 | 1.422E-02 | 5.554E-01 | 6.187E-01 | 1.151E-04 | 1.031E+04 | 9.078E-05 | 7.839E+03 | 5.708E-04 | 7.890E+04 | 9.895E+03 | 2.612E+00 |
| **95H** | 7.417E-01 | 3.165E+01 | 1.640E-02 | 5.092E-01 | 5.186E-01 | 1.038E-04 | 1.136E+04 | 7.848E-05 | 8.409E+03 | 2.396E-03 | 4.351E+05 | 1.089E+04 | 3.718E+00 |
| **96H** | 7.475E-01 | 1.056E+01 | 1.394E-02 | 5.173E-01 | 5.563E-01 | 1.138E-04 | 1.037E+04 | 8.738E-05 | 7.505E+03 | 8.811E-04 | 1.450E+05 | 9.984E+03 | 3.231E+00 |
| **97H** | 7.807E-01 | 1.064E+01 | 1.054E-02 | 5.661E-01 | 6.035E-01 | 7.954E-05 | 1.549E+04 | 6.349E-05 | 1.179E+04 | 5.666E-04 | 2.277E+05 | 1.475E+04 | 2.746E+00 |
| **98H** | 7.138E-01 | 1.525E+01 | 1.734E-02 | 4.711E-01 | 4.842E-01 | 9.265E-05 | 1.195E+04 | 6.657E-05 | 8.583E+03 | 1.321E-03 | 1.855E+05 | 1.165E+04 | 4.265E+00 |
| **99H** | 8.357E-01 | 3.749E+00 | 1.478E-02 | 6.556E-01 | 7.155E-01 | 3.601E-04 | 7.674E+03 | 3.219E-04 | 6.410E+03 | 9.024E-04 | 2.785E+04 | 7.042E+03 | 1.953E+00 |
| **101H** | 7.958E-01 | 7.296E+00 | 1.425E-02 | 5.891E-01 | 6.543E-01 | 1.091E-04 | 1.125E+04 | 8.982E-05 | 8.746E+03 | 5.434E-04 | 1.093E+05 | 1.086E+04 | 2.336E+00 |
| **102H** | 8.227E-01 | 3.357E+00 | 1.392E-02 | 6.332E-01 | 7.065E-01 | 9.614E-05 | 1.267E+04 | 7.864E-05 | 1.062E+04 | 3.232E-04 | 3.956E+04 | 1.204E+04 | 2.002E+00 |
| **103H** | 7.692E-01 | 1.672E+01 | 1.135E-02 | 5.489E-01 | 5.802E-01 | 8.201E-05 | 1.497E+04 | 6.505E-05 | 1.122E+04 | 9.059E-04 | 3.274E+05 | 1.435E+04 | 2.970E+00 |
| **104H** | 6.934E-01 | 2.935E+01 | 1.729E-02 | 4.447E-01 | 4.479E-01 | 1.048E-04 | 1.066E+04 | 7.352E-05 | 7.331E+03 | 2.881E-03 | 3.181E+05 | 1.037E+04 | 4.985E+00 |
| **105H** | 7.087E-01 | 3.470E+01 | 1.726E-02 | 4.643E-01 | 4.533E-01 | 9.191E-05 | 1.225E+04 | 6.608E-05 | 8.659E+03 | 3.248E-03 | 3.884E+05 | 1.192E+04 | 4.867E+00 |
| **108H** | 7.082E-01 | 2.182E+01 | 2.206E-02 | 4.637E-01 | 4.538E-01 | 1.107E-04 | 1.013E+04 | 7.878E-05 | 7.325E+03 | 2.252E-03 | 2.172E+05 | 9.915E+03 | 4.855E+00 |
| **109H** | 6.762E-01 | 1.980E+01 | 2.054E-02 | 4.230E-01 | 4.361E-01 | 1.378E-04 | 8.166E+03 | 9.109E-05 | 5.691E+03 | 3.119E-03 | 1.352E+05 | 7.901E+03 | 5.258E+00 |
| **110H** | 6.514E-01 | 2.145E+01 | 2.239E-02 | 3.929E-01 | 4.118E-01 | 1.131E-04 | 9.484E+03 | 7.326E-05 | 6.243E+03 | 2.443E-03 | 1.956E+05 | 9.315E+03 | 5.897E+00 |
| **111H** | 7.295E-01 | 4.668E+01 | 8.183E-03 | 4.924E-01 | 5.049E-01 | 7.795E-05 | 1.718E+04 | 5.772E-05 | 1.216E+04 | 1.610E-03 | 1.584E+06 | 1.606E+04 | 3.922E+00 |
| **112H** | 6.791E-01 | 3.033E+01 | 2.097E-02 | 4.269E-01 | 4.509E-01 | 1.090E-04 | 9.924E+03 | 7.490E-05 | 6.688E+03 | 2.815E-03 | 3.373E+05 | 9.726E+03 | 4.919E+00 |
| **113H** | 7.376E-01 | 6.107E+00 | 1.942E-02 | 5.034E-01 | 5.830E-01 | 1.035E-04 | 1.075E+04 | 7.649E-05 | 7.947E+03 | 6.196E-04 | 6.541E+04 | 1.043E+04 | 2.941E+00 |
| **114H** | 7.568E-01 | 1.299E+01 | 9.505E-03 | 5.307E-01 | 5.599E-01 | 8.985E-05 | 1.442E+04 | 6.886E-05 | 1.066E+04 | 8.593E-04 | 2.447E+05 | 1.358E+04 | 3.190E+00 |
| **115H** | 7.279E-01 | 1.156E+01 | 1.381E-02 | 4.900E-01 | 5.438E-01 | 8.743E-05 | 1.301E+04 | 6.494E-05 | 9.282E+03 | 7.321E-04 | 2.014E+05 | 1.261E+04 | 3.382E+00 |
| **116H** | 7.115E-01 | 1.829E+01 | 1.879E-02 | 4.681E-01 | 4.900E-01 | 1.385E-04 | 8.953E+03 | 1.030E-04 | 6.290E+03 | 1.996E-03 | 1.819E+05 | 8.629E+03 | 4.164E+00 |
| **117H** | 7.786E-01 | 5.009E+00 | 8.210E-03 | 5.625E-01 | 6.481E-01 | 6.649E-05 | 1.949E+04 | 5.304E-05 | 1.466E+04 | 2.415E-04 | 1.411E+05 | 1.833E+04 | 2.381E+00 |
| **118H** | 8.003E-01 | 4.002E+00 | 1.500E-02 | 5.963E-01 | 6.766E-01 | 1.109E-04 | 1.062E+04 | 8.836E-05 | 8.590E+03 | 4.607E-04 | 3.904E+04 | 1.017E+04 | 2.184E+00 |
| **119H** | 7.374E-01 | 6.642E+00 | 1.288E-02 | 5.031E-01 | 5.806E-01 | 7.534E-05 | 1.507E+04 | 5.652E-05 | 1.092E+04 | 4.173E-04 | 1.199E+05 | 1.461E+04 | 2.966E+00 |
| **120H** | 7.204E-01 | 7.500E+00 | 1.748E-02 | 4.799E-01 | 5.509E-01 | 1.081E-04 | 1.030E+04 | 7.740E-05 | 7.515E+03 | 8.399E-04 | 7.146E+04 | 1.003E+04 | 3.294E+00 |
| **121H** | 6.107E-01 | 2.796E+01 | 2.148E-02 | 3.492E-01 | 3.898E-01 | 1.237E-04 | 8.821E+03 | 7.592E-05 | 5.352E+03 | 2.759E-03 | 3.265E+05 | 8.623E+03 | 6.580E+00 |
| **122H** | 7.501E-01 | 1.551E+01 | 1.190E-02 | 5.211E-01 | 5.609E-01 | 1.063E-04 | 1.154E+04 | 8.167E-05 | 8.363E+03 | 9.143E-04 | 3.075E+05 | 1.099E+04 | 3.179E+00 |
| **123H** | 6.869E-01 | 1.474E+01 | 2.594E-02 | 4.369E-01 | 4.882E-01 | 1.261E-04 | 8.478E+03 | 8.643E-05 | 5.866E+03 | 1.982E-03 | 1.130E+05 | 8.331E+03 | 4.195E+00 |
| **124H** | 7.176E-01 | 1.352E+01 | 9.091E-03 | 4.763E-01 | 5.244E-01 | 8.955E-05 | 1.507E+04 | 6.399E-05 | 1.080E+04 | 1.212E-03 | 2.422E+05 | 1.399E+04 | 3.636E+00 |
| **125H** | 7.432E-01 | 1.606E+01 | 1.453E-02 | 5.112E-01 | 5.582E-01 | 8.013E-05 | 1.391E+04 | 6.059E-05 | 1.015E+04 | 8.790E-04 | 3.150E+05 | 1.354E+04 | 3.210E+00 |
| **126H** | 7.688E-01 | 6.663E+00 | 1.064E-02 | 5.481E-01 | 6.145E-01 | 7.310E-05 | 1.632E+04 | 5.726E-05 | 1.223E+04 | 3.725E-04 | 1.426E+05 | 1.565E+04 | 2.648E+00 |
| **127H** | 7.431E-01 | 1.600E+01 | 9.781E-03 | 5.112E-01 | 5.464E-01 | 5.311E-05 | 2.205E+04 | 4.050E-05 | 1.592E+04 | 5.421E-04 | 5.227E+05 | 2.125E+04 | 3.349E+00 |
| **128H** | 6.577E-01 | 5.509E+01 | 2.262E-02 | 4.005E-01 | 3.841E-01 | 1.220E-04 | 8.844E+03 | 8.047E-05 | 5.825E+03 | 5.569E-03 | 5.769E+05 | 8.671E+03 | 6.777E+00 |
| **129H** | 7.003E-01 | 3.110E+01 | 1.529E-02 | 4.533E-01 | 4.367E-01 | 1.320E-04 | 8.934E+03 | 9.229E-05 | 6.307E+03 | 4.190E-03 | 2.659E+05 | 8.571E+03 | 5.244E+00 |
| **130H** | 7.286E-01 | 3.990E+01 | 1.742E-02 | 4.912E-01 | 4.806E-01 | 1.170E-04 | 9.778E+03 | 8.268E-05 | 7.397E+03 | 5.824E-03 | 2.812E+05 | 9.420E+03 | 4.329E+00 |
| **131H** | 7.200E-01 | 8.132E+00 | 1.477E-02 | 4.795E-01 | 5.475E-01 | 8.963E-05 | 1.268E+04 | 6.407E-05 | 9.264E+03 | 7.190E-04 | 9.956E+04 | 1.225E+04 | 3.336E+00 |
| **132H** | 7.346E-01 | 1.256E+01 | 1.706E-02 | 4.992E-01 | 5.430E-01 | 9.806E-05 | 1.132E+04 | 7.099E-05 | 8.484E+03 | 1.520E-03 | 1.120E+05 | 1.102E+04 | 3.391E+00 |
| **133H** | 7.387E-01 | 9.146E+00 | 1.184E-02 | 5.049E-01 | 5.614E-01 | 6.948E-05 | 1.649E+04 | 5.260E-05 | 1.189E+04 | 4.916E-04 | 1.905E+05 | 1.596E+04 | 3.172E+00 |
| **134H** | 7.016E-01 | 1.583E+01 | 1.526E-02 | 4.552E-01 | 4.953E-01 | 1.001E-04 | 1.128E+04 | 7.088E-05 | 7.834E+03 | 1.243E-03 | 2.222E+05 | 1.094E+04 | 4.077E+00 |
| **135H** | 7.154E-01 | 4.112E+02 | 1.601E-02 | 4.734E-01 | 3.823E-01 | 1.188E-04 | 9.609E+03 | 8.737E-05 | 6.679E+03 | 3.025E-02 | 5.680E+06 | 9.317E+03 | 6.842E+00 |
| **136H** | 7.319E-01 | 6.953E+00 | 1.154E-02 | 4.956E-01 | 5.677E-01 | 9.217E-05 | 1.308E+04 | 6.779E-05 | 9.481E+03 | 5.683E-04 | 1.042E+05 | 1.249E+04 | 3.103E+00 |
| **137H** | 7.266E-01 | 7.364E+00 | 1.389E-02 | 4.884E-01 | 5.582E-01 | 1.285E-04 | 9.711E+03 | 9.319E-05 | 7.158E+03 | 9.553E-04 | 6.555E+04 | 9.189E+03 | 3.209E+00 |
| **138H** | 7.410E-01 | 5.412E+00 | 1.433E-02 | 5.083E-01 | 5.975E-01 | 1.307E-04 | 9.350E+03 | 9.789E-05 | 6.896E+03 | 6.775E-04 | 5.114E+04 | 8.934E+03 | 2.800E+00 |
| **139H** | 6.852E-01 | 4.045E+01 | 1.915E-02 | 4.341E-01 | 4.105E-01 | 9.913E-05 | 1.097E+04 | 6.914E-05 | 7.426E+03 | 3.288E-03 | 5.131E+05 | 1.074E+04 | 5.933E+00 |
| **140H** | 6.712E-01 | 6.004E+01 | 1.975E-02 | 4.166E-01 | 3.891E-01 | 1.051E-04 | 1.039E+04 | 7.018E-05 | 7.028E+03 | 7.868E-03 | 4.845E+05 | 1.015E+04 | 6.606E+00 |
| **141H** | 7.525E-01 | 1.395E+01 | 8.122E-03 | 5.244E-01 | 5.773E-01 | 6.763E-05 | 1.947E+04 | 5.168E-05 | 1.421E+04 | 5.242E-04 | 4.793E+05 | 1.825E+04 | 3.000E+00 |
| **142H** | 6.567E-01 | 4.481E+01 | 2.027E-02 | 3.996E-01 | 3.874E-01 | 1.155E-04 | 9.396E+03 | 7.756E-05 | 6.038E+03 | 3.814E-03 | 5.476E+05 | 9.211E+03 | 6.662E+00 |
| **143H** | 6.393E-01 | 1.165E+02 | 2.262E-02 | 3.789E-01 | 3.352E-01 | 1.074E-04 | 9.934E+03 | 6.920E-05 | 6.313E+03 | 1.028E-02 | 1.355E+06 | 9.771E+03 | 8.900E+00 |
| **144H** | 6.830E-01 | 1.457E+01 | 2.246E-02 | 4.313E-01 | 4.637E-01 | 1.085E-04 | 9.896E+03 | 7.466E-05 | 6.763E+03 | 1.565E-03 | 1.400E+05 | 9.718E+03 | 4.650E+00 |
| **145H** | 7.408E-01 | 1.276E+01 | 1.123E-02 | 5.080E-01 | 5.503E-01 | 7.087E-05 | 1.648E+04 | 5.401E-05 | 1.182E+04 | 5.918E-04 | 3.086E+05 | 1.587E+04 | 3.302E+00 |
| **146H** | 6.991E-01 | 1.390E+01 | 1.261E-02 | 4.522E-01 | 5.007E-01 | 8.350E-05 | 1.386E+04 | 5.937E-05 | 9.497E+03 | 9.159E-04 | 2.421E+05 | 1.337E+04 | 3.989E+00 |
| **147H** | 7.353E-01 | 6.750E+00 | 1.095E-02 | 5.002E-01 | 5.771E-01 | 9.764E-05 | 1.279E+04 | 7.146E-05 | 9.377E+03 | 6.699E-04 | 9.243E+04 | 1.211E+04 | 3.003E+00 |
| **148H** | 7.226E-01 | 3.770E+01 | 1.522E-02 | 4.830E-01 | 4.753E-01 | 9.743E-05 | 1.155E+04 | 7.238E-05 | 8.096E+03 | 2.277E-03 | 6.512E+05 | 1.123E+04 | 4.427E+00 |
| **149H** | 7.456E-01 | 7.413E+00 | 1.550E-02 | 5.146E-01 | 5.769E-01 | 8.598E-05 | 1.302E+04 | 6.462E-05 | 9.625E+03 | 5.695E-04 | 1.096E+05 | 1.264E+04 | 3.004E+00 |
| **150H** | 6.349E-01 | 1.111E+02 | 2.397E-02 | 3.738E-01 | 3.320E-01 | 1.233E-04 | 8.772E+03 | 7.782E-05 | 5.650E+03 | 1.406E-02 | 9.038E+05 | 8.590E+03 | 9.073E+00 |
| **151H** | 7.928E-01 | 4.045E+00 | 1.686E-02 | 5.844E-01 | 6.684E-01 | 1.293E-04 | 9.207E+03 | 1.021E-04 | 7.419E+03 | 5.103E-04 | 3.553E+04 | 8.762E+03 | 2.237E+00 |
| **152H** | 7.007E-01 | 1.548E+01 | 1.136E-02 | 4.541E-01 | 4.943E-01 | 8.911E-05 | 1.356E+04 | 6.358E-05 | 9.286E+03 | 1.046E-03 | 2.752E+05 | 1.298E+04 | 4.093E+00 |
| **153H** | 6.955E-01 | 1.898E+01 | 1.381E-02 | 4.473E-01 | 4.760E-01 | 2.016E-04 | 8.352E+03 | 1.490E-04 | 5.724E+03 | 2.517E-03 | 1.791E+05 | 7.845E+03 | 4.413E+00 |
| **154H** | 7.415E-01 | 5.172E+00 | 1.541E-02 | 5.088E-01 | 6.021E-01 | 1.143E-04 | 1.097E+04 | 8.358E-05 | 8.259E+03 | 6.595E-04 | 4.877E+04 | 1.026E+04 | 2.757E+00 |
| **155H** | 6.500E-01 | 7.555E+01 | 1.717E-02 | 3.923E-01 | 3.714E-01 | 1.266E-04 | 9.040E+03 | 8.175E-05 | 5.915E+03 | 1.213E-02 | 4.957E+05 | 8.732E+03 | 7.248E+00 |
| **156H** | 6.256E-01 | 2.254E+02 | 2.806E-02 | 3.628E-01 | 2.480E-01 | 1.414E-04 | 7.551E+03 | 8.772E-05 | 4.819E+03 | 2.981E-02 | 1.748E+06 | 7.417E+03 | 1.627E+01 |
| **157H** | 6.506E-01 | 1.362E+02 | 2.167E-02 | 3.923E-01 | 3.456E-01 | 1.276E-04 | 8.527E+03 | 8.422E-05 | 5.498E+03 | 1.274E-02 | 1.497E+06 | 8.354E+03 | 8.373E+00 |
| **158H** | 7.819E-01 | 3.610E+01 | 9.642E-03 | 5.681E-01 | 5.532E-01 | 7.827E-05 | 1.622E+04 | 6.252E-05 | 1.220E+04 | 1.368E-03 | 1.036E+06 | 1.535E+04 | 3.268E+00 |
| **160H** | 7.144E-01 | 7.604E+00 | 1.507E-02 | 4.721E-01 | 5.460E-01 | 9.508E-05 | 1.194E+04 | 6.751E-05 | 8.615E+03 | 7.517E-04 | 8.545E+04 | 1.155E+04 | 3.353E+00 |
| **161H** | 7.224E-01 | 8.999E+00 | 2.004E-02 | 4.826E-01 | 5.402E-01 | 1.088E-04 | 1.001E+04 | 7.953E-05 | 7.178E+03 | 8.753E-04 | 9.842E+04 | 9.794E+03 | 3.426E+00 |
| **162H** | 7.249E-01 | 5.757E+00 | 2.019E-02 | 4.863E-01 | 5.806E-01 | 1.025E-04 | 1.072E+04 | 7.339E-05 | 7.882E+03 | 6.179E-04 | 5.876E+04 | 1.044E+04 | 2.966E+00 |
| **163H** | 7.570E-01 | 4.865E+00 | 1.162E-02 | 5.308E-01 | 6.259E-01 | 8.246E-05 | 1.442E+04 | 6.334E-05 | 1.078E+04 | 3.570E-04 | 7.745E+04 | 1.380E+04 | 2.552E+00 |
| **164H** | 7.056E-01 | 9.372E+00 | 2.149E-02 | 4.603E-01 | 5.142E-01 | 1.236E-04 | 8.813E+03 | 8.636E-05 | 6.330E+03 | 1.182E-03 | 7.824E+04 | 8.617E+03 | 3.781E+00 |
| **165H** | 6.268E-01 | 2.406E+02 | 1.787E-02 | 3.653E-01 | 3.086E-01 | 1.123E-04 | 9.996E+03 | 7.010E-05 | 6.272E+03 | 3.269E-02 | 1.872E+06 | 9.699E+03 | 1.050E+01 |
| **166H** | 6.789E-01 | 1.237E+02 | 2.088E-02 | 4.265E-01 | 3.939E-01 | 1.121E-04 | 9.649E+03 | 7.755E-05 | 6.458E+03 | 9.698E-03 | 1.606E+06 | 9.459E+03 | 6.446E+00 |
| **167H** | 7.053E-01 | 6.782E+01 | 1.675E-02 | 4.600E-01 | 4.437E-01 | 9.967E-05 | 1.116E+04 | 7.071E-05 | 7.832E+03 | 5.150E-03 | 9.331E+05 | 1.086E+04 | 5.080E+00 |
| **168H** | 7.354E-01 | 7.111E+00 | 1.256E-02 | 5.004E-01 | 5.818E-01 | 1.001E-04 | 1.212E+04 | 7.475E-05 | 8.730E+03 | 5.493E-04 | 1.140E+05 | 1.154E+04 | 2.954E+00 |
| **169H** | 7.975E-01 | 3.620E+00 | 1.254E-02 | 5.917E-01 | 6.835E-01 | 1.104E-04 | 1.111E+04 | 8.792E-05 | 8.868E+03 | 3.766E-04 | 4.314E+04 | 1.056E+04 | 2.139E+00 |
| **170H** | 6.951E-01 | 1.521E+01 | 2.133E-02 | 4.466E-01 | 4.783E-01 | 1.077E-04 | 1.003E+04 | 7.456E-05 | 7.057E+03 | 1.627E-03 | 1.476E+05 | 9.832E+03 | 4.370E+00 |
| **171H** | 7.137E-01 | 1.574E+01 | 1.291E-02 | 4.714E-01 | 5.287E-01 | 7.316E-05 | 1.543E+04 | 5.315E-05 | 1.081E+04 | 7.833E-04 | 3.491E+05 | 1.498E+04 | 3.578E+00 |
| **172H** | 7.673E-01 | 3.543E+01 | 1.028E-02 | 5.459E-01 | 5.578E-01 | 7.243E-05 | 1.673E+04 | 5.699E-05 | 1.245E+04 | 1.377E-03 | 9.612E+05 | 1.602E+04 | 3.214E+00 |
| **173H** | 7.377E-01 | 6.154E+00 | 1.051E-02 | 5.035E-01 | 5.840E-01 | 9.074E-05 | 1.387E+04 | 6.670E-05 | 1.021E+04 | 5.328E-04 | 9.366E+04 | 1.308E+04 | 2.931E+00 |
| **174H** | 7.562E-01 | 2.358E+01 | 9.424E-03 | 5.299E-01 | 5.495E-01 | 8.004E-05 | 1.590E+04 | 6.139E-05 | 1.177E+04 | 1.119E-03 | 5.533E+05 | 1.501E+04 | 3.312E+00 |
| **175H** | 7.186E-01 | 2.531E+01 | 1.084E-02 | 4.777E-01 | 4.977E-01 | 9.659E-05 | 1.297E+04 | 6.985E-05 | 9.213E+03 | 2.446E-03 | 3.770E+05 | 1.228E+04 | 4.038E+00 |
| **176H** | 7.696E-01 | 5.186E+00 | 1.059E-02 | 5.492E-01 | 6.302E-01 | 8.258E-05 | 1.480E+04 | 6.514E-05 | 1.111E+04 | 3.406E-04 | 9.492E+04 | 1.411E+04 | 2.518E+00 |
| **177H** | 7.360E-01 | 3.483E+01 | 1.099E-02 | 5.012E-01 | 5.114E-01 | 8.555E-05 | 1.418E+04 | 6.409E-05 | 1.020E+04 | 1.775E-03 | 7.336E+05 | 1.353E+04 | 3.823E+00 |
| **178H** | 6.648E-01 | 7.585E+01 | 2.710E-02 | 4.088E-01 | 3.714E-01 | 1.582E-04 | 6.901E+03 | 1.036E-04 | 4.716E+03 | 1.261E-02 | 4.625E+05 | 6.729E+03 | 7.251E+00 |
| **181H** | 6.805E-01 | 1.908E+01 | 1.219E-02 | 4.284E-01 | 4.598E-01 | 9.315E-05 | 1.275E+04 | 6.377E-05 | 8.614E+03 | 1.767E-03 | 2.548E+05 | 1.222E+04 | 4.729E+00 |
| **182H** | 6.956E-01 | 2.932E+01 | 1.283E-02 | 4.475E-01 | 4.640E-01 | 8.945E-05 | 1.304E+04 | 6.251E-05 | 9.037E+03 | 2.459E-03 | 3.888E+05 | 1.255E+04 | 4.645E+00 |
| **183H** | 7.210E-01 | 8.492E+00 | 1.148E-02 | 4.808E-01 | 5.459E-01 | 9.304E-05 | 1.329E+04 | 6.712E-05 | 9.564E+03 | 7.939E-04 | 1.150E+05 | 1.257E+04 | 3.356E+00 |
| **184H** | 7.035E-01 | 1.147E+01 | 1.640E-02 | 4.578E-01 | 5.155E-01 | 1.039E-04 | 1.081E+04 | 7.385E-05 | 7.579E+03 | 9.774E-04 | 1.484E+05 | 1.051E+04 | 3.762E+00 |
| **185H** | 6.486E-01 | 2.883E+01 | 2.455E-02 | 3.892E-01 | 3.791E-01 | 1.246E-04 | 8.560E+03 | 8.058E-05 | 5.595E+03 | 3.597E-03 | 2.447E+05 | 8.418E+03 | 6.956E+00 |
| **186H** | 6.729E-01 | 1.962E+01 | 2.613E-02 | 4.187E-01 | 4.377E-01 | 1.439E-04 | 7.468E+03 | 9.566E-05 | 5.116E+03 | 3.088E-03 | 1.304E+05 | 7.325E+03 | 5.220E+00 |
| **187H** | 7.841E-01 | 3.893E+00 | 1.086E-02 | 5.709E-01 | 6.647E-01 | 9.204E-05 | 1.342E+04 | 7.324E-05 | 1.039E+04 | 3.139E-04 | 5.934E+04 | 1.276E+04 | 2.263E+00 |
| **188H** | 6.539E-01 | 2.765E+01 | 1.930E-02 | 3.960E-01 | 4.127E-01 | 1.148E-04 | 9.602E+03 | 7.469E-05 | 6.318E+03 | 3.356E-03 | 2.390E+05 | 9.360E+03 | 5.872E+00 |
| **189H** | 7.411E-01 | 6.448E+00 | 1.124E-02 | 5.082E-01 | 5.882E-01 | 8.523E-05 | 1.412E+04 | 6.327E-05 | 1.044E+04 | 4.841E-04 | 1.060E+05 | 1.348E+04 | 2.890E+00 |
| **190H** | 7.747E-01 | 5.698E+00 | 1.275E-02 | 5.568E-01 | 6.279E-01 | 7.778E-05 | 1.477E+04 | 6.097E-05 | 1.128E+04 | 3.739E-04 | 1.007E+05 | 1.426E+04 | 2.536E+00 |
| **191H** | 7.202E-01 | 1.057E+01 | 1.285E-02 | 4.797E-01 | 5.298E-01 | 1.058E-04 | 1.139E+04 | 7.649E-05 | 8.158E+03 | 1.030E-03 | 1.362E+05 | 1.087E+04 | 3.562E+00 |
| **192H** | 6.161E-01 | 4.815E+01 | 2.486E-02 | 3.532E-01 | 3.438E-01 | 1.105E-04 | 9.614E+03 | 6.755E-05 | 5.992E+03 | 5.540E-03 | 4.286E+05 | 9.461E+03 | 8.460E+00 |
| **193H** | 8.174E-01 | 3.985E+00 | 9.620E-03 | 6.237E-01 | 6.980E-01 | 9.785E-05 | 1.365E+04 | 7.739E-05 | 1.154E+04 | 4.740E-04 | 4.106E+04 | 1.270E+04 | 2.052E+00 |
| **194H** | 6.515E-01 | 4.150E+01 | 2.163E-02 | 3.930E-01 | 3.729E-01 | 1.115E-04 | 9.698E+03 | 7.219E-05 | 6.404E+03 | 4.846E-03 | 3.651E+05 | 9.499E+03 | 7.192E+00 |
| **195H** | 7.308E-01 | 2.214E+01 | 9.077E-03 | 4.941E-01 | 5.168E-01 | 7.457E-05 | 1.703E+04 | 5.524E-05 | 1.206E+04 | 9.308E-04 | 6.428E+05 | 1.613E+04 | 3.744E+00 |
| **196H** | 7.470E-01 | 1.797E+01 | 1.392E-02 | 5.167E-01 | 5.505E-01 | 8.962E-05 | 1.298E+04 | 6.618E-05 | 9.805E+03 | 2.185E-03 | 1.654E+05 | 1.246E+04 | 3.299E+00 |
| **197H** | 7.062E-01 | 1.996E+01 | 1.075E-02 | 4.613E-01 | 4.879E-01 | 1.120E-04 | 1.158E+04 | 7.999E-05 | 8.038E+03 | 1.608E-03 | 3.126E+05 | 1.091E+04 | 4.201E+00 |
| **198H** | 7.667E-01 | 7.444E+00 | 8.837E-03 | 5.449E-01 | 6.200E-01 | 6.727E-05 | 1.865E+04 | 5.247E-05 | 1.403E+04 | 3.559E-04 | 1.891E+05 | 1.769E+04 | 2.601E+00 |
| **199H** | 7.179E-01 | 1.889E+01 | 1.686E-02 | 4.766E-01 | 4.939E-01 | 9.534E-05 | 1.164E+04 | 6.851E-05 | 8.410E+03 | 1.620E-03 | 2.364E+05 | 1.133E+04 | 4.098E+00 |
| **200H** | 7.995E-01 | 3.816E+00 | 9.843E-03 | 5.948E-01 | 6.803E-01 | 5.795E-05 | 2.050E+04 | 4.772E-05 | 1.597E+04 | 1.826E-04 | 9.113E+04 | 1.971E+04 | 2.160E+00 |
| **201H** | 7.398E-01 | 8.477E+00 | 1.154E-02 | 5.065E-01 | 5.793E-01 | 9.210E-05 | 1.341E+04 | 6.974E-05 | 9.726E+03 | 5.477E-04 | 1.680E+05 | 1.281E+04 | 2.980E+00 |
| **202H** | 7.393E-01 | 5.973E+00 | 1.203E-02 | 5.058E-01 | 5.883E-01 | 8.343E-05 | 1.414E+04 | 6.230E-05 | 1.030E+04 | 4.398E-04 | 9.963E+04 | 1.362E+04 | 2.889E+00 |
| **203H** | 7.680E-01 | 6.428E+00 | 1.134E-02 | 5.469E-01 | 6.145E-01 | 7.543E-05 | 1.568E+04 | 5.926E-05 | 1.179E+04 | 3.835E-04 | 1.218E+05 | 1.509E+04 | 2.648E+00 |
| **206H** | 7.275E-01 | 8.071E+01 | 1.543E-02 | 4.896E-01 | 4.724E-01 | 9.551E-05 | 1.178E+04 | 7.047E-05 | 8.452E+03 | 4.965E-03 | 1.339E+06 | 1.143E+04 | 4.480E+00 |
| **207H** | 6.307E-01 | 8.997E+01 | 2.417E-02 | 3.694E-01 | 3.242E-01 | 1.138E-04 | 9.356E+03 | 7.223E-05 | 5.887E+03 | 8.810E-03 | 9.371E+05 | 9.206E+03 | 9.516E+00 |
| **208H** | 6.127E-01 | 1.294E+03 | 2.823E-02 | 3.483E-01 | 1.921E-01 | 1.369E-04 | 7.779E+03 | 8.368E-05 | 4.821E+03 | 1.768E-01 | 9.601E+06 | 7.650E+03 | 2.709E+01 |
| **209H** | 6.510E-01 | 2.818E+02 | 2.550E-02 | 3.928E-01 | 2.684E-01 | 1.620E-04 | 6.675E+03 | 1.060E-04 | 4.363E+03 | 4.331E-02 | 1.862E+06 | 6.547E+03 | 1.388E+01 |
| **210H** | 6.502E-01 | 1.029E+02 | 2.502E-02 | 3.923E-01 | 3.481E-01 | 1.258E-04 | 8.448E+03 | 8.290E-05 | 5.424E+03 | 1.114E-02 | 9.908E+05 | 8.317E+03 | 8.250E+00 |

| **LGG** | ***F*_szm.sze_** | ***F*_szm.lze_** | ***F*_szm.glnu_** | ***F*_szm.zsnu_** | ***F*_szm.z.perc_** | ***F*_szm.lgze_** | ***F*_szm.hgze_** | ***F*_szm.szlge_** | ***F*_szm.szhge_** | ***F*_szm.lzlge_** | ***F*_szm.lzhge_** | ***F*_szm.gl.var_** | ***F*_szm.zs.var_** |
| --- | --- | --- | --- | --- | --- | --- | --- | --- | --- | --- | --- | --- | --- |
| **1L** | 7.148E-01 | 8.462E+00 | 1.857E-02 | 4.724E-01 | 5.350E-01 | 4.236E-04 | 8.005E+03 | 2.544E-04 | 5.670E+03 | 1.873E-03 | 7.308E+04 | 7.754E+03 | 3.494E+00 |
| **3L** | 7.228E-01 | 9.184E+00 | 1.056E-02 | 4.833E-01 | 5.446E-01 | 8.041E-05 | 1.516E+04 | 5.836E-05 | 1.090E+04 | 7.337E-04 | 1.416E+05 | 1.445E+04 | 3.371E+00 |
| **4L** | 7.149E-01 | 9.117E+00 | 1.643E-02 | 4.726E-01 | 5.348E-01 | 1.077E-04 | 1.045E+04 | 7.809E-05 | 7.380E+03 | 8.246E-04 | 1.120E+05 | 1.015E+04 | 3.496E+00 |
| **5L** | 8.668E-01 | 2.026E+00 | 1.004E-02 | 7.086E-01 | 8.024E-01 | 6.667E-05 | 1.804E+04 | 5.773E-05 | 1.569E+04 | 1.332E-04 | 3.679E+04 | 1.727E+04 | 1.552E+00 |
| **7L** | 7.571E-01 | 5.117E+00 | 1.237E-02 | 5.309E-01 | 6.153E-01 | 9.622E-05 | 1.332E+04 | 7.461E-05 | 1.012E+04 | 4.766E-04 | 6.350E+04 | 1.276E+04 | 2.641E+00 |
| **9L** | 8.011E-01 | 3.675E+00 | 1.099E-02 | 5.975E-01 | 6.815E-01 | 9.268E-05 | 1.550E+04 | 7.706E-05 | 1.222E+04 | 3.058E-04 | 5.904E+04 | 1.477E+04 | 2.152E+00 |
| **11L** | 6.111E-01 | 7.306E+02 | 3.749E-02 | 3.468E-01 | 1.998E-01 | 1.519E-04 | 6.820E+03 | 9.234E-05 | 4.214E+03 | 1.157E-01 | 4.635E+06 | 6.759E+03 | 2.506E+01 |
| **12L** | 6.113E-01 | 4.816E+02 | 2.251E-02 | 3.472E-01 | 2.511E-01 | 1.298E-04 | 8.327E+03 | 7.878E-05 | 5.145E+03 | 6.850E-02 | 3.736E+06 | 8.164E+03 | 1.586E+01 |
| **13L** | 7.500E-01 | 5.751E+00 | 9.206E-03 | 5.208E-01 | 6.107E-01 | 7.431E-05 | 1.695E+04 | 5.608E-05 | 1.259E+04 | 3.695E-04 | 1.182E+05 | 1.605E+04 | 2.681E+00 |
| **14L** | 6.652E-01 | 1.232E+01 | 2.072E-02 | 4.101E-01 | 4.653E-01 | 1.001E-04 | 1.069E+04 | 6.733E-05 | 7.037E+03 | 1.165E-03 | 1.387E+05 | 1.050E+04 | 4.617E+00 |
| **15L** | 7.856E-01 | 3.659E+00 | 1.704E-02 | 5.732E-01 | 6.694E-01 | 1.196E-04 | 9.725E+03 | 9.369E-05 | 7.770E+03 | 4.424E-04 | 3.336E+04 | 9.330E+03 | 2.231E+00 |
| **16L** | 7.371E-01 | 7.675E+00 | 8.620E-03 | 5.028E-01 | 5.634E-01 | 5.170E-05 | 2.356E+04 | 3.931E-05 | 1.684E+04 | 3.236E-04 | 2.184E+05 | 2.255E+04 | 3.150E+00 |
| **17L** | 6.890E-01 | 1.272E+01 | 1.340E-02 | 4.391E-01 | 4.874E-01 | 9.785E-05 | 1.187E+04 | 6.772E-05 | 8.154E+03 | 1.203E-03 | 1.556E+05 | 1.144E+04 | 4.210E+00 |
| **18L** | 6.802E-01 | 8.884E+01 | 1.893E-02 | 4.276E-01 | 3.671E-01 | 1.097E-04 | 1.029E+04 | 7.301E-05 | 7.280E+03 | 1.010E-02 | 7.930E+05 | 9.964E+03 | 7.420E+00 |
| **19L** | 7.163E-01 | 8.615E+00 | 1.861E-02 | 4.744E-01 | 5.331E-01 | 1.273E-04 | 8.768E+03 | 9.073E-05 | 6.346E+03 | 1.104E-03 | 7.364E+04 | 8.529E+03 | 3.517E+00 |
| **20L** | 7.402E-01 | 9.529E+00 | 1.336E-02 | 5.071E-01 | 5.694E-01 | 1.286E-04 | 9.536E+03 | 9.372E-05 | 7.124E+03 | 1.583E-03 | 7.032E+04 | 9.059E+03 | 3.084E+00 |
| **21L** | 7.936E-01 | 6.641E+00 | 1.450E-02 | 5.856E-01 | 6.477E-01 | 7.081E-05 | 1.568E+04 | 5.544E-05 | 1.266E+04 | 5.233E-04 | 8.858E+04 | 1.526E+04 | 2.383E+00 |
| **22L** | 5.902E-01 | 9.798E+02 | 2.170E-02 | 3.257E-01 | 2.291E-01 | 2.133E-04 | 6.327E+03 | 1.312E-04 | 3.687E+03 | 1.530E-01 | 6.732E+06 | 6.087E+03 | 1.905E+01 |
| **24L** | 6.196E-01 | 1.364E+02 | 2.257E-02 | 3.564E-01 | 3.007E-01 | 1.532E-04 | 7.178E+03 | 9.612E-05 | 4.416E+03 | 1.819E-02 | 1.059E+06 | 7.005E+03 | 1.106E+01 |
| **25L** | 7.990E-01 | 3.237E+00 | 3.077E-02 | 5.937E-01 | 6.979E-01 | 1.355E-04 | 7.744E+03 | 1.075E-04 | 6.252E+03 | 4.601E-04 | 2.371E+04 | 7.644E+03 | 2.052E+00 |
| **26L** | 6.221E-01 | 2.883E+01 | 2.029E-02 | 3.598E-01 | 3.693E-01 | 9.628E-05 | 1.119E+04 | 6.033E-05 | 6.925E+03 | 2.540E-03 | 3.523E+05 | 1.098E+04 | 7.330E+00 |
| **27L** | 6.176E-01 | 7.767E+02 | 1.985E-02 | 3.543E-01 | 2.150E-01 | 1.193E-04 | 9.196E+03 | 7.379E-05 | 5.673E+03 | 9.633E-02 | 6.697E+06 | 8.992E+03 | 2.164E+01 |
| **28L** | 8.307E-01 | 3.821E+00 | 1.109E-02 | 6.458E-01 | 7.165E-01 | 6.497E-05 | 1.824E+04 | 5.293E-05 | 1.550E+04 | 2.860E-04 | 5.674E+04 | 1.743E+04 | 1.948E+00 |
| **29L** | 6.852E-01 | 1.282E+01 | 1.339E-02 | 4.347E-01 | 4.850E-01 | 8.259E-05 | 1.376E+04 | 5.664E-05 | 9.420E+03 | 9.945E-04 | 1.868E+05 | 1.333E+04 | 4.251E+00 |
| **30L** | 6.476E-01 | 6.390E+01 | 2.157E-02 | 3.890E-01 | 3.724E-01 | 1.075E-04 | 9.952E+03 | 7.026E-05 | 6.400E+03 | 5.955E-03 | 7.062E+05 | 9.788E+03 | 7.212E+00 |
| **31L** | 6.420E-01 | 1.658E+01 | 2.919E-02 | 3.835E-01 | 4.328E-01 | 1.051E-04 | 9.905E+03 | 6.724E-05 | 6.409E+03 | 1.746E-03 | 1.603E+05 | 9.803E+03 | 5.336E+00 |
| **32L** | 6.472E-01 | 2.405E+01 | 1.641E-02 | 3.885E-01 | 4.093E-01 | 1.022E-04 | 1.099E+04 | 6.577E-05 | 7.184E+03 | 2.612E-03 | 2.372E+05 | 1.066E+04 | 5.969E+00 |
| **33L** | 6.620E-01 | 2.305E+01 | 1.980E-02 | 4.058E-01 | 4.231E-01 | 1.271E-04 | 8.670E+03 | 8.339E-05 | 5.813E+03 | 3.294E-03 | 1.717E+05 | 8.457E+03 | 5.585E+00 |
| **34L** | 7.482E-01 | 5.289E+00 | 1.812E-02 | 5.182E-01 | 6.096E-01 | 1.085E-04 | 1.014E+04 | 8.105E-05 | 7.629E+03 | 6.104E-04 | 4.970E+04 | 9.905E+03 | 2.690E+00 |
| **35L** | 7.594E-01 | 3.935E+00 | 1.784E-02 | 5.341E-01 | 6.450E-01 | 1.235E-04 | 9.064E+03 | 9.370E-05 | 6.909E+03 | 4.791E-04 | 3.557E+04 | 8.819E+03 | 2.403E+00 |
| **36L** | 6.336E-01 | 1.157E+03 | 2.017E-02 | 3.716E-01 | 1.961E-01 | 1.141E-04 | 9.612E+03 | 7.108E-05 | 6.249E+03 | 1.542E-01 | 8.935E+06 | 9.376E+03 | 2.599E+01 |
| **37L** | 6.435E-01 | 2.712E+01 | 2.163E-02 | 3.838E-01 | 3.901E-01 | 1.255E-04 | 8.652E+03 | 8.000E-05 | 5.637E+03 | 3.781E-03 | 2.052E+05 | 8.472E+03 | 6.569E+00 |
| **38L** | 7.477E-01 | 7.095E+00 | 1.696E-02 | 5.176E-01 | 5.783E-01 | 1.175E-04 | 9.809E+03 | 8.582E-05 | 7.589E+03 | 9.145E-04 | 5.874E+04 | 9.430E+03 | 2.990E+00 |
| **39L** | 8.408E-01 | 2.597E+00 | 1.048E-02 | 6.626E-01 | 7.549E-01 | 8.218E-05 | 1.518E+04 | 6.755E-05 | 1.303E+04 | 2.549E-04 | 3.313E+04 | 1.432E+04 | 1.754E+00 |
| **40L** | 6.286E-01 | 1.651E+02 | 2.444E-02 | 3.664E-01 | 2.709E-01 | 1.208E-04 | 8.836E+03 | 7.706E-05 | 5.500E+03 | 1.775E-02 | 1.578E+06 | 8.693E+03 | 1.362E+01 |
| **41L** | 6.631E-01 | 4.366E+01 | 1.865E-02 | 4.070E-01 | 3.936E-01 | 1.229E-04 | 9.080E+03 | 8.218E-05 | 5.999E+03 | 4.965E-03 | 4.111E+05 | 8.831E+03 | 6.454E+00 |
| **42L** | 5.962E-01 | 5.036E+02 | 3.263E-02 | 3.305E-01 | 2.098E-01 | 1.637E-04 | 6.452E+03 | 9.585E-05 | 3.948E+03 | 8.619E-02 | 2.995E+06 | 6.358E+03 | 2.272E+01 |
| **43L** | 7.072E-01 | 8.579E+00 | 1.681E-02 | 4.629E-01 | 5.291E-01 | 1.107E-04 | 1.008E+04 | 7.834E-05 | 7.120E+03 | 8.601E-04 | 9.762E+04 | 9.813E+03 | 3.570E+00 |
| **44L** | 6.987E-01 | 1.005E+01 | 1.229E-02 | 4.514E-01 | 5.081E-01 | 7.280E-05 | 1.571E+04 | 5.166E-05 | 1.083E+04 | 6.338E-04 | 1.770E+05 | 1.521E+04 | 3.873E+00 |
| **45L** | 7.571E-01 | 9.116E+00 | 2.385E-02 | 5.317E-01 | 5.566E-01 | 2.188E-04 | 6.369E+03 | 1.715E-04 | 4.999E+03 | 1.857E-03 | 4.813E+04 | 6.132E+03 | 3.227E+00 |
| **46L** | 7.656E-01 | 4.909E+00 | 1.329E-02 | 5.433E-01 | 6.316E-01 | 7.859E-05 | 1.446E+04 | 6.038E-05 | 1.100E+04 | 3.699E-04 | 7.550E+04 | 1.401E+04 | 2.506E+00 |
| **47L** | 7.644E-01 | 4.252E+00 | 1.229E-02 | 5.414E-01 | 6.450E-01 | 8.817E-05 | 1.330E+04 | 6.645E-05 | 1.031E+04 | 4.345E-04 | 4.929E+04 | 1.279E+04 | 2.403E+00 |
| **48L** | 6.549E-01 | 4.534E+01 | 1.506E-02 | 3.974E-01 | 3.888E-01 | 1.295E-04 | 9.108E+03 | 8.377E-05 | 6.072E+03 | 6.907E-03 | 3.421E+05 | 8.729E+03 | 6.616E+00 |
| **49L** | 6.610E-01 | 1.754E+01 | 1.532E-02 | 4.046E-01 | 4.335E-01 | 9.487E-05 | 1.186E+04 | 6.342E-05 | 7.794E+03 | 1.437E-03 | 2.311E+05 | 1.152E+04 | 5.320E+00 |
| **50L** | 7.393E-01 | 5.730E+00 | 1.333E-02 | 5.057E-01 | 5.926E-01 | 8.602E-05 | 1.335E+04 | 6.368E-05 | 9.887E+03 | 4.925E-04 | 7.394E+04 | 1.291E+04 | 2.847E+00 |
| **51L** | 6.363E-01 | 3.323E+02 | 2.697E-02 | 3.751E-01 | 2.544E-01 | 1.432E-04 | 8.032E+03 | 9.233E-05 | 5.250E+03 | 4.305E-02 | 2.618E+06 | 7.782E+03 | 1.545E+01 |
| **52L** | 8.406E-01 | 2.564E+00 | 9.747E-03 | 6.620E-01 | 7.589E-01 | 7.359E-05 | 1.693E+04 | 6.129E-05 | 1.437E+04 | 1.953E-04 | 4.223E+04 | 1.603E+04 | 1.736E+00 |
| **53L** | 6.270E-01 | 1.406E+02 | 2.520E-02 | 3.645E-01 | 2.984E-01 | 1.389E-04 | 7.844E+03 | 8.553E-05 | 5.048E+03 | 2.246E-02 | 8.970E+05 | 7.659E+03 | 1.123E+01 |
| **55L** | 6.704E-01 | 3.974E+01 | 2.454E-02 | 4.157E-01 | 3.855E-01 | 1.304E-04 | 8.212E+03 | 8.744E-05 | 5.544E+03 | 4.830E-03 | 3.363E+05 | 8.072E+03 | 6.728E+00 |
| **57L** | 6.768E-01 | 2.390E+01 | 1.786E-02 | 4.238E-01 | 4.400E-01 | 8.817E-05 | 1.231E+04 | 6.048E-05 | 8.238E+03 | 1.969E-03 | 3.027E+05 | 1.207E+04 | 5.166E+00 |
| **58L** | 6.007E-01 | 8.582E+02 | 2.158E-02 | 3.357E-01 | 1.968E-01 | 1.890E-04 | 6.275E+03 | 1.175E-04 | 3.801E+03 | 1.509E-01 | 5.021E+06 | 6.090E+03 | 2.582E+01 |
| **59L** | 6.405E-01 | 3.384E+02 | 1.655E-02 | 3.806E-01 | 2.902E-01 | 1.292E-04 | 8.852E+03 | 8.290E-05 | 5.677E+03 | 4.690E-02 | 2.713E+06 | 8.560E+03 | 1.187E+01 |
| **60L** | 6.566E-01 | 4.023E+01 | 1.934E-02 | 3.995E-01 | 3.946E-01 | 1.623E-04 | 6.979E+03 | 1.056E-04 | 4.628E+03 | 7.776E-03 | 2.238E+05 | 6.767E+03 | 6.423E+00 |
| **61L** | 6.487E-01 | 1.141E+02 | 2.166E-02 | 3.897E-01 | 3.240E-01 | 1.234E-04 | 8.859E+03 | 7.881E-05 | 5.890E+03 | 1.448E-02 | 9.197E+05 | 8.656E+03 | 9.527E+00 |
| **62L** | 6.444E-01 | 2.612E+01 | 2.843E-02 | 3.850E-01 | 3.912E-01 | 1.171E-04 | 8.950E+03 | 7.546E-05 | 5.777E+03 | 3.080E-03 | 2.280E+05 | 8.839E+03 | 6.535E+00 |
| **63L** | 7.936E-01 | 4.264E+00 | 1.145E-02 | 5.854E-01 | 6.727E-01 | 7.544E-05 | 1.564E+04 | 5.925E-05 | 1.255E+04 | 3.470E-04 | 5.938E+04 | 1.504E+04 | 2.209E+00 |
| **65L** | 6.737E-01 | 2.355E+02 | 1.345E-02 | 4.200E-01 | 3.908E-01 | 2.227E-04 | 7.564E+03 | 1.469E-04 | 5.264E+03 | 4.726E-02 | 1.217E+06 | 6.973E+03 | 6.547E+00 |
| **66L** | 7.005E-01 | 5.462E+01 | 1.352E-02 | 4.537E-01 | 4.197E-01 | 1.145E-04 | 1.054E+04 | 8.359E-05 | 7.147E+03 | 4.382E-03 | 7.234E+05 | 1.013E+04 | 5.677E+00 |
| **70L** | 7.341E-01 | 1.061E+01 | 2.053E-02 | 4.987E-01 | 5.316E-01 | 1.728E-04 | 6.611E+03 | 1.249E-04 | 4.944E+03 | 2.263E-03 | 5.580E+04 | 6.385E+03 | 3.538E+00 |
| **71L** | 6.677E-01 | 4.404E+01 | 1.412E-02 | 4.124E-01 | 3.980E-01 | 1.001E-04 | 1.156E+04 | 6.456E-05 | 8.006E+03 | 6.054E-03 | 3.474E+05 | 1.114E+04 | 6.314E+00 |
| **72L** | 7.943E-01 | 4.013E+00 | 1.368E-02 | 5.869E-01 | 6.666E-01 | 9.607E-05 | 1.213E+04 | 7.597E-05 | 9.710E+03 | 3.974E-04 | 4.642E+04 | 1.169E+04 | 2.250E+00 |
| **73L** | 7.307E-01 | 5.444E+00 | 1.993E-02 | 4.939E-01 | 5.873E-01 | 1.098E-04 | 9.913E+03 | 7.996E-05 | 7.298E+03 | 6.232E-04 | 5.141E+04 | 9.706E+03 | 2.898E+00 |
| **74L** | 6.385E-01 | 1.806E+02 | 1.920E-02 | 3.787E-01 | 3.064E-01 | 8.530E-05 | 1.255E+04 | 5.566E-05 | 7.842E+03 | 1.334E-02 | 2.498E+06 | 1.234E+04 | 1.065E+01 |
| **75L** | 5.444E-01 | 1.902E+03 | 3.732E-02 | 2.794E-01 | 1.515E-01 | 2.267E-04 | 4.720E+03 | 1.201E-04 | 2.667E+03 | 4.049E-01 | 9.029E+06 | 4.634E+03 | 4.355E+01 |
