# Supplementary material for: A simple model for glioma grading based on texture analysis applied to conventional brain MRI
Source: PLoS One. 2020 May 15;15(5):e0228972. doi: 10.1371/journal.pone.0228972 (PMC7228074; doi:10.1371/journal.pone.0228972)
Supplement: S9 Table — (DOCX) [file pone.0228972.s009.docx]

| **Reference** | **Feature** | **MRI^reg^** |
| --- | --- | --- |
| 1 | *F*_szm.sze_ | T_1Gd_^1^ |
| 2 | *F*_szm.lze_ | T_1Gd_^1^ |
| 3 | *F*_szm.glnu_ | T_1Gd_^1^ |
| 4 | *F*_szm.zsnu_ | T_1Gd_^1^ |
| 5 | *F*_szm.z.perc_ | T_1Gd_^1^ |
| 6 | *F*_szm.lgze_ | T_1Gd_^1^ |
| 7 | *F*_szm.hgze_ | T_1Gd_^1^ |
| 8 | *F*_szm.szlge_ | T_1Gd_^1^ |
| 9 | *F*_szm.szhge_ | T_1Gd_^1^ |
| 10 | *F*_szm.lzlge_ | T_1Gd_^1^ |
| 11 | *F*_szm.lzhge_ | T_1Gd_^1^ |
| 12 | *F*_szm.gl.var_ | T_1Gd_^1^ |
| 13 | *F*_szm.zs.var_ | T_1Gd_^1^ |
| 14 | *F*_szm.sze_ | T_2_^1^ |
| 15 | *F*_szm.lze_ | T_2_^1^ |
| 16 | *F*_szm.glnu_ | T_2_^1^ |
| 17 | *F*_szm.zsnu_ | T_2_^1^ |
| 18 | *F*_szm.z.perc_ | T_2_^1^ |
| 19 | *F*_szm.lgze_ | T_2_^1^ |
| 20 | *F*_szm.hgze_ | T_2_^1^ |
| 21 | *F*_szm.szlge_ | T_2_^1^ |
| 22 | *F*_szm.szhge_ | T_2_^1^ |
| 23 | *F*_szm.lzlge_ | T_2_^1^ |
| 24 | *F*_szm.lzhge_ | T_2_^1^ |
| 25 | *F*_szm.gl.var_ | T_2_^1^ |
| 26 | *F*_szm.zs.var_ | T_2_^1^ |

| **Combination 1: T_1Gd_^1^** | | | | | | | | | | | | | | | | |  |
| --- | --- | --- | --- | --- | --- | --- | --- | --- | --- | --- | --- | --- | --- | --- | --- | --- | --- |
| **Number of ordered features: 1** | | |  |  |  |  |  |  |  |  |  |  |  |  |  |  |  |
| **Reference** | 1 | cte |  |  |  |  |  |  |  |  |  |  |  |  |  |  |  |
| **Coeficient** | 6.548E+01 | -4.698E+01 |  |  |  |  |  |  |  |  |  |  |  |  |  |  |  |
|  |  |  |  |  |  |  |  |  |  |  |  |  |  |  |  |  |  |
| **Number of ordered features: 2** | | | |  |  |  |  |  |  |  |  |  |  |  |  |  |  |
| **Reference** | 1 | 4 | cte |  |  |  |  |  |  |  |  |  |  |  |  |  |  |
| **Coeficient** | 9.814E+01 | -2.378E+01 | -5.886E+01 |  |  |  |  |  |  |  |  |  |  |  |  |  |  |
|  |  |  |  |  |  |  |  |  |  |  |  |  |  |  |  |  |  |
| **Number of ordered features: 3** | | | | |  |  |  |  |  |  |  |  |  |  |  |  |  |
| **Reference** | 1 | 4 | 9 | cte |  |  |  |  |  |  |  |  |  |  |  |  |  |
| **Coeficient** | 1.277E+02 | -5.958E+01 | 2.126E-03 | -7.049E+01 |  |  |  |  |  |  |  |  |  |  |  |  |  |
|  |  |  |  |  |  |  |  |  |  |  |  |  |  |  |  |  |  |
| **Number of ordered features: 4** | | | | | |  |  |  |  |  |  |  |  |  |  |  |  |
| **Reference** | 1 | 4 | 9 | 5 | cte |  |  |  |  |  |  |  |  |  |  |  |  |
| **Coeficient** | 2.091E+02 | -6.679E+01 | 2.089E-03 | -3.135E+01 | -1.106E+02 |  |  |  |  |  |  |  |  |  |  |  |  |
|  |  |  |  |  |  |  |  |  |  |  |  |  |  |  |  |  |  |
| **Number of ordered features: 5** | | | | | | |  |  |  |  |  |  |  |  |  |  |  |
| **Reference** | 1 | 4 | 9 | 5 | 13 | cte |  |  |  |  |  |  |  |  |  |  |  |
| **Coeficient** | 2.018E+02 | -6.138E+01 | 2.091E-03 | -3.159E+01 | -1.932E-03 | -1.079E+02 |  |  |  |  |  |  |  |  |  |  |  |

| **Combination 3: T_2_^1^** | | | | | | | | | | |
| --- | --- | --- | --- | --- | --- | --- | --- | --- | --- | --- |
| **Number of ordered features: 1** | | |  |  |  |  |  |  |  |  |
| **Reference** | 18 | cte |  |  |  |  |  |  |  |  |
| **Coeficient** | 3.492E+01 | -1.732E+01 |  |  |  |  |  |  |  |  |
|  |  |  |  |  |  |  |  |  |  |  |
| **Number of ordered features: 2** | | | |  |  |  |  |  |  |  |
| **Reference** | 18 | 26 | cte |  |  |  |  |  |  |  |
| **Coeficient** | 3.104E+01 | -1.453E-01 | 1.451E+01 |  |  |  |  |  |  |  |
|  |  |  |  |  |  |  |  |  |  |  |
| **Number of ordered features: 3** | | | | |  |  |  |  |  |  |
| **Reference** | 18 | 26 | 23 | cte |  |  |  |  |  |  |
| **Coeficient** | 2.958E+01 | -1.703E-01 | -9.476E-01 | -1.357E+01 |  |  |  |  |  |  |
|  |  |  |  |  |  |  |  |  |  |  |
| **Number of ordered features: 4** | | | | | |  |  |  |  |  |
| **Reference** | 18 | 26 | 23 | 15 | cte |  |  |  |  |  |
| **Coeficient** | 2.814E+01 | -2.828E-01 | -1.592E+01 | 1.777E-03 | -1.228E+01 |  |  |  |  |  |
|  |  |  |  |  |  |  |  |  |  |  |
| **Number of ordered features: 5** | | | | | | |  |  |  |  |
| **Reference** | 18 | 26 | 23 | 15 | 24 | cte |  |  |  |  |
| **Coeficient** | 2.381E+01 | -4.793E-01 | -3.833E+01 | 7.508E-03 | -2.315E-07 | -8.943E+00 |  |  |  |  |
|  |  |  |  |  |  |  |  |  |  |  |
| **Number of ordered features: 6** | | | | | | | |  |  |  |
| **Reference** | 18 | 26 | 23 | 15 | 24 | 14 | cte |  |  |  |
| **Coeficient** | 4.359E+01 | -4.172E-01 | -3.254E+01 | 6.276E-03 | -1.415E-07 | -4.344E+01 | 1.221E+01 |  |  |  |
|  |  |  |  |  |  |  |  |  |  |  |
| **Number of ordered features: 7** | | | | | | | | |  |  |
| **Reference** | 18 | 26 | 23 | 15 | 24 | 14 | 17 | cte |  |  |
| **Coeficient** | 7.134E+01 | 5.239E-01 | -8.022E+00 | 8.535E-04 | -4.006E-08 | 4.604E+02 | -3.714E+02 | -1.892E+02 |  |  |

| **Combination 6: T_1Gd_^1^ – T_2_^1^** | | | | | | | | | | | | | | | | |  |
| --- | --- | --- | --- | --- | --- | --- | --- | --- | --- | --- | --- | --- | --- | --- | --- | --- | --- |
| **Number of ordered features: 1** | | |  |  |  |  |  |  |  |  |  |  |  |  |  |  |  |
| **Reference** | 18 | cte |  |  |  |  |  |  |  |  |  |  |  |  |  |  |  |
| **Coeficient** | 3.525E+01 | -1.751E+01 |  |  |  |  |  |  |  |  |  |  |  |  |  |  |  |
|  |  |  |  |  |  |  |  |  |  |  |  |  |  |  |  |  |  |
| **Number of ordered features: 2** | | | |  |  |  |  |  |  |  |  |  |  |  |  |  |  |
| **Reference** | 18 | 26 | cte |  |  |  |  |  |  |  |  |  |  |  |  |  |  |
| **Coeficient** | 3.177E+01 | -1.317E-01 | -1.498E+01 |  |  |  |  |  |  |  |  |  |  |  |  |  |  |
|  |  |  |  |  |  |  |  |  |  |  |  |  |  |  |  |  |  |
| **Number of ordered features: 3** | | | | |  |  |  |  |  |  |  |  |  |  |  |  |  |
| **Reference** | 18 | 26 | 23 | cte |  |  |  |  |  |  |  |  |  |  |  |  |  |
| **Coeficient** | 3.150E+01 | -1.328E-01 | -7.192E-01 | -1.483E+01 |  |  |  |  |  |  |  |  |  |  |  |  |  |
|  |  |  |  |  |  |  |  |  |  |  |  |  |  |  |  |  |  |
| **Number of ordered features: 4** | | | | | |  |  |  |  |  |  |  |  |  |  |  |  |
| **Reference** | 18 | 26 | 23 | 15 | cte |  |  |  |  |  |  |  |  |  |  |  |  |
| **Coeficient** | 3.489E+01 | -5.250E-02 | -9.574E+00 | 8.581E-04 | -1.714E+01 |  |  |  |  |  |  |  |  |  |  |  |  |
|  |  |  |  |  |  |  |  |  |  |  |  |  |  |  |  |  |  |
| **Number of ordered features: 5** | | | | | | |  |  |  |  |  |  |  |  |  |  |  |
| **Reference** | 18 | 26 | 23 | 15 | 4 | cte |  |  |  |  |  |  |  |  |  |  |  |
| **Coeficient** | 1.070E+01 | -5.768E-01 | -4.860E+00 | 1.309E-03 | 3.212E+01 | -1.735E+01 |  |  |  |  |  |  |  |  |  |  |  |
|  |  |  |  |  |  |  |  |  |  |  |  |  |  |  |  |  |  |
| **Number of ordered features: 6** | | | | | | | |  |  |  |  |  |  |  |  |  |  |
| **Reference** | 18 | 26 | 23 | 15 | 4 | 1 | cte |  |  |  |  |  |  |  |  |  |  |
| **Coeficient** | 1.270E+01 | -5.324E-01 | -1.365E+00 | 8.992E-04 | -1.109E+02 | 1.890E+02 | -8.506E+01 |  |  |  |  |  |  |  |  |  |  |
|  |  |  |  |  |  |  |  |  |  |  |  |  |  |  |  |  |  |
| **Number of ordered features: 7** | | | | | | | | |  |  |  |  |  |  |  |  |  |
| **Reference** | 18 | 26 | 23 | 15 | 4 | 1 | 14 | cte |  |  |  |  |  |  |  |  |  |
| **Coeficient** | 4.454E+01 | -5.111E-01 | -9.474E+00 | 2.804E-03 | -1.623E+02 | 2.566E+02 | -7.297E+01 | -7.200E+01 |  |  |  |  |  |  |  |  |  |
|  |  |  |  |  |  |  |  |  |  |  |  |  |  |  |  |  |  |
| **Number of ordered features: 8** | | | | | | | | | |  |  |  |  |  |  |  |  |
| **Reference** | 18 | 26 | 23 | 15 | 4 | 1 | 14 | 17 | cte |  |  |  |  |  |  |  |  |
| **Coeficient** | 6.122E+01 | 3.518E-01 | 2.742E+00 | -2.287E-04 | -3.721E+01 | 8.763E+01 | 4.431E+02 | -3.647E+02 | -2.189E+02 |  |  |  |  |  |  |  |  |
|  |  |  |  |  |  |  |  |  |  |  |  |  |  |  |  |  |  |
| **Number of ordered features: 9** | | | | | | | | | | |  |  |  |  |  |  |  |
| **Reference** | 18 | 26 | 23 | 15 | 4 | 1 | 14 | 17 | 24 | cte |  |  |  |  |  |  |  |
| **Coeficient** | 5.485E+01 | 2.474E-01 | -1.011E+01 | 2.687E-03 | -3.957E+01 | 9.070E+01 | 4.326E+02 | -3.509E+02 | -1.189E-07 | -2.153E+02 |  |  |  |  |  |  |  |
|  |  |  |  |  |  |  |  |  |  |  |  |  |  |  |  |  |  |
| **Number of ordered features: 10** | | | | | | | | | | | |  |  |  |  |  |  |
| **Reference** | 18 | 26 | 23 | 15 | 4 | 1 | 14 | 17 | 24 | 16 | cte |  |  |  |  |  |  |
| **Coeficient** | 4.992E+01 | -9.989E-02 | -1.947E+01 | 5.017E-03 | -7.384E+00 | 6.814E+01 | 1.997E+02 | -2.059E+02 | -1.642E-07 | -3.396E+02 | -1.074E+02 |  |  |  |  |  |  |
|  |  |  |  |  |  |  |  |  |  |  |  |  |  |  |  |  |  |
| **Number of ordered features: 11** | | | | | | | | | | | | |  |  |  |  |  |
| **Reference** | 18 | 26 | 23 | 15 | 4 | 1 | 14 | 17 | 24 | 16 | 9 | cte |  |  |  |  |  |
| **Coeficient** | 4.617E+01 | -8.663E-02 | -2.858E+01 | 4.938E-03 | -2.315E+01 | 8.553E+01 | 1.619E+02 | -1.822E+02 | -1.206E-07 | -4.040E+02 | 1.598E-03 | -9.926E+01 |  |  |  |  |  |
|  |  |  |  |  |  |  |  |  |  |  |  |  |  |  |  |  |  |
| **Number of ordered features: 12** | | | | | | | | | | | | | |  |  |  |  |
| **Reference** | 18 | 26 | 23 | 15 | 4 | 1 | 14 | 17 | 24 | 16 | 9 | 22 | cte |  |  |  |  |
| **Coeficient** | 5.401E+01 | 6.932E-02 | -4.287E+01 | 1.149E-02 | -4.329E+01 | 1.239E+02 | 9.639E+01 | -1.666E+02 | -4.367E-07 | -3.348E+02 | 1.913E-03 | 6.217E-04 | -9.238E+01 |  |  |  |  |
|  |  |  |  |  |  |  |  |  |  |  |  |  |  |  |  |  |  |
| **Number of ordered features: 13** | | | | | | | | | | | | | | |  |  |  |
| **Reference** | 18 | 26 | 23 | 15 | 4 | 1 | 14 | 17 | 24 | 16 | 9 | 22 | 5 | cte |  |  |  |
| **Coeficient** | 7.290E+01 | 3.371E-01 | -1.408E+01 | 7.480E-03 | -8.661E+01 | 2.609E+02 | 5.979E+01 | -1.439E+02 | -3.713E-07 | -2.082E+02 | 1.144E-03 | 4.360E-04 | -3.857E+01 | -1.443E+02 |  |  |  |
|  |  |  |  |  |  |  |  |  |  |  |  |  |  |  |  |  |  |
| **Number of ordered features: 14** | | | | | | | | | | | | | | | |  |  |
| **Reference** | 18 | 26 | 23 | 15 | 4 | 1 | 14 | 17 | 24 | 16 | 9 | 22 | 5 | 13 | cte |  |  |
| **Coeficient** | 7.490E+01 | 3.545E-01 | -1.446E+01 | 7.287E-03 | -5.822E+01 | 2.224E+02 | 6.692E+01 | -1.507E+02 | -3.600E-07 | -2.065E+02 | 1.152E-03 | 4.213E-04 | -4.008E+01 | -9.211E-03 | -1.323E+02 |  |  |
|  |  |  |  |  |  |  |  |  |  |  |  |  |  |  |  |  |  |
|  |  |  |  |  |  |  |  |  |  |  |  |  |  |  |  |  |  |
| **Number of ordered features: 15** | | | | | | | | | | | | | | | | |  |
| **Reference** | 18 | 26 | 23 | 15 | 4 | 1 | 14 | 17 | 24 | 16 | 9 | 22 | 5 | 13 | 20 | cte |  |
| **Coeficient** | 7.167E+01 | 1.344E-01 | -3.406E+01 | 9.501E-03 | 2.889E+00 | 1.288E+02 | 1.151E+02 | -2.446E+02 | -3.872E-07 | -1.435E+02 | 1.099E-03 | 5.440E-03 | -4.073E+01 | -2.380E-02 | -3.520E-03 | -8.297E+01 |  |
|  |  |  |  |  |  |  |  |  |  |  |  |  |  |  |  |  |  |
| **Number of ordered features: 16** | | | | | | | | | | | | | | | | | |
| **Reference** | 18 | 26 | 23 | 15 | 4 | 1 | 14 | 17 | 24 | 16 | 9 | 22 | 5 | 13 | 20 | 25 | cte |
| **Coeficient** | 7.656E+01 | 2.635E-01 | -2.954E+01 | 8.586E-03 | 4.178E+01 | 7.977E+01 | 1.101E+02 | -2.336E+02 | -3.613E-07 | -8.293E+01 | 1.179E-03 | 4.145E-03 | -4.126E+01 | -2.419E-02 | 6.719E-04 | -3.356E-03 | -7.287E+01 |
