# Supplementary material for: A simple model for glioma grading based on texture analysis applied to conventional brain MRI
Source: PLoS One. 2020 May 15;15(5):e0228972. doi: 10.1371/journal.pone.0228972 (PMC7228074; doi:10.1371/journal.pone.0228972)
Supplement: S10 Table — (DOCX) [file pone.0228972.s010.docx]

| **Reduced models** | | | | | | |
| --- | --- | --- | --- | --- | --- | --- |
|  |  |  |  |  |  |  |
| **Order** | 1 | cte |  |  |  |  |
| **Reference** | 18 |  |  |  |  |  |
| **Coeficient** | 3.525E+01 | -1.751E+01 |  |  |  |  |
|  |  |  |  |  |  |  |
| **Order** | 2 | cte |  |  |  |  |
| **Reference** | 26 |  |  |  |  |  |
| **Coeficient** | -9.568E-01 | 5.888E+00 |  |  |  |  |
|  |  |  |  |  |  |  |
| **Order** | 3 | cte |  |  |  |  |
| **Reference** | 23 |  |  |  |  |  |
| **Coeficient** | -3.530E+01 | 8.002E-01 |  |  |  |  |
|  |  |  |  |  |  |  |
| **Order** | 4 | cte |  |  |  |  |
| **Reference** | 15 |  |  |  |  |  |
| **Coeficient** | -2.823E-03 | 6.935E-01 |  |  |  |  |
|  |  |  |  |  |  |  |
| **Order** | 5 | cte |  |  |  |  |
| **Reference** | 4 |  |  |  |  |  |
| **Coeficient** | 5.600E+01 | -2.679E+01 |  |  |  |  |
|  |  |  |  |  |  |  |
| **Order** | 1 | 2 | cte |  |  |  |
| **Reference** | 18 | 26 |  |  |  |  |
| **Coeficient** | 3.177E+01 | -1.317E-01 | -1.498E+01 |  |  |  |
|  |  |  |  |  |  |  |
| **Order** | 1 | 3 | cte |  |  |  |
| **Reference** | 18 | 23 |  |  |  |  |
| **Coeficient** | 3.451E+01 | -3.700E+00 | -1.704E+01 |  |  |  |
|  |  |  |  |  |  |  |
| **Order** | 1 | 4 | cte |  |  |  |
| **Reference** | 18 | 15 |  |  |  |  |
| **Coeficient** | 3.623E+01 | 2.437E-05 | -1.807E+01 |  |  |  |
|  |  |  |  |  |  |  |
| **Order** | 1 | 5 | cte |  |  |  |
| **Reference** | 18 | 4 |  |  |  |  |
| **Coeficient** | 2.652E+01 | 2.946E+01 | -2.718E+01 |  |  |  |
|  |  |  |  |  |  |  |
| **Order** | 2 | 3 | cte |  |  |  |
| **Reference** | 26 | 23 |  |  |  |  |
| **Coeficient** | -1.036E+00 | 9.234E+00 | 6.174E+00 |  |  |  |
|  |  |  |  |  |  |  |
| **Order** | 2 | 4 | cte |  |  |  |
| **Reference** | 26 | 15 |  |  |  |  |
| **Coeficient** | -1.106E+00 | 1.615E-03 | 6.390E+00 |  |  |  |
|  |  |  |  |  |  |  |
| **Order** | 2 | 5 | cte |  |  |  |
| **Reference** | 26 | 4 |  |  |  |  |
| **Coeficient** | -6.962E-01 | 3.825E+01 | -1.408E+01 |  |  |  |
|  |  |  |  |  |  |  |
| **Order** | 3 | 4 | cte |  |  |  |
| **Reference** | 23 | 15 |  |  |  |  |
| **Coeficient** | -3.317E+01 | -2.332E-04 | 7.995E-01 |  |  |  |
|  |  |  |  |  |  |  |
| **Order** | 3 | 5 | cte |  |  |  |
| **Reference** | 23 | 4 |  |  |  |  |
| **Coeficient** | -1.906E+01 | 5.223E+01 | -2.457E+01 |  |  |  |
|  |  |  |  |  |  |  |
| **Order** | 4 | 5 | cte |  |  |  |
| **Reference** | 15 | 4 |  |  |  |  |
| **Coeficient** | -1.635E-03 | 5.289E+01 | -2.491E+01 |  |  |  |
|  |  |  |  |  |  |  |
| **Order** | 1 | 2 | 3 | cte |  |  |
| **Reference** | 18 | 26 | 23 |  |  |  |
| **Coeficient** | 3.150E+01 | -1.328E-01 | -7.192E-01 | -1.483E+01 |  |  |
|  |  |  |  |  |  |  |
| **Order** | 1 | 2 | 4 | cte |  |  |
| **Reference** | 18 | 26 | 15 |  |  |  |
| **Coeficient** | 3.514E+01 | -5.539E-02 | 1.551E-04 | -1.728E+01 |  |  |
|  |  |  |  |  |  |  |
| **Order** | 1 | 2 | 5 | cte |  |  |
| **Reference** | 18 | 26 | 4 |  |  |  |
| **Coeficient** | 1.369E+01 | -4.103E-01 | 3.184E+01 | -1.950E+01 |  |  |
|  |  |  |  |  |  |  |
| **Order** | 1 | 3 | 4 | cte |  |  |
| **Reference** | 18 | 23 | 15 |  |  |  |
| **Coeficient** | 3.590E+01 | -9.804E+00 | 7.496E-04 | -1.788E+01 |  |  |
|  |  |  |  |  |  |  |
| **Order** | 1 | 3 | 5 | cte |  |  |
| **Reference** | 18 | 23 | 4 |  |  |  |
| **Coeficient** | 2.562E+01 | -4.057E+00 | 2.957E+01 | -2.669E+01 |  |  |
|  |  |  |  |  |  |  |
| **Order** | 1 | 4 | 5 | cte |  |  |
| **Reference** | 18 | 15 | 4 |  |  |  |
| **Coeficient** | 2.575E+01 | -3.079E-04 | 2.965E+01 | -2.681E+01 |  |  |
|  |  |  |  |  |  |  |
| **Order** | 2 | 3 | 4 | cte |  |  |
| **Reference** | 26 | 23 | 15 |  |  |  |
| **Coeficient** | -1.088E+00 | -1.769E+01 | 2.918E-03 | 6.351E+00 |  |  |
|  |  |  |  |  |  |  |
| **Order** | 2 | 3 | 5 | cte |  |  |
| **Reference** | 26 | 23 | 4 |  |  |  |
| **Coeficient** | -7.852E-01 | 9.601E+00 | 3.788E+01 | -1.358E+01 |  |  |
|  |  |  |  |  |  |  |
| **Order** | 2 | 4 | 5 | cte |  |  |
| **Reference** | 26 | 15 | 4 |  |  |  |
| **Coeficient** | -8.465E-01 | 1.319E-03 | 3.693E+01 | -1.285E+01 |  |  |
|  |  |  |  |  |  |  |
| **Order** | 3 | 4 | 5 | cte |  |  |
| **Reference** | 23 | 15 | 4 |  |  |  |
| **Coeficient** | -1.225E+01 | -6.797E-04 | 5.229E+01 | -2.458E+01 |  |  |
|  |  |  |  |  |  |  |
| **Order** | 1 | 2 | 3 | 4 | cte |  |
| **Reference** | 18 | 26 | 23 | 15 |  |  |
| **Coeficient** | 3.489E+01 | -5.250E-02 | -9.574E+00 | 8.581E-04 | -1.714E+01 |  |
|  |  |  |  |  |  |  |
| **Order** | 1 | 2 | 3 | 5 | cte |  |
| **Reference** | 18 | 26 | 23 | 4 |  |  |
| **Coeficient** | 1.248E+01 | -4.938E-01 | 6.272E+00 | 3.217E+01 | -1.869E+01 |  |
|  |  |  |  |  |  |  |
| **Order** | 1 | 2 | 4 | 5 | cte |  |
| **Reference** | 18 | 26 | 15 | 4 |  |  |
| **Coeficient** | 1.089E+01 | -5.752E-01 | 9.336E-04 | 3.222E+01 | -1.752E+01 |  |
|  |  |  |  |  |  |  |
| **Order** | 1 | 3 | 4 | 5 | cte |  |
| **Reference** | 18 | 26 | 15 | 4 |  |  |
| **Coeficient** | 2.562E+01 | -4.151E+00 | 9.511E-06 | 2.956E+01 | -2.669E+01 |  |
|  |  |  |  |  |  |  |
| **Order** | 1 | 2 | 3 | 4 | 5 | cte |
| **Reference** | 18 | 26 | 23 | 15 | 4 |  |
| **Coeficient** | 1.070E+01 | -5.768E-01 | -4.860E+00 | 1.309E-03 | 3.212E+01 | -1.735E+01 |
