# Supplementary material for: A simple model for glioma grading based on texture analysis applied to conventional brain MRI
Source: PLoS One. 2020 May 15;15(5):e0228972. doi: 10.1371/journal.pone.0228972 (PMC7228074; doi:10.1371/journal.pone.0228972)
Supplement: Attachment — Submitted filename: Response to reviewers.docx [file pone.0228972.s011.docx]

Dear Editor and reviewers:

We thank you for your comments and considerations. In general reviews were really positive and enthusiastic and no major criticism to methodology, impact and results was raised. In fact, we were congratulated by reviewers which is not that common. We would like to thank them both for this.

We have addressed the minor points raised by reviewers and editor in the following manner:

1. The main criticism of reviewer 1, was exclusively: “The work can benefit from minor grammatical editing”. We have sent the manuscript to a native speaking editing company (see file: certificate.pdf attached in this resubmission). This has greatly improved the work, and we believe the paper now has very high standards in this aspect.
2. Editor asked to adapt the manuscript to PLOS ONE style format. We have done so thoroughly. Images were passed through website which checked image format and quality and passed but were re-dimensioned. Because of this in the resubmission, we attach all images and supplementary material again.
3. No laboratory specific lab protocols were used for this publication, so we did not consider the petition to load them to the journals site applicable. Therefore, no specific protocol from this work was submitted to the journal.

Please find enclosed in this re-submission:

- Revised manuscript with track changes (pdf and .tex format). This is manuscript with highlighted changes in it (yellow).
- Manuscript (pdf and .tex format). This is manuscript with changes already implemented in it.
- Certificate (pdf). Certificate of style and English formatting.

We hope all these changes are enough to get this work accepted in your journal.

Yours,

Dr. Benito de Celis Alonso
